# Identification of Ferroptosis-Related lncRNA Pairs for Predicting the Prognosis of Head and Neck Squamous Cell Carcinoma

**DOI:** 10.1155/2022/7602482

**Published:** 2022-07-20

**Authors:** Congxiao Wu, Fei Liu, Haixu Chen, Qin Liu, Chao Song, Kang Cheng, Zhiyong Gao, Cheng Fan

**Affiliations:** ^1^Department of Plastic Surgery, Shenzhen Qianhai Taikang Hospital, Shenzhen 518000, Guangdong, China; ^2^Department of Orthopedics, The Affiliated Traditional Chinese Medicine Hospital of Southwest Medical University, Luzhou 646000, Sichuan, China; ^3^Department of Basic Medicine, Sichuan Vocational College of Health and Rehabilitation, Zigong 643000, Sichuan, China; ^4^Department of Neurosurgery, Anyue County People's Hospital, Ziyang 641300, Sichuan, China; ^5^Department of Burn and Plastic Surgery, The First People's Hospital of Ziyang, Ziyang 641300, Sichuan, China

## Abstract

**Background:**

Ferrogenesis was strongly associated with tumorigenesis and development, and activating the ferrogenic process was a novel regimen in treating cancer, especially conventional treatment-resistant cancers. The purpose of the article was to construct a ferroptosis-related long noncoding RNAs (FRlncRNAs) signature, regardless of expression levels to effectively predict prognosis and immunotherapeutic response for head and neck squamous cell carcinoma (HNSCC).

**Methods:**

The RNA-seq data for HNSCC and corresponding clinical information were obtained in the TCGA database, and ferroptosis-related genes (FRGs) were extracted in the ferroptosis database. On this basis, differentially expressed FRlncRNAs (DEFRlncRNAs) pairs were identified through coexpression analysis, differential expression analysis, and a fresh pairing algorithm. Then, a risk assessment model was established with univariate Cox, LASSO, and multivariate Cox regression analysis. Finally, we evaluated the model from various aspects, including survival status, clinicopathological characteristics, infiltration status of immune cells, immune functions, chemotherapeutic sensitivity, immune checkpoint inhibitors (ICIs)-related molecules, and N6-methyladenosine (m^6^A) mRNA status.

**Result:**

We established a signature of 11-DEFRlncRNA pairs related to the prognosis of HNSCC that had AUC values above 0.75 in the one-, three-, and five-year ROC curves, underscoring the high susceptibility and specifiability of predicting HNSCC prognosis. Survival rates were remarkably higher for the low-risk patients than for the high-risk patients, and the signature was significantly correlated with survival, clinical, T, and N stages. Finally, immune cell infiltration status, immune functions, chemotherapeutic sensitivity, and expression levels of ICIs-related and m^6^A-related molecules were statistically different among different groups.

**Conclusion:**

Our study established a novel lncRNA signature, which is independent of specific expression levels, could predict patient prognosis, and might have promising clinical applications in HNCSS.

## 1. Introduction

Worldwide, the incidence and mortality of head and neck cancer are estimated at 930,000 and 470,000, respectively [1]. Head and neck squamous cell carcinoma (HNSCC) constitutes the majority of pathological types of head and neck cancers, and its major risk contributors are alcohol consumption, cigarette smoking, and human papilloma virus infection. Despite significant advances in treatment with surgery, radiation, chemotherapy, targeted therapies, and immunotherapy, the mortality rate of HNSCC remains high [2]. High insensitivity or resistance to chemotherapy is a major cause of death in patients with advanced HNSCC [3]. Significantly, ferroptosis inducers may be an effective weapon in the treatment of various chemotherapy-resistant tumors, including HNSCC [4].

Cancer cells are usually characterized by a defect in the cell death executioner mechanism, which is a main cause of resistance to treatment. Compared to normal cells, cancer cells have an increased need for iron in order to promote growth, and this dependence on iron leads to cancer cells being more susceptible to iron-catalyzed necrosis called ferroptosis [5]. Abundant evidence has found that ferrogenesis is strongly associated with tumorigenesis and development, and activating the ferrogenic process is a new treatment regimen for cancer, especially conventional treatment-resistant cancers [6–8]. Long noncoding RNAs (lncRNAs) are the most common RNAs, which are more than 200 nucleotides in length and have no protein-coding ability [9]. A study illustrated that LINC00336 functioned through interaction with ELAVL1 as a significant inhibitor of ferroptosis in oncogenesis [10]. Mao et al. indicated that P53RRA promoted ferroptosis in cancer by nuclear sequestration of p53 [11]. One recent study revealed that GABPB1-AS1 was upregulated by erastin, inhibiting peroxidase gene expression and accumulating reactive oxygen species and cancer cell death, indicating that GABPB1-AS1 might have an essential molecular function in ferroptosis with hepatocellular carcinoma cells [12]. Some other research has suggested that lncRNAs exert their antitumor effects by modulating ferroptosis [13, 14].

Consequently, the identification of ferroptosis-related lncRNAs (FRlncRNAs) has significant implications for elucidating the specific mechanism of oncogenesis and predicting the prognosis of HNSCC. A variety of studies identified different FRlncRNAs and established signatures to predict patient prognosis with malignancies [15–18]. However, these promising signatures have a few intrinsic shortcomings that might not be clinically applicable for translation. These signatures briefly incorporate transcriptomic data and clinical data based on sequencing or microarrays. However, because of varied platforms, detection, and batch technologies, the expression level of individual genes has great variability. In this scenario, the utilization of these models would be severely limited, and they would be prone to biased diagnostic results, so their diagnostic accuracy would not be sufficient to translate into practical applications [19, 20]. Here, we built a 0-or-1 matrix based on differentially expressed FRlncRNAs (DEFRlncRNA) pairs and replaced particular transcriptome expression values with dichotomous aggregated values to eliminate bias in the transcriptome expression values obtained under diverse situations [21].

The study established a novel prognostic model on the basis of DEFRlncRNA pairs that is independent of expression level. We then assessed the model's predictive power, tumor immune infiltration, N6-methyladenosine (m^6^A) mRNA status, chemotherapeutic efficacy, and immune checkpoint inhibitors (ICIs)-related molecules. In conclusion, this signature can accurately predict patient prognosis and characterize diverse immune landscapes, which is a promising prognostic biomarker.

## 2. Materials and Methods

### 2.1. Collection of Data and Identification of DEFRlncRNAs

The RNA-seq data of HNSCC patients and corresponding clinical characteristics were extracted from The Cancer Genome Atlas (TCGA (https://tcga-data.nci.nih.gov)). Tissue sources of HNSCC in the TCGA database include mainly the oral cavity, tonsils, pharynx, and larynx. By removing duplicate or severely missing data (unknown or 0-day follow-up time and unknown survival status), valid clinical data were obtained. On the basis of GTF files in the Ensembl database, we added annotations to these RNA-seq data and then obtained the expression profiles of mRNA and lncRNA [22]. Ferroptosis-related genes (FRGs, S1 Table) were obtained from the ferroptosis database (FerrDb; https://www.zhounan.org/ferrdb) and were applied to define FRlncRNAs through a coexpression strategy [23]. Some lncRNAs were recognized as FRlncRNAs by criteria of a *p* value smaller than 0.001 and cor greater than 0.4. Finally, we utilized the R package Limma to distinguish DEFRlncRNAs from FRlncRNAs, and statistical significance was assumed as FDR < 0.05 and |log2FC| ≥ 1.5.

### 2.2. Pairing DEFRlncRNAs

The DEFRlncRNAs were separately paired, and a 0-or-1 matrix was created. If the first DEFRlncRNA expression is lower than the second one, it is scored as 0; otherwise, it is scored as 1. Since some pairs with no certain class do not precisely predict patient prognosis, these pairs were filtered out, when the amounts with an expression quantity of 0 or 1 made up less than 20% and more than 80% of all pairs, respectively.

### 2.3. Establishment of the Risk Model

Univariate Cox, LASSO, and multivariate Cox regression analyses were utilized to construct a prognostic model. The concrete risk score on each patient was computed, and the risk score formula was as follows: the time-dependent receiver-operating characteristic (ROC) curves and the area under the curve (AUC) were obtained to assess the predictive ability of the model for survival. On the basis of the maximum inflection point of the Akaike information criterion (AIC) values in the five-year ROC curve, we used it to be a cut-off point for classifying patients into high- or low-risk groups. The ROC curve and decision curve analysis (DCA) were also used to assess the precision of the model compared to the traditional clinical features. The comparison between the established models was utilized to assess the forecasting ability of the novel model [16, 24–28].

### 2.4. Validation of the Prognostic Model

The Kaplan–Meier analysis and log-rank test were applied to assess the difference in survival between different groups on the basis of the R package Limma. Next, the chi-squared test was applied to show the association between the signature and clinical characteristics. Then, the Wilcoxon signed-rank test was utilized to compute the risk score differences among different groups of these clinical characteristics. Also, univariate and multivariate Cox analyses were applied to confirm that the risk score was an independent predictor of clinical prognosis. Finally, we developed a nomogram integrating the signature and other clinical characteristics to predict the one-, three-, and five-year survival rates of patients.

### 2.5. Evaluation of Immune Cell Infiltration

To obtain accurate immune infiltration status, the widely accepted approaches to estimate immune cell infiltration were used, including TIMER, CIBERSORT, CIBERSORT-ABS, QUANTISEQ, MCPCOUNTER, XCELL, and EPIC.

### 2.6. Exploration of the Risk Assessment Model

The single-sample gene set enrichment analysis (ssGSEA) was utilized to evaluate the difference in immune function among diverse groups. Then, we investigate the expression levels of ICIs-related molecules and m^6^A-related genes and the half-inhibitory concentration (IC50) of chemotherapeutic agents in different groups.

## 3. Results

### 3.1. Identification of DEFRlncRNAs

**Figure 1:**
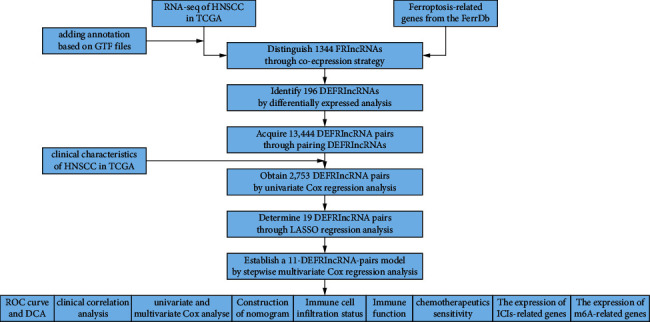
Flowchart for this article.

**Figure 2:**
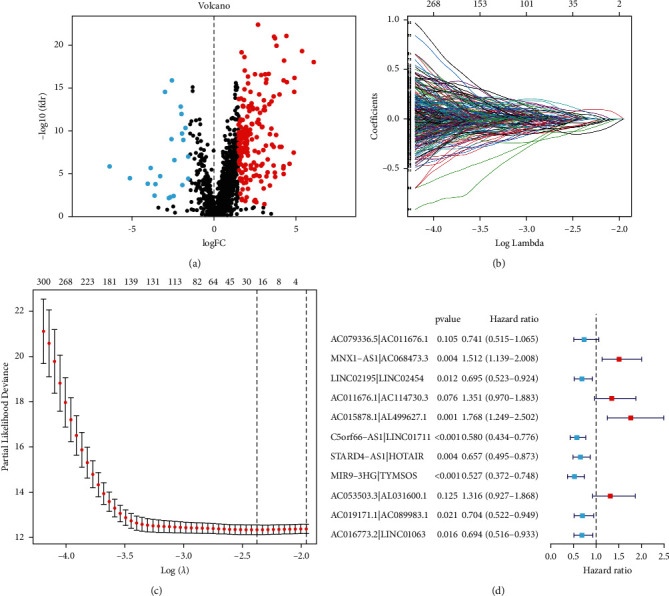
Establishment of a prognostic risk model. (a) Recognition of DEFRlncRNAs. (b) LASSO coefficient profiles. (c) Coefficient profile plot generated. (d) 11 DEFRlncRNA pairs identified by multivariate Cox stepwise regression analysis.

Figure 1 depicts a flowchart of the complete procedure. The RNA-seq data and clinical information contained 44 normal samples and 501 tumor samples in TCGA-HNSCC.  Table 1 shows the clinical features of the patients. Next, according to GTF files from Ensembl, the data were annotated and coexpression analysis between FRGs and lncRNAs was done. Finally, 1344 FRlncRNAs were distinguished by the criteria of *p* value less than 0.001 and cor more than 0.4 (Table S2), and 196 FRlncRNAs were identified as DEFRlncRNAs by the standard of FDR < 0.05 and |log2FC| ≥ 1 (Table S3), among which 174 genes were upregulated and 22 genes were downregulated (Figure 2(a)).

### 3.2. Construction of the Risk Assessment Model

13,444 DEFRlncRNA pairs were acquired through the pairing of these DEFRlncRNAs. Next, 2,753 DEFRlncRNA pairs were distinguished as statistically significant through univariate Cox regression analysis (Table S4). Next, 19 candidate DEFRlncRNA pairs were determined via the LASSO regression analysis, whose coefficient profiles and a partial likelihood deviation plot are presented in Figures 2(b) and 2(c). Finally, we utilized multivariate Cox regression analysis to distinguish 11 DEFRlncRNA pairs and establish the prognostic model (Figure 2(d)).

**Figure 3:**
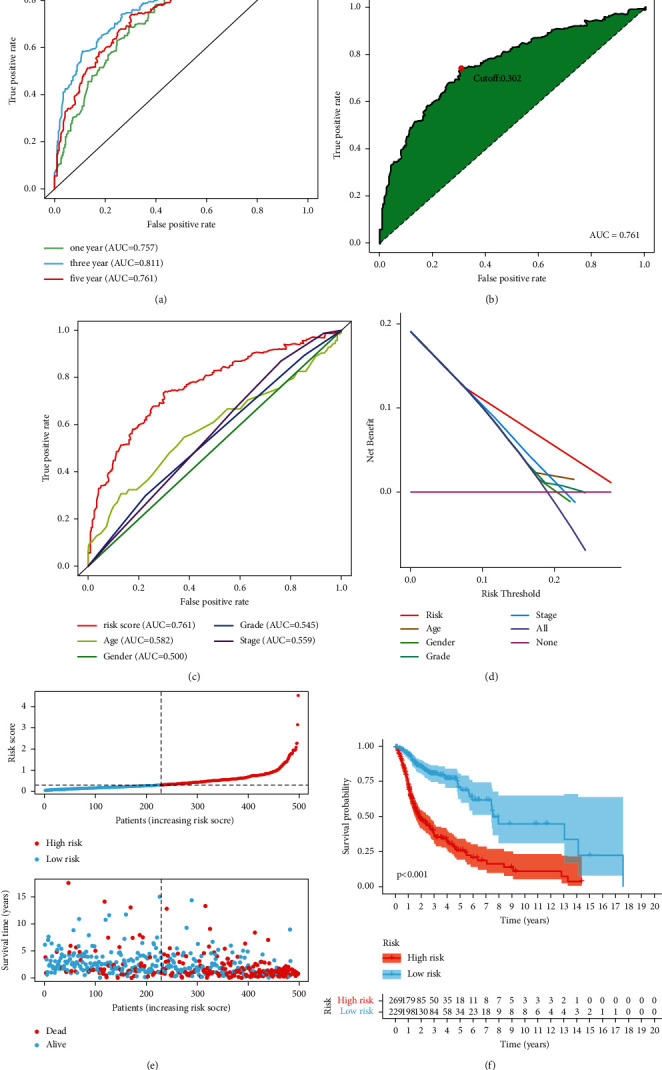
Evaluation of survival prediction for the risk signature. (a) The AUC values of one-, three-, and five-year ROC were more than 0.75. (b) The cut-off point was the maximum inflection point of the AIC. (c) ROC curve indicated the supremacy of the risk signature compared to other clinical features. (d) The DCA of the risk model. (e) Risk scores and survival status of an individual patient. (f) Kaplan–Meier analysis showed a longer survival time for the low-risk patients.

To evaluate the predictability of the model for survival, ROC curves, AUC, and DCA were assessed. The ROC curves indicated the high sensitiveness and specification of the 11-DEFRlncRNA-pair model for one-, three-, and five-year survival prediction, with all AUC values exceeding 0.75 (Figure 3(a)). Also, this signature has a larger AUC value compared to other established signatures (Figure S1). Subsequently, in light of the maximum inflection point (0.302) of AIC values on the five-year ROC curve, we categorized patients into high- or low-risk groups (Figure 3(b)). The AUC and DCA for five-year survival prediction demonstrated that the signature was more precise than other traditional clinicopathological features (Figures 3(c) and 3(d)).

### 3.3. Assessment of the Prognostic Risk Model

The profiles of risk score and survival status were displayed in the different groups of HNSCC patients, suggesting that the low-risk patients had better survival status than the high-risk patients (Figure 3(e)). With patients separated by the 11-DEFRlncRNA-pair signature, the low-risk patients showed substantially longer survival than the high-risk patients (Figure 3(f)).

**Figure 4:**
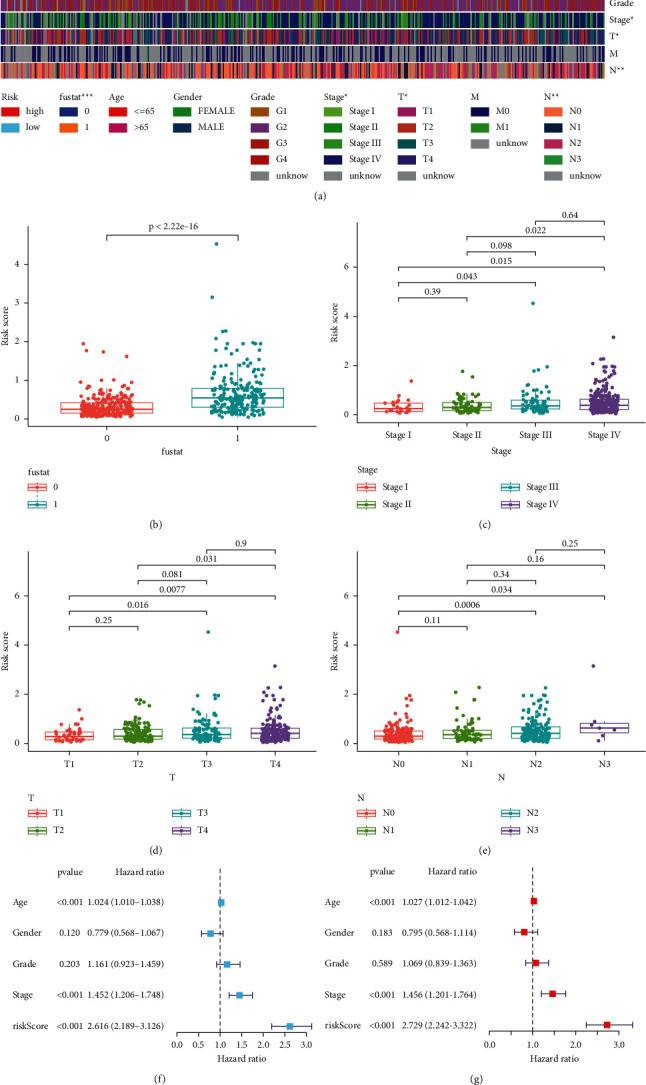
Assessment of clinical features. (a–e) The risk score was significantly associated with survival status, clinical stage, T stage, and N stage. (f–g) Univariate and multivariate Cox analysis showed that the differences in risk score, age, and clinical stage were statistically significant and were independently prognostic risk contributors.

**Figure 5:**
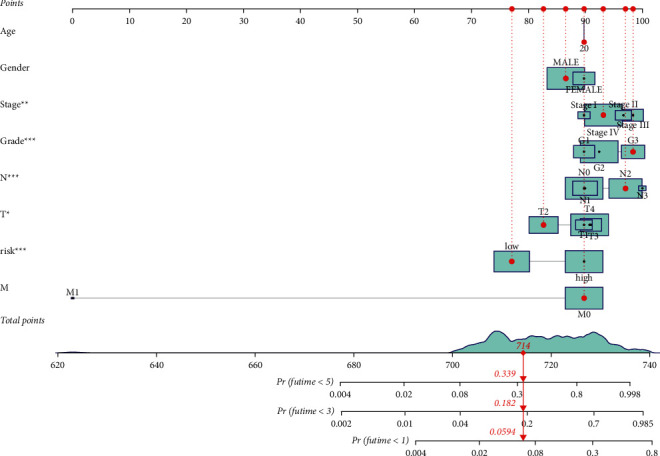
A nomogram based on clinical characteristics and risk groups.

The association of the risk score of the model with the clinicopathological features of the patients was performed by chi-squared tests. The strip chart and scatter diagrams acquired indicated that survival status, clinical, T, and N stages were significantly related to the risk score (Figures 4(a)–4(e)). Univariate Cox analysis demonstrated that clinical elements, such as risk score (*p* value <0.001), age (*p* value<0.001), and clinical stage (*p* value<0.001), were significantly related to prognosis (Figure 4(f)), and multivariate Cox analysis demonstrated that risk score (*p* value<0.001), age (*p* value <0.001), and clinical stage (*p* value <0.001) were also individual prognostic risk elements (Figure 4(g)). Detailed information is provided in Supplementary Table S5. The nomogram combining clinicopathological characteristics and the 11-DEFRlncRNA-pair signature was reliable and accurate and can be utilized in the survival prediction of HNSCC patients (Figure 5).

### 3.4. Exploration of Tumor-Infiltrating Immune Cells

**Figure 6:**
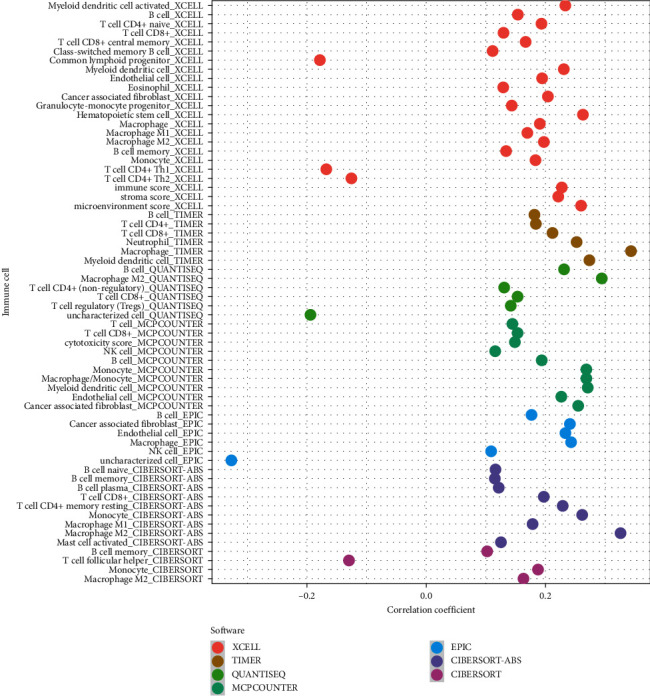
Exploration of tumor-infiltrating cells. The high-risk group was positively related to tumor-infiltrating immune cells such as B cells, CD4+ T cells, CD8+ T cells, NK cells, macrophages, monocytes, and myeloid dendritic cells.

A large number of clinical trials in cohorts have shown that immunotherapy has a significant role in the therapy of HNSCC, so we have further investigated the association of this model with the tumor immune microenvironment. The immune cell infiltration on the basis of TIMER, CIBERSORT, CIBERSORT-ABS, QUANTISEQ, MCPCOUNTER, XCELL, and EPIC algorithms is presented in Figure 6. The findings revealed that the high-risk group was positively related to tumor-infiltrating immune cells, including B cells, CD4+ T cells, CD8+ T cells, NK cells, macrophages, monocytes, and myeloid dendritic cells. The detailed Spearman correlation analysis is presented in Supplementary Table S6.

### 3.5. Association between the Risk Model and Other Biomarkers

**Figure 7:**
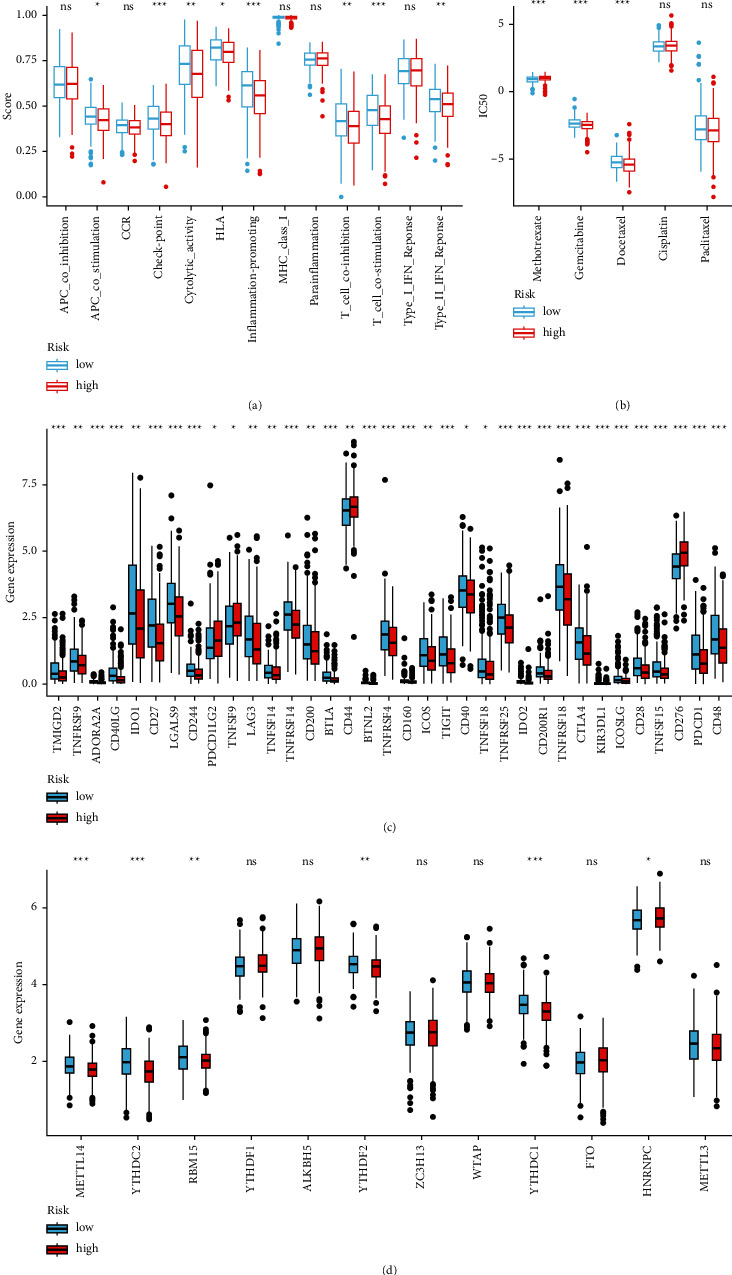
Analysis of immunity and chemotherapeutic sensitivity and expression of ICIs-related molecules and m6A-related genes. (a) Study for the correlation between subgroups of immune cells and related functions. (b–d) Differences in IC50 of chemotherapeutics, ICIs-related molecules' gene expression, and m6A-related gene expression in different groups.

Association between immune cell subsets and immune functions according to ssGSEA suggested that costimulation of APC, immune checkpoint response, cytolytic response, HLA, promotion of inflammation, costimulation of T cells, coinhibition of T cells, and type II INF response were observed as statistical differences among the different groups (Figure 7(a)). We discovered that the IC50 of methotrexate, gemcitabine, and docetaxel was statistical differences between the different groups; however, the difference in IC50 for cisplatin and paclitaxel was small (Figure 7(b)). We also explored if the model was associated with ICIs and revealed remarkable differences in the expression of CTLA-4 (*p* < 0.001), PDCD1 (*p* < 0.001), LAG3 (*p* < 0.01), TIGIT (*p* < 0.001), and BTLA (*p* < 0.001) among others, between the different groups (Figure 7(c)). The comparison of m^6^A-related mRNAs in different subgroups showed that the expression levels of METTL14 (*p* < 0.001), YTHDC2 (*p* < 0.001), RBM15 (*p* < 0.01), YTHDF2 (*p* < 0.01), YTHDC1 (*p* < 0.001), and HNRNPC (*p* < 0.05) were significant (Figure 7(d)).

## 4. Discussion

Ferroptosis is a unique pattern of cell death that has received extensive interest, especially in the area of tumorigenesis and therapies [29]. Several research studies have mainly concentrated on developing DEFRlncRNA signatures to assess the prognosis of tumor patients [16, 18, 30–32]. Nevertheless, the vast majority of signatures are established on the basis of the expression levels of quantitative transcripts. The study was motivated by the idea of establishing an immune-related lncRNA-pair signature and tried to establish a rational signature with two FRlncRNA combinations, which are not affected by their precise expression levels in the signature [33].

To begin with, we acquired the RNA-seq data in TCGA, conducted coexpression analysis and differentially expressed analysis to screen out DEFRlncRNAs, and obtained DEFRlncRNA pairs by pairing DEFRlncRNAs. Secondly, univariable Cox, LASSO, and multivariate Cox regression analysis were applied to identify these DEFRlncRNA pairs, and a prognostic model was established. Thirdly, we computed the AIC values for each point on the AUC to decide the optimum cut-off point to classify patients into different groups. Lastly, we assessed the novel model in various clinical settings, such as survival, clinicopathological characteristics, tumor-infiltrating immune cells, immune functions, chemotherapeutic sensitivity, ICIs-related molecules, and m^6^A-related genes.

Currently, the regulatory mechanisms of ferroptosis are not sufficiently clear, particularly in the field of lncRNAs. A study showed that P53RRA specifically interacted with the functional regions of signaling proteins in the cytoplasm and suppressed tumor development by regulating nuclear sequestration of p53 through ferroptosis [11]. A group recently reported that LINC00618 accelerated ferroptosis by inhibiting SLC7A11, a significant negative modulator of ferroptosis [34]. Wang et al. demonstrated that LINC00336 suppressed ferroptosis and facilitated tumor development in lung cancer by its interaction with ELAVL1 [10]. Zhang et al. indicated that OIP5-AS1 promotes ferroptosis resistance in prostate cancer by miR-128-3p/SLC7A11 signaling [35]. In this study, various FRlncRNAs in the modeling process that have been identified perform an essential function in the tumorigenesis and progression of HNSCC. A study showed that overexpression of C5orf66-AS1 can prevent oral squamous cell carcinoma through suppressing cell growth and metastasis by modulating CYC1 [36]. Li et al. revealed that HOTAIR can bind with miR-206, facilitating STC2 and activating the PI3K/AKT signal pathway, thereby regulating cell biological functions in HNSCC [37]. Cui et al. disclosed that MNX1-AS1 competitively bounds miR-370, regulating FoxM1 and thereby modulating laryngeal squamous cell carcinoma progression [38]. These findings might provide a valuable perspective for future research.

Immune regulation is critical in the development, establishment, advancement, and treatment of HNSCC. A study revealed that patients with low levels of CD8+ T cell infiltration received worse treatment responses to pembrolizumab [39]. To obtain the correlation between risk score and tumor-infiltrating immune cells, some widely accepted approaches were used to estimate immune infiltrating cells, including TIMER, CIBERSORT, CIBERSORT-ABS, QUANTISEQ, MCPCOUNTER, XCELL, and EPIC [40–45]. By integrating and analyzing these results, the results revealed that the high-risk group was positively related to tumor-infiltrating immune cells such as B cells, CD4+ T cells, CD8+ T cells, NK cells, macrophages, monocytes, and myeloid dendritic cells. Recent research revealed that tumor cells experiencing ferroptosis could enhance antitumor immunity and their efficacy can be collaboratively contributed by ICIs, including in ICI-resistant tumors [46, 47]. A novel study has shown that macrophages can effectively engulf ferroptotic cancer cells in vitro [48]. An increasing number of evidence shows that ferroptotic cancer cells may have inherent immunogenicity, similar to necroptotic cancer cells [49, 50].

In the study, we developed for the first time a prognostic signature based on FRlncRNA pairs in HNSCC by structuring a 0 or 1 matrix. Moreover, the signature showed superior diagnostic accuracy and was predictive of patient prognosis, which has clinical application in HNCSS. The model can guide clinicians to choose appropriate chemotherapeutic agents for the treatment of HNSCC by comparing the sensitivity of the different groups to commonly administered drugs. The expression levels of ICIs-related molecules and m^6^A-related genes were statistically different in the different groups, which could provide a therapeutic theory for different immune and targeted therapies for HNSCC patients.

Ferroptosis is a novel mode of programmed cell death that may offer a novel way of antitumor treatment. Nonetheless, the current study has many shortcomings and limitations. Firstly, our sample size is relatively small and the normal to tumor sample counts are not proportional. Secondly, the results may be biased as the majority of samples from TCGA are nonmetastatic. Thirdly, our signature needs further validation using external validation to be more convincing.

In general, our research identified a new lncRNA signature that was unaffected by expression level, which might be used to predict patient prognosis and has potential clinical uses in HNCSS. The putative regulatory mechanisms by which lncRNA regulates ferroptosis and how it impacts the therapeutic efficacy of ferroptosis inducers are likely to be explored in future investigations. We expect that the practicability of the model can be verified in further clinical studies.

## Figures and Tables

**Table 1:** The clinical features of HNSCC patients.

Variables	Type	Total
Age	<=65/>65	323/174
Gender	Female/male	133/364
Grade	G1/G2/G3/G4/unknown	61/297/118/2/19
Stage	I/II/III/IV/unknown	25/69/78/258/67
T	T1/T2/T3/T4/unknown	45/131/96/170/55
M	M0/M1/unknown	183/1/313
N	N0/N1/N2/N3/unknown	168/65/164/7/93

## Data Availability

The datasets generated and analyzed in the current study can be obtained in the TCGA repository at https://tcga-data.nci.nih.gov/tcga.

## Supplementary Materials

Supplementary Materials Supplementary Figure 1: a comparison of one-, three-, and five-year ROC curves with other established models indicates the superiority of this risk model. Supplementary Table 1: the ferroptosis-related genes. Supplementary Table 2: 722 FRlncRNAs by coexpression analysis. Supplementary Table 3: 196 DEFRlncRNA by differential expression analysis by the fresh pairing algorithm. Supplementary Table 4: 13,444 DEFRlncRNA pairs by differential expression analysis. Supplementary Table 5: 2,753 DEFRlncRNA pairs via univariate Cox regression analysis. Supplementary Table 6: 11 DEFRlncRNA pairs through multivariate Cox regression analysis. Supplementary Table 7: the details of univariate and multivariate Cox regression analysis. Supplementary Table 8: the detailed comparison of the relationship between tumour-infiltrating immune cells and risk sores.

## References

[1] Sung H., Ferlay J., Siegel R. L. (2021). Global cancer statistics 2020: GLOBOCAN estimates of incidence and mortality worldwide for 36 cancers in 185 countries. *CA: A Cancer Journal for Clinicians*.

[2] Leemans C. R., Snijders P. J. F., Brakenhoff R. H. (2018). The molecular landscape of head and neck cancer. *Nature Reviews Cancer*.

[3] Marur S., Forastiere A. A. (2016). Head and neck squamous cell carcinoma: update on epidemiology, diagnosis, and treatment. *Mayo Clinic Proceedings*.

[4] Friedmann Angeli J. P., Krysko D. V., Conrad M. (2019). Ferroptosis at the crossroads of cancer-acquired drug resistance and immune evasion. *Nature Reviews Cancer*.

[5] Hassannia B., Vandenabeele P., Vanden Berghe T. (2019). Targeting ferroptosis to iron out cancer. *Cancer Cell*.

[6] Lu D., Yang Z., Xia Q. (2020). ACADSB regulates ferroptosis and affects the migration, invasion, and proliferation of colorectal cancer cells. *Cell Biology International*.

[7] Mou Y., Wang J., Wu J. (2019). Ferroptosis, a new form of cell death: opportunities and challenges in cancer. *Journal of Hematology & Oncology*.

[8] Chekerov R., Hilpert F., Mahner S. (2018). Sorafenib plus topotecan versus placebo plus topotecan for platinum-resistant ovarian cancer (TRIAS): a multicentre, randomised, double-blind, placebo-controlled, phase 2 trial. *The Lancet Oncology*.

[9] Li C. H., Chen Y. (2016). Insight into the role of long noncoding RNA in cancer development and progression. *International Review of Cell and Molecular Biology*.

[10] Wang M., Mao C., Ouyang L. (2019). Long noncoding RNA LINC00336 inhibits ferroptosis in lung cancer by functioning as a competing endogenous RNA. *Cell Death & Differentiation*.

[11] Mao C., Wang X., Liu Y. (2018). A G3BP1-interacting lncRNA promotes ferroptosis and apoptosis in cancer via nuclear sequestration of p53. *Cancer Research*.

[12] Qi W., Li Z., Xia L. (2019). LncRNA GABPB1-AS1 and GABPB1 regulate oxidative stress during erastin-induced ferroptosis in HepG2 hepatocellular carcinoma cells. *Scientific Reports*.

[13] Luo W., Wang J., Xu W. (2021). LncRNA RP11-89 facilitates tumorigenesis and ferroptosis resistance through PROM2-activated iron export by sponging miR-129-5p in bladder cancer. *Cell Death & Disease*.

[14] Chen Q., Wang W., Wu Z. (2021). Over-expression of lncRNA TMEM161B-AS1 promotes the malignant biological behavior of glioma cells and the resistance to temozolomide via up-regulating the expression of multiple ferroptosis-related genes by sponging HSA-miR-27a-3p. *Cell Death Discovery*.

[15] Liang J., Zhi Y., Deng W. (2021). Development and validation of ferroptosis-related lncRNAs signature for hepatocellular carcinoma. *PeerJ*.

[16] Tang Y., Li C., Zhang Y. J., Wu Z. H. (2021). Ferroptosis-related long non-coding RNA signature predicts the prognosis of head and neck squamous cell carcinoma. *International Journal of Biological Sciences*.

[17] Zhang K., Ping L., Du T. (2021). A ferroptosis-related lncRNAs signature predicts prognosis and immune microenvironment for breast cancer. *Frontiers in Molecular Biosciences*.

[18] Cai H., Zhuang Z., Wu Y. (2021). Development and validation of a ferroptosis-related lncRNAs prognosis signature in colon cancer. *Bosnian Journal of Basic Medical Sciences*.

[19] Tang R., Wu Z., Rong Z. (2022). Ferroptosis-related lncRNA pairs to predict the clinical outcome and molecular characteristics of pancreatic ductal adenocarcinoma. *Briefings in Bioinformatics*.

[20] Lv Y., Lin S. Y., Hu F. F. (2019). Landscape of cancer diagnostic biomarkers from specifically expressed genes. *Briefings in Bioinformatics*.

[21] Yin J., Li X., Lv C. (2021). Immune-related lncRNA signature for predicting the immune landscape of head and neck squamous cell carcinoma. *Frontiers in Molecular Biosciences*.

[22] Yates A. D., Allen J., Amode R. M. (2022). Ensembl genomes 2022: an expanding genome resource for non-vertebrates. *Nucleic Acids Research*.

[23] Zhou N., Bao J. (2020). FerrDb: a manually curated resource for regulators and markers of ferroptosis and ferroptosis-disease associations. *Database*.

[24] Lu W., Wu Y., Huang S., Zhang D. (2021). A ferroptosis-related gene signature for predicting the prognosis and drug sensitivity of head and neck squamous cell carcinoma. *Frontiers in Genetics*.

[25] Han F., Li W., Chen T. (2021). Ferroptosis-related genes for predicting prognosis of patients with laryngeal squamous cell carcinoma. *European Archives of Oto-Rhino-Laryngology*.

[26] Guo Q., Zhang X., Shen T., Wang X. (2021). Identification of autophagy- and ferroptosis-related lncRNAs functioned through immune-related pathways in head and neck squamous carcinoma. *Life*.

[27] He D., Liao S., Xiao L. (2021). Prognostic value of a ferroptosis-related gene signature in patients with head and neck squamous cell carcinoma. *Frontiers in Cell and Developmental Biology*.

[28] Fan X., Ou Y., Liu H. (2021). A ferroptosis-related prognostic signature based on antitumor immunity and tumor protein p53 mutation exploration for guiding treatment in patients with head and neck squamous cell carcinoma. *Frontiers in Genetics*.

[29] Liang C., Zhang X., Yang M., Dong X. (2019). Recent progress in ferroptosis inducers for cancer therapy. *Advances in Materials*.

[30] Dong Y., Liu D., Zhou H., Gao Y., Nueraihemaiti Y., Xu Y. (2022). A prognostic signature for clear cell renal cell carcinoma based on ferroptosis-related lncRNAs and immune checkpoints. *Frontiers in Genetics*.

[31] Lin X., Yang S. (2022). A prognostic signature based on the expression profile of the ferroptosis-related long non-coding RNAs in hepatocellular carcinoma. *Advances in Clinical and Experimental Medicine: Official Organ Wroclaw Medical University*.

[32] Yang X., Mei M., Yang J., Guo J., Du F., Liu S. (2022). Ferroptosis-related long non-coding RNA signature predicts the prognosis of hepatocellular carcinoma. *Aging*.

[33] Hong W. F., Liang L., Gu Y. J. (2020). Immune-related lncRNA to construct novel signature and predict the immune landscape of human hepatocellular carcinoma. *Molecular Therapy - Nucleic Acids*.

[34] Wang Z., Chen X., Liu N. (2021). A nuclear long non-coding RNA LINC00618 accelerates ferroptosis in a manner dependent upon apoptosis. *Molecular Therapy*.

[35] Zhang Y., Guo S., Wang S. (2021). LncRNA OIP5-AS1 inhibits ferroptosis in prostate cancer with long-term cadmium exposure through miR-128-3p/SLC7A11 signaling. *Ecotoxicology and Environmental Safety*.

[36] Lu T., Liu H., You G. (2018). Long non-coding RNA C5orf66-AS1 prevents oral squamous cell carcinoma through inhibiting cell growth and metastasis. *International Journal of Molecular Medicine*.

[37] Li T., Qin Y., Zhen Z. (2019). Long non coding RNA HOTAIR/microRNA 206 sponge regulates STC2 and further influences cell biological functions in head and neck squamous cell carcinoma. *Cell Proliferation*.

[38] Cui X., Yu H., Yu T., Xiao D., Wang X. (2021). LncRNA MNX1-AS1 drives aggressive laryngeal squamous cell carcinoma progression and serves as a ceRNA to target FoxM1 by sponging microRNA-370. *Aging*.

[39] Garon E. B., Hellmann M. D., Rizvi N. A. (2019). Five-year overall survival for patients with advanced non‒small-cell lung cancer treated with pembrolizumab: results from the phase I KEYNOTE-001 study. *Journal of Clinical Oncology*.

[40] Aran D., Hu Z., Butte A. J. (2017). xCell: digitally portraying the tissue cellular heterogeneity landscape. *Genome Biology*.

[41] Chen B., Khodadoust M. S., Liu C. L., Newman A. M., Alizadeh A. A. (2018). Profiling tumor infiltrating immune cells with CIBERSORT. *Methods in Molecular Biology*.

[42] Li M. Q., Liang M. N., Lan T. (2020). Four immune-related long non-coding RNAs for prognosis prediction in patients with hepatocellular carcinoma. *Frontiers in Molecular Biosciences*.

[43] Plattner C., Finotello F., Rieder D. (2020). Deconvoluting tumor-infiltrating immune cells from RNA-seq data using quanTIseq. *Methods in Enzymology*.

[44] Racle J., de Jonge K., Baumgaertner P., Speiser D. E., Gfeller D. (2017). Simultaneous enumeration of cancer and immune cell types from bulk tumor gene expression data. *eLife*.

[45] Becht E., Giraldo N. A., Lacroix L. (2016). Estimating the population abundance of tissue-infiltrating immune and stromal cell populations using gene expression. *Genome Biology*.

[46] Antonia S., Goldberg S. B., Balmanoukian A. (2016). Safety and antitumour activity of durvalumab plus tremelimumab in non-small cell lung cancer: a multicentre, phase 1b study. *The Lancet Oncology*.

[47] Socinski M. A., Jotte R. M., Cappuzzo F. (2018). Atezolizumab for first-line treatment of metastatic nonsquamous NSCLC. *New England Journal of Medicine*.

[48] Klöditz K., Fadeel B. (2019). Three cell deaths and a funeral: macrophage clearance of cells undergoing distinct modes of cell death. *Cell Death Discovery*.

[49] Aaes T. L., Kaczmarek A., Delvaeye T. (2016). Vaccination with necroptotic cancer cells induces efficient anti-tumor immunity. *Cell Reports*.

[50] Yatim N., Jusforgues-Saklani H., Orozco S. (2015). RIPK1 and NF-*κ*B signaling in dying cells determines cross-priming of CD8+ T cells. *Science*.

