# Supplementary material for: Identification of Ferroptosis-Related lncRNA Pairs for Predicting the Prognosis of Head and Neck Squamous Cell Carcinoma
Source: J Oncol. 2022 Jul 20;2022:7602482. doi: 10.1155/2022/7602482 (PMC9328971; doi:10.1155/2022/7602482)
Supplement: Supplementary Materials — Supplementary Figure 1: a comparison of one-, three-, and five-year ROC curves with other established models indicates the superiority of this risk model. Supplementary Table 1: the ferroptosis-related genes. Supplementary Table 2: 722 FRlncRNAs by coexpression analysis. Supplementary Table 3: 196 DEFRlncRNA by differential expression analysis by the fresh pairing algorithm. Supplementary Table 4: 13,444 DEFRlncRNA pairs by differential expression analysis. Supplementary Table 5: 2,753 DEFRlncRNA pairs via univariate Cox regression analysis. Supplementary Table 6: 11 DEFRlncRNA pairs through multivariate Cox regression analysis. Supplementary Table 7: the details of univariate and multivariate Cox regression analysis. Supplementary Table 8: the detailed comparison of the relationship between tumour-infiltrating immune cells and risk sores. [file 7602482.f1.zip › Supplementary Table All (1).docx]

**Supplementary Table 1:** The ferroptosis-related genes.

| **Driver** |  |
| --- | --- |
| Symbol | Name |
| RPL8 | Ribosomal protein L8 |
| IREB2 | Iron response element binding protein 2 |
| ATP5MC3 | ATP synthase membrane subunit c locus 3 |
| CS | Citrate synthase |
| EMC2 | ER membrane protein complex subunit 2 |
| ACSF2 | Acyl-CoA synthetase family member 2 |
| NOX1 | Nicotinamide adenine dinucleotide phosphate (NADPH) oxidase (NOX) 1 |
| CYBB | Cytochrome b-245 beta chain |
| NOX3 | Nicotinamide adenine dinucleotide phosphate (NADPH) oxidase (NOX) 3 |
| NOX4 | Nicotinamide adenine dinucleotide phosphate (NADPH) oxidase (NOX) 4 |
| NOX5 | Nicotinamide adenine dinucleotide phosphate (NADPH) oxidase (NOX) 5 |
| DUOX1 | Dual oxidase 1 |
| DUOX2 | Dual oxidase 2 |
| G6PD | Glucose-6-phosphate dehydrogenase |
| PGD | Phosphoglycerate dehydrogenase |
| VDAC2 | Valtage-dependent anion channels 2 |
| PIK3CA | Phosphatidylinositol-4,5-bisphosphate 3-kinase catalytic subunit alpha |
| FLT3 | Fms related tyrosine kinase 3 |
| SCP2 | Sterol carrier protein 2 |
| TP53 | Tumor protein p53 |
| ACSL4 | Acyl-CoA synthetase long chain family member 4 |
| LPCAT3 | Lysophosphatidylcholine acyltransferase 3 |
| NRAS | NRAS proto-oncogene, GTPase |
| KRAS | KRAS proto-oncogene, GTPase |
| HRAS | HRas proto-oncogene, GTPase |
| TF | Transferrin |
| TFRC | Transferrin receptor |
| TFR2 | Transferrin receptor 2 |
| SLC38A1 | Solute carrier family 38 member 1 |
| SLC1A5 | Solute carrier family 1 member 5 |
| GLS2 | Glutaminase 2 |
| GOT1 | Glutamic-oxaloacetic transaminase 1 |
| CARS1 | Cysteinyl-tRNA synthetase 1 |
| TP53 | Tumor protein p53 |
| ALOX5 | Arachidonate 5-lipoxygenase |
| KEAP1 | Kelch like ECH associated protein 1 |
| HMOX1 | Heme oxygenase 1 |
| TP53 | Tumor protein p53 |
| TP53 | Tumor protein p53 |
| GLS2 | Glutaminase 2 |
| ATG5 | Autophagy related 5 |
| ATG7 | Autophagy related 7 |
| NCOA4 | Nuclear receptor coactivator 4 |
| TF | Transferrin |
| ALOX5 | Arachidonate 5-lipoxygenase |
| ALOX12 | Arachidonate 12-lipoxygenase, 12S type |
| ALOX12B | Arachidonate 12-lipoxygenase, 12R type |
| ALOX15 | Arachidonate 15-lipoxygenase |
| ALOX15B | Arachidonate 15-lipoxygenase type B |
| ALOXE3 | Arachidonate lipoxygenase 3 |
| PHKG2 | Phosphorylase kinase catalytic subunit gamma 2 |
| TFRC | Transferrin receptor |
| ACO1 | Aconitase 1 |
| IREB2 | iron responsive element binding protein 2 |
| SLC38A1 | Solute carrier family 38 member 1 |
| GLS2 | Glutaminase 2 |
| G6PDX | _NA_ |
| ULK1 | Unc-51 like autophagy activating kinase 1 |
| ATG3 | Autophagy related 3 |
| ATG4D | Autophagy related 4D cysteine peptidase |
| ATG5 | Autophagy related 5 |
| BECN1 | Beclin 1 |
| MAP1LC3A | Microtubule associated protein 1 light chain 3 alpha |
| GABARAPL2 | GABA type A receptor associated protein like 2 |
| GABARAPL1 | GABA type A receptor associated protein like 1 |
| ATG16L1 | Autophagy related 16 like 1 |
| WIPI1 | WD repeat domain, phosphoinositide interacting 1 |
| WIPI2 | WD repeat domain, phosphoinositide interacting 2 |
| SNX4 | Sorting nexin 4 |
| ATG13 | Autophagy related 13 |
| ULK2 | Unc-51 like autophagy activating kinase 2 |
| NCOA4 | Nuclear receptor coactivator 4 |
| ACSL4 | Acyl-CoA synthetase long chain family member 4 |
| TP53 | Tumor protein p53 |
| SAT1 | Spermidine/spermine N1-acetyltransferase 1 |
| ALOX15 | Arachidonate 15-lipoxygenase |
| ACSL4 | Acyl-CoA synthetase long chain family member 4 |
| LPCAT3 | Lysophosphatidylcholine acyltransferase 3 |
| ALOX15 | Arachidonate 15-lipoxygenase |
| ACSL4 | Acyl-CoA synthetase long chain family member 4 |
| KEAP1 | Kelch like ECH associated protein 1 |
| EGFR | Epidermal growth factor receptor |
| NOX4 | NADPH oxidase 4 |
| MAPK3 | Mitogen-activated protein kinase 3 |
| MAPK1 | Mitogen-activated protein kinase 1 |
| BID | BH3 interacting domain death agonist |
| ACSL4 | Acyl-CoA synthetase long chain family member 4 |
| ZEB1 | Zinc finger E-box binding homeobox 1 |
| KEAP1 | Kelch like ECH associated protein 1 |
| DPP4 | Dipeptidyl peptidase 4 |
| ALOX15 | Arachidonate 15-lipoxygenase |
| ALOX12 | Arachidonate 12-lipoxygenase, 12S type |
| CDKN2A | Cyclin dependent kinase inhibitor 2A |
| PEBP1 | Phosphatidylethanolamine binding protein 1 |
| SOCS1 | Suppressor of cytokine signaling 1 |
| CDO1 | Cysteine dioxygenase type 1 |
| MYB | MYB proto-oncogene, transcription factor |
| HMOX1 | Heme oxygenase 1 |
| MAPK8 | Mitogen-activated protein kinase 8 |
| MAPK9 | Mitogen-activated protein kinase 9 |
| MAPK1 | Mitogen-activated protein kinase 1 |
| MAPK3 | Mitogen-activated protein kinase 3 |
| SLC1A5 | Solute carrier family 1 member 5 |
| CHAC1 | ChaC glutathione specific gamma-glutamylcyclotransferase 1 |
| MAPK14 | Mitogen-activated protein kinase 14 |
| LINC00472 | Long intergenic non-protein coding RNA 472 |
| NOX4 | NADPH oxidase 4 |
| GOT1 | Glutamic-oxaloacetic transaminase 1 |
| BECN1 | Beclin 1 |
| PRKAA2 | Protein kinase AMP-activated catalytic subunit alpha 2 |
| PRKAA1 | Protein kinase AMP-activated catalytic subunit alpha 1 |
| ELAVL1 | ELAV like RNA binding protein 1 |
| BAP1 | BRCA1 associated protein 1 |
| TP53 | Tumor protein p53 |
| ABCC1 | ATP binding cassette subfamily C member 1 |
| ACSL4 | Acyl-CoA synthetase long chain family member 4 |
| MIR6852 | microRNA 6852 |
| ACVR1B | Activin A receptor type 1B |
| TGFBR1 | Transforming growth factor beta receptor 1 |
| BAP1 | BRCA1 associated protein 1 |
| EPAS1 | Endothelial PAS domain protein 1 |
| HILPDA | Hypoxia inducible lipid droplet associated |
| HIF1A | Hypoxia inducible factor 1 subunit alpha |
| ALOX12 | Arachidonate 12-lipoxygenase, 12S type |
| ACSL4 | Acyl-CoA synthetase long chain family member 4 |
| HMOX1 | Heme oxygenase 1 |
| IFNG | Interferon gamma |
| ANO6 | Anoctamin 6 |
| LPIN1 | Lipin 1 |
| HMGB1 | High mobility group box 1 |
| TNFAIP3 | TNF alpha induced protein 3 |
| TLR4 | Toll like receptor 4 |
| NOX4 | NADPH oxidase 4 |
| ATF3 | Activating transcription factor 3 |
| ATM | ATM serine/threonine kinase |
| YY1AP1 | YY1 associated protein 1 |
| EGLN2 | Egl-9 family hypoxia inducible factor 2 |
| MIOX | Myo-inositol oxygenase |
| TAZ | Tafazzin |
| MTDH | Metadherin |
| IDH1 | Isocitrate dehydrogenase (NADP(+)) 1 |
| SIRT1 | Sirtuin 1 |
| TAZ | Tafazzin |
| BECN1 | Beclin 1 |
| FBXW7 | F-box and WD repeat domain containing 7 |
| PANX1 | Pannexin 1 |
| DNAJB6 | DnaJ heat shock protein family (Hsp40) member B6 |
| BACH1 | BTB domain and CNC homolog 1 |
| ACSL4 | Acyl-CoA synthetase long chain family member 4 |
| LONP1 | Lon peptidase 1, mitochondrial |
| **Suppressor** |  |
| SLC7A11 | Solute carrier family 7 member 11 |
| GPX4 | Glutathione peroxidase 4 |
| AKR1C1 | Aldo-keto reductase family 1 member C1 |
| AKR1C2 | Aldo-keto reductase family 1 member C2 |
| AKR1C3 | Aldo-keto reductase family 1 member C3 |
| GPX4 | Glutathione peroxidase 4 |
| RB1 | RB transcriptional corepressor 1 |
| HSPB1 | Heat shock protein family B (small) member 1 |
| HSF1 | Heat shock transcription factor 1 |
| SLC7A11 | Solute carrier family 7 member 11 |
| GPX4 | Glutathione peroxidase 4 |
| GCLC | Glutamate-cysteine ligase catalytic subunit |
| SLC7A11 | Solute carrier family 7 member 11 |
| NFE2L2 | Nuclear factor, erythroid 2 like 2 |
| SQSTM1 | Sequestosome 1 |
| NQO1 | NAD(P)H quinone dehydrogenase 1 |
| HMOX1 | Heme oxygenase 1 |
| FTH1 | Ferritin heavy chain 1 |
| MUC1 | Mucin 1, cell surface associated |
| SLC3A2 | Solute carrier family 3 member 2 |
| MT1G | Metallothionein 1G |
| NFE2L2 | Nuclear factor, erythroid 2 like 2 |
| SLC40A1 | Solute carrier family 40 member 1 |
| SLC7A11 | Solute carrier family 7 member 11 |
| GPX4 | Glutathione peroxidase 4 |
| SLC7A11 | Solute carrier family 7 member 11 |
| CISD1 | CDGSH iron sulfur domain 1 |
| SLC7A11 | Solute carrier family 7 member 11 |
| FANCD2 | FA complementation group D2 |
| GPX4 | Glutathione peroxidase 4 |
| NFE2L2 | Nuclear factor, erythroid 2 like 2 |
| FTMT | Ferritin mitochondrial |
| HSPA5 | Heat shock protein family A (Hsp70) member 5 |
| ATF4 | Activating transcription factor 4 |
| SLC7A11 | Solute carrier family 7 member 11 |
| GPX4 | Glutathione peroxidase 4 |
| GPX4 | Glutathione peroxidase 4 |
| HMOX1 | Heme oxygenase 1 |
| ATF4 | Activating transcription factor 4 |
| NFE2L2 | Nuclear factor, erythroid 2 like 2 |
| TP53 | Tumor protein p53 |
| SLC7A11 | Solute carrier family 7 member 11 |
| HELLS | Helicase, lymphoid specific |
| SCD | Stearoyl-CoA desaturase |
| FADS2 | Fatty acid desaturase 2 |
| SRC | SRC proto-oncogene, non-receptor tyrosine kinase |
| STAT3 | Signal transducer and activator of transcription 3 |
| NFE2L2 | Nuclear factor, erythroid 2 like 2 |
| PML | Promyelocytic leukemia |
| MTOR | Mechanistic target of rapamycin kinase |
| NFS1 | NFS1 cysteine desulfurase |
| TP63 | Tumor protein p63 |
| SLC7A11 | Solute carrier family 7 member 11 |
| TP53 | Tumor protein p53 |
| CDKN1A | Cyclin dependent kinase inhibitor 1A |
| MIR137 | microRNA 137 |
| SLC40A1 | Solute carrier family 40 member 1 |
| GPX4 | Glutathione peroxidase 4 |
| GPX4 | Glutathione peroxidase 4 |
| ENPP2 | Ectonucleotide pyrophosphatase/phosphodiesterase 2 |
| VDAC2 | Voltage dependent anion channel 2 |
| FH | Fumarate hydratase |
| CISD2 | CDGSH iron sulfur domain 2 |
| SLC40A1 | Solute carrier family 40 member 1 |
| MIR9-1 | microRNA 9-1 |
| MIR9-2 | microRNA 9-2 |
| MIR9-3 | microRNA 9-3 |
| CBS | Cystathionine beta-synthase |
| NFE2L2 | Nuclear factor, erythroid 2 like 2 |
| SQSTM1 | Sequestosome 1 |
| GPX4 | Glutathione peroxidase 4 |
| ISCU | Iron-sulfur cluster assembly enzyme |
| FTH1 | Ferritin heavy chain 1 |
| ACSL3 | Acyl-CoA synthetase long chain family member 3 |
| OTUB1 | OTU deubiquitinase, ubiquitin aldehyde binding 1 |
| CD44 | CD44 molecule (Indian blood group) |
| LINC00336 | Long intergenic non-protein coding RNA 336 |
| STAT3 | Signal transducer and activator of transcription 3 |
| BRD4 | Bromodomain containing 4 |
| PRDX6 | Peroxiredoxin 6 |
| MIR17 | microRNA 17 |
| SCD | Stearoyl-CoA desaturase |
| SESN2 | Sestrin 2 |
| NF2 | Neurofibromin 2 |
| ARNTL | Aryl hydrocarbon receptor nuclear translocator like |
| HIF1A | Hypoxia inducible factor 1 subunit alpha |
| JUN | Jun proto-oncogene, AP-1 transcription factor subunit |
| CA9 | Carbonic anhydrase 9 |
| HSPA5 | Heat shock protein family A (Hsp70) member 5 |
| TMBIM4 | Transmembrane BAX inhibitor motif containing 4 |
| HSPA5 | Heat shock protein family A (Hsp70) member 5 |
| PLIN2 | Perilipin 2 |
| MIR212 | microRNA 212 |
| Fer1HCH | Ferritin 1 Heavy Chain Homolog |
| AIFM2 | Apoptosis inducing factor mitochondria associated 2 |
| AIFM2 | Apoptosis inducing factor mitochondria associated 2 |
| LAMP2 | Lysosomal associated membrane protein 2 |
| ZFP36 | ZFP36 ring finger protein |
| GPX4 | Glutathione peroxidase 4 |
| PROM2 | Prominin 2 |
| CHMP5 | Charged multivesicular body protein 5 |
| CHMP6 | Charged multivesicular body protein 6 |
| AKR1C1 | Aldo-keto reductase family 1 member C1 |
| AKR1C2 | Aldo-keto reductase family 1 member C2 |
| AKR1C3 | Aldo-keto reductase family 1 member C3 |
| CBS | Cystathionine beta-synthase |
| NFE2L2 | Nuclear factor, erythroid 2 like 2 |
| CAV1 | Caveolin 1 |
| GCH1 | GTP cyclohydrolase 1 |
| **Marker** |  |
| PTGS2 | Prostaglandin-endoperoxide synthase 2 |
| DUSP1 | Dual specificity phosphatase 1 |
| NOS2 | Nitric oxide synthase 2 |
| NCF2 | Neutrophil cytosolic factor 2 |
| MT3 | Metallothionein 3 |
| UBC | Ubiquitin C |
| ALB | Albumin |
| TXNRD1 | Thioredoxin reductase 1 |
| SRXN1 | Sulfiredoxin 1 |
| GPX2 | Glutathione peroxidase 2 |
| BNIP3 | BCL2 interacting protein 3 |
| OXSR1 | Oxidative stress responsive kinase 1 |
| SELENOS | Selenoprotein S |
| ANGPTL7 | Angiopoietin like 7 |
| CHAC1 | ChaC glutathione specific gamma-glutamylcyclotransferase 1 |
| SLC7A11 | Solute carrier family 7 member 11 |
| DDIT4 | DNA damage inducible transcript 4 |
| LOC284561 | _NA_ |
| ASNS | Asparagine synthetase (glutamine-hydrolyzing) |
| TSC22D3 | TSC22 domain family member 3 |
| DDIT3 | DNA damage inducible transcript 3 |
| JDP2 | Jun dimerization protein 2 |
| SESN2 | Sestrin 2 |
| SLC1A4 | Solute carrier family 1 member 4 |
| PCK2 | Phosphoenolpyruvate carboxykinase 2, mitochondrial |
| TXNIP | Thioredoxin interacting protein |
| VLDLR | Very low density lipoprotein receptor |
| GPT2 | Glutamic--pyruvic transaminase 2 |
| PSAT1 | Phosphoserine aminotransferase 1 |
| LURAP1L | Leucine rich adaptor protein 1 like |
| SLC7A5 | Solute carrier family 7 member 5 |
| HERPUD1 | Homocysteine inducible ER protein with ubiquitin like domain 1 |
| XBP1 | X-box binding protein 1 |
| ATF3 | Activating transcription factor 3 |
| SLC3A2 | Solute carrier family 3 member 2 |
| CBS | Cystathionine beta-synthase |
| ATF4 | Activating transcription factor 4 |
| ZNF419 | Zinc finger protein 419 |
| KLHL24 | Kelch like family member 24 |
| TRIB3 | Tribbles pseudokinase 3 |
| ZFP69B | ZFP69 zinc finger protein B |
| ATP6V1G2 | ATPase H+ transporting V1 subunit G2 |
| VEGFA | Vascular endothelial growth factor A |
| GDF15 | Growth differentiation factor 15 |
| TUBE1 | Tubulin epsilon 1 |
| ARRDC3 | Arrestin domain containing 3 |
| CEBPG | CCAAT enhancer binding protein gamma |
| SNORA16A | Small nucleolar RNA, H/ACA box 16A |
| RGS4 | Regulator of G protein signaling 4 |
| BLOC1S5-TXNDC5 | BLOC1S5-TXNDC5 readthrough (NMD candidate) |
| LOC390705 | _NA_ |
| EIF2S1 | Eukaryotic translation initiation factor 2 subunit 1 |
| KIM-1 | Kidney injury molecule-1 |
| IL6 | Interleukin 6 |
| CXCL2 | C-X-C motif chemokine ligand 2 |
| RELA | RELA proto-oncogene, NF-kB subunit |
| HSD17B11 | Hydroxysteroid 17-beta dehydrogenase 11 |
| AGPAT3 | 1-acylglycerol-3-phosphate O-acyltransferase 3 |
| SETD1B | SET domain containing 1B, histone lysine methyltransferase |
| HMOX1 | Heme oxygenase 1 |
| TF | Transferrin |
| FTL | Ferritin light chain |
| RPL8 | Ribosomal protein L8 |
| ATP5MC3 | ATP synthase membrane subunit c locus 3 |
| TFRC | Transferrin receptor |
| MAFG | MAF bZIP transcription factor G |
| IL33 | Interleukin 33 |
| FTH1 | Ferritin heavy chain 1 |
| SLC40A1 | Solute carrier family 40 member 1 |
| TF | Transferrin |
| TFRC | Transferrin receptor |
| FTH1 | Ferritin heavy chain 1 |
| GPX4 | Glutathione peroxidase 4 |
| HAMP | Hepcidin antimicrobial peptide |
| HSPB1 | Heat shock protein family B (small) member 1 |
| NFE2L2 | Nuclear factor, erythroid 2 like 2 |
| STEAP3 | STEAP3 metalloreductase |
| DRD5 | Dopamine receptor D5 |
| GPX4 | Glutathione peroxidase 4 |
| DRD4 | Dopamine receptor D4 |
| MAP3K5 | Mitogen-activated protein kinase kinase kinase 5 |
| MAPK14 | Mitogen-activated protein kinase 14 |
| SLC2A1 | Solute carrier family 2 member 1 |
| SLC2A3 | Solute carrier family 2 member 3 |
| SLC2A6 | Solute carrier family 2 member 6 |
| SLC2A8 | Solute carrier family 2 member 8 |
| SLC2A12 | Solute carrier family 2 member 12 |
| GLUT13 | _NA_ |
| SLC2A14 | Solute carrier family 2 member 14 |
| EIF2AK4 | Eukaryotic translation initiation factor 2 alpha kinase 4 |
| EIF2S1 | Eukaryotic translation initiation factor 2 subunit alpha |
| ATF4 | Activating transcription factor 4 |
| ALOX5 | Arachidonate 5-lipoxygenase |
| ALOX12 | Arachidonate 12-lipoxygenase, 12S type |
| ALOX15 | Arachidonate 15-lipoxygenase |
| ALOX5 | Arachidonate 5-lipoxygenase |
| ACSF2 | Acyl-CoA synthetase family member 2 |
| IREB2 | Iron responsive element binding protein 2 |
| GPX4 | Glutathione peroxidase 4 |
| HMGB1 | High mobility group box 1 |
| HMOX1 | Heme oxygenase 1 |
| NFE2L2 | Nuclear factor, erythroid 2 like 2 |
| ELAVL1 | ELAV like RNA binding protein 1 |
| SLC3A2 | Solute carrier family 3 member 2 |
| SLC7A11 | Solute carrier family 7 member 11 |
| TFAP2C | Transcription factor AP-2 gamma |
| SP1 | Sp1 transcription factor |
| HBA1 | Hemoglobin subunit alpha 1 |
| NNMT | Nicotinamide N-methyltransferase |
| PLIN4 | Perilipin 4 |
| HIC1 | HIC ZBTB transcriptional repressor 1 |
| STMN1 | Stathmin 1 |
| RRM2 | Ribonucleotide reductase regulatory subunit M2 |
| CAPG | Capping actin protein, gelsolin like |
| HNF4A | Hepatocyte nuclear factor 4 alpha |
| NGB | Neuroglobin |
| YWHAE | Tyrosine 3-monooxygenase/tryptophan 5-monooxygenase activation protein epsilon |
| GABPB1 | GA binding protein transcription factor subunit beta 1 |
| AURKA | Aurora kinase A |
| MIR4715 | microRNA 4715 |
| RIPK1 | Receptor interacting serine/threonine kinase 1 |
| PRDX1 | Peroxiredoxin 1 |
| MIR30B | microRNA 30b |

**Supplementary Table 2:** 722 FRlncRNAs by co-expression analysis.

| ferrGene | lncRNA | cor | pvalue | Regulation |
| --- | --- | --- | --- | --- |
| DUOX1 | AL451042.1 | 0.422342943 | 4.31E-23 | postive |
| NFE2L2 | PTCSC2 | 0.478479224 | 4.99E-30 | postive |
| GPX2 | PTCSC2 | 0.402014777 | 6.97E-21 | postive |
| KEAP1 | PTCSC2 | 0.437299479 | 8.14E-25 | postive |
| ATG4D | PTCSC2 | 0.450693737 | 1.96E-26 | postive |
| FANCD2 | AC090425.3 | 0.425587016 | 1.85E-23 | postive |
| HELLS | AC090425.3 | 0.563412185 | 2.63E-43 | postive |
| ISCU | AC090425.3 | 0.430980414 | 4.46E-24 | postive |
| KLHL24 | AC090425.3 | 0.50614845 | 5.97E-34 | postive |
| TUBE1 | AC090425.3 | 0.468105554 | 1.20E-28 | postive |
| ELAVL1 | AC090425.3 | 0.510064105 | 1.55E-34 | postive |
| SP1 | AC090425.3 | 0.450974458 | 1.81E-26 | postive |
| STMN1 | AC090425.3 | 0.437245295 | 8.26E-25 | postive |
| GABPB1 | AC090425.3 | 0.412338794 | 5.50E-22 | postive |
| GLS2 | AC090425.3 | 0.540940676 | 2.02E-39 | postive |
| ATG3 | AC090425.3 | 0.423941293 | 2.85E-23 | postive |
| ATG4D | AC090425.3 | 0.498009337 | 9.27E-33 | postive |
| SNX4 | AC090425.3 | 0.440517029 | 3.38E-25 | postive |
| MAPK8 | AC090425.3 | 0.435069303 | 1.49E-24 | postive |
| ACVR1B | AC090425.3 | 0.42994458 | 5.87E-24 | postive |
| CHMP6 | AP004609.5 | 0.412747725 | 4.96E-22 | postive |
| RPL8 | AP004609.5 | 0.423531483 | 3.17E-23 | postive |
| SLC2A8 | AP004609.5 | 0.451902737 | 1.39E-26 | postive |
| PHKG2 | AP004609.5 | 0.538152775 | 5.87E-39 | postive |
| PEBP1 | AP004609.5 | 0.410243347 | 9.27E-22 | postive |
| EGLN2 | AP004609.5 | 0.571116107 | 1.04E-44 | postive |
| TAZ | AP004609.5 | 0.4410897 | 2.88E-25 | postive |
| MIOX | AC079336.5 | 0.472110002 | 3.56E-29 | postive |
| GPX4 | AZIN1-AS1 | 0.427640908 | 1.08E-23 | postive |
| GLS2 | LINC01018 | 0.531713223 | 6.60E-38 | postive |
| NOX1 | ELF3-AS1 | 0.403179598 | 5.25E-21 | postive |
| HELLS | SP2-AS1 | 0.432986657 | 2.61E-24 | postive |
| TFR2 | SP2-AS1 | 0.407058125 | 2.04E-21 | postive |
| TAZ | AC245140.2 | 0.524218595 | 1.03E-36 | postive |
| PHKG2 | LINC00853 | 0.400050887 | 1.12E-20 | postive |
| EGLN2 | LINC00853 | 0.406519689 | 2.33E-21 | postive |
| ASNS | EMSLR | 0.487078057 | 3.29E-31 | postive |
| DUOX1 | PLA2G4E-AS1 | 0.527382888 | 3.26E-37 | postive |
| ALOX12B | PLA2G4E-AS1 | 0.586757057 | 1.13E-47 | postive |
| ALOXE3 | PLA2G4E-AS1 | 0.51568049 | 2.19E-35 | postive |
| DNAJB6 | PLA2G4E-AS1 | 0.41427644 | 3.38E-22 | postive |
| SLC40A1 | AC015911.3 | 0.437394803 | 7.93E-25 | postive |
| ENPP2 | AC015911.3 | 0.456097419 | 4.16E-27 | postive |
| GCH1 | AC015911.3 | 0.425688792 | 1.80E-23 | postive |
| ALOX5 | AC015911.3 | 0.472053674 | 3.62E-29 | postive |
| SP1 | AC015911.3 | 0.4051771 | 3.23E-21 | postive |
| CYBB | AC015911.3 | 0.564128581 | 1.95E-43 | postive |
| FLT3 | AC015911.3 | 0.656776977 | 3.69E-63 | postive |
| IFNG | AC015911.3 | 0.632044744 | 3.11E-57 | postive |
| TUBE1 | AC079684.2 | 0.422805669 | 3.82E-23 | postive |
| GPX4 | AC012615.1 | 0.546025655 | 2.83E-40 | postive |
| ATF4 | AC012615.1 | 0.449686206 | 2.61E-26 | postive |
| HELLS | AC012615.1 | 0.438660382 | 5.62E-25 | postive |
| ISCU | AC012615.1 | 0.48683366 | 3.56E-31 | postive |
| TUBE1 | AC012615.1 | 0.449476114 | 2.77E-26 | postive |
| ELAVL1 | AC012615.1 | 0.512184064 | 7.45E-35 | postive |
| STMN1 | AC012615.1 | 0.513884908 | 4.11E-35 | postive |
| GABPB1 | AC012615.1 | 0.408164454 | 1.55E-21 | postive |
| NOX1 | AC012615.1 | 0.469599248 | 7.63E-29 | postive |
| GLS2 | AC012615.1 | 0.407026059 | 2.05E-21 | postive |
| PHKG2 | AC012615.1 | 0.54357842 | 7.33E-40 | postive |
| ATG4D | AC012615.1 | 0.492228239 | 6.22E-32 | postive |
| MAP1LC3A | AC012615.1 | 0.431249598 | 4.15E-24 | postive |
| PEBP1 | AC012615.1 | 0.495268634 | 2.30E-32 | postive |
| TAZ | AC012615.1 | 0.463657141 | 4.53E-28 | postive |
| PANX1 | AC012615.1 | -0.417432389 | 1.52E-22 | negative |
| LONP1 | AC012615.1 | 0.417993798 | 1.32E-22 | postive |
| FANCD2 | UBL7-AS1 | 0.564368523 | 1.77E-43 | postive |
| HELLS | UBL7-AS1 | 0.55410379 | 1.16E-41 | postive |
| TUBE1 | UBL7-AS1 | 0.408756298 | 1.34E-21 | postive |
| TFRC | UBL7-AS1 | 0.417471356 | 1.50E-22 | postive |
| IREB2 | UBL7-AS1 | 0.472703391 | 2.97E-29 | postive |
| HMGB1 | UBL7-AS1 | 0.43675952 | 9.42E-25 | postive |
| ELAVL1 | UBL7-AS1 | 0.436227858 | 1.09E-24 | postive |
| SP1 | UBL7-AS1 | 0.437413881 | 7.89E-25 | postive |
| STMN1 | UBL7-AS1 | 0.430024916 | 5.75E-24 | postive |
| RRM2 | UBL7-AS1 | 0.464728474 | 3.30E-28 | postive |
| GABPB1 | UBL7-AS1 | 0.49017873 | 1.21E-31 | postive |
| GLS2 | UBL7-AS1 | 0.49834205 | 8.30E-33 | postive |
| KEAP1 | UBL7-AS1 | 0.412037099 | 5.93E-22 | postive |
| SNX4 | UBL7-AS1 | 0.436233974 | 1.09E-24 | postive |
| CAV1 | AL049775.1 | 0.504056103 | 1.22E-33 | postive |
| ANO6 | AL049775.1 | 0.413196806 | 4.43E-22 | postive |
| PANX1 | AL049775.1 | 0.431680385 | 3.70E-24 | postive |
| IL6 | AC004704.1 | 0.480939459 | 2.31E-30 | postive |
| CXCL2 | AC004704.1 | 0.406971323 | 2.08E-21 | postive |
| GPX4 | AC127024.6 | 0.401291441 | 8.30E-21 | postive |
| ATF4 | AC127024.6 | 0.402667363 | 5.95E-21 | postive |
| ISCU | AC127024.6 | 0.415189877 | 2.68E-22 | postive |
| NOX1 | AC127024.6 | 0.494171848 | 3.29E-32 | postive |
| PHKG2 | AC127024.6 | 0.575284848 | 1.75E-45 | postive |
| MAP1LC3A | AC127024.6 | 0.445816736 | 7.76E-26 | postive |
| SOCS1 | AC127024.6 | 0.419084673 | 9.97E-23 | postive |
| EGLN2 | AC127024.6 | 0.463015207 | 5.48E-28 | postive |
| TAZ | AC127024.6 | 0.455475812 | 4.98E-27 | postive |
| GPX4 | AC006449.7 | 0.443620384 | 1.43E-25 | postive |
| FANCD2 | AC006449.7 | 0.401302755 | 8.27E-21 | postive |
| HELLS | AC006449.7 | 0.428605619 | 8.38E-24 | postive |
| ISCU | AC006449.7 | 0.488612612 | 2.01E-31 | postive |
| ELAVL1 | AC006449.7 | 0.431846691 | 3.54E-24 | postive |
| STMN1 | AC006449.7 | 0.486427638 | 4.05E-31 | postive |
| NOX1 | AC006449.7 | 0.471874332 | 3.82E-29 | postive |
| GLS2 | AC006449.7 | 0.451407689 | 1.60E-26 | postive |
| KEAP1 | AC006449.7 | 0.457675906 | 2.63E-27 | postive |
| PHKG2 | AC006449.7 | 0.500587226 | 3.92E-33 | postive |
| ATG4D | AC006449.7 | 0.495398505 | 2.20E-32 | postive |
| MAP1LC3A | AC006449.7 | 0.422755345 | 3.87E-23 | postive |
| BID | AC006449.7 | 0.534078827 | 2.73E-38 | postive |
| PEBP1 | AC006449.7 | 0.487432933 | 2.94E-31 | postive |
| MIOX | AC006449.7 | 0.428570236 | 8.45E-24 | postive |
| TAZ | AC006449.7 | 0.486389044 | 4.10E-31 | postive |
| PANX1 | AC006449.7 | -0.429803103 | 6.10E-24 | negative |
| MTOR | AP000759.1 | 0.403049565 | 5.42E-21 | postive |
| RELA | AP000759.1 | 0.539142018 | 4.03E-39 | postive |
| ANO6 | AP000759.1 | 0.425931377 | 1.69E-23 | postive |
| MTOR | AC098484.1 | 0.409336452 | 1.16E-21 | postive |
| SLC2A1 | SLC2A1-AS1 | 0.428843151 | 7.87E-24 | postive |
| FANCD2 | AC131159.1 | 0.524100948 | 1.08E-36 | postive |
| HELLS | AC131159.1 | 0.631545996 | 4.05E-57 | postive |
| ISCU | AC131159.1 | 0.429704399 | 6.26E-24 | postive |
| TUBE1 | AC131159.1 | 0.412998696 | 4.66E-22 | postive |
| ELAVL1 | AC131159.1 | 0.472037987 | 3.64E-29 | postive |
| SP1 | AC131159.1 | 0.471374849 | 4.45E-29 | postive |
| STMN1 | AC131159.1 | 0.454219306 | 7.15E-27 | postive |
| GABPB1 | AC131159.1 | 0.42685878 | 1.33E-23 | postive |
| GLS2 | AC131159.1 | 0.55703621 | 3.57E-42 | postive |
| KEAP1 | AC131159.1 | 0.402902582 | 5.62E-21 | postive |
| ATG4D | AC131159.1 | 0.464287563 | 3.76E-28 | postive |
| MYB | AC131159.1 | 0.432628728 | 2.87E-24 | postive |
| MAPK8 | AC131159.1 | 0.438865171 | 5.31E-25 | postive |
| HELLS | TNRC6C-AS1 | 0.443128798 | 1.64E-25 | postive |
| ISCU | TNRC6C-AS1 | 0.564466141 | 1.70E-43 | postive |
| GCH1 | TNRC6C-AS1 | 0.415425843 | 2.53E-22 | postive |
| NOX1 | TNRC6C-AS1 | 0.440181685 | 3.70E-25 | postive |
| GLS2 | TNRC6C-AS1 | 0.495757831 | 1.95E-32 | postive |
| ATG4D | TNRC6C-AS1 | 0.478763377 | 4.57E-30 | postive |
| SOCS1 | TNRC6C-AS1 | 0.511369568 | 9.89E-35 | postive |
| MYB | TNRC6C-AS1 | 0.406927914 | 2.10E-21 | postive |
| MAPK8 | TNRC6C-AS1 | 0.439761811 | 4.15E-25 | postive |
| GPX4 | SNHG8 | 0.447726438 | 4.54E-26 | postive |
| CISD1 | SNHG8 | 0.474506815 | 1.71E-29 | postive |
| ATF4 | SNHG8 | 0.538151544 | 5.87E-39 | postive |
| DDIT3 | SNHG8 | 0.405486021 | 3.00E-21 | postive |
| RPL8 | SNHG8 | 0.57711939 | 7.91E-46 | postive |
| HRAS | SNHG8 | 0.419521785 | 8.91E-23 | postive |
| PHKG2 | SNHG8 | 0.514650044 | 3.14E-35 | postive |
| MAPK1 | SNHG8 | -0.406485811 | 2.35E-21 | negative |
| PEBP1 | SNHG8 | 0.438390089 | 6.05E-25 | postive |
| ANO6 | SNHG8 | -0.472054237 | 3.62E-29 | negative |
| EGLN2 | SNHG8 | 0.565603987 | 1.06E-43 | postive |
| TAZ | SNHG8 | 0.465470132 | 2.64E-28 | postive |
| LONP1 | SNHG8 | 0.433128585 | 2.51E-24 | postive |
| HERPUD1 | LINC02362 | 0.71632129 | 5.12E-80 | postive |
| FLT3 | LINC02362 | 0.506067942 | 6.14E-34 | postive |
| GPX2 | AC036214.2 | 0.416108707 | 2.13E-22 | postive |
| EMC2 | AC036214.2 | 0.434495263 | 1.74E-24 | postive |
| KLHL24 | LPP-AS2 | 0.510015982 | 1.58E-34 | postive |
| TFRC | LPP-AS2 | 0.45040388 | 2.13E-26 | postive |
| PIK3CA | LPP-AS2 | 0.528502678 | 2.16E-37 | postive |
| SNX4 | LPP-AS2 | 0.416198997 | 2.08E-22 | postive |
| ABCC1 | LPP-AS2 | 0.419511955 | 8.93E-23 | postive |
| YY1AP1 | LPP-AS2 | 0.419811064 | 8.27E-23 | postive |
| PHKG2 | MNX1-AS1 | 0.404122443 | 4.18E-21 | postive |
| SLC7A11 | AL033397.1 | 0.483104425 | 1.17E-30 | postive |
| FTH1 | AL033397.1 | 0.417100402 | 1.65E-22 | postive |
| TXNRD1 | AL033397.1 | 0.522184438 | 2.16E-36 | postive |
| SRXN1 | AL033397.1 | 0.450765015 | 1.92E-26 | postive |
| GPX2 | AL033397.1 | 0.471685743 | 4.05E-29 | postive |
| MAFG | AL033397.1 | 0.425166779 | 2.07E-23 | postive |
| PGD | AL033397.1 | 0.471974806 | 3.71E-29 | postive |
| ABCC1 | AL033397.1 | 0.468479168 | 1.07E-28 | postive |
| HELLS | AC008438.1 | 0.401854493 | 7.24E-21 | postive |
| ISCU | AC008438.1 | 0.453092582 | 9.88E-27 | postive |
| STMN1 | AC008438.1 | 0.446884233 | 5.75E-26 | postive |
| GLS2 | AC008438.1 | 0.408947945 | 1.28E-21 | postive |
| GOT1 | AC008438.1 | 0.463395629 | 4.90E-28 | postive |
| ATG3 | AC008438.1 | 0.453398183 | 9.05E-27 | postive |
| ATG4D | AC008438.1 | 0.474115115 | 1.93E-29 | postive |
| GPX4 | AL807752.5 | 0.454930152 | 5.83E-27 | postive |
| CISD1 | AL807752.5 | 0.477783162 | 6.20E-30 | postive |
| FANCD2 | AL807752.5 | 0.434392033 | 1.79E-24 | postive |
| TP53 | AL807752.5 | 0.410836455 | 8.00E-22 | postive |
| HELLS | AL807752.5 | 0.465396481 | 2.70E-28 | postive |
| ISCU | AL807752.5 | 0.483909008 | 9.05E-31 | postive |
| GCH1 | AL807752.5 | 0.400841182 | 9.25E-21 | postive |
| GLS2 | AL807752.5 | 0.523432472 | 1.37E-36 | postive |
| CARS1 | AL807752.5 | 0.507384956 | 3.91E-34 | postive |
| ATG4D | AL807752.5 | 0.452153145 | 1.29E-26 | postive |
| SOCS1 | AL807752.5 | 0.467856241 | 1.29E-28 | postive |
| MAPK8 | AL807752.5 | 0.427155701 | 1.23E-23 | postive |
| TUBE1 | AC004223.4 | 0.425339 | 1.98E-23 | postive |
| NOX1 | AC004223.4 | 0.407260784 | 1.94E-21 | postive |
| PHKG2 | AC004223.4 | 0.464242461 | 3.81E-28 | postive |
| TAZ | AC004223.4 | 0.446871922 | 5.77E-26 | postive |
| DUOX1 | AL512274.1 | 0.552026592 | 2.66E-41 | postive |
| ALOX12B | AL512274.1 | 0.428061487 | 9.67E-24 | postive |
| FANCD2 | PTOV1-AS2 | 0.461148909 | 9.51E-28 | postive |
| HELLS | PTOV1-AS2 | 0.528563652 | 2.11E-37 | postive |
| ISCU | PTOV1-AS2 | 0.506822291 | 4.74E-34 | postive |
| TUBE1 | PTOV1-AS2 | 0.430316889 | 5.32E-24 | postive |
| STMN1 | PTOV1-AS2 | 0.470608083 | 5.62E-29 | postive |
| GLS2 | PTOV1-AS2 | 0.423653509 | 3.07E-23 | postive |
| PHKG2 | PTOV1-AS2 | 0.41142805 | 6.90E-22 | postive |
| ATG4D | PTOV1-AS2 | 0.435419258 | 1.36E-24 | postive |
| MYB | PTOV1-AS2 | 0.436151047 | 1.11E-24 | postive |
| TAZ | PTOV1-AS2 | 0.442732666 | 1.83E-25 | postive |
| HELLS | AL354892.2 | 0.486287797 | 4.24E-31 | postive |
| FADS2 | AL354892.2 | 0.487813625 | 2.60E-31 | postive |
| KLHL24 | AL354892.2 | 0.484840941 | 6.73E-31 | postive |
| TUBE1 | AL354892.2 | 0.529420198 | 1.54E-37 | postive |
| ELAVL1 | AL354892.2 | 0.532630078 | 4.69E-38 | postive |
| STMN1 | AL354892.2 | 0.463184849 | 5.21E-28 | postive |
| GABPB1 | AL354892.2 | 0.525538542 | 6.40E-37 | postive |
| GLS2 | AL354892.2 | 0.475079256 | 1.43E-29 | postive |
| ATG4D | AL354892.2 | 0.400963031 | 8.98E-21 | postive |
| PEBP1 | AL354892.2 | 0.439007156 | 5.11E-25 | postive |
| MYB | AL354892.2 | 0.412776345 | 4.93E-22 | postive |
| LPIN1 | AL354892.2 | 0.419189065 | 9.71E-23 | postive |
| YY1AP1 | AL354892.2 | 0.484537977 | 7.41E-31 | postive |
| NFE2L2 | AC007098.1 | 0.55754715 | 2.90E-42 | postive |
| IFNG | LINC02195 | 0.641305749 | 2.18E-59 | postive |
| ATF4 | AC007038.1 | 0.402992054 | 5.50E-21 | postive |
| HELLS | AC007038.1 | 0.450794928 | 1.90E-26 | postive |
| STMN1 | AC007038.1 | 0.488776468 | 1.91E-31 | postive |
| NOX1 | AC007038.1 | 0.444707312 | 1.06E-25 | postive |
| PHKG2 | AC007038.1 | 0.53271835 | 4.54E-38 | postive |
| PEBP1 | AC007038.1 | 0.42388675 | 2.89E-23 | postive |
| TAZ | AC007038.1 | 0.511360426 | 9.92E-35 | postive |
| MT3 | MIAT | 0.466822538 | 1.76E-28 | postive |
| CDO1 | MIAT | 0.557695804 | 2.73E-42 | postive |
| ISCU | B3GALT5-AS1 | 0.42705199 | 1.26E-23 | postive |
| GLS2 | B3GALT5-AS1 | 0.448845921 | 3.31E-26 | postive |
| YY1AP1 | AC092295.2 | 0.409834428 | 1.03E-21 | postive |
| OTUB1 | LHX1-DT | 0.412232549 | 5.65E-22 | postive |
| CAV1 | LHX1-DT | 0.474428174 | 1.75E-29 | postive |
| NOX1 | AC099518.6 | 0.421675263 | 5.12E-23 | postive |
| PHKG2 | AC099518.6 | 0.414059497 | 3.57E-22 | postive |
| TAZ | AC099518.6 | 0.439400629 | 4.59E-25 | postive |
| MAP1LC3A | AC093702.1 | 0.461060857 | 9.76E-28 | postive |
| GPX4 | AC106795.2 | 0.443068248 | 1.67E-25 | postive |
| ISCU | AC106795.2 | 0.450422654 | 2.12E-26 | postive |
| AIFM2 | AC106795.2 | 0.403567489 | 4.78E-21 | postive |
| MT3 | AC106795.2 | 0.533408004 | 3.51E-38 | postive |
| ATG4D | AC106795.2 | 0.420010793 | 7.86E-23 | postive |
| MAP1LC3A | AC106795.2 | 0.453601964 | 8.54E-27 | postive |
| PEBP1 | AC106795.2 | 0.445757345 | 7.89E-26 | postive |
| SOCS1 | AC106795.2 | 0.422829047 | 3.80E-23 | postive |
| MIOX | AC106795.2 | 0.416099142 | 2.13E-22 | postive |
| GPX4 | AC087752.4 | 0.405243565 | 3.18E-21 | postive |
| HELLS | AC087752.4 | 0.478821927 | 4.48E-30 | postive |
| ISCU | AC087752.4 | 0.492335367 | 6.01E-32 | postive |
| STMN1 | AC087752.4 | 0.501055754 | 3.35E-33 | postive |
| NOX1 | AC087752.4 | 0.481896792 | 1.71E-30 | postive |
| PHKG2 | AC087752.4 | 0.416619544 | 1.87E-22 | postive |
| PEBP1 | AC087752.4 | 0.400026533 | 1.12E-20 | postive |
| MYB | AC087752.4 | 0.411245951 | 7.22E-22 | postive |
| TUBE1 | ERVK13-1 | 0.474736147 | 1.59E-29 | postive |
| MYB | ERVK13-1 | 0.401497559 | 7.89E-21 | postive |
| DPP4 | LINC01694 | 0.400523578 | 9.98E-21 | postive |
| MIOX | LINC01694 | 0.423383785 | 3.29E-23 | postive |
| RPL8 | AC004816.2 | 0.426608311 | 1.42E-23 | postive |
| PHKG2 | AC004816.2 | 0.455791995 | 4.54E-27 | postive |
| EGLN2 | AC004816.2 | 0.624111161 | 1.91E-55 | postive |
| SLC2A3 | MRPS9-AS1 | 0.421871681 | 4.87E-23 | postive |
| RPL8 | AL023803.1 | 0.444215235 | 1.21E-25 | postive |
| SLC2A8 | AL023803.1 | 0.53007674 | 1.21E-37 | postive |
| PHKG2 | AL023803.1 | 0.459545484 | 1.52E-27 | postive |
| EGLN2 | AL023803.1 | 0.544887477 | 4.41E-40 | postive |
| TAZ | AL023803.1 | 0.432962213 | 2.63E-24 | postive |
| PHKG2 | AC064836.2 | 0.423194685 | 3.46E-23 | postive |
| EGLN2 | AC064836.2 | 0.470585871 | 5.66E-29 | postive |
| TAZ | AC064836.2 | 0.433454513 | 2.30E-24 | postive |
| NFE2L2 | LINC00630 | 0.405669912 | 2.86E-21 | postive |
| HELLS | LINC00630 | 0.430480936 | 5.09E-24 | postive |
| BRD4 | LINC00630 | 0.466575664 | 1.90E-28 | postive |
| LAMP2 | LINC00630 | 0.431082367 | 4.34E-24 | postive |
| KLHL24 | LINC00630 | 0.426215487 | 1.57E-23 | postive |
| TUBE1 | LINC00630 | 0.419010931 | 1.02E-22 | postive |
| SETD1B | LINC00630 | 0.450247273 | 2.23E-26 | postive |
| MAFG | LINC00630 | 0.458446341 | 2.10E-27 | postive |
| EIF2AK4 | LINC00630 | 0.411576765 | 6.65E-22 | postive |
| IREB2 | LINC00630 | 0.463502513 | 4.75E-28 | postive |
| ELAVL1 | LINC00630 | 0.421668693 | 5.13E-23 | postive |
| SP1 | LINC00630 | 0.504598624 | 1.01E-33 | postive |
| CS | LINC00630 | 0.45461981 | 6.37E-27 | postive |
| PIK3CA | LINC00630 | 0.441698596 | 2.44E-25 | postive |
| LPCAT3 | LINC00630 | 0.577545757 | 6.58E-46 | postive |
| BECN1 | LINC00630 | 0.404682435 | 3.65E-21 | postive |
| MAPK8 | LINC00630 | 0.429069363 | 7.41E-24 | postive |
| PRKAA1 | LINC00630 | 0.431945289 | 3.45E-24 | postive |
| ABCC1 | LINC00630 | 0.408098967 | 1.58E-21 | postive |
| ATM | LINC00630 | 0.432151426 | 3.26E-24 | postive |
| YY1AP1 | LINC00630 | 0.438825668 | 5.37E-25 | postive |
| SIRT1 | LINC00630 | 0.478921351 | 4.35E-30 | postive |
| GCLC | AC125257.1 | 0.433243451 | 2.44E-24 | postive |
| NFE2L2 | AC125257.1 | 0.484898253 | 6.61E-31 | postive |
| HELLS | AC125257.1 | 0.494760809 | 2.72E-32 | postive |
| PSAT1 | AC125257.1 | 0.472837203 | 2.85E-29 | postive |
| KLHL24 | AC125257.1 | 0.452703643 | 1.10E-26 | postive |
| TUBE1 | AC125257.1 | 0.58791925 | 6.71E-48 | postive |
| MAFG | AC125257.1 | 0.401537849 | 7.82E-21 | postive |
| ELAVL1 | AC125257.1 | 0.592350119 | 8.99E-49 | postive |
| GABPB1 | AC125257.1 | 0.485609752 | 5.26E-31 | postive |
| NOX1 | AC125257.1 | 0.454633024 | 6.35E-27 | postive |
| GLS2 | AC125257.1 | 0.433086447 | 2.54E-24 | postive |
| ATG4D | AC125257.1 | 0.452084762 | 1.32E-26 | postive |
| BID | AC125257.1 | 0.444230423 | 1.21E-25 | postive |
| YY1AP1 | AC125257.1 | 0.406800343 | 2.17E-21 | postive |
| TXNRD1 | AL590666.2 | 0.464525362 | 3.50E-28 | postive |
| SRXN1 | AL590666.2 | 0.4448666 | 1.01E-25 | postive |
| MAFG | AL590666.2 | 0.574662688 | 2.28E-45 | postive |
| SLC2A12 | AL590666.2 | 0.461811128 | 7.83E-28 | postive |
| ABCC1 | AL590666.2 | 0.407769996 | 1.71E-21 | postive |
| NFE2L2 | AC074117.1 | 0.430559662 | 4.99E-24 | postive |
| BRD4 | AC074117.1 | 0.40630149 | 2.45E-21 | postive |
| TUBE1 | AC074117.1 | 0.439565532 | 4.38E-25 | postive |
| PHKG2 | AC012676.1 | 0.459476225 | 1.55E-27 | postive |
| HELLS | EP300-AS1 | 0.480054412 | 3.05E-30 | postive |
| GCH1 | EP300-AS1 | 0.48545118 | 5.54E-31 | postive |
| SP1 | EP300-AS1 | 0.441290323 | 2.73E-25 | postive |
| GLS2 | EP300-AS1 | 0.470137424 | 6.49E-29 | postive |
| PHKG2 | AC005899.7 | 0.429947149 | 5.87E-24 | postive |
| PML | HCP5 | 0.51620639 | 1.82E-35 | postive |
| ALOX5 | LINC02345 | 0.444218799 | 1.21E-25 | postive |
| CYBB | LINC02345 | 0.460375071 | 1.19E-27 | postive |
| ALOX12B | AL034376.2 | 0.470342169 | 6.10E-29 | postive |
| ALOXE3 | AL034376.2 | 0.431427903 | 3.96E-24 | postive |
| NOX1 | POLR2J4 | 0.412781498 | 4.92E-22 | postive |
| PHKG2 | AL121601.1 | 0.438868366 | 5.31E-25 | postive |
| TUBE1 | LINC00265 | 0.415003203 | 2.81E-22 | postive |
| SETD1B | LINC00265 | 0.509900349 | 1.65E-34 | postive |
| IREB2 | LINC00265 | 0.437601458 | 7.50E-25 | postive |
| SP1 | LINC00265 | 0.504001742 | 1.24E-33 | postive |
| ACVR1B | LINC00265 | 0.475281394 | 1.35E-29 | postive |
| ATM | LINC00265 | 0.427313163 | 1.18E-23 | postive |
| YY1AP1 | LINC00265 | 0.435249504 | 1.42E-24 | postive |
| HIC1 | LINC01614 | 0.482150438 | 1.58E-30 | postive |
| NOX4 | LINC01614 | 0.718785589 | 8.28E-81 | postive |
| ZEB1 | LINC01614 | 0.470239451 | 6.29E-29 | postive |
| TUBE1 | LINC00526 | 0.470939449 | 5.08E-29 | postive |
| STMN1 | LINC00526 | 0.438326808 | 6.15E-25 | postive |
| GLS2 | LINC00526 | 0.415715883 | 2.35E-22 | postive |
| HERPUD1 | COPDA1 | 0.512836726 | 5.93E-35 | postive |
| FLT3 | COPDA1 | 0.561162942 | 6.63E-43 | postive |
| ENPP2 | AP001189.3 | 0.463402686 | 4.89E-28 | postive |
| ALOX5 | AP001189.3 | 0.468562636 | 1.04E-28 | postive |
| HIC1 | AP001189.3 | 0.556245347 | 4.91E-42 | postive |
| CYBB | AP001189.3 | 0.449946324 | 2.42E-26 | postive |
| NOX4 | AP001189.3 | 0.586086977 | 1.53E-47 | postive |
| ZEB1 | AP001189.3 | 0.781494853 | 2.66E-104 | postive |
| ANO6 | AP001189.3 | 0.424522471 | 2.45E-23 | postive |
| TLR4 | AP001189.3 | 0.542780168 | 9.98E-40 | postive |
| ISCU | AC107464.2 | 0.465155708 | 2.90E-28 | postive |
| STMN1 | AC107464.2 | 0.557530702 | 2.92E-42 | postive |
| PHKG2 | AC107464.2 | 0.458636436 | 1.99E-27 | postive |
| SOCS1 | AC107464.2 | 0.449479571 | 2.77E-26 | postive |
| MYB | AC107464.2 | 0.419643173 | 8.64E-23 | postive |
| CHMP5 | EBLN3P | 0.411352043 | 7.04E-22 | postive |
| SP1 | EBLN3P | 0.40580508 | 2.77E-21 | postive |
| ACO1 | EBLN3P | 0.453288599 | 9.34E-27 | postive |
| GPX4 | AC133552.5 | 0.482989293 | 1.21E-30 | postive |
| CHMP6 | AC133552.5 | 0.425363196 | 1.96E-23 | postive |
| RPL8 | AC133552.5 | 0.412897984 | 4.78E-22 | postive |
| PHKG2 | AC133552.5 | 0.604056766 | 3.82E-51 | postive |
| EGLN2 | AC133552.5 | 0.632070151 | 3.07E-57 | postive |
| TAZ | AC133552.5 | 0.4472152 | 5.24E-26 | postive |
| LONP1 | AC133552.5 | 0.40013399 | 1.10E-20 | postive |
| ISCU | AC009318.3 | 0.423700187 | 3.03E-23 | postive |
| NOX1 | AC009318.3 | 0.429673201 | 6.31E-24 | postive |
| MUC1 | SLCO4A1-AS1 | 0.408209513 | 1.53E-21 | postive |
| GPX4 | AC008443.5 | 0.453630173 | 8.47E-27 | postive |
| RPL8 | AC008443.5 | 0.452350503 | 1.22E-26 | postive |
| SLC2A8 | AC008443.5 | 0.427009094 | 1.28E-23 | postive |
| STMN1 | AC008443.5 | 0.423455085 | 3.23E-23 | postive |
| PHKG2 | AC008443.5 | 0.481301652 | 2.06E-30 | postive |
| EGLN2 | AC008443.5 | 0.516863189 | 1.44E-35 | postive |
| TAZ | AC008443.5 | 0.447944125 | 4.27E-26 | postive |
| ATF4 | AC132192.2 | 0.491459638 | 7.99E-32 | postive |
| ISCU | AC132192.2 | 0.454610703 | 6.39E-27 | postive |
| ACSF2 | AC132192.2 | 0.408444627 | 1.45E-21 | postive |
| STMN1 | AC132192.2 | 0.443134075 | 1.64E-25 | postive |
| NOX1 | AC132192.2 | 0.42823794 | 9.23E-24 | postive |
| CARS1 | AC132192.2 | 0.400938445 | 9.03E-21 | postive |
| PHKG2 | AC132192.2 | 0.590774163 | 1.84E-48 | postive |
| EGLN2 | AC132192.2 | 0.44106611 | 2.90E-25 | postive |
| TAZ | AC132192.2 | 0.575974535 | 1.30E-45 | postive |
| GPT2 | AC108134.1 | 0.43356985 | 2.23E-24 | postive |
| DUOX1 | AC108134.1 | 0.487479306 | 2.89E-31 | postive |
| ALOX12B | AC108134.1 | 0.509173807 | 2.11E-34 | postive |
| KLHL24 | AC092910.3 | 0.404061534 | 4.24E-21 | postive |
| SP1 | AC092910.3 | 0.400668724 | 9.64E-21 | postive |
| ATG3 | AC092910.3 | 0.515764504 | 2.12E-35 | postive |
| SNX4 | AC092910.3 | 0.557814691 | 2.60E-42 | postive |
| ACVR1B | AC092910.3 | 0.41051717 | 8.66E-22 | postive |
| PHKG2 | AC108488.1 | 0.538582768 | 4.98E-39 | postive |
| MAP1LC3A | AC108488.1 | 0.455286764 | 5.26E-27 | postive |
| MIOX | AC108488.1 | 0.500972071 | 3.45E-33 | postive |
| TAZ | AC108488.1 | 0.448995103 | 3.17E-26 | postive |
| ALOX5 | AC008105.3 | 0.447290287 | 5.13E-26 | postive |
| FLT3 | AC008105.3 | 0.459740339 | 1.44E-27 | postive |
| IFNG | AC008105.3 | 0.427139167 | 1.23E-23 | postive |
| CAPG | LINC01871 | 0.415027672 | 2.80E-22 | postive |
| IFNG | LINC01871 | 0.741458839 | 1.64E-88 | postive |
| TUBE1 | AL137003.1 | 0.540408187 | 2.48E-39 | postive |
| MYB | AL137003.1 | 0.441443725 | 2.62E-25 | postive |
| CD44 | CD44-AS1 | 0.468046981 | 1.22E-28 | postive |
| ATF4 | AC116913.1 | 0.416894709 | 1.74E-22 | postive |
| HELLS | AC116913.1 | 0.413713941 | 3.89E-22 | postive |
| ISCU | AC116913.1 | 0.435654124 | 1.27E-24 | postive |
| SLC2A8 | AC116913.1 | 0.430376696 | 5.24E-24 | postive |
| STMN1 | AC116913.1 | 0.462564264 | 6.27E-28 | postive |
| GABPB1 | AC116913.1 | 0.425091188 | 2.11E-23 | postive |
| NOX1 | AC116913.1 | 0.50121307 | 3.18E-33 | postive |
| PHKG2 | AC116913.1 | 0.570709641 | 1.24E-44 | postive |
| TAZ | AC116913.1 | 0.555789559 | 5.90E-42 | postive |
| SLC2A1 | SLC9A3-AS1 | 0.42532986 | 1.98E-23 | postive |
| ATF4 | SNHG7 | 0.515539031 | 2.30E-35 | postive |
| RPL8 | SNHG7 | 0.498922347 | 6.84E-33 | postive |
| SLC2A8 | SNHG7 | 0.412950387 | 4.72E-22 | postive |
| PHKG2 | SNHG7 | 0.480344389 | 2.79E-30 | postive |
| EGLN2 | SNHG7 | 0.407769584 | 1.71E-21 | postive |
| TAZ | SNHG7 | 0.465696328 | 2.47E-28 | postive |
| GPX4 | EPB41L4A-AS1 | 0.478932736 | 4.33E-30 | postive |
| CISD1 | EPB41L4A-AS1 | 0.438839763 | 5.35E-25 | postive |
| ATF4 | EPB41L4A-AS1 | 0.502317155 | 2.19E-33 | postive |
| ISCU | EPB41L4A-AS1 | 0.477854385 | 6.06E-30 | postive |
| RPL8 | EPB41L4A-AS1 | 0.481645469 | 1.85E-30 | postive |
| HMGB1 | EPB41L4A-AS1 | 0.445274773 | 9.03E-26 | postive |
| STMN1 | EPB41L4A-AS1 | 0.441406605 | 2.64E-25 | postive |
| NOX1 | EPB41L4A-AS1 | 0.41114386 | 7.41E-22 | postive |
| PHKG2 | EPB41L4A-AS1 | 0.414347801 | 3.32E-22 | postive |
| PEBP1 | EPB41L4A-AS1 | 0.423082157 | 3.56E-23 | postive |
| TAZ | EPB41L4A-AS1 | 0.437346746 | 8.03E-25 | postive |
| PHKG2 | AC007773.1 | 0.459684084 | 1.46E-27 | postive |
| PEBP1 | AC007773.1 | 0.413206207 | 4.42E-22 | postive |
| EGLN2 | AC007773.1 | 0.459754518 | 1.43E-27 | postive |
| SP1 | AC138956.2 | 0.41799952 | 1.32E-22 | postive |
| ACVR1B | AC138956.2 | 0.45616882 | 4.07E-27 | postive |
| PHKG2 | AL023284.4 | 0.403099885 | 5.36E-21 | postive |
| ATF4 | LINC01578 | 0.418720433 | 1.09E-22 | postive |
| DDIT3 | LINC01578 | 0.456092182 | 4.16E-27 | postive |
| GABPB1 | LINC01578 | 0.459394205 | 1.59E-27 | postive |
| NOX1 | LINC01578 | 0.411175116 | 7.35E-22 | postive |
| FANCD2 | AC012313.6 | 0.442459431 | 1.98E-25 | postive |
| TP53 | AC012313.6 | 0.449296461 | 2.91E-26 | postive |
| HELLS | AC012313.6 | 0.540633397 | 2.28E-39 | postive |
| ISCU | AC012313.6 | 0.49983318 | 5.05E-33 | postive |
| STMN1 | AC012313.6 | 0.483769068 | 9.46E-31 | postive |
| NOX1 | AC012313.6 | 0.413938512 | 3.68E-22 | postive |
| GLS2 | AC012313.6 | 0.567512177 | 4.76E-44 | postive |
| ATG4D | AC012313.6 | 0.417978084 | 1.32E-22 | postive |
| MYB | AC012313.6 | 0.49571177 | 1.98E-32 | postive |
| MAPK8 | AC012313.6 | 0.501592641 | 2.80E-33 | postive |
| SLC2A8 | AC011444.1 | 0.420981813 | 6.12E-23 | postive |
| STMN1 | AC011444.1 | 0.534532654 | 2.30E-38 | postive |
| PHKG2 | AC011444.1 | 0.506452802 | 5.38E-34 | postive |
| PEBP1 | AC011444.1 | 0.45094317 | 1.83E-26 | postive |
| TAZ | AC011444.1 | 0.462812609 | 5.82E-28 | postive |
| CHMP6 | AC010654.1 | 0.42719195 | 1.22E-23 | postive |
| PHKG2 | AC010654.1 | 0.514206975 | 3.67E-35 | postive |
| TUBE1 | CHROMR | 0.414478929 | 3.21E-22 | postive |
| FANCD2 | AC020907.1 | 0.407663143 | 1.76E-21 | postive |
| HELLS | AC020907.1 | 0.44047956 | 3.41E-25 | postive |
| GLS2 | AC020907.1 | 0.443685803 | 1.41E-25 | postive |
| KEAP1 | AC020907.1 | 0.413017538 | 4.64E-22 | postive |
| ATG4D | AC020907.1 | 0.460734847 | 1.07E-27 | postive |
| CDKN2A | AC020907.1 | 0.41041209 | 8.89E-22 | postive |
| YY1AP1 | AC020907.1 | 0.444777744 | 1.04E-25 | postive |
| STMN1 | FOXD2-AS1 | 0.408196972 | 1.54E-21 | postive |
| FLT3 | AC133644.1 | 0.437913249 | 6.89E-25 | postive |
| AKR1C1 | AP000688.1 | 0.554515728 | 9.85E-42 | postive |
| AKR1C2 | AP000688.1 | 0.47058115 | 5.67E-29 | postive |
| AKR1C3 | AP000688.1 | 0.513790439 | 4.25E-35 | postive |
| GPX2 | AP000688.1 | 0.477525975 | 6.71E-30 | postive |
| PRDX1 | AP000688.1 | 0.452781807 | 1.08E-26 | postive |
| PGD | AP000688.1 | 0.439846032 | 4.06E-25 | postive |
| ABCC1 | AP000688.1 | 0.421173004 | 5.83E-23 | postive |
| GPX4 | Z84492.1 | 0.440473679 | 3.42E-25 | postive |
| ISCU | Z84492.1 | 0.421649284 | 5.16E-23 | postive |
| STMN1 | Z84492.1 | 0.409060875 | 1.24E-21 | postive |
| PHKG2 | Z84492.1 | 0.522759367 | 1.75E-36 | postive |
| MAP1LC3A | Z84492.1 | 0.483333018 | 1.09E-30 | postive |
| TAZ | Z84492.1 | 0.472999827 | 2.71E-29 | postive |
| KEAP1 | ILF3-DT | 0.417736583 | 1.41E-22 | postive |
| PHKG2 | ILF3-DT | 0.500891209 | 3.54E-33 | postive |
| EGLN2 | ILF3-DT | 0.424194135 | 2.66E-23 | postive |
| TAZ | ILF3-DT | 0.458782349 | 1.90E-27 | postive |
| LONP1 | ILF3-DT | 0.443359986 | 1.54E-25 | postive |
| GPX4 | AC073896.2 | 0.496948763 | 1.32E-32 | postive |
| CISD1 | AC073896.2 | 0.432408261 | 3.05E-24 | postive |
| FH | AC073896.2 | 0.412011928 | 5.97E-22 | postive |
| ISCU | AC073896.2 | 0.51489536 | 2.88E-35 | postive |
| SLC2A8 | AC073896.2 | 0.433485015 | 2.28E-24 | postive |
| STMN1 | AC073896.2 | 0.516330176 | 1.74E-35 | postive |
| NOX1 | AC073896.2 | 0.538240532 | 5.67E-39 | postive |
| GOT1 | AC073896.2 | 0.434815078 | 1.60E-24 | postive |
| PHKG2 | AC073896.2 | 0.511890707 | 8.25E-35 | postive |
| PEBP1 | AC073896.2 | 0.409334999 | 1.16E-21 | postive |
| EGLN2 | AC073896.2 | 0.506281741 | 5.71E-34 | postive |
| TAZ | AC073896.2 | 0.549560696 | 7.08E-41 | postive |
| PANX1 | AC073896.2 | -0.406210712 | 2.51E-21 | negative |
| HELLS | AC107068.1 | 0.448164674 | 4.01E-26 | postive |
| ELAVL1 | AC107068.1 | 0.409638548 | 1.08E-21 | postive |
| MAPK8 | AC107068.1 | 0.41745476 | 1.51E-22 | postive |
| GPX4 | AC026803.2 | 0.445300686 | 8.96E-26 | postive |
| PHKG2 | AC026803.2 | 0.50197112 | 2.46E-33 | postive |
| MAP1LC3A | AC026803.2 | 0.431855944 | 3.53E-24 | postive |
| EGLN2 | AC026803.2 | 0.425854888 | 1.73E-23 | postive |
| TAZ | AC026803.2 | 0.454505297 | 6.59E-27 | postive |
| CHMP6 | AC003101.2 | 0.457735481 | 2.58E-27 | postive |
| PHKG2 | AC003101.2 | 0.457192788 | 3.03E-27 | postive |
| EGLN2 | AC003101.2 | 0.52749287 | 3.13E-37 | postive |
| IREB2 | ST20-AS1 | 0.402537505 | 6.14E-21 | postive |
| STMN1 | SBNO1-AS1 | 0.447216407 | 5.24E-26 | postive |
| PHKG2 | SBNO1-AS1 | 0.542810088 | 9.86E-40 | postive |
| PEBP1 | SBNO1-AS1 | 0.532502732 | 4.92E-38 | postive |
| TAZ | SBNO1-AS1 | 0.462529525 | 6.33E-28 | postive |
| MAP1LC3A | MIR99AHG | 0.405991277 | 2.65E-21 | postive |
| IFNG | MMP25-AS1 | 0.410126118 | 9.55E-22 | postive |
| NFE2L2 | AC019080.3 | 0.512505899 | 6.66E-35 | postive |
| NFE2L2 | AC138305.1 | 0.449326757 | 2.89E-26 | postive |
| GPX4 | MELTF-AS1 | 0.402416413 | 6.32E-21 | postive |
| HELLS | MELTF-AS1 | 0.409972183 | 9.92E-22 | postive |
| SLC2A8 | MELTF-AS1 | 0.464050844 | 4.03E-28 | postive |
| ELAVL1 | MELTF-AS1 | 0.462060378 | 7.27E-28 | postive |
| STMN1 | MELTF-AS1 | 0.560680052 | 8.09E-43 | postive |
| NOX1 | MELTF-AS1 | 0.412885555 | 4.79E-22 | postive |
| PHKG2 | MELTF-AS1 | 0.5382178 | 5.72E-39 | postive |
| PEBP1 | MELTF-AS1 | 0.441614174 | 2.50E-25 | postive |
| SOCS1 | MELTF-AS1 | 0.422272802 | 4.39E-23 | postive |
| TAZ | MELTF-AS1 | 0.491558854 | 7.74E-32 | postive |
| ISCU | AC015802.3 | 0.429883955 | 5.97E-24 | postive |
| SOCS1 | AC015802.3 | 0.413349789 | 4.27E-22 | postive |
| MYB | AC015802.3 | 0.418458145 | 1.17E-22 | postive |
| STMN1 | AC107375.1 | 0.435633628 | 1.28E-24 | postive |
| GABPB1 | AC107375.1 | 0.424036004 | 2.78E-23 | postive |
| HELLS | MAP3K14-AS1 | 0.408744914 | 1.34E-21 | postive |
| KLHL24 | MAP3K14-AS1 | 0.485050289 | 6.29E-31 | postive |
| TUBE1 | MAP3K14-AS1 | 0.409062351 | 1.24E-21 | postive |
| SP1 | MAP3K14-AS1 | 0.465182546 | 2.88E-28 | postive |
| GLS2 | MAP3K14-AS1 | 0.463040697 | 5.44E-28 | postive |
| MAPK8 | MAP3K14-AS1 | 0.453520486 | 8.74E-27 | postive |
| ACVR1B | MAP3K14-AS1 | 0.420296624 | 7.30E-23 | postive |
| LPIN1 | MAP3K14-AS1 | 0.410653988 | 8.37E-22 | postive |
| ATM | MAP3K14-AS1 | 0.452646713 | 1.12E-26 | postive |
| YY1AP1 | MAP3K14-AS1 | 0.462811967 | 5.82E-28 | postive |
| SIRT1 | MAP3K14-AS1 | 0.49319222 | 4.54E-32 | postive |
| SLC2A3 | AC011352.3 | 0.438154267 | 6.45E-25 | postive |
| NOX4 | AC011352.3 | 0.510165473 | 1.50E-34 | postive |
| SP1 | AC005034.6 | 0.539608174 | 3.37E-39 | postive |
| MAPK8 | AC005034.6 | 0.441730795 | 2.42E-25 | postive |
| SIRT1 | AC005034.6 | 0.456710617 | 3.48E-27 | postive |
| GPX4 | AC107952.2 | 0.42291523 | 3.72E-23 | postive |
| FANCD2 | AC107952.2 | 0.411471384 | 6.83E-22 | postive |
| ISCU | AC107952.2 | 0.479275655 | 3.89E-30 | postive |
| MT3 | AC107952.2 | 0.498792438 | 7.15E-33 | postive |
| STMN1 | AC107952.2 | 0.518939508 | 6.89E-36 | postive |
| NOX1 | AC107952.2 | 0.542405948 | 1.15E-39 | postive |
| ATG4D | AC107952.2 | 0.41048468 | 8.73E-22 | postive |
| PEBP1 | AC107952.2 | 0.467334563 | 1.51E-28 | postive |
| SOCS1 | AC107952.2 | 0.490772308 | 9.99E-32 | postive |
| MYB | AC107952.2 | 0.518321575 | 8.59E-36 | postive |
| ATG3 | LINC02057 | 0.428945814 | 7.65E-24 | postive |
| ATF4 | AC092718.4 | 0.403274436 | 5.14E-21 | postive |
| STMN1 | AC092718.4 | 0.482598436 | 1.37E-30 | postive |
| NOX1 | AC092718.4 | 0.520867741 | 3.46E-36 | postive |
| PHKG2 | AC092718.4 | 0.428613612 | 8.36E-24 | postive |
| ATG3 | AC092718.4 | 0.41103428 | 7.62E-22 | postive |
| CDKN2A | AC092718.4 | 0.457520804 | 2.75E-27 | postive |
| HELLS | AL390778.2 | 0.406408998 | 2.39E-21 | postive |
| ISCU | AL390778.2 | 0.412082471 | 5.86E-22 | postive |
| GLS2 | AL390778.2 | 0.498750993 | 7.25E-33 | postive |
| MAPK8 | AL390778.2 | 0.496724238 | 1.42E-32 | postive |
| TFR2 | AC133785.1 | 0.453796604 | 8.07E-27 | postive |
| HELLS | AL021707.3 | 0.436272623 | 1.08E-24 | postive |
| TUBE1 | AL021707.3 | 0.446984696 | 5.59E-26 | postive |
| SETD1B | AL021707.3 | 0.459793665 | 1.42E-27 | postive |
| SP1 | AL021707.3 | 0.49235406 | 5.97E-32 | postive |
| GLS2 | AL021707.3 | 0.424425938 | 2.51E-23 | postive |
| SNX4 | AL021707.3 | 0.404203337 | 4.10E-21 | postive |
| ACVR1B | AL021707.3 | 0.465926011 | 2.31E-28 | postive |
| YY1AP1 | AL021707.3 | 0.415586418 | 2.43E-22 | postive |
| HELLS | AC009120.2 | 0.502436224 | 2.11E-33 | postive |
| ISCU | AC009120.2 | 0.426916642 | 1.31E-23 | postive |
| TUBE1 | AC009120.2 | 0.46980447 | 7.17E-29 | postive |
| STMN1 | AC009120.2 | 0.427848713 | 1.02E-23 | postive |
| PHKG2 | AC009120.2 | 0.470475493 | 5.85E-29 | postive |
| TAZ | AC009120.2 | 0.448435535 | 3.72E-26 | postive |
| TUBE1 | NIFK-AS1 | 0.42625576 | 1.55E-23 | postive |
| STMN1 | NIFK-AS1 | 0.417500433 | 1.49E-22 | postive |
| PHKG2 | NIFK-AS1 | 0.416907297 | 1.74E-22 | postive |
| TAZ | NIFK-AS1 | 0.425999898 | 1.66E-23 | postive |
| PHKG2 | AC008267.5 | 0.422157176 | 4.52E-23 | postive |
| PEBP1 | AC008267.5 | 0.42389225 | 2.88E-23 | postive |
| TAZ | AC008267.5 | 0.523324565 | 1.43E-36 | postive |
| HELLS | AC011461.1 | 0.44425052 | 1.20E-25 | postive |
| KEAP1 | AC011461.1 | 0.46801313 | 1.23E-28 | postive |
| ATG4D | AC011461.1 | 0.492946873 | 4.92E-32 | postive |
| ARRDC3 | AC254633.1 | 0.411902796 | 6.13E-22 | postive |
| SP1 | AC254633.1 | 0.473907289 | 2.05E-29 | postive |
| ACVR1B | AC254633.1 | 0.423205239 | 3.45E-23 | postive |
| NOS2 | AC008759.3 | 0.424539269 | 2.44E-23 | postive |
| ATF4 | SNHG25 | 0.413495617 | 4.11E-22 | postive |
| RPL8 | SNHG25 | 0.637529541 | 1.68E-58 | postive |
| SLC2A8 | SNHG25 | 0.4672669 | 1.54E-28 | postive |
| HRAS | SNHG25 | 0.450091328 | 2.33E-26 | postive |
| PHKG2 | SNHG25 | 0.545719921 | 3.19E-40 | postive |
| ANO6 | SNHG25 | -0.426230812 | 1.56E-23 | negative |
| EGLN2 | SNHG25 | 0.649078698 | 2.96E-61 | postive |
| TAZ | SNHG25 | 0.506286072 | 5.70E-34 | postive |
| GPX4 | AP000223.1 | 0.440151298 | 3.73E-25 | postive |
| ISCU | AP000223.1 | 0.504966112 | 8.94E-34 | postive |
| STMN1 | AP000223.1 | 0.42552646 | 1.88E-23 | postive |
| NOX1 | AP000223.1 | 0.537920147 | 6.41E-39 | postive |
| CDKN2A | AP000223.1 | 0.403025572 | 5.46E-21 | postive |
| HELLS | ANK3-DT | 0.446880099 | 5.76E-26 | postive |
| GLS2 | ANK3-DT | 0.406262926 | 2.48E-21 | postive |
| SETD1B | AC253536.3 | 0.469306185 | 8.34E-29 | postive |
| SP1 | AC253536.3 | 0.472012119 | 3.67E-29 | postive |
| ACVR1B | AC253536.3 | 0.407708457 | 1.74E-21 | postive |
| ATM | AC253536.3 | 0.468777165 | 9.79E-29 | postive |
| HELLS | AC109460.2 | 0.490549488 | 1.07E-31 | postive |
| TUBE1 | AC109460.2 | 0.507806599 | 3.39E-34 | postive |
| SETD1B | AC109460.2 | 0.414532827 | 3.17E-22 | postive |
| SP1 | AC109460.2 | 0.442271031 | 2.08E-25 | postive |
| SNX4 | AC109460.2 | 0.407405268 | 1.87E-21 | postive |
| ACVR1B | AC109460.2 | 0.462654622 | 6.10E-28 | postive |
| LPIN1 | AC109460.2 | 0.414074556 | 3.56E-22 | postive |
| ATM | AC109460.2 | 0.423866259 | 2.90E-23 | postive |
| STMN1 | LINC01786 | 0.407708413 | 1.74E-21 | postive |
| PHKG2 | LINC01786 | 0.479428822 | 3.71E-30 | postive |
| TAZ | LINC01786 | 0.443546413 | 1.46E-25 | postive |
| HSPB1 | AC011472.2 | 0.440200825 | 3.68E-25 | postive |
| MTOR | AC011472.2 | -0.409990714 | 9.87E-22 | negative |
| RPL8 | AC011472.2 | 0.456543514 | 3.65E-27 | postive |
| HRAS | AC011472.2 | 0.420642496 | 6.68E-23 | postive |
| PHKG2 | AC011472.2 | 0.52127095 | 2.99E-36 | postive |
| ANO6 | AC011472.2 | -0.442804388 | 1.80E-25 | negative |
| EGLN2 | AC011472.2 | 0.563428725 | 2.61E-43 | postive |
| TAZ | AC011472.2 | 0.401647216 | 7.61E-21 | postive |
| GPX4 | AC008608.2 | 0.471949236 | 3.74E-29 | postive |
| RB1 | AC008608.2 | -0.403865046 | 4.45E-21 | negative |
| CHMP6 | AC008608.2 | 0.497782775 | 1.00E-32 | postive |
| RPL8 | AC008608.2 | 0.46427958 | 3.77E-28 | postive |
| PHKG2 | AC008608.2 | 0.617052304 | 6.77E-54 | postive |
| MAP1LC3A | AC008608.2 | 0.403733711 | 4.59E-21 | postive |
| EGLN2 | AC008608.2 | 0.585581605 | 1.91E-47 | postive |
| TAZ | AC008608.2 | 0.457605704 | 2.68E-27 | postive |
| SETD1B | HCG11 | 0.406627507 | 2.27E-21 | postive |
| SIRT1 | HCG11 | 0.416863598 | 1.76E-22 | postive |
| HELLS | AC012615.6 | 0.403909174 | 4.40E-21 | postive |
| TUBE1 | AC012615.6 | 0.432699492 | 2.82E-24 | postive |
| SNX4 | AC012615.6 | 0.400618513 | 9.76E-21 | postive |
| PHKG2 | AC099522.2 | 0.500730812 | 3.74E-33 | postive |
| EGLN2 | AC099522.2 | 0.428850525 | 7.85E-24 | postive |
| TAZ | AC099522.2 | 0.482595824 | 1.37E-30 | postive |
| HELLS | LINC01139 | 0.428907804 | 7.73E-24 | postive |
| SIRT1 | LINC01139 | 0.451601748 | 1.51E-26 | postive |
| DUOX2 | AC024592.2 | 0.424256759 | 2.62E-23 | postive |
| MUC1 | AL022067.1 | 0.642312268 | 1.26E-59 | postive |
| HELLS | AL513477.2 | 0.478028003 | 5.74E-30 | postive |
| ISCU | AL513477.2 | 0.515188768 | 2.60E-35 | postive |
| STMN1 | AL513477.2 | 0.499240702 | 6.15E-33 | postive |
| NOX1 | AL513477.2 | 0.512152704 | 7.53E-35 | postive |
| PHKG2 | AL513477.2 | 0.420512786 | 6.91E-23 | postive |
| BID | LINC01273 | 0.431459592 | 3.93E-24 | postive |
| MIOX | LINC01273 | 0.501334895 | 3.05E-33 | postive |
| TUBE1 | AC090181.2 | 0.443505513 | 1.48E-25 | postive |
| ELAVL1 | AC090181.2 | 0.402972828 | 5.53E-21 | postive |
| ATF4 | AL139089.1 | 0.452047517 | 1.33E-26 | postive |
| ISCU | AL139089.1 | 0.4182 | 1.25E-22 | postive |
| TUBE1 | AL139089.1 | 0.400883819 | 9.15E-21 | postive |
| HMGB1 | AL139089.1 | 0.456431992 | 3.77E-27 | postive |
| STMN1 | AL139089.1 | 0.432097197 | 3.31E-24 | postive |
| NOX1 | AL139089.1 | 0.424543341 | 2.43E-23 | postive |
| PHKG2 | AL139089.1 | 0.503045092 | 1.71E-33 | postive |
| PEBP1 | AL139089.1 | 0.408717516 | 1.35E-21 | postive |
| TAZ | AL139089.1 | 0.538407248 | 5.33E-39 | postive |
| GPX4 | AC104463.2 | 0.464731359 | 3.29E-28 | postive |
| CISD1 | AC104463.2 | 0.415859758 | 2.27E-22 | postive |
| ISCU | AC104463.2 | 0.410000719 | 9.85E-22 | postive |
| ACSF2 | AC104463.2 | 0.458010104 | 2.39E-27 | postive |
| NOX1 | AC104463.2 | 0.514816553 | 2.96E-35 | postive |
| PHKG2 | AC104463.2 | 0.524806907 | 8.35E-37 | postive |
| MAP1LC3A | AC104463.2 | 0.438081538 | 6.58E-25 | postive |
| EGLN2 | AC104463.2 | 0.490258132 | 1.18E-31 | postive |
| TAZ | AC104463.2 | 0.469841108 | 7.10E-29 | postive |
| ISCU | AC025165.5 | 0.408370056 | 1.47E-21 | postive |
| CAV1 | LINC01322 | 0.461116443 | 9.61E-28 | postive |
| PANX1 | LINC01322 | 0.483789707 | 9.39E-31 | postive |
| DDIT4 | AC039056.2 | 0.452417264 | 1.20E-26 | postive |
| VEGFA | AC039056.2 | 0.416306381 | 2.02E-22 | postive |
| GPX4 | KMT2E-AS1 | 0.452534149 | 1.16E-26 | postive |
| ATF4 | KMT2E-AS1 | 0.406580588 | 2.29E-21 | postive |
| DDIT3 | KMT2E-AS1 | 0.412851717 | 4.84E-22 | postive |
| RPL8 | KMT2E-AS1 | 0.442362242 | 2.03E-25 | postive |
| SLC2A8 | KMT2E-AS1 | 0.463826518 | 4.31E-28 | postive |
| NOX1 | KMT2E-AS1 | 0.413819974 | 3.79E-22 | postive |
| PHKG2 | KMT2E-AS1 | 0.661292405 | 2.65E-64 | postive |
| PEBP1 | KMT2E-AS1 | 0.402356653 | 6.41E-21 | postive |
| ANO6 | KMT2E-AS1 | -0.467902956 | 1.27E-28 | negative |
| EGLN2 | KMT2E-AS1 | 0.567232049 | 5.35E-44 | postive |
| TAZ | KMT2E-AS1 | 0.578890711 | 3.66E-46 | postive |
| LONP1 | KMT2E-AS1 | 0.411908379 | 6.12E-22 | postive |
| ATG4D | WEE2-AS1 | 0.417188457 | 1.62E-22 | postive |
| FANCD2 | AC069224.2 | 0.447853962 | 4.38E-26 | postive |
| HELLS | AC069224.2 | 0.524997571 | 7.79E-37 | postive |
| KLHL24 | AC069224.2 | 0.530963046 | 8.72E-38 | postive |
| TUBE1 | AC069224.2 | 0.403150508 | 5.29E-21 | postive |
| HMGB1 | AC069224.2 | 0.400124897 | 1.10E-20 | postive |
| STMN1 | AC069224.2 | 0.48012699 | 2.98E-30 | postive |
| GABPB1 | AC069224.2 | 0.437934068 | 6.85E-25 | postive |
| GLS2 | AC069224.2 | 0.488605615 | 2.01E-31 | postive |
| SNX4 | AC069224.2 | 0.466061039 | 2.21E-28 | postive |
| PHKG2 | AC106738.1 | 0.499000751 | 6.67E-33 | postive |
| ENPP2 | CARD8-AS1 | 0.433548998 | 2.24E-24 | postive |
| CYBB | CARD8-AS1 | 0.412190256 | 5.71E-22 | postive |
| FLT3 | CARD8-AS1 | 0.481142219 | 2.17E-30 | postive |
| LPCAT3 | AL139035.1 | 0.401527804 | 7.84E-21 | postive |
| PHKG2 | AC009171.2 | 0.412913678 | 4.76E-22 | postive |
| PHKG2 | BX284668.5 | 0.417004087 | 1.69E-22 | postive |
| GPX4 | AC015982.2 | 0.402493562 | 6.21E-21 | postive |
| PHKG2 | AC015982.2 | 0.590372213 | 2.21E-48 | postive |
| ANO6 | AC015982.2 | -0.40730686 | 1.92E-21 | negative |
| EGLN2 | AC015982.2 | 0.540430138 | 2.46E-39 | postive |
| TAZ | AC015982.2 | 0.491661715 | 7.49E-32 | postive |
| LONP1 | AC015982.2 | 0.413397955 | 4.22E-22 | postive |
| RPL8 | AC103706.1 | 0.444416936 | 1.15E-25 | postive |
| SLC2A8 | AC103706.1 | 0.487534594 | 2.84E-31 | postive |
| PHKG2 | AC103706.1 | 0.403538722 | 4.82E-21 | postive |
| EGLN2 | AC103706.1 | 0.546111626 | 2.74E-40 | postive |
| GLS2 | AC009126.1 | 0.479356825 | 3.79E-30 | postive |
| TUBE1 | ALOX12-AS1 | 0.437849815 | 7.01E-25 | postive |
| NNMT | AC010457.1 | 0.408139969 | 1.56E-21 | postive |
| PRKAA2 | AC010457.1 | 0.43241133 | 3.04E-24 | postive |
| SQSTM1 | AC009779.3 | 0.445596713 | 8.25E-26 | postive |
| HELLS | AC009779.3 | 0.402893643 | 5.63E-21 | postive |
| FADS2 | AC009779.3 | 0.411771405 | 6.34E-22 | postive |
| ISCU | AC009779.3 | 0.5515353 | 3.24E-41 | postive |
| AIFM2 | AC009779.3 | 0.437958785 | 6.80E-25 | postive |
| GLS2 | AC009779.3 | 0.416480762 | 1.94E-22 | postive |
| GABARAPL1 | AC009779.3 | 0.418797064 | 1.07E-22 | postive |
| SLC7A11 | AC026785.3 | 0.400920054 | 9.07E-21 | postive |
| FTH1 | AC026785.3 | 0.401823183 | 7.30E-21 | postive |
| TXNRD1 | AC026785.3 | 0.443174853 | 1.62E-25 | postive |
| PML | AC083862.1 | 0.493258448 | 4.44E-32 | postive |
| CYBB | AC083862.1 | 0.426925426 | 1.30E-23 | postive |
| ISCU | AC005393.1 | 0.403513328 | 4.85E-21 | postive |
| PLIN4 | AC005393.1 | 0.453235366 | 9.49E-27 | postive |
| HELLS | CAPN10-DT | 0.437078979 | 8.64E-25 | postive |
| ISCU | CAPN10-DT | 0.448699883 | 3.45E-26 | postive |
| TUBE1 | CAPN10-DT | 0.530307993 | 1.11E-37 | postive |
| GABPB1 | CAPN10-DT | 0.412562007 | 5.20E-22 | postive |
| GLS2 | CAPN10-DT | 0.420249855 | 7.39E-23 | postive |
| PHKG2 | CAPN10-DT | 0.428843936 | 7.86E-24 | postive |
| MYB | CAPN10-DT | 0.471098562 | 4.84E-29 | postive |
| MYB | PCAT7 | 0.405875803 | 2.72E-21 | postive |
| FANCD2 | AC104794.3 | 0.525841412 | 5.73E-37 | postive |
| TP53 | AC104794.3 | 0.500490906 | 4.05E-33 | postive |
| HELLS | AC104794.3 | 0.629038131 | 1.50E-56 | postive |
| FADS2 | AC104794.3 | 0.476136291 | 1.03E-29 | postive |
| ISCU | AC104794.3 | 0.556877402 | 3.80E-42 | postive |
| AGPAT3 | AC104794.3 | 0.428636168 | 8.31E-24 | postive |
| ELAVL1 | AC104794.3 | 0.413448258 | 4.16E-22 | postive |
| SP1 | AC104794.3 | 0.433038299 | 2.57E-24 | postive |
| STMN1 | AC104794.3 | 0.551479139 | 3.31E-41 | postive |
| NOX1 | AC104794.3 | 0.406619812 | 2.27E-21 | postive |
| GLS2 | AC104794.3 | 0.558737668 | 1.79E-42 | postive |
| ATG4D | AC104794.3 | 0.409794416 | 1.04E-21 | postive |
| CDKN2A | AC104794.3 | 0.468043907 | 1.22E-28 | postive |
| MYB | AC104794.3 | 0.488302153 | 2.22E-31 | postive |
| MAPK8 | AC104794.3 | 0.500314802 | 4.30E-33 | postive |
| LPIN1 | AC104794.3 | 0.408077284 | 1.59E-21 | postive |
| YY1AP1 | AC104794.3 | 0.483077526 | 1.18E-30 | postive |
| CARS1 | LINC00839 | 0.455722902 | 4.63E-27 | postive |
| GPX4 | MAPKAPK5-AS1 | 0.540314343 | 2.57E-39 | postive |
| CISD1 | MAPKAPK5-AS1 | 0.465552607 | 2.58E-28 | postive |
| ATF4 | MAPKAPK5-AS1 | 0.573166549 | 4.34E-45 | postive |
| FH | MAPKAPK5-AS1 | 0.441051559 | 2.91E-25 | postive |
| CISD2 | MAPKAPK5-AS1 | 0.425661171 | 1.82E-23 | postive |
| ISCU | MAPKAPK5-AS1 | 0.486305165 | 4.22E-31 | postive |
| PRDX6 | MAPKAPK5-AS1 | 0.474119177 | 1.92E-29 | postive |
| AIFM2 | MAPKAPK5-AS1 | 0.437038775 | 8.74E-25 | postive |
| SELENOS | MAPKAPK5-AS1 | 0.406728995 | 2.21E-21 | postive |
| ASNS | MAPKAPK5-AS1 | 0.430300801 | 5.34E-24 | postive |
| DDIT3 | MAPKAPK5-AS1 | 0.485059916 | 6.27E-31 | postive |
| ZNF419 | MAPKAPK5-AS1 | 0.489211612 | 1.66E-31 | postive |
| TUBE1 | MAPKAPK5-AS1 | 0.428592645 | 8.40E-24 | postive |
| RPL8 | MAPKAPK5-AS1 | 0.459567234 | 1.51E-27 | postive |
| SLC2A8 | MAPKAPK5-AS1 | 0.441986687 | 2.25E-25 | postive |
| STMN1 | MAPKAPK5-AS1 | 0.4418724 | 2.32E-25 | postive |
| NOX1 | MAPKAPK5-AS1 | 0.458835438 | 1.88E-27 | postive |
| DUOX1 | MAPKAPK5-AS1 | -0.419873114 | 8.14E-23 | negative |
| SLC1A5 | MAPKAPK5-AS1 | 0.419884054 | 8.12E-23 | postive |
| PHKG2 | MAPKAPK5-AS1 | 0.62744046 | 3.45E-56 | postive |
| MAP1LC3A | MAPKAPK5-AS1 | 0.452289422 | 1.24E-26 | postive |
| BID | MAPKAPK5-AS1 | 0.499384056 | 5.87E-33 | postive |
| PEBP1 | MAPKAPK5-AS1 | 0.558971044 | 1.63E-42 | postive |
| EGLN2 | MAPKAPK5-AS1 | 0.546068216 | 2.79E-40 | postive |
| TAZ | MAPKAPK5-AS1 | 0.582765466 | 6.68E-47 | postive |
| PANX1 | MAPKAPK5-AS1 | -0.40771555 | 1.73E-21 | negative |
| BACH1 | MAPKAPK5-AS1 | -0.422603173 | 4.03E-23 | negative |
| LONP1 | MAPKAPK5-AS1 | 0.446790809 | 5.91E-26 | postive |
| PHKG2 | AGBL5-IT1 | 0.523805891 | 1.20E-36 | postive |
| TAZ | AGBL5-IT1 | 0.430848678 | 4.62E-24 | postive |
| SETD1B | AL132989.1 | 0.415060315 | 2.77E-22 | postive |
| SP1 | AL132989.1 | 0.419354352 | 9.30E-23 | postive |
| ACVR1B | AL132989.1 | 0.407700788 | 1.74E-21 | postive |
| NNMT | AC009509.5 | 0.412394379 | 5.42E-22 | postive |
| FANCD2 | PSMA3-AS1 | 0.460561548 | 1.13E-27 | postive |
| HELLS | PSMA3-AS1 | 0.53767896 | 7.02E-39 | postive |
| ISCU | PSMA3-AS1 | 0.413801336 | 3.81E-22 | postive |
| TUBE1 | PSMA3-AS1 | 0.438552132 | 5.78E-25 | postive |
| HMGB1 | PSMA3-AS1 | 0.430622626 | 4.91E-24 | postive |
| STMN1 | PSMA3-AS1 | 0.457416948 | 2.84E-27 | postive |
| GABPB1 | PSMA3-AS1 | 0.440039254 | 3.85E-25 | postive |
| PHKG2 | MAP3K5-AS1 | 0.408581272 | 1.40E-21 | postive |
| EGLN2 | MAP3K5-AS1 | 0.475664756 | 1.19E-29 | postive |
| TF | SSTR5-AS1 | 0.411739219 | 6.39E-22 | postive |
| TFR2 | ELFN1-AS1 | 0.404208032 | 4.09E-21 | postive |
| MIOX | ELFN1-AS1 | 0.404094502 | 4.21E-21 | postive |
| HSPB1 | AP001816.1 | 0.406833904 | 2.15E-21 | postive |
| CHMP6 | AP001816.1 | 0.470674218 | 5.51E-29 | postive |
| SETD1B | AP001816.1 | -0.407178824 | 1.98E-21 | negative |
| RPL8 | AP001816.1 | 0.470905414 | 5.14E-29 | postive |
| IREB2 | AP001816.1 | -0.431592823 | 3.79E-24 | negative |
| SP1 | AP001816.1 | -0.439572408 | 4.37E-25 | negative |
| HRAS | AP001816.1 | 0.423975861 | 2.82E-23 | postive |
| PHKG2 | AP001816.1 | 0.45123451 | 1.68E-26 | postive |
| EGLN2 | AP001816.1 | 0.586863231 | 1.08E-47 | postive |
| EGLN2 | AC026803.1 | 0.485857459 | 4.86E-31 | postive |
| PHKG2 | AC027307.2 | 0.518297951 | 8.66E-36 | postive |
| EGLN2 | AC027307.2 | 0.405050923 | 3.33E-21 | postive |
| TAZ | AC027307.2 | 0.464783354 | 3.24E-28 | postive |
| LONP1 | AC027307.2 | 0.439621162 | 4.32E-25 | postive |
| TUBE1 | AC007663.4 | 0.409454672 | 1.13E-21 | postive |
| PLIN4 | SH3RF3-AS1 | 0.409775159 | 1.04E-21 | postive |
| WIPI1 | SH3RF3-AS1 | 0.415224364 | 2.66E-22 | postive |
| ZEB1 | SH3RF3-AS1 | 0.655984434 | 5.83E-63 | postive |
| PRKAA2 | SH3RF3-AS1 | 0.534415183 | 2.40E-38 | postive |
| TLR4 | SH3RF3-AS1 | 0.419860806 | 8.17E-23 | postive |
| NOX1 | AC002550.2 | 0.429514391 | 6.58E-24 | postive |
| PHKG2 | AC002550.2 | 0.530973809 | 8.68E-38 | postive |
| TAZ | AC002550.2 | 0.44826277 | 3.90E-26 | postive |
| NFE2L2 | LINC01133 | 0.405298025 | 3.14E-21 | postive |
| PSAT1 | LINC01133 | 0.405811091 | 2.77E-21 | postive |
| ELAVL1 | LINC01133 | 0.405875162 | 2.72E-21 | postive |
| PHKG2 | LINC01133 | 0.416896852 | 1.74E-22 | postive |
| HELLS | MCF2L-AS1 | 0.464662147 | 3.36E-28 | postive |
| ISCU | MCF2L-AS1 | 0.414147711 | 3.49E-22 | postive |
| KLHL24 | MCF2L-AS1 | 0.536073964 | 1.29E-38 | postive |
| ELAVL1 | MCF2L-AS1 | 0.442186499 | 2.13E-25 | postive |
| STMN1 | MCF2L-AS1 | 0.428544213 | 8.51E-24 | postive |
| GLS2 | MCF2L-AS1 | 0.513972516 | 3.99E-35 | postive |
| GOT1 | MCF2L-AS1 | 0.427487821 | 1.12E-23 | postive |
| ATG4D | MCF2L-AS1 | 0.434837 | 1.59E-24 | postive |
| LPIN1 | MCF2L-AS1 | 0.429411378 | 6.77E-24 | postive |
| ATG3 | LINC02004 | 0.439206221 | 4.84E-25 | postive |
| DNAJB6 | AC000123.1 | 0.424815007 | 2.27E-23 | postive |
| PHKG2 | AC010883.1 | 0.526554814 | 4.42E-37 | postive |
| MYB | AC010883.1 | 0.408618216 | 1.39E-21 | postive |
| TAZ | AC010883.1 | 0.446530341 | 6.35E-26 | postive |
| DUOX1 | AC083841.1 | 0.474428499 | 1.75E-29 | postive |
| ALOX12B | AC083841.1 | 0.706330289 | 6.81E-77 | postive |
| ALOXE3 | AC083841.1 | 0.52548141 | 6.53E-37 | postive |
| HRAS | AC012065.3 | 0.403008643 | 5.48E-21 | postive |
| STMN1 | AC005696.4 | 0.428060861 | 9.67E-24 | postive |
| DUOX1 | AC115522.1 | 0.515068141 | 2.71E-35 | postive |
| ALOX12B | AC115522.1 | 0.648776216 | 3.50E-61 | postive |
| ALOXE3 | AC115522.1 | 0.544632243 | 4.87E-40 | postive |
| MAPK3 | AC115522.1 | 0.428683804 | 8.20E-24 | postive |
| DNAJB6 | AC115522.1 | 0.418805559 | 1.07E-22 | postive |
| PHKG2 | SNHG10 | 0.500537914 | 3.99E-33 | postive |
| TAZ | SNHG10 | 0.503118795 | 1.67E-33 | postive |
| GPX4 | SNHG6 | 0.513111122 | 5.39E-35 | postive |
| CISD1 | SNHG6 | 0.403363811 | 5.03E-21 | postive |
| ATF4 | SNHG6 | 0.438179677 | 6.40E-25 | postive |
| RPL8 | SNHG6 | 0.640860743 | 2.77E-59 | postive |
| SLC2A8 | SNHG6 | 0.457844984 | 2.50E-27 | postive |
| STMN1 | SNHG6 | 0.517474917 | 1.16E-35 | postive |
| NOX1 | SNHG6 | 0.440837884 | 3.09E-25 | postive |
| PHKG2 | SNHG6 | 0.432263711 | 3.17E-24 | postive |
| PEBP1 | SNHG6 | 0.44669651 | 6.06E-26 | postive |
| EGLN2 | SNHG6 | 0.521216549 | 3.05E-36 | postive |
| TAZ | SNHG6 | 0.450756164 | 1.93E-26 | postive |
| LONP1 | SNHG6 | 0.403902844 | 4.41E-21 | postive |
| SP1 | AL049840.6 | -0.472388074 | 3.27E-29 | negative |
| CS | AL049840.6 | -0.407058328 | 2.04E-21 | negative |
| LPCAT3 | AL049840.6 | -0.441085541 | 2.89E-25 | negative |
| HRAS | AL049840.6 | 0.533191899 | 3.80E-38 | postive |
| NCOA4 | AL049840.6 | -0.406004157 | 2.64E-21 | negative |
| EGLN2 | AL049840.6 | 0.527357292 | 3.29E-37 | postive |
| SIRT1 | AL049840.6 | -0.419162622 | 9.77E-23 | negative |
| FANCD2 | LINC01305 | 0.453710758 | 8.28E-27 | postive |
| HELLS | LINC01305 | 0.496462575 | 1.55E-32 | postive |
| GLS2 | LINC01305 | 0.419014955 | 1.01E-22 | postive |
| MYB | LINC01305 | 0.420641918 | 6.68E-23 | postive |
| HELLS | LINC00342 | 0.407130897 | 2.00E-21 | postive |
| NOX4 | C2orf27A | 0.515810655 | 2.09E-35 | postive |
| ALOX5 | LINC01943 | 0.464603975 | 3.42E-28 | postive |
| CYBB | LINC01943 | 0.48457655 | 7.32E-31 | postive |
| FLT3 | LINC01943 | 0.421071474 | 5.98E-23 | postive |
| IFNG | LINC01943 | 0.574721594 | 2.23E-45 | postive |
| GPX4 | AC016405.3 | 0.634022954 | 1.09E-57 | postive |
| CISD1 | AC016405.3 | 0.605025553 | 2.41E-51 | postive |
| ISCU | AC016405.3 | 0.504576145 | 1.02E-33 | postive |
| CARS1 | AC016405.3 | 0.541559762 | 1.60E-39 | postive |
| MAP1LC3A | AC016405.3 | 0.4887635 | 1.91E-31 | postive |
| AKR1C1 | AL033397.2 | 0.516935017 | 1.40E-35 | postive |
| AKR1C2 | AL033397.2 | 0.538628949 | 4.90E-39 | postive |
| AKR1C3 | AL033397.2 | 0.522034133 | 2.28E-36 | postive |
| GCLC | AL033397.2 | 0.733217778 | 1.28E-85 | postive |
| NFE2L2 | AL033397.2 | 0.452515807 | 1.17E-26 | postive |
| TXNRD1 | AL033397.2 | 0.553871886 | 1.28E-41 | postive |
| SRXN1 | AL033397.2 | 0.437518469 | 7.67E-25 | postive |
| GPX2 | AL033397.2 | 0.542725226 | 1.02E-39 | postive |
| MAFG | AL033397.2 | 0.510042564 | 1.57E-34 | postive |
| G6PD | AL033397.2 | 0.405139652 | 3.26E-21 | postive |
| PGD | AL033397.2 | 0.407002868 | 2.07E-21 | postive |
| ABCC1 | AL033397.2 | 0.495000784 | 2.51E-32 | postive |
| SIRT1 | AC004492.1 | 0.428277597 | 9.13E-24 | postive |
| GPX4 | TRIM52-AS1 | 0.453047136 | 1.00E-26 | postive |
| CHMP6 | TRIM52-AS1 | 0.410704312 | 8.27E-22 | postive |
| PHKG2 | TRIM52-AS1 | 0.489380425 | 1.57E-31 | postive |
| MAP1LC3A | TRIM52-AS1 | 0.410675892 | 8.33E-22 | postive |
| EGLN2 | TRIM52-AS1 | 0.427604019 | 1.09E-23 | postive |
| ELAVL1 | AC005034.4 | 0.452222087 | 1.27E-26 | postive |
| PHKG2 | AC097641.2 | 0.446965696 | 5.62E-26 | postive |
| TAZ | AC097641.2 | 0.428850846 | 7.85E-24 | postive |
| GPX4 | AC244090.1 | 0.560738876 | 7.89E-43 | postive |
| HSPB1 | AC244090.1 | 0.445913516 | 7.55E-26 | postive |
| CISD1 | AC244090.1 | 0.413371241 | 4.24E-22 | postive |
| RPL8 | AC244090.1 | 0.45623506 | 4.00E-27 | postive |
| SLC2A8 | AC244090.1 | 0.415386182 | 2.55E-22 | postive |
| NOX1 | AC244090.1 | 0.41688563 | 1.75E-22 | postive |
| PHKG2 | AC244090.1 | 0.60509585 | 2.33E-51 | postive |
| EGLN2 | AC244090.1 | 0.743885285 | 2.21E-89 | postive |
| TAZ | AC244090.1 | 0.559205507 | 1.48E-42 | postive |
| LONP1 | AC244090.1 | 0.411327295 | 7.08E-22 | postive |
| TUBE1 | AL135999.1 | 0.496418685 | 1.57E-32 | postive |
| TAZ | AL135999.1 | 0.426163058 | 1.59E-23 | postive |
| FANCD2 | AL161891.1 | 0.512495352 | 6.68E-35 | postive |
| HELLS | AL161891.1 | 0.544972453 | 4.27E-40 | postive |
| FADS2 | AL161891.1 | 0.402512022 | 6.18E-21 | postive |
| IREB2 | AL161891.1 | 0.439353014 | 4.65E-25 | postive |
| HMGB1 | AL161891.1 | 0.464821553 | 3.21E-28 | postive |
| ELAVL1 | AL161891.1 | 0.47424234 | 1.85E-29 | postive |
| SP1 | AL161891.1 | 0.486888418 | 3.50E-31 | postive |
| RRM2 | AL161891.1 | 0.423535713 | 3.16E-23 | postive |
| GLS2 | AL161891.1 | 0.440447845 | 3.44E-25 | postive |
| SNX4 | AL161891.1 | 0.423644129 | 3.07E-23 | postive |
| SIRT1 | AL161891.1 | 0.413110102 | 4.53E-22 | postive |
| GPX4 | AL591895.1 | 0.419271276 | 9.50E-23 | postive |
| ATF4 | AL591895.1 | 0.400670698 | 9.64E-21 | postive |
| ISCU | AL591895.1 | 0.522508468 | 1.92E-36 | postive |
| STMN1 | AL591895.1 | 0.450002263 | 2.39E-26 | postive |
| NOX1 | AL591895.1 | 0.499046537 | 6.57E-33 | postive |
| PHKG2 | AL591895.1 | 0.573310946 | 4.08E-45 | postive |
| MYB | AL591895.1 | 0.455266607 | 5.29E-27 | postive |
| YY1AP1 | AL591895.1 | 0.418158516 | 1.26E-22 | postive |
| TAZ | AL591895.1 | 0.478693093 | 4.67E-30 | postive |
| GPX4 | GIHCG | 0.545386237 | 3.64E-40 | postive |
| PHKG2 | GIHCG | 0.477621 | 6.52E-30 | postive |
| EGLN2 | GIHCG | 0.422157347 | 4.52E-23 | postive |
| GPX4 | AC124016.2 | 0.545585391 | 3.36E-40 | postive |
| CISD1 | AC124016.2 | 0.454468848 | 6.65E-27 | postive |
| ATF4 | AC124016.2 | 0.474388527 | 1.77E-29 | postive |
| CISD2 | AC124016.2 | 0.41361842 | 3.99E-22 | postive |
| ISCU | AC124016.2 | 0.484816747 | 6.78E-31 | postive |
| SLC2A8 | AC124016.2 | 0.415376692 | 2.56E-22 | postive |
| ACSF2 | AC124016.2 | 0.400053347 | 1.12E-20 | postive |
| STMN1 | AC124016.2 | 0.5466512 | 2.22E-40 | postive |
| NOX1 | AC124016.2 | 0.484953321 | 6.49E-31 | postive |
| PHKG2 | AC124016.2 | 0.565262071 | 1.22E-43 | postive |
| MAP1LC3A | AC124016.2 | 0.415004707 | 2.81E-22 | postive |
| PEBP1 | AC124016.2 | 0.422296246 | 4.36E-23 | postive |
| SOCS1 | AC124016.2 | 0.489684496 | 1.42E-31 | postive |
| EGLN2 | AC124016.2 | 0.548998755 | 8.83E-41 | postive |
| TAZ | AC124016.2 | 0.495638209 | 2.03E-32 | postive |
| ZNF419 | AC011468.1 | 0.496431208 | 1.57E-32 | postive |
| PHKG2 | AC011468.1 | 0.48911731 | 1.71E-31 | postive |
| EGLN2 | AC011468.1 | 0.483768603 | 9.46E-31 | postive |
| PIK3CA | AC022364.1 | 0.431187332 | 4.22E-24 | postive |
| LPCAT3 | AC022364.1 | 0.480536715 | 2.62E-30 | postive |
| DDIT3 | AC011676.1 | 0.515990001 | 1.96E-35 | postive |
| ELAVL1 | MIR762HG | 0.425584784 | 1.85E-23 | postive |
| STMN1 | MIR762HG | 0.407730743 | 1.73E-21 | postive |
| PHKG2 | MIR762HG | 0.614846327 | 2.03E-53 | postive |
| PEBP1 | MIR762HG | 0.44404954 | 1.27E-25 | postive |
| TAZ | MIR762HG | 0.538826663 | 4.54E-39 | postive |
| MYB | AP001628.1 | 0.452267316 | 1.25E-26 | postive |
| HELLS | JPX | 0.410133333 | 9.53E-22 | postive |
| TUBE1 | JPX | 0.447429284 | 4.94E-26 | postive |
| HMGB1 | JPX | 0.480753097 | 2.45E-30 | postive |
| ELAVL1 | JPX | 0.414557701 | 3.15E-22 | postive |
| STMN1 | JPX | 0.409603276 | 1.09E-21 | postive |
| NOX1 | JPX | 0.493889922 | 3.61E-32 | postive |
| TAZ | JPX | 0.490559013 | 1.07E-31 | postive |
| TXNRD1 | AC007365.1 | 0.515664152 | 2.20E-35 | postive |
| SRXN1 | AC007365.1 | 0.432495062 | 2.98E-24 | postive |
| GPX2 | AC007365.1 | 0.430800566 | 4.68E-24 | postive |
| MAFG | AC007365.1 | 0.427110061 | 1.24E-23 | postive |
| G6PD | AC007365.1 | 0.415296785 | 2.61E-22 | postive |
| ABCC1 | AC007365.1 | 0.425665372 | 1.81E-23 | postive |
| IDH1 | AC007365.1 | 0.414760589 | 2.99E-22 | postive |
| TUBE1 | AC006435.2 | 0.48220146 | 1.55E-30 | postive |
| PSAT1 | TMEM44-AS1 | 0.416862499 | 1.76E-22 | postive |
| KLHL24 | TMEM44-AS1 | 0.446577341 | 6.27E-26 | postive |
| TUBE1 | TMEM44-AS1 | 0.414096336 | 3.54E-22 | postive |
| TAZ | TMEM44-AS1 | 0.400914335 | 9.09E-21 | postive |
| PHKG2 | CRIM1-DT | 0.413209963 | 4.42E-22 | postive |
| FANCD2 | MUC20-OT1 | 0.432608622 | 2.89E-24 | postive |
| HELLS | MUC20-OT1 | 0.469889213 | 6.99E-29 | postive |
| NOS2 | MUC20-OT1 | 0.481363906 | 2.02E-30 | postive |
| KLHL24 | MUC20-OT1 | 0.446211365 | 6.95E-26 | postive |
| TFRC | MUC20-OT1 | 0.409004639 | 1.26E-21 | postive |
| GLS2 | MUC20-OT1 | 0.441144305 | 2.84E-25 | postive |
| ATG3 | MUC20-OT1 | 0.480023283 | 3.08E-30 | postive |
| ATG4D | MUC20-OT1 | 0.419299562 | 9.43E-23 | postive |
| SNX4 | MUC20-OT1 | 0.447561827 | 4.76E-26 | postive |
| NNMT | AC015878.1 | 0.474795027 | 1.56E-29 | postive |
| PLIN4 | AC015878.1 | 0.786640536 | 1.42E-106 | postive |
| PRKAA2 | AC015878.1 | 0.794894477 | 2.36E-110 | postive |
| AIFM2 | DLGAP1-AS1 | 0.462417883 | 6.54E-28 | postive |
| TUBE1 | DLGAP1-AS1 | 0.429300301 | 6.97E-24 | postive |
| CISD1 | AC008610.1 | 0.427920678 | 1.00E-23 | postive |
| RPL8 | AC008610.1 | 0.441046176 | 2.92E-25 | postive |
| STMN1 | AC008610.1 | 0.422011835 | 4.69E-23 | postive |
| NOX1 | AC008610.1 | 0.421555193 | 5.28E-23 | postive |
| PHKG2 | AC008610.1 | 0.510066815 | 1.55E-34 | postive |
| EGLN2 | AC008610.1 | 0.487497167 | 2.88E-31 | postive |
| TAZ | AC008610.1 | 0.488637787 | 1.99E-31 | postive |
| HIC1 | AC009093.2 | 0.5289189 | 1.86E-37 | postive |
| NOX4 | AC009093.2 | 0.495789409 | 1.93E-32 | postive |
| NFE2L2 | AC079305.2 | 0.543575399 | 7.34E-40 | postive |
| HELLS | AC245884.8 | 0.402383829 | 6.37E-21 | postive |
| ISCU | AC245884.8 | 0.411250654 | 7.22E-22 | postive |
| STMN1 | AC245884.8 | 0.430694323 | 4.81E-24 | postive |
| NOX1 | AC245884.8 | 0.400882163 | 9.16E-21 | postive |
| PHKG2 | AC245884.8 | 0.506266415 | 5.74E-34 | postive |
| MYB | AC245884.8 | 0.460514969 | 1.15E-27 | postive |
| EGLN2 | AC245884.8 | 0.404661729 | 3.66E-21 | postive |
| TAZ | AC245884.8 | 0.504819613 | 9.39E-34 | postive |
| MT3 | AC087623.1 | 0.404926532 | 3.43E-21 | postive |
| TUBE1 | AC087623.1 | 0.413020787 | 4.63E-22 | postive |
| CDO1 | AC087623.1 | 0.495883315 | 1.88E-32 | postive |
| MYB | AC087623.1 | 0.46884417 | 9.59E-29 | postive |
| FANCD2 | AL035587.1 | 0.471008798 | 4.98E-29 | postive |
| TP53 | AL035587.1 | 0.459069459 | 1.75E-27 | postive |
| HELLS | AL035587.1 | 0.520723651 | 3.64E-36 | postive |
| ISCU | AL035587.1 | 0.406199416 | 2.52E-21 | postive |
| KLHL24 | AL035587.1 | 0.421733464 | 5.04E-23 | postive |
| ELAVL1 | AL035587.1 | 0.429014925 | 7.52E-24 | postive |
| SP1 | AL035587.1 | 0.533304254 | 3.65E-38 | postive |
| GLS2 | AL035587.1 | 0.510616652 | 1.28E-34 | postive |
| MYB | AL035587.1 | 0.419109994 | 9.90E-23 | postive |
| ACVR1B | AL035587.1 | 0.419510911 | 8.94E-23 | postive |
| ATM | AL035587.1 | 0.420707578 | 6.57E-23 | postive |
| YY1AP1 | AL035587.1 | 0.41101226 | 7.66E-22 | postive |
| ZNF419 | AC020915.3 | 0.412807113 | 4.89E-22 | postive |
| NFE2L2 | AC015871.3 | 0.401875628 | 7.21E-21 | postive |
| KLHL24 | AC015871.3 | 0.451166225 | 1.71E-26 | postive |
| TUBE1 | AC015871.3 | 0.517006858 | 1.37E-35 | postive |
| GABPB1 | AC015871.3 | 0.500935335 | 3.49E-33 | postive |
| YY1AP1 | AC015871.3 | 0.430863724 | 4.60E-24 | postive |
| CHMP6 | AC087741.2 | 0.487943083 | 2.49E-31 | postive |
| PHKG2 | AC087741.2 | 0.510123669 | 1.52E-34 | postive |
| EGLN2 | AC087741.2 | 0.469562614 | 7.72E-29 | postive |
| TAZ | AC087741.2 | 0.461245036 | 9.25E-28 | postive |
| ENPP2 | LINC01857 | 0.417035666 | 1.68E-22 | postive |
| ALOX5 | LINC01857 | 0.479021165 | 4.21E-30 | postive |
| CYBB | LINC01857 | 0.423467614 | 3.22E-23 | postive |
| FLT3 | LINC01857 | 0.545421423 | 3.59E-40 | postive |
| ISCU | MRPL20-AS1 | 0.430390262 | 5.22E-24 | postive |
| PHKG2 | MRPL20-AS1 | 0.467559288 | 1.41E-28 | postive |
| TAZ | MRPL20-AS1 | 0.40546056 | 3.01E-21 | postive |
| PHKG2 | NALT1 | 0.416834533 | 1.77E-22 | postive |
| PIK3CA | AC092953.2 | 0.422609256 | 4.02E-23 | postive |
| SETD1B | MID1IP1-AS1 | 0.404114289 | 4.19E-21 | postive |
| IREB2 | MID1IP1-AS1 | 0.407710129 | 1.74E-21 | postive |
| SP1 | MID1IP1-AS1 | 0.408583005 | 1.40E-21 | postive |
| PIK3CA | MID1IP1-AS1 | 0.42798952 | 9.85E-24 | postive |
| PHKG2 | SNAI3-AS1 | 0.435497915 | 1.33E-24 | postive |
| TMBIM4 | AC026124.2 | 0.42019942 | 7.49E-23 | postive |
| ATF4 | AC005387.1 | 0.423276611 | 3.38E-23 | postive |
| PHKG2 | AC005387.1 | 0.517975075 | 9.71E-36 | postive |
| TAZ | AC005387.1 | 0.430854353 | 4.61E-24 | postive |
| GPX4 | AC097468.3 | 0.478544449 | 4.89E-30 | postive |
| CISD1 | AC097468.3 | 0.472375997 | 3.28E-29 | postive |
| ISCU | AC097468.3 | 0.413563062 | 4.04E-22 | postive |
| SLC2A8 | AC097468.3 | 0.400578685 | 9.85E-21 | postive |
| NOX1 | AC097468.3 | 0.459234358 | 1.67E-27 | postive |
| PHKG2 | AC097468.3 | 0.502403451 | 2.13E-33 | postive |
| EGLN2 | AC097468.3 | 0.464768626 | 3.26E-28 | postive |
| HELLS | AL390195.3 | 0.444678083 | 1.07E-25 | postive |
| ELAVL1 | AL390195.3 | 0.409364653 | 1.15E-21 | postive |
| MAPK8 | AL390195.3 | 0.403825998 | 4.49E-21 | postive |
| PHKG2 | RAB30-DT | 0.464687955 | 3.34E-28 | postive |
| ISCU | LINC01569 | 0.414073242 | 3.56E-22 | postive |
| PHKG2 | LINC01569 | 0.467285934 | 1.53E-28 | postive |
| MAP1LC3A | LINC01569 | 0.456778217 | 3.41E-27 | postive |
| TAZ | LINC01569 | 0.448253694 | 3.91E-26 | postive |
| SOCS1 | AC083964.1 | 0.474516058 | 1.70E-29 | postive |
| GPX4 | TMEM161B-AS1 | 0.475133508 | 1.41E-29 | postive |
| CISD1 | TMEM161B-AS1 | 0.480990088 | 2.27E-30 | postive |
| ATF4 | TMEM161B-AS1 | 0.407826138 | 1.69E-21 | postive |
| HELLS | TMEM161B-AS1 | 0.417259359 | 1.59E-22 | postive |
| ISCU | TMEM161B-AS1 | 0.581731175 | 1.05E-46 | postive |
| STMN1 | TMEM161B-AS1 | 0.527771963 | 2.83E-37 | postive |
| NOX1 | TMEM161B-AS1 | 0.453386977 | 9.08E-27 | postive |
| GOT1 | TMEM161B-AS1 | 0.456484675 | 3.72E-27 | postive |
| PHKG2 | TMEM161B-AS1 | 0.429408507 | 6.77E-24 | postive |
| ATG4D | TMEM161B-AS1 | 0.43818641 | 6.39E-25 | postive |
| MAP1LC3A | TMEM161B-AS1 | 0.405827766 | 2.76E-21 | postive |
| SOCS1 | TMEM161B-AS1 | 0.406625176 | 2.27E-21 | postive |
| MYB | TMEM161B-AS1 | 0.416071523 | 2.15E-22 | postive |
| TUBE1 | AL031714.1 | 0.420804402 | 6.41E-23 | postive |
| TUBE1 | LINC00667 | 0.420553449 | 6.84E-23 | postive |
| EGLN2 | LINC02062 | 0.470226664 | 6.31E-29 | postive |
| WIPI2 | AC104041.1 | 0.402857816 | 5.68E-21 | postive |
| PHKG2 | VASH1-AS1 | 0.495756203 | 1.96E-32 | postive |
| EGLN2 | VASH1-AS1 | 0.522371467 | 2.02E-36 | postive |
| TAZ | VASH1-AS1 | 0.406248273 | 2.49E-21 | postive |
| GPX4 | AC092354.2 | 0.528609528 | 2.08E-37 | postive |
| CISD1 | AC092354.2 | 0.492548665 | 5.61E-32 | postive |
| ISCU | AC092354.2 | 0.573564049 | 3.66E-45 | postive |
| STMN1 | AC092354.2 | 0.44865841 | 3.49E-26 | postive |
| NOX1 | AC092354.2 | 0.476957979 | 8.01E-30 | postive |
| MAP1LC3A | AC092354.2 | 0.423969244 | 2.83E-23 | postive |
| SOCS1 | AC092354.2 | 0.464220664 | 3.83E-28 | postive |
| AIFM2 | NRSN2-AS1 | 0.424973036 | 2.17E-23 | postive |
| GLS2 | NRSN2-AS1 | 0.403748277 | 4.58E-21 | postive |
| CHMP6 | AL118558.4 | 0.405524432 | 2.97E-21 | postive |
| PHKG2 | AL118558.4 | 0.528367919 | 2.27E-37 | postive |
| EGLN2 | AL118558.4 | 0.436401535 | 1.04E-24 | postive |
| TAZ | AL118558.4 | 0.455967618 | 4.32E-27 | postive |
| MYB | AC009318.4 | 0.45394953 | 7.73E-27 | postive |
| HELLS | AL121929.3 | 0.413971968 | 3.65E-22 | postive |
| ISCU | AL121929.3 | 0.423108106 | 3.53E-23 | postive |
| STMN1 | AL121929.3 | 0.51420299 | 3.68E-35 | postive |
| NOX1 | AL121929.3 | 0.43536028 | 1.38E-24 | postive |
| SOCS1 | AL121929.3 | 0.438669487 | 5.60E-25 | postive |
| GPX4 | AC142472.1 | 0.41742538 | 1.52E-22 | postive |
| PHKG2 | AC142472.1 | 0.630381892 | 7.45E-57 | postive |
| MAP1LC3A | AC142472.1 | 0.43978661 | 4.13E-25 | postive |
| ANO6 | AC142472.1 | -0.414960391 | 2.84E-22 | negative |
| EGLN2 | AC142472.1 | 0.494420795 | 3.04E-32 | postive |
| TAZ | AC142472.1 | 0.483585416 | 1.00E-30 | postive |
| GABARAPL2 | AC012184.3 | 0.416348087 | 2.00E-22 | postive |
| HELLS | AC008735.2 | 0.428619168 | 8.35E-24 | postive |
| TUBE1 | AC008735.2 | 0.46250956 | 6.37E-28 | postive |
| GPX4 | PITPNA-AS1 | 0.401514966 | 7.86E-21 | postive |
| YWHAE | PITPNA-AS1 | 0.583124217 | 5.70E-47 | postive |
| PGD | AC016888.1 | 0.532097856 | 5.72E-38 | postive |
| KEAP1 | AC016888.1 | 0.444875865 | 1.01E-25 | postive |
| IDH1 | AC016888.1 | 0.492980479 | 4.87E-32 | postive |
| HELLS | AL450384.2 | 0.419215673 | 9.64E-23 | postive |
| STMN1 | AL450384.2 | 0.513860984 | 4.15E-35 | postive |
| NOX1 | AL450384.2 | 0.451538267 | 1.54E-26 | postive |
| PHKG2 | AL450384.2 | 0.433400822 | 2.33E-24 | postive |
| TAZ | AL450384.2 | 0.438527604 | 5.82E-25 | postive |
| HRAS | AC002401.4 | 0.460037357 | 1.32E-27 | postive |
| ISCU | AL354760.1 | 0.455898654 | 4.40E-27 | postive |
| NOX1 | AL354760.1 | 0.422595826 | 4.04E-23 | postive |
| MAP1LC3A | AL354760.1 | 0.430115779 | 5.61E-24 | postive |
| SOCS1 | AL354760.1 | 0.497809164 | 9.91E-33 | postive |
| MT3 | LINC02178 | 0.448083844 | 4.10E-26 | postive |
| CDO1 | LINC02178 | 0.479364559 | 3.79E-30 | postive |
| ELAVL1 | AC016065.1 | 0.43351278 | 2.27E-24 | postive |
| STMN1 | AC016065.1 | 0.417721653 | 1.41E-22 | postive |
| FANCD2 | AC008115.3 | 0.433961548 | 2.01E-24 | postive |
| HELLS | AC008115.3 | 0.539758407 | 3.18E-39 | postive |
| TUBE1 | AC008115.3 | 0.429742551 | 6.20E-24 | postive |
| ELAVL1 | AC008115.3 | 0.430593066 | 4.94E-24 | postive |
| SP1 | AC008115.3 | 0.445900252 | 7.58E-26 | postive |
| GLS2 | AC008115.3 | 0.477538859 | 6.69E-30 | postive |
| MYB | AC008115.3 | 0.471980714 | 3.70E-29 | postive |
| LPIN1 | AC008115.3 | 0.41356633 | 4.04E-22 | postive |
| ATM | AC008115.3 | 0.42749467 | 1.12E-23 | postive |
| ATF4 | AC009065.9 | 0.477252203 | 7.31E-30 | postive |
| FH | AC009065.9 | 0.409432217 | 1.13E-21 | postive |
| ISCU | AC009065.9 | 0.506303304 | 5.67E-34 | postive |
| SLC2A8 | AC009065.9 | 0.413189841 | 4.44E-22 | postive |
| STMN1 | AC009065.9 | 0.435181257 | 1.45E-24 | postive |
| NOX1 | AC009065.9 | 0.474470883 | 1.73E-29 | postive |
| PHKG2 | AC009065.9 | 0.652531643 | 4.20E-62 | postive |
| ATG4D | AC009065.9 | 0.448902998 | 3.26E-26 | postive |
| ANO6 | AC009065.9 | -0.429068156 | 7.41E-24 | negative |
| EGLN2 | AC009065.9 | 0.422616597 | 4.01E-23 | postive |
| TAZ | AC009065.9 | 0.530006011 | 1.24E-37 | postive |
| TUBE1 | AL121906.2 | 0.403080682 | 5.38E-21 | postive |
| PHKG2 | AL121906.2 | 0.421493798 | 5.37E-23 | postive |
| TAZ | AL121906.2 | 0.412689709 | 5.04E-22 | postive |
| GPX4 | AL157394.1 | 0.424546054 | 2.43E-23 | postive |
| CISD1 | AL157394.1 | 0.569412773 | 2.14E-44 | postive |
| HELLS | AL157394.1 | 0.425900922 | 1.71E-23 | postive |
| ISCU | AL157394.1 | 0.650486373 | 1.34E-61 | postive |
| GOT1 | AL157394.1 | 0.419417655 | 9.15E-23 | postive |
| ATG7 | AL157394.1 | 0.406141459 | 2.55E-21 | postive |
| SOCS1 | AL157394.1 | 0.409603072 | 1.09E-21 | postive |
| MAPK8 | AL157394.1 | 0.445724302 | 7.96E-26 | postive |
| CISD1 | Z97200.1 | 0.498193871 | 8.72E-33 | postive |
| ISCU | Z97200.1 | 0.408737901 | 1.35E-21 | postive |
| MAP1LC3A | Z97200.1 | 0.43758036 | 7.54E-25 | postive |
| TUBE1 | AC002553.1 | 0.457607627 | 2.68E-27 | postive |
| GPX4 | URB1-AS1 | 0.486337769 | 4.17E-31 | postive |
| PHKG2 | URB1-AS1 | 0.539034125 | 4.20E-39 | postive |
| EGLN2 | URB1-AS1 | 0.433361364 | 2.36E-24 | postive |
| TAZ | URB1-AS1 | 0.46602856 | 2.24E-28 | postive |
| LONP1 | URB1-AS1 | 0.410442603 | 8.82E-22 | postive |
| GPX4 | GAS5-AS1 | 0.440344706 | 3.54E-25 | postive |
| FANCD2 | GAS5-AS1 | 0.43439617 | 1.79E-24 | postive |
| HELLS | GAS5-AS1 | 0.550620476 | 4.66E-41 | postive |
| ISCU | GAS5-AS1 | 0.549562345 | 7.07E-41 | postive |
| ELAVL1 | GAS5-AS1 | 0.452549587 | 1.15E-26 | postive |
| STMN1 | GAS5-AS1 | 0.606081302 | 1.45E-51 | postive |
| NOX1 | GAS5-AS1 | 0.582689645 | 6.91E-47 | postive |
| GLS2 | GAS5-AS1 | 0.449335465 | 2.88E-26 | postive |
| KEAP1 | GAS5-AS1 | 0.400167201 | 1.09E-20 | postive |
| ATG4D | GAS5-AS1 | 0.480660029 | 2.52E-30 | postive |
| CDKN2A | GAS5-AS1 | 0.50222912 | 2.26E-33 | postive |
| YY1AP1 | GAS5-AS1 | 0.41692996 | 1.73E-22 | postive |
| SCP2 | AC008074.2 | 0.402172411 | 6.71E-21 | postive |
| MT3 | ZNF793-AS1 | 0.419090696 | 9.95E-23 | postive |
| ISCU | DM1-AS | 0.407990259 | 1.62E-21 | postive |
| PHKG2 | DM1-AS | 0.448390835 | 3.76E-26 | postive |
| TAZ | DM1-AS | 0.456643418 | 3.55E-27 | postive |
| GPX4 | AC040169.1 | 0.418859814 | 1.06E-22 | postive |
| CHMP6 | AC040169.1 | 0.422545226 | 4.09E-23 | postive |
| RPL8 | AC040169.1 | 0.458601516 | 2.01E-27 | postive |
| PHKG2 | AC040169.1 | 0.580040494 | 2.22E-46 | postive |
| EGLN2 | AC040169.1 | 0.59596549 | 1.70E-49 | postive |
| TAZ | AC040169.1 | 0.462305516 | 6.76E-28 | postive |
| ACVR1B | CR936218.1 | 0.444812228 | 1.03E-25 | postive |
| HELLS | AL356740.3 | 0.430580705 | 4.96E-24 | postive |
| ISCU | AL356740.3 | 0.414058466 | 3.57E-22 | postive |
| ATG4D | AL356740.3 | 0.401191984 | 8.50E-21 | postive |
| MYB | AL356740.3 | 0.476552483 | 9.08E-30 | postive |
| HELLS | AC145343.1 | 0.429249544 | 7.06E-24 | postive |
| STMN1 | AC145343.1 | 0.50029478 | 4.33E-33 | postive |
| NOX1 | AC145343.1 | 0.42266184 | 3.97E-23 | postive |
| TAZ | AC145343.1 | 0.405298865 | 3.14E-21 | postive |
| ZFP69B | HCG18 | 0.472706598 | 2.97E-29 | postive |
| RIPK1 | HCG18 | 0.418146618 | 1.27E-22 | postive |
| ATM | HCG18 | 0.403502912 | 4.86E-21 | postive |
| SETD1B | AC096733.3 | 0.411150682 | 7.40E-22 | postive |
| SP1 | AC096733.3 | 0.421840523 | 4.91E-23 | postive |
| TUBE1 | AC104564.3 | 0.486998574 | 3.38E-31 | postive |
| PHKG2 | AC060766.4 | 0.401375576 | 8.13E-21 | postive |
| PLIN4 | MYHAS | 0.814368469 | 5.27E-120 | postive |
| ZEB1 | MYHAS | 0.408375098 | 1.47E-21 | postive |
| PRKAA2 | MYHAS | 0.760411554 | 1.34E-95 | postive |
| SLC7A11 | AL136018.1 | 0.416467223 | 1.94E-22 | postive |
| TXNRD1 | AL136018.1 | 0.457808405 | 2.53E-27 | postive |
| SRXN1 | AL136018.1 | 0.415540974 | 2.46E-22 | postive |
| GPX2 | AL136018.1 | 0.418114187 | 1.28E-22 | postive |
| PGD | AL136018.1 | 0.427162435 | 1.23E-23 | postive |
| ABCC1 | AL136018.1 | 0.453026992 | 1.01E-26 | postive |
| ELAVL1 | AP001462.1 | 0.412566581 | 5.19E-22 | postive |
| CEBPG | AC132872.1 | 0.405146573 | 3.26E-21 | postive |
| TAZ | AC132872.1 | 0.450801331 | 1.90E-26 | postive |
| PHKG2 | AC116351.2 | 0.411673906 | 6.49E-22 | postive |
| MAP1LC3A | AC116351.2 | 0.55377429 | 1.33E-41 | postive |
| HELLS | AC096677.1 | 0.444042715 | 1.27E-25 | postive |
| ISCU | AC096677.1 | 0.430479923 | 5.10E-24 | postive |
| TUBE1 | AC096677.1 | 0.460803986 | 1.05E-27 | postive |
| NOX1 | AC096677.1 | 0.422086336 | 4.60E-23 | postive |
| PHKG2 | AC096677.1 | 0.429391893 | 6.80E-24 | postive |
| MYB | AC096677.1 | 0.44649586 | 6.42E-26 | postive |
| YY1AP1 | AC096677.1 | 0.453011612 | 1.01E-26 | postive |
| HELLS | AC005253.1 | 0.416849405 | 1.76E-22 | postive |
| BRD4 | AC005253.1 | 0.470690103 | 5.48E-29 | postive |
| TUBE1 | AC005253.1 | 0.543864362 | 6.56E-40 | postive |
| LPIN1 | AC005253.1 | 0.404447778 | 3.86E-21 | postive |
| MAPK9 | AC008443.1 | 0.5130345 | 5.54E-35 | postive |
| HERPUD1 | AC012181.1 | 0.578968515 | 3.54E-46 | postive |
| SQSTM1 | AC005332.6 | 0.461026094 | 9.87E-28 | postive |
| FANCD2 | AC005332.6 | 0.441205625 | 2.79E-25 | postive |
| TP53 | AC005332.6 | 0.421685716 | 5.11E-23 | postive |
| HELLS | AC005332.6 | 0.534487148 | 2.34E-38 | postive |
| FADS2 | AC005332.6 | 0.476542497 | 9.11E-30 | postive |
| ISCU | AC005332.6 | 0.443990837 | 1.29E-25 | postive |
| KLHL24 | AC005332.6 | 0.545548953 | 3.41E-40 | postive |
| AGPAT3 | AC005332.6 | 0.454250599 | 7.09E-27 | postive |
| ELAVL1 | AC005332.6 | 0.510881893 | 1.17E-34 | postive |
| SP1 | AC005332.6 | 0.542262438 | 1.22E-39 | postive |
| STMN1 | AC005332.6 | 0.459187059 | 1.69E-27 | postive |
| CS | AC005332.6 | 0.414052504 | 3.58E-22 | postive |
| GLS2 | AC005332.6 | 0.564671965 | 1.56E-43 | postive |
| KEAP1 | AC005332.6 | 0.413278298 | 4.34E-22 | postive |
| ATG4D | AC005332.6 | 0.443792511 | 1.36E-25 | postive |
| SNX4 | AC005332.6 | 0.435996034 | 1.16E-24 | postive |
| MAPK8 | AC005332.6 | 0.476142651 | 1.03E-29 | postive |
| MAPK9 | AC005332.6 | 0.411002606 | 7.68E-22 | postive |
| YY1AP1 | AC005332.6 | 0.497183562 | 1.22E-32 | postive |
| SIRT1 | AC005332.6 | 0.445562854 | 8.33E-26 | postive |
| ISCU | LINC01138 | 0.429795991 | 6.11E-24 | postive |
| YY1AP1 | LINC01138 | 0.402803033 | 5.76E-21 | postive |
| PHKG2 | AL355802.3 | 0.440464225 | 3.42E-25 | postive |
| GPX4 | AL117332.1 | 0.479860166 | 3.24E-30 | postive |
| CHMP6 | AL117332.1 | 0.437509435 | 7.69E-25 | postive |
| SLC2A8 | AL117332.1 | 0.491415283 | 8.11E-32 | postive |
| STMN1 | AL117332.1 | 0.456317768 | 3.90E-27 | postive |
| NOX1 | AL117332.1 | 0.41279693 | 4.90E-22 | postive |
| PHKG2 | AL117332.1 | 0.554788533 | 8.83E-42 | postive |
| PEBP1 | AL117332.1 | 0.427762205 | 1.05E-23 | postive |
| EGLN2 | AL117332.1 | 0.528667731 | 2.03E-37 | postive |
| TAZ | AL117332.1 | 0.530652997 | 9.78E-38 | postive |
| LONP1 | AL117332.1 | 0.462729851 | 5.97E-28 | postive |
| ISCU | AC025165.4 | 0.401654389 | 7.60E-21 | postive |
| FANCD2 | RAD51-AS1 | 0.42568519 | 1.81E-23 | postive |
| HELLS | RAD51-AS1 | 0.504695258 | 9.80E-34 | postive |
| TUBE1 | RAD51-AS1 | 0.540816205 | 2.12E-39 | postive |
| STMN1 | RAD51-AS1 | 0.403553569 | 4.80E-21 | postive |
| GABPB1 | RAD51-AS1 | 0.492357215 | 5.97E-32 | postive |
| MYB | RAD51-AS1 | 0.479477873 | 3.65E-30 | postive |
| PHKG2 | AL603839.3 | 0.547848536 | 1.39E-40 | postive |
| MAP1LC3A | AL603839.3 | 0.409372955 | 1.15E-21 | postive |
| EGLN2 | AL603839.3 | 0.478016783 | 5.76E-30 | postive |
| TAZ | AL603839.3 | 0.486134736 | 4.45E-31 | postive |
| HELLS | AC097376.3 | 0.486774667 | 3.63E-31 | postive |
| BRD4 | AC097376.3 | 0.443963502 | 1.30E-25 | postive |
| KLHL24 | AC097376.3 | 0.408354704 | 1.48E-21 | postive |
| SP1 | AC097376.3 | 0.413042045 | 4.61E-22 | postive |
| GABPB1 | AC097376.3 | 0.444726385 | 1.05E-25 | postive |
| GLS2 | AC097376.3 | 0.40069464 | 9.58E-21 | postive |
| MAPK8 | AC097376.3 | 0.405042638 | 3.34E-21 | postive |
| YY1AP1 | AC097376.3 | 0.495044595 | 2.47E-32 | postive |
| SIRT1 | AC097376.3 | 0.413557309 | 4.05E-22 | postive |
| CYBB | AP002954.1 | 0.415541092 | 2.46E-22 | postive |
| HRAS | AC139100.1 | 0.410730096 | 8.22E-22 | postive |
| LPIN1 | RUSC1-AS1 | 0.43596337 | 1.17E-24 | postive |
| PHKG2 | AC009133.1 | 0.443170527 | 1.62E-25 | postive |
| GPX4 | LINC02585 | 0.400317169 | 1.05E-20 | postive |
| STMN1 | LINC02585 | 0.44573014 | 7.95E-26 | postive |
| PHKG2 | LINC02585 | 0.511792498 | 8.54E-35 | postive |
| PEBP1 | LINC02585 | 0.457728364 | 2.59E-27 | postive |
| TAZ | LINC02585 | 0.423356801 | 3.31E-23 | postive |
| IREB2 | AC027319.1 | -0.40147706 | 7.93E-21 | negative |
| SP1 | AC027319.1 | -0.415960277 | 2.21E-22 | negative |
| CS | AC027319.1 | -0.407656867 | 1.76E-21 | negative |
| HRAS | AC027319.1 | 0.489017654 | 1.76E-31 | postive |
| BECN1 | AC027319.1 | -0.457884174 | 2.48E-27 | negative |
| EGLN2 | AC027319.1 | 0.406715266 | 2.22E-21 | postive |
| ENPP2 | AL133467.1 | 0.407255929 | 1.94E-21 | postive |
| HERPUD1 | AL133467.1 | 0.679696269 | 3.57E-69 | postive |
| FLT3 | AL133467.1 | 0.573254928 | 4.18E-45 | postive |
| GPX4 | AC009005.1 | 0.459920646 | 1.36E-27 | postive |
| AIFM2 | AC009005.1 | 0.489918412 | 1.32E-31 | postive |
| MT3 | AC009005.1 | 0.469059382 | 8.99E-29 | postive |
| ELAVL1 | AC009005.1 | 0.415810168 | 2.29E-22 | postive |
| STMN1 | AC009005.1 | 0.501746948 | 2.66E-33 | postive |
| PHKG2 | AC009005.1 | 0.418599692 | 1.13E-22 | postive |
| PEBP1 | AC009005.1 | 0.603836762 | 4.24E-51 | postive |
| MT3 | GLIDR | 0.472018634 | 3.66E-29 | postive |
| CDO1 | GLIDR | 0.438694417 | 5.56E-25 | postive |
| FANCD2 | AL513327.1 | 0.457946477 | 2.43E-27 | postive |
| HELLS | AL513327.1 | 0.533245927 | 3.73E-38 | postive |
| STMN1 | AL513327.1 | 0.467519529 | 1.43E-28 | postive |
| MAPK8 | AL513327.1 | 0.441185643 | 2.81E-25 | postive |
| ALOX5 | AC008972.2 | 0.462488842 | 6.41E-28 | postive |
| CYBB | AC008972.2 | 0.46945786 | 7.97E-29 | postive |
| FLT3 | AC008972.2 | 0.403867047 | 4.45E-21 | postive |
| MT3 | AL359504.1 | 0.464956673 | 3.08E-28 | postive |
| STMN1 | AL359504.1 | 0.414594674 | 3.12E-22 | postive |
| PEBP1 | AL359504.1 | 0.410443667 | 8.82E-22 | postive |
| CDO1 | AL359504.1 | 0.480060916 | 3.04E-30 | postive |
| CHMP6 | AC026471.2 | 0.420842215 | 6.35E-23 | postive |
| PHKG2 | AC026471.2 | 0.531540912 | 7.03E-38 | postive |
| MAP1LC3A | AC026471.2 | 0.405293253 | 3.14E-21 | postive |
| WIPI2 | AC026471.2 | 0.401511151 | 7.87E-21 | postive |
| TAZ | AC026471.2 | 0.422673933 | 3.95E-23 | postive |
| PHKG2 | HOTAIRM1 | 0.449352845 | 2.87E-26 | postive |
| TAZ | HOTAIRM1 | 0.420787866 | 6.44E-23 | postive |
| CHMP6 | AC053503.4 | 0.414558918 | 3.15E-22 | postive |
| NNMT | AC053503.4 | 0.460581938 | 1.12E-27 | postive |
| PHKG2 | AC053503.4 | 0.462915878 | 5.65E-28 | postive |
| EGLN2 | AC053503.4 | 0.513971257 | 3.99E-35 | postive |
| PEBP1 | LINC02482 | 0.400103343 | 1.10E-20 | postive |
| PHKG2 | LINC01176 | 0.417397257 | 1.53E-22 | postive |
| TAZ | LINC01176 | 0.470078667 | 6.60E-29 | postive |
| HELLS | AC108860.2 | 0.413200866 | 4.43E-22 | postive |
| ELAVL1 | AC108860.2 | 0.401490017 | 7.91E-21 | postive |
| STMN1 | AC108860.2 | 0.474592838 | 1.66E-29 | postive |
| GLS2 | AC108860.2 | 0.404735845 | 3.60E-21 | postive |
| PEBP1 | AC108860.2 | 0.403961409 | 4.35E-21 | postive |
| MYB | AP001574.1 | 0.491793497 | 7.17E-32 | postive |
| ACVR1B | HM13-IT1 | 0.45784888 | 2.50E-27 | postive |
| FANCD2 | AC090948.3 | 0.42056761 | 6.81E-23 | postive |
| ISCU | AC090948.3 | 0.55103014 | 3.96E-41 | postive |
| STMN1 | AC090948.3 | 0.440191999 | 3.69E-25 | postive |
| ATF4 | AC009118.3 | 0.414386781 | 3.29E-22 | postive |
| HELLS | AC009118.3 | 0.463028011 | 5.46E-28 | postive |
| FH | AC009118.3 | 0.450243013 | 2.23E-26 | postive |
| ISCU | AC009118.3 | 0.501131608 | 3.27E-33 | postive |
| STMN1 | AC009118.3 | 0.495909632 | 1.86E-32 | postive |
| GABPB1 | AC009118.3 | 0.413807307 | 3.80E-22 | postive |
| NOX1 | AC009118.3 | 0.541569125 | 1.59E-39 | postive |
| PHKG2 | AC009118.3 | 0.567166091 | 5.50E-44 | postive |
| PEBP1 | AC009118.3 | 0.452031944 | 1.34E-26 | postive |
| TAZ | AC009118.3 | 0.532456033 | 5.00E-38 | postive |
| PRKAA1 | AC034231.1 | 0.460778151 | 1.06E-27 | postive |
| GPX4 | AC010503.5 | 0.430976392 | 4.46E-24 | postive |
| ISCU | AC010503.5 | 0.440273265 | 3.61E-25 | postive |
| NOX1 | AC010503.5 | 0.51202068 | 7.88E-35 | postive |
| ACSL4 | AC010503.5 | -0.411889755 | 6.15E-22 | negative |
| KEAP1 | AC010503.5 | 0.416507949 | 1.92E-22 | postive |
| PHKG2 | AC010503.5 | 0.50335042 | 1.55E-33 | postive |
| ATG4D | AC010503.5 | 0.581440852 | 1.20E-46 | postive |
| SAT1 | AC010503.5 | 0.406732235 | 2.21E-21 | postive |
| CDKN2A | AC010503.5 | 0.423548944 | 3.15E-23 | postive |
| PEBP1 | AC010503.5 | 0.410700926 | 8.27E-22 | postive |
| ANO6 | AC010503.5 | -0.495945509 | 1.84E-32 | negative |
| LONP1 | AC010503.5 | 0.404009876 | 4.29E-21 | postive |
| ATF4 | AC018926.2 | 0.44910598 | 3.08E-26 | postive |
| PHKG2 | AC018926.2 | 0.4406566 | 3.25E-25 | postive |
| ENPP2 | AL590764.1 | 0.474996862 | 1.47E-29 | postive |
| ISCU | AL590764.1 | 0.419813474 | 8.27E-23 | postive |
| ALOX5 | AL590764.1 | 0.506436829 | 5.41E-34 | postive |
| CYBB | AL590764.1 | 0.541587956 | 1.58E-39 | postive |
| FLT3 | AL590764.1 | 0.614438236 | 2.48E-53 | postive |
| IFNG | AL590764.1 | 0.597662015 | 7.76E-50 | postive |
| HIC1 | CASC15 | 0.4673273 | 1.51E-28 | postive |
| NOX4 | CASC15 | 0.50779878 | 3.39E-34 | postive |
| TUBE1 | AC008105.1 | 0.427469853 | 1.13E-23 | postive |
| ELAVL1 | AC008105.1 | 0.414782705 | 2.98E-22 | postive |
| GABPB1 | AC008105.1 | 0.404045874 | 4.26E-21 | postive |
| LPIN1 | AC008105.1 | 0.48369564 | 9.68E-31 | postive |
| HELLS | AC012360.3 | 0.456883956 | 3.31E-27 | postive |
| TUBE1 | AC012360.3 | 0.492986503 | 4.86E-32 | postive |
| ELAVL1 | AC012360.3 | 0.467091846 | 1.63E-28 | postive |
| STMN1 | AC012360.3 | 0.439359542 | 4.64E-25 | postive |
| GABPB1 | AC012360.3 | 0.466498791 | 1.94E-28 | postive |
| SNX4 | AC012360.3 | 0.444391944 | 1.16E-25 | postive |
| PHKG2 | AL096828.3 | 0.405669575 | 2.86E-21 | postive |
| TAZ | AL096828.3 | 0.474096881 | 1.94E-29 | postive |
| WIPI2 | AC092171.2 | 0.534515807 | 2.32E-38 | postive |
| KLHL24 | KCNMB2-AS1 | 0.532983254 | 4.11E-38 | postive |
| PIK3CA | KCNMB2-AS1 | 0.497836515 | 9.82E-33 | postive |
| OXSR1 | AC112220.2 | 0.508319629 | 2.84E-34 | postive |
| RPL8 | SNHG29 | 0.419349146 | 9.32E-23 | postive |
| YWHAE | SNHG29 | 0.529403698 | 1.55E-37 | postive |
| IL6 | IER3-AS1 | 0.513323693 | 5.00E-35 | postive |
| CXCL2 | IER3-AS1 | 0.475116792 | 1.42E-29 | postive |
| ENPP2 | PCED1B-AS1 | 0.493196859 | 4.54E-32 | postive |
| ISCU | PCED1B-AS1 | 0.431362195 | 4.03E-24 | postive |
| HERPUD1 | PCED1B-AS1 | 0.515427686 | 2.39E-35 | postive |
| ALOX5 | PCED1B-AS1 | 0.537150175 | 8.58E-39 | postive |
| CYBB | PCED1B-AS1 | 0.511176837 | 1.06E-34 | postive |
| FLT3 | PCED1B-AS1 | 0.67779977 | 1.18E-68 | postive |
| IFNG | PCED1B-AS1 | 0.581589281 | 1.12E-46 | postive |
| HIF1A | AL135818.2 | 0.414492555 | 3.20E-22 | postive |
| GPX4 | AL118516.1 | 0.405343841 | 3.10E-21 | postive |
| PHKG2 | AL118516.1 | 0.41556235 | 2.44E-22 | postive |
| TAZ | AL118516.1 | 0.411208463 | 7.29E-22 | postive |
| STMN1 | AC000068.2 | 0.516002695 | 1.95E-35 | postive |
| NOX1 | AC000068.2 | 0.461419799 | 8.79E-28 | postive |
| PHKG2 | AC000068.2 | 0.53472142 | 2.14E-38 | postive |
| TAZ | AC000068.2 | 0.517741722 | 1.06E-35 | postive |
| KLHL24 | LINC01096 | 0.406190207 | 2.52E-21 | postive |
| HELLS | AC073655.2 | 0.406728745 | 2.21E-21 | postive |
| PHKG2 | AC073655.2 | 0.417624076 | 1.45E-22 | postive |
| TAZ | AC073655.2 | 0.447987912 | 4.22E-26 | postive |
| SETD1B | CYTOR | -0.420781687 | 6.45E-23 | negative |
| RPL8 | CYTOR | 0.404691393 | 3.64E-21 | postive |
| SP1 | CYTOR | -0.427168001 | 1.22E-23 | negative |
| NNMT | CYTOR | 0.505577719 | 7.26E-34 | postive |
| BECN1 | CYTOR | -0.405480693 | 3.00E-21 | negative |
| SLC40A1 | CCR5AS | 0.421856951 | 4.89E-23 | postive |
| ENPP2 | CCR5AS | 0.492881544 | 5.03E-32 | postive |
| ALOX5 | CCR5AS | 0.651850613 | 6.19E-62 | postive |
| CYBB | CCR5AS | 0.770581409 | 1.11E-99 | postive |
| FLT3 | CCR5AS | 0.496826411 | 1.37E-32 | postive |
| IFNG | CCR5AS | 0.519643347 | 5.36E-36 | postive |
| TLR4 | CCR5AS | 0.526693653 | 4.20E-37 | postive |
| AGPAT3 | LINC00649 | 0.400313966 | 1.05E-20 | postive |
| FANCD2 | AC010319.4 | 0.407299253 | 1.92E-21 | postive |
| HELLS | AC010319.4 | 0.510197738 | 1.48E-34 | postive |
| ISCU | AC010319.4 | 0.413022975 | 4.63E-22 | postive |
| TUBE1 | AC010319.4 | 0.449759898 | 2.56E-26 | postive |
| ELAVL1 | AC010319.4 | 0.422468927 | 4.17E-23 | postive |
| STMN1 | AC010319.4 | 0.459677355 | 1.47E-27 | postive |
| GLS2 | AC010319.4 | 0.45626853 | 3.96E-27 | postive |
| ATG4D | AC010319.4 | 0.477387373 | 7.01E-30 | postive |
| KRAS | NDUFV2-AS1 | 0.4082476 | 1.52E-21 | postive |
| HELLS | AC129510.1 | 0.480392871 | 2.74E-30 | postive |
| TUBE1 | AC129510.1 | 0.496731092 | 1.42E-32 | postive |
| MYB | AC129510.1 | 0.419665955 | 8.59E-23 | postive |
| PHKG2 | MIATNB | 0.470310863 | 6.15E-29 | postive |
| EGLN2 | MIATNB | 0.43524663 | 1.42E-24 | postive |
| AIFM2 | AL365181.3 | 0.445946254 | 7.48E-26 | postive |
| TXNRD1 | AL365181.3 | 0.457360335 | 2.88E-27 | postive |
| SRXN1 | AL365181.3 | 0.405194001 | 3.22E-21 | postive |
| MAFG | AL365181.3 | 0.538899333 | 4.42E-39 | postive |
| SLC2A12 | AL365181.3 | 0.401426655 | 8.03E-21 | postive |
| PHKG2 | LINC00685 | 0.506184908 | 5.90E-34 | postive |
| TAZ | LINC00685 | 0.445577183 | 8.30E-26 | postive |
| FH | LINC01703 | 0.419659976 | 8.60E-23 | postive |
| PRDX6 | LINC01703 | 0.443762379 | 1.38E-25 | postive |
| TAZ | LINC01703 | 0.410331158 | 9.07E-22 | postive |
| MTOR | AC093752.3 | 0.461348423 | 8.97E-28 | postive |
| SETD1B | AC093752.3 | 0.564879317 | 1.43E-43 | postive |
| IREB2 | AC093752.3 | 0.449099812 | 3.08E-26 | postive |
| SP1 | AC093752.3 | 0.566186879 | 8.29E-44 | postive |
| ACVR1B | AC093752.3 | 0.420648667 | 6.67E-23 | postive |
| ATM | AC093752.3 | 0.484104192 | 8.50E-31 | postive |
| SIRT1 | AC093752.3 | 0.407420529 | 1.86E-21 | postive |
| ISCU | AC034236.2 | 0.40705079 | 2.04E-21 | postive |
| STMN1 | AC034236.2 | 0.425684369 | 1.81E-23 | postive |
| PHKG2 | AC034236.2 | 0.577832266 | 5.81E-46 | postive |
| EGLN2 | AC034236.2 | 0.478895245 | 4.38E-30 | postive |
| TAZ | AC034236.2 | 0.514418574 | 3.41E-35 | postive |
| EGLN2 | SLC12A5-AS1 | 0.478645623 | 4.74E-30 | postive |
| NOS2 | AC097713.1 | 0.471568862 | 4.20E-29 | postive |
| GPX4 | AC106900.1 | 0.517273447 | 1.25E-35 | postive |
| CISD1 | AC106900.1 | 0.439513316 | 4.45E-25 | postive |
| CARS1 | AC106900.1 | 0.418357241 | 1.20E-22 | postive |
| PHKG2 | AC106900.1 | 0.409138371 | 1.22E-21 | postive |
| MAP1LC3A | AC106900.1 | 0.402340284 | 6.44E-21 | postive |
| GPX4 | AC104126.1 | 0.484092237 | 8.53E-31 | postive |
| CISD1 | AC104126.1 | 0.481878998 | 1.72E-30 | postive |
| ISCU | AC104126.1 | 0.502727079 | 1.91E-33 | postive |
| CARS1 | AC104126.1 | 0.448603836 | 3.54E-26 | postive |
| MAP1LC3A | AC104126.1 | 0.450050911 | 2.35E-26 | postive |
| SOCS1 | AC104126.1 | 0.460004031 | 1.33E-27 | postive |
| STMN1 | AC010719.1 | 0.447927388 | 4.29E-26 | postive |
| PHKG2 | AC010719.1 | 0.530609363 | 9.94E-38 | postive |
| TAZ | AC010719.1 | 0.501373748 | 3.01E-33 | postive |
| PHKG2 | Z97653.1 | 0.466996219 | 1.67E-28 | postive |
| MAP1LC3A | Z97653.1 | 0.498158684 | 8.82E-33 | postive |
| ASNS | LINC02875 | 0.443190174 | 1.61E-25 | postive |
| PCK2 | LINC02875 | 0.402738627 | 5.85E-21 | postive |
| GABARAPL1 | LINC02875 | 0.415552819 | 2.45E-22 | postive |
| SP1 | AP001469.2 | 0.418860956 | 1.06E-22 | postive |
| YY1AP1 | AP001469.2 | 0.408734888 | 1.35E-21 | postive |
| HELLS | AC015813.1 | 0.422768 | 3.86E-23 | postive |
| SLC2A8 | AC046143.2 | 0.426856551 | 1.33E-23 | postive |
| PHKG2 | AC046143.2 | 0.564347179 | 1.78E-43 | postive |
| EGLN2 | AC046143.2 | 0.593853371 | 4.51E-49 | postive |
| TAZ | AC046143.2 | 0.462417362 | 6.54E-28 | postive |
| FANCD2 | OIP5-AS1 | 0.431950451 | 3.44E-24 | postive |
| HELLS | OIP5-AS1 | 0.520679261 | 3.70E-36 | postive |
| KLHL24 | OIP5-AS1 | 0.493544026 | 4.05E-32 | postive |
| SETD1B | OIP5-AS1 | 0.459777179 | 1.42E-27 | postive |
| EIF2AK4 | OIP5-AS1 | 0.447532625 | 4.79E-26 | postive |
| IREB2 | OIP5-AS1 | 0.609631026 | 2.61E-52 | postive |
| ELAVL1 | OIP5-AS1 | 0.432314098 | 3.12E-24 | postive |
| SP1 | OIP5-AS1 | 0.649335341 | 2.56E-61 | postive |
| GABPB1 | OIP5-AS1 | 0.4422205 | 2.11E-25 | postive |
| CS | OIP5-AS1 | 0.418988349 | 1.02E-22 | postive |
| PIK3CA | OIP5-AS1 | 0.401378466 | 8.12E-21 | postive |
| GLS2 | OIP5-AS1 | 0.489172548 | 1.68E-31 | postive |
| NCOA4 | OIP5-AS1 | 0.424704254 | 2.33E-23 | postive |
| MAPK8 | OIP5-AS1 | 0.406190664 | 2.52E-21 | postive |
| ACVR1B | OIP5-AS1 | 0.447102859 | 5.41E-26 | postive |
| LPIN1 | OIP5-AS1 | 0.404676335 | 3.65E-21 | postive |
| ATM | OIP5-AS1 | 0.474280293 | 1.83E-29 | postive |
| SIRT1 | OIP5-AS1 | 0.524255771 | 1.02E-36 | postive |
| ISCU | LINC00476 | 0.407496238 | 1.83E-21 | postive |
| SLC2A8 | LINC00476 | 0.444438587 | 1.14E-25 | postive |
| STMN1 | LINC00476 | 0.442894113 | 1.75E-25 | postive |
| PHKG2 | LINC00476 | 0.429339215 | 6.90E-24 | postive |
| PLIN4 | AL137246.2 | 0.787366728 | 6.72E-107 | postive |
| PRKAA2 | AL137246.2 | 0.45571678 | 4.64E-27 | postive |
| PEBP1 | ERICD | 0.45186661 | 1.40E-26 | postive |
| ATF4 | AC013731.1 | 0.416853928 | 1.76E-22 | postive |
| TUBE1 | AC013731.1 | 0.433108672 | 2.52E-24 | postive |
| TAZ | AC013731.1 | 0.463625035 | 4.58E-28 | postive |
| PRKAA2 | AL137782.1 | 0.416679662 | 1.84E-22 | postive |
| STMN1 | C5orf66-AS1 | 0.411720162 | 6.42E-22 | postive |
| NOX1 | C5orf66-AS1 | 0.406801035 | 2.17E-21 | postive |
| PHKG2 | C5orf66-AS1 | 0.411841036 | 6.23E-22 | postive |
| CDKN2A | C5orf66-AS1 | 0.416961528 | 1.71E-22 | postive |
| GPX4 | LINC00847 | 0.418280089 | 1.22E-22 | postive |
| SQSTM1 | LINC00847 | 0.421446799 | 5.43E-23 | postive |
| ISCU | LINC00847 | 0.534079434 | 2.73E-38 | postive |
| KLHL24 | LINC00847 | 0.428365693 | 8.92E-24 | postive |
| STMN1 | LINC00847 | 0.47239311 | 3.26E-29 | postive |
| GLS2 | LINC00847 | 0.463106335 | 5.34E-28 | postive |
| GOT1 | LINC00847 | 0.415772529 | 2.32E-22 | postive |
| ATG4D | LINC00847 | 0.455099876 | 5.55E-27 | postive |
| MAPK9 | LINC00847 | 0.525494629 | 6.50E-37 | postive |
| CISD2 | UBE2D3-AS1 | 0.495578259 | 2.07E-32 | postive |
| KLHL24 | AC091271.1 | 0.401400007 | 8.08E-21 | postive |
| GABPB1 | AC091271.1 | 0.401786043 | 7.36E-21 | postive |
| HELLS | IQCH-AS1 | 0.404436595 | 3.87E-21 | postive |
| ISCU | IQCH-AS1 | 0.464684069 | 3.34E-28 | postive |
| STMN1 | IQCH-AS1 | 0.46479863 | 3.23E-28 | postive |
| NOX1 | IQCH-AS1 | 0.444357062 | 1.17E-25 | postive |
| GLS2 | IQCH-AS1 | 0.434329236 | 1.82E-24 | postive |
| PEBP1 | IQCH-AS1 | 0.476568583 | 9.04E-30 | postive |
| MAPK8 | IQCH-AS1 | 0.403115608 | 5.34E-21 | postive |
| TUBE1 | AL022328.1 | 0.435946562 | 1.17E-24 | postive |
| PHKG2 | AL022328.1 | 0.41067431 | 8.33E-22 | postive |
| MYB | AL022328.1 | 0.404151526 | 4.15E-21 | postive |
| YY1AP1 | AL022328.1 | 0.430017347 | 5.76E-24 | postive |
| TUBE1 | NCBP2-AS1 | 0.451177062 | 1.71E-26 | postive |
| SNX4 | NCBP2-AS1 | 0.423226066 | 3.43E-23 | postive |
| ACVR1B | NCBP2-AS1 | 0.408160901 | 1.55E-21 | postive |
| NFE2L2 | ATP2B1-AS1 | 0.460958151 | 1.01E-27 | postive |
| KLHL24 | ATP2B1-AS1 | 0.480653172 | 2.53E-30 | postive |
| GABARAPL1 | ATP2B1-AS1 | 0.527955829 | 2.64E-37 | postive |
| PHKG2 | AC026401.3 | 0.453333815 | 9.22E-27 | postive |
| TUBE1 | AL161669.3 | 0.408254674 | 1.52E-21 | postive |
| LPIN1 | AL161669.3 | 0.434788919 | 1.61E-24 | postive |
| PHKG2 | AC004687.1 | 0.434145896 | 1.91E-24 | postive |
| IFNG | AC004687.1 | 0.436957699 | 8.93E-25 | postive |
| EGLN2 | AC004687.1 | 0.446977772 | 5.60E-26 | postive |
| STMN1 | AC093788.1 | 0.405043105 | 3.34E-21 | postive |
| NOX1 | AC093788.1 | 0.413860199 | 3.75E-22 | postive |
| PHKG2 | AC093788.1 | 0.414230802 | 3.42E-22 | postive |
| TAZ | AC093788.1 | 0.431523114 | 3.86E-24 | postive |
| GPX4 | PXN-AS1 | 0.570242212 | 1.51E-44 | postive |
| ATF4 | PXN-AS1 | 0.424935265 | 2.20E-23 | postive |
| ISCU | PXN-AS1 | 0.447670721 | 4.61E-26 | postive |
| SLC2A8 | PXN-AS1 | 0.407433907 | 1.86E-21 | postive |
| STMN1 | PXN-AS1 | 0.499800259 | 5.10E-33 | postive |
| NOX1 | PXN-AS1 | 0.410241716 | 9.28E-22 | postive |
| PHKG2 | PXN-AS1 | 0.591214925 | 1.51E-48 | postive |
| MAP1LC3A | PXN-AS1 | 0.484961718 | 6.47E-31 | postive |
| PEBP1 | PXN-AS1 | 0.437793353 | 7.11E-25 | postive |
| EGLN2 | PXN-AS1 | 0.467877476 | 1.28E-28 | postive |
| TAZ | PXN-AS1 | 0.482109335 | 1.60E-30 | postive |
| PANX1 | PXN-AS1 | -0.403030407 | 5.45E-21 | negative |
| LONP1 | PXN-AS1 | 0.42607774 | 1.63E-23 | postive |
| PHKG2 | AL133342.1 | 0.427284057 | 1.19E-23 | postive |
| EGLN2 | AL133342.1 | 0.475378171 | 1.31E-29 | postive |
| EGLN2 | IRX4-AS1 | 0.429691307 | 6.28E-24 | postive |
| ALOX5 | TRG-AS1 | 0.453716388 | 8.26E-27 | postive |
| CYBB | TRG-AS1 | 0.511528817 | 9.36E-35 | postive |
| FLT3 | TRG-AS1 | 0.602180433 | 9.30E-51 | postive |
| IFNG | TRG-AS1 | 0.736515105 | 9.19E-87 | postive |
| CHMP6 | AP001160.3 | 0.422035564 | 4.67E-23 | postive |
| PHKG2 | AP001160.3 | 0.537705382 | 6.95E-39 | postive |
| ANO6 | AP001160.3 | -0.418383525 | 1.19E-22 | negative |
| PHKG2 | ZNF213-AS1 | 0.610784921 | 1.49E-52 | postive |
| TAZ | ZNF213-AS1 | 0.429047466 | 7.45E-24 | postive |
| HELLS | AP001469.3 | 0.459199537 | 1.69E-27 | postive |
| ELAVL1 | AP001469.3 | 0.439494323 | 4.47E-25 | postive |
| STMN1 | AP001469.3 | 0.49324391 | 4.47E-32 | postive |
| PHKG2 | AP001469.3 | 0.479268495 | 3.90E-30 | postive |
| YY1AP1 | AP001469.3 | 0.479113284 | 4.09E-30 | postive |
| TUBE1 | AC009495.3 | 0.423717585 | 3.02E-23 | postive |
| GPX4 | SPINT1-AS1 | 0.449239278 | 2.96E-26 | postive |
| ATF4 | SPINT1-AS1 | 0.410090914 | 9.63E-22 | postive |
| PRDX6 | SPINT1-AS1 | 0.432467951 | 3.00E-24 | postive |
| SLC2A8 | SPINT1-AS1 | 0.447541931 | 4.78E-26 | postive |
| NOX1 | SPINT1-AS1 | 0.523196027 | 1.50E-36 | postive |
| SLC1A5 | SPINT1-AS1 | 0.444065325 | 1.27E-25 | postive |
| PHKG2 | SPINT1-AS1 | 0.576499741 | 1.03E-45 | postive |
| MAP1LC3A | SPINT1-AS1 | 0.401273092 | 8.33E-21 | postive |
| BID | SPINT1-AS1 | 0.414909035 | 2.88E-22 | postive |
| PEBP1 | SPINT1-AS1 | 0.548454412 | 1.09E-40 | postive |
| ANO6 | SPINT1-AS1 | -0.487640484 | 2.75E-31 | negative |
| EGLN2 | SPINT1-AS1 | 0.529655324 | 1.41E-37 | postive |
| TAZ | SPINT1-AS1 | 0.517883055 | 1.00E-35 | postive |
| MTDH | SPINT1-AS1 | -0.408813533 | 1.32E-21 | negative |
| PANX1 | SPINT1-AS1 | -0.407466515 | 1.84E-21 | negative |
| HIC1 | BNC2-AS1 | 0.425427447 | 1.93E-23 | postive |
| IFNG | LINC02084 | 0.485555853 | 5.36E-31 | postive |
| GPX4 | AC084036.1 | 0.403549246 | 4.80E-21 | postive |
| ISCU | AC084036.1 | 0.435052496 | 1.50E-24 | postive |
| STMN1 | AC084036.1 | 0.443836574 | 1.35E-25 | postive |
| NOX1 | AC084036.1 | 0.48670378 | 3.71E-31 | postive |
| PHKG2 | AC084036.1 | 0.486082745 | 4.53E-31 | postive |
| TAZ | AC084036.1 | 0.419754551 | 8.40E-23 | postive |
| TUBE1 | AL021707.2 | 0.470436646 | 5.92E-29 | postive |
| GPX4 | HEIH | 0.502334278 | 2.18E-33 | postive |
| CISD1 | HEIH | 0.461657826 | 8.19E-28 | postive |
| ATF4 | HEIH | 0.409418404 | 1.14E-21 | postive |
| ISCU | HEIH | 0.594692319 | 3.07E-49 | postive |
| STMN1 | HEIH | 0.430176565 | 5.52E-24 | postive |
| NOX1 | HEIH | 0.443002963 | 1.70E-25 | postive |
| ATG4D | HEIH | 0.412620561 | 5.12E-22 | postive |
| MAP1LC3A | HEIH | 0.421488379 | 5.37E-23 | postive |
| TAZ | HEIH | 0.456005469 | 4.27E-27 | postive |
| NOX1 | AC026740.1 | 0.442167017 | 2.14E-25 | postive |
| EGLN2 | AC026740.1 | 0.422153237 | 4.53E-23 | postive |
| TAZ | AC026740.1 | 0.400082466 | 1.11E-20 | postive |
| ISCU | AC004865.2 | 0.421155823 | 5.86E-23 | postive |
| ALOX5 | AC004865.2 | 0.409242224 | 1.19E-21 | postive |
| FLT3 | AC004865.2 | 0.430880655 | 4.58E-24 | postive |
| ATF4 | AC107081.2 | 0.438285051 | 6.22E-25 | postive |
| PHKG2 | AC107081.2 | 0.529818047 | 1.33E-37 | postive |
| EGLN2 | AC107081.2 | 0.447129199 | 5.37E-26 | postive |
| TAZ | AC107081.2 | 0.537108167 | 8.72E-39 | postive |
| ENPP2 | LINC01150 | 0.422895271 | 3.73E-23 | postive |
| ALOX5 | LINC01150 | 0.58966911 | 3.04E-48 | postive |
| CYBB | LINC01150 | 0.566820121 | 6.36E-44 | postive |
| ZEB1 | LINC01150 | 0.427097395 | 1.25E-23 | postive |
| TLR4 | LINC01150 | 0.42916635 | 7.22E-24 | postive |
| ATG3 | NCK1-DT | 0.434906944 | 1.56E-24 | postive |
| ATG4D | NCK1-DT | 0.401360792 | 8.16E-21 | postive |
| BID | NCK1-DT | 0.426694941 | 1.39E-23 | postive |
| KLHL24 | AL353804.1 | 0.429516392 | 6.58E-24 | postive |
| SP1 | AL353804.1 | 0.420831452 | 6.37E-23 | postive |
| ATM | AL353804.1 | 0.413829773 | 3.78E-22 | postive |
| NOX1 | AL354836.1 | 0.456842842 | 3.35E-27 | postive |
| PHKG2 | AL354836.1 | 0.599857928 | 2.78E-50 | postive |
| EGLN2 | AL354836.1 | 0.447019514 | 5.54E-26 | postive |
| TAZ | AL354836.1 | 0.527919564 | 2.68E-37 | postive |
| BRD4 | MCCC1-AS1 | 0.410096253 | 9.62E-22 | postive |
| KLHL24 | MCCC1-AS1 | 0.484691196 | 7.06E-31 | postive |
| TUBE1 | MCCC1-AS1 | 0.495042807 | 2.47E-32 | postive |
| CEBPG | MCCC1-AS1 | 0.427660763 | 1.07E-23 | postive |
| ELAVL1 | MCCC1-AS1 | 0.417110591 | 1.65E-22 | postive |
| GABPB1 | MCCC1-AS1 | 0.446405391 | 6.58E-26 | postive |
| PIK3CA | MCCC1-AS1 | 0.410998042 | 7.68E-22 | postive |
| ATG3 | MCCC1-AS1 | 0.403407583 | 4.97E-21 | postive |
| SNX4 | MCCC1-AS1 | 0.468251956 | 1.15E-28 | postive |
| LPIN1 | MCCC1-AS1 | 0.40092479 | 9.06E-21 | postive |
| FANCD2 | AC090948.2 | 0.432420849 | 3.04E-24 | postive |
| TP53 | AC090948.2 | 0.418919847 | 1.04E-22 | postive |
| GCH1 | AC090948.2 | 0.432981101 | 2.61E-24 | postive |
| AGPAT3 | AC090948.2 | 0.427508349 | 1.12E-23 | postive |
| SP1 | AC090948.2 | 0.529001456 | 1.80E-37 | postive |
| CYBB | AC090948.2 | 0.401686642 | 7.54E-21 | postive |
| FLT3 | AC090948.2 | 0.537438294 | 7.69E-39 | postive |
| ATG7 | AC090948.2 | 0.426987092 | 1.28E-23 | postive |
| NCOA4 | AC090948.2 | 0.439464597 | 4.51E-25 | postive |
| MAPK8 | AC090948.2 | 0.446905613 | 5.72E-26 | postive |
| ATM | AC090948.2 | 0.442656454 | 1.87E-25 | postive |
| GPX4 | AC020765.2 | 0.497443961 | 1.12E-32 | postive |
| ATF4 | AC020765.2 | 0.424915315 | 2.21E-23 | postive |
| RPL8 | AC020765.2 | 0.414465515 | 3.22E-22 | postive |
| SLC2A8 | AC020765.2 | 0.482623646 | 1.36E-30 | postive |
| STMN1 | AC020765.2 | 0.466561849 | 1.91E-28 | postive |
| NOX1 | AC020765.2 | 0.462597319 | 6.21E-28 | postive |
| PHKG2 | AC020765.2 | 0.648296671 | 4.58E-61 | postive |
| MAP1LC3A | AC020765.2 | 0.408761567 | 1.34E-21 | postive |
| PEBP1 | AC020765.2 | 0.432168879 | 3.25E-24 | postive |
| SOCS1 | AC020765.2 | 0.408483067 | 1.43E-21 | postive |
| EGLN2 | AC020765.2 | 0.517306064 | 1.23E-35 | postive |
| TAZ | AC020765.2 | 0.564389129 | 1.75E-43 | postive |
| LONP1 | AC020765.2 | 0.428570849 | 8.45E-24 | postive |
| TUBE1 | AL513534.3 | 0.422735505 | 3.89E-23 | postive |
| GABPB1 | AL513534.3 | 0.410871616 | 7.93E-22 | postive |
| PRDX6 | ASH1L-AS1 | 0.405437743 | 3.03E-21 | postive |
| PHKG2 | ASH1L-AS1 | 0.431509131 | 3.87E-24 | postive |
| YY1AP1 | ASH1L-AS1 | 0.43790693 | 6.90E-25 | postive |
| RPL8 | AC131009.3 | 0.475766996 | 1.16E-29 | postive |
| EGLN2 | AC131009.3 | 0.491079345 | 9.05E-32 | postive |
| CHMP6 | ZNF426-DT | 0.460993107 | 9.96E-28 | postive |
| NGB | ZNF426-DT | 0.422989731 | 3.64E-23 | postive |
| PHKG2 | ZNF426-DT | 0.439993726 | 3.90E-25 | postive |
| BID | ZNF426-DT | 0.400991118 | 8.92E-21 | postive |
| HIC1 | AL139393.3 | 0.402813989 | 5.74E-21 | postive |
| TUBE1 | ZNF674-AS1 | 0.414007475 | 3.62E-22 | postive |
| SELENOS | SPATA41 | 0.407749817 | 1.72E-21 | postive |
| SLC2A8 | SPATA41 | 0.424348335 | 2.56E-23 | postive |
| NOX1 | SPATA41 | 0.454564568 | 6.47E-27 | postive |
| KEAP1 | SPATA41 | 0.470460611 | 5.88E-29 | postive |
| PHKG2 | SPATA41 | 0.488546392 | 2.05E-31 | postive |
| ATG4D | SPATA41 | 0.446063345 | 7.24E-26 | postive |
| TAZ | SPATA41 | 0.539403706 | 3.64E-39 | postive |
| PRKAA1 | AC093297.2 | 0.401413685 | 8.06E-21 | postive |
| GPX4 | AC104113.1 | 0.523604871 | 1.29E-36 | postive |
| CISD1 | AC104113.1 | 0.449960378 | 2.41E-26 | postive |
| ISCU | AC104113.1 | 0.550381957 | 5.12E-41 | postive |
| STMN1 | AC104113.1 | 0.448607545 | 3.54E-26 | postive |
| NOX1 | AC104113.1 | 0.608591849 | 4.33E-52 | postive |
| PHKG2 | AC104113.1 | 0.461841736 | 7.76E-28 | postive |
| TAZ | AC104113.1 | 0.402002714 | 6.99E-21 | postive |
| TAZ | LINC00653 | 0.474353832 | 1.79E-29 | postive |
| ATF4 | SNHG17 | 0.476695906 | 8.69E-30 | postive |
| PHKG2 | SNHG17 | 0.415536519 | 2.46E-22 | postive |
| TAZ | SNHG17 | 0.417171518 | 1.62E-22 | postive |
| LONP1 | SNHG17 | 0.412688582 | 5.04E-22 | postive |
| GPX4 | AC079922.2 | 0.450536909 | 2.05E-26 | postive |
| STMN1 | AC079922.2 | 0.412838513 | 4.85E-22 | postive |
| PHKG2 | AC079922.2 | 0.532722798 | 4.53E-38 | postive |
| MAP1LC3A | AC079922.2 | 0.424821592 | 2.26E-23 | postive |
| EGLN2 | AC079922.2 | 0.429767528 | 6.16E-24 | postive |
| TAZ | AC079922.2 | 0.470967547 | 5.04E-29 | postive |
| GABARAPL2 | VPS13B-DT | 0.433933492 | 2.02E-24 | postive |
| PEBP1 | VPS13B-DT | 0.501803656 | 2.61E-33 | postive |
| TAZ | VPS13B-DT | 0.428867082 | 7.82E-24 | postive |
| GPX4 | AC069281.2 | 0.437118895 | 8.55E-25 | postive |
| SLC2A8 | AC069281.2 | 0.451786156 | 1.44E-26 | postive |
| PHKG2 | AC069281.2 | 0.618155132 | 3.90E-54 | postive |
| EGLN2 | AC069281.2 | 0.539595972 | 3.39E-39 | postive |
| TAZ | AC069281.2 | 0.487312899 | 3.05E-31 | postive |
| PHKG2 | AC012442.2 | 0.404102751 | 4.20E-21 | postive |
| BRD4 | AL109976.1 | 0.452221756 | 1.27E-26 | postive |
| PSAT1 | AL109976.1 | 0.410267953 | 9.22E-22 | postive |
| KLHL24 | AL109976.1 | 0.446527457 | 6.36E-26 | postive |
| TUBE1 | AL109976.1 | 0.434669712 | 1.66E-24 | postive |
| GLS2 | AL109976.1 | 0.431688883 | 3.69E-24 | postive |
| ABCC1 | AL109976.1 | 0.40043528 | 1.02E-20 | postive |
| ALOXE3 | AC112236.1 | 0.454309719 | 6.97E-27 | postive |
| PML | AC009950.1 | 0.54312174 | 8.75E-40 | postive |
| ATF4 | AL136295.7 | 0.414794156 | 2.97E-22 | postive |
| ISCU | AL136295.7 | 0.477112587 | 7.63E-30 | postive |
| STMN1 | AL136295.7 | 0.425631709 | 1.83E-23 | postive |
| NOX1 | AL136295.7 | 0.474479896 | 1.72E-29 | postive |
| PHKG2 | AL136295.7 | 0.554024969 | 1.20E-41 | postive |
| EGLN2 | AL136295.7 | 0.412775071 | 4.93E-22 | postive |
| TAZ | AL136295.7 | 0.569730741 | 1.87E-44 | postive |
| ATG3 | TBILA | 0.435130649 | 1.47E-24 | postive |
| PHKG2 | AL358472.3 | 0.504996467 | 8.85E-34 | postive |
| TAZ | AL358472.3 | 0.437502978 | 7.70E-25 | postive |
| FANCD2 | AC139795.2 | 0.444223061 | 1.21E-25 | postive |
| TP53 | AC139795.2 | 0.46289026 | 5.69E-28 | postive |
| HELLS | AC139795.2 | 0.557546434 | 2.90E-42 | postive |
| FADS2 | AC139795.2 | 0.444998691 | 9.75E-26 | postive |
| ISCU | AC139795.2 | 0.436225703 | 1.09E-24 | postive |
| KLHL24 | AC139795.2 | 0.451416494 | 1.60E-26 | postive |
| AGPAT3 | AC139795.2 | 0.436192618 | 1.10E-24 | postive |
| SP1 | AC139795.2 | 0.516589699 | 1.59E-35 | postive |
| GLS2 | AC139795.2 | 0.603471425 | 5.04E-51 | postive |
| ATG4D | AC139795.2 | 0.434380548 | 1.79E-24 | postive |
| MAPK8 | AC139795.2 | 0.477256866 | 7.30E-30 | postive |
| MAPK9 | AC139795.2 | 0.471940554 | 3.75E-29 | postive |
| ACVR1B | AC139795.2 | 0.465011807 | 3.03E-28 | postive |
| LPIN1 | AC139795.2 | 0.420723719 | 6.54E-23 | postive |
| YY1AP1 | AC139795.2 | 0.47084792 | 5.23E-29 | postive |
| ISCU | KIF1C-AS1 | 0.416804801 | 1.78E-22 | postive |
| PHKG2 | KIF1C-AS1 | 0.461836133 | 7.77E-28 | postive |
| MAP1LC3A | KIF1C-AS1 | 0.407347266 | 1.90E-21 | postive |
| SOCS1 | KIF1C-AS1 | 0.401190897 | 8.50E-21 | postive |
| TAZ | KIF1C-AS1 | 0.400804286 | 9.33E-21 | postive |
| MIOX | LINC01820 | 0.414245026 | 3.41E-22 | postive |
| SLC7A11 | LINC02561 | 0.499777891 | 5.14E-33 | postive |
| AKR1C1 | LINC02561 | 0.586427982 | 1.31E-47 | postive |
| AKR1C2 | LINC02561 | 0.510729681 | 1.23E-34 | postive |
| AKR1C3 | LINC02561 | 0.590798213 | 1.82E-48 | postive |
| GCLC | LINC02561 | 0.430169092 | 5.53E-24 | postive |
| NQO1 | LINC02561 | 0.470035359 | 6.69E-29 | postive |
| FTH1 | LINC02561 | 0.403654158 | 4.68E-21 | postive |
| TXNRD1 | LINC02561 | 0.67256857 | 3.02E-67 | postive |
| SRXN1 | LINC02561 | 0.572811592 | 5.05E-45 | postive |
| GPX2 | LINC02561 | 0.582608702 | 7.16E-47 | postive |
| FTL | LINC02561 | 0.435237265 | 1.42E-24 | postive |
| TFRC | LINC02561 | 0.418335467 | 1.21E-22 | postive |
| MAFG | LINC02561 | 0.447086917 | 5.43E-26 | postive |
| G6PD | LINC02561 | 0.425310858 | 1.99E-23 | postive |
| PGD | LINC02561 | 0.486495367 | 3.97E-31 | postive |
| ABCC1 | LINC02561 | 0.511063281 | 1.10E-34 | postive |
| IDH1 | LINC02561 | 0.451644157 | 1.50E-26 | postive |
| GOT1 | AC016866.1 | 0.401769797 | 7.39E-21 | postive |
| ATF4 | TIPARP-AS1 | 0.421786277 | 4.98E-23 | postive |
| DDIT3 | TIPARP-AS1 | 0.411933109 | 6.09E-22 | postive |
| TAZ | TIPARP-AS1 | 0.457854969 | 2.50E-27 | postive |
| PHKG2 | AC026304.1 | 0.421900068 | 4.83E-23 | postive |
| EGLN2 | AC026304.1 | 0.469382094 | 8.15E-29 | postive |
| ISCU | AL390719.2 | 0.423644843 | 3.07E-23 | postive |
| STMN1 | AL390719.2 | 0.421883553 | 4.85E-23 | postive |
| NOX1 | AL390719.2 | 0.463769392 | 4.38E-28 | postive |
| PHKG2 | AL390719.2 | 0.56782965 | 4.17E-44 | postive |
| PEBP1 | AL390719.2 | 0.400218047 | 1.07E-20 | postive |
| MYB | AL390719.2 | 0.400917683 | 9.08E-21 | postive |
| ANO6 | AL390719.2 | -0.408139739 | 1.56E-21 | negative |
| TAZ | AL390719.2 | 0.473480327 | 2.34E-29 | postive |
| SLC2A8 | AC023302.1 | 0.433267394 | 2.42E-24 | postive |
| PHKG2 | AC023302.1 | 0.583685429 | 4.45E-47 | postive |
| ANO6 | AC023302.1 | -0.403916873 | 4.39E-21 | negative |
| EGLN2 | AC023302.1 | 0.441196309 | 2.80E-25 | postive |
| TAZ | AC023302.1 | 0.541592784 | 1.58E-39 | postive |
| MUC1 | AC025154.2 | 0.546422445 | 2.43E-40 | postive |
| CAV1 | LINC02454 | 0.401732175 | 7.46E-21 | postive |
| DDIT4 | AC084117.1 | 0.514297452 | 3.56E-35 | postive |
| HELLS | AC024075.1 | 0.410947041 | 7.78E-22 | postive |
| MTOR | AC024075.1 | 0.421894527 | 4.84E-23 | postive |
| BRD4 | AC024075.1 | 0.464271314 | 3.78E-28 | postive |
| KLHL24 | AC024075.1 | 0.422215297 | 4.45E-23 | postive |
| ZFP69B | AC024075.1 | 0.409963697 | 9.94E-22 | postive |
| SETD1B | AC024075.1 | 0.557001831 | 3.62E-42 | postive |
| IREB2 | AC024075.1 | 0.478811387 | 4.50E-30 | postive |
| SP1 | AC024075.1 | 0.649134374 | 2.87E-61 | postive |
| CS | AC024075.1 | 0.412255309 | 5.61E-22 | postive |
| GLS2 | AC024075.1 | 0.449157557 | 3.03E-26 | postive |
| NCOA4 | AC024075.1 | 0.402920177 | 5.60E-21 | postive |
| MAPK8 | AC024075.1 | 0.402249639 | 6.58E-21 | postive |
| ACVR1B | AC024075.1 | 0.494986042 | 2.52E-32 | postive |
| ATM | AC024075.1 | 0.574604676 | 2.34E-45 | postive |
| SIRT1 | AC024075.1 | 0.462620429 | 6.16E-28 | postive |
| HILPDA | MIR210HG | 0.415605709 | 2.42E-22 | postive |
| IFNG | HLA-DQB1-AS1 | 0.468110992 | 1.20E-28 | postive |
| GPX4 | NAPA-AS1 | 0.506153943 | 5.96E-34 | postive |
| FANCD2 | SH3BP5-AS1 | 0.502260982 | 2.23E-33 | postive |
| HELLS | SH3BP5-AS1 | 0.525384671 | 6.77E-37 | postive |
| ISCU | SH3BP5-AS1 | 0.478127962 | 5.57E-30 | postive |
| TUBE1 | SH3BP5-AS1 | 0.447806484 | 4.44E-26 | postive |
| STMN1 | SH3BP5-AS1 | 0.42230975 | 4.35E-23 | postive |
| GLS2 | SH3BP5-AS1 | 0.45670495 | 3.49E-27 | postive |
| MYB | SH3BP5-AS1 | 0.491886497 | 6.96E-32 | postive |
| MAPK8 | SH3BP5-AS1 | 0.434001222 | 1.99E-24 | postive |
| LPIN1 | SH3BP5-AS1 | 0.462205523 | 6.97E-28 | postive |
| CAV1 | AC245041.1 | 0.431407781 | 3.98E-24 | postive |
| ANO6 | AC245041.1 | 0.440455955 | 3.43E-25 | postive |
| ISCU | AC124798.1 | 0.401817654 | 7.31E-21 | postive |
| ELAVL1 | AC124798.1 | 0.423277709 | 3.38E-23 | postive |
| GLS2 | AC124798.1 | 0.433362238 | 2.36E-24 | postive |
| ATG4D | AC124798.1 | 0.40328408 | 5.12E-21 | postive |
| LONP1 | AC124798.1 | 0.430866029 | 4.60E-24 | postive |
| MTOR | AL162595.1 | 0.409011854 | 1.26E-21 | postive |
| SP1 | AL162595.1 | 0.49380651 | 3.71E-32 | postive |
| MAPK8 | AL162595.1 | 0.426745572 | 1.37E-23 | postive |
| YY1AP1 | AL162595.1 | 0.412415405 | 5.39E-22 | postive |
| GPX4 | ZSCAN16-AS1 | 0.434004621 | 1.98E-24 | postive |
| CHMP6 | ZSCAN16-AS1 | 0.456562414 | 3.63E-27 | postive |
| RPL8 | ZSCAN16-AS1 | 0.453951189 | 7.72E-27 | postive |
| PHKG2 | ZSCAN16-AS1 | 0.561158506 | 6.65E-43 | postive |
| EGLN2 | ZSCAN16-AS1 | 0.649893026 | 1.87E-61 | postive |
| STMN1 | PRKAG2-AS1 | 0.447587157 | 4.72E-26 | postive |
| ATF4 | AC092171.4 | 0.42871105 | 8.15E-24 | postive |
| ISCU | AC092171.4 | 0.432728696 | 2.80E-24 | postive |
| STMN1 | AC092171.4 | 0.430292721 | 5.36E-24 | postive |
| NOX1 | AC092171.4 | 0.425351146 | 1.97E-23 | postive |
| PHKG2 | AC092171.4 | 0.574981646 | 1.99E-45 | postive |
| MAP1LC3A | AC092171.4 | 0.46800092 | 1.24E-28 | postive |
| PEBP1 | AC092171.4 | 0.420593871 | 6.77E-23 | postive |
| TAZ | AC092171.4 | 0.559706157 | 1.20E-42 | postive |
| PML | AL357054.4 | 0.492470174 | 5.75E-32 | postive |
| PHKG2 | ARHGAP27P1-BPTFP1-KPNA2P3 | 0.413598141 | 4.01E-22 | postive |
| GPX4 | AC120053.1 | 0.411554016 | 6.69E-22 | postive |
| ISCU | AC120053.1 | 0.469443659 | 8.00E-29 | postive |
| ACSF2 | AC120053.1 | 0.430739302 | 4.76E-24 | postive |
| STMN1 | AC120053.1 | 0.455499269 | 4.94E-27 | postive |
| NOX1 | AC120053.1 | 0.524010934 | 1.11E-36 | postive |
| PHKG2 | AC120053.1 | 0.539699892 | 3.25E-39 | postive |
| TAZ | AC120053.1 | 0.506649937 | 5.03E-34 | postive |
| GPX4 | AL049840.4 | -0.414663517 | 3.07E-22 | negative |
| PHKG2 | AL049840.4 | -0.428831338 | 7.89E-24 | negative |
| MT3 | AL158166.1 | 0.488332518 | 2.20E-31 | postive |
| CDO1 | AL158166.1 | 0.632924125 | 1.96E-57 | postive |
| HELLS | SOS1-IT1 | 0.426298598 | 1.54E-23 | postive |
| FADS2 | SOS1-IT1 | 0.413025736 | 4.63E-22 | postive |
| ISCU | SOS1-IT1 | 0.444550766 | 1.11E-25 | postive |
| TUBE1 | SOS1-IT1 | 0.455545331 | 4.88E-27 | postive |
| ELAVL1 | SOS1-IT1 | 0.429275876 | 7.01E-24 | postive |
| GABPB1 | SOS1-IT1 | 0.415002743 | 2.81E-22 | postive |
| GLS2 | SOS1-IT1 | 0.440222917 | 3.66E-25 | postive |
| LPIN1 | SOS1-IT1 | 0.485901252 | 4.80E-31 | postive |
| SIRT1 | SOS1-IT1 | 0.404555158 | 3.76E-21 | postive |
| AIFM2 | AL365181.2 | 0.435834773 | 1.21E-24 | postive |
| GPX2 | AL365181.2 | 0.409645504 | 1.08E-21 | postive |
| TUBE1 | AL133338.1 | 0.416859975 | 1.76E-22 | postive |
| HSF1 | PVT1 | 0.417329 | 1.56E-22 | postive |
| ATF4 | PVT1 | 0.469592583 | 7.65E-29 | postive |
| PRDX6 | PVT1 | 0.464663701 | 3.36E-28 | postive |
| RPL8 | PVT1 | 0.484941916 | 6.51E-31 | postive |
| FANCD2 | AL136531.1 | 0.400055882 | 1.12E-20 | postive |
| HELLS | AL136531.1 | 0.440439175 | 3.45E-25 | postive |
| NOX1 | AL354920.1 | 0.401970609 | 7.04E-21 | postive |
| MTOR | AC018752.1 | 0.401770657 | 7.39E-21 | postive |
| SP1 | AC018752.1 | 0.417335047 | 1.56E-22 | postive |
| ATM | AC018752.1 | 0.414947001 | 2.85E-22 | postive |
| FANCD2 | AC127024.5 | 0.428427264 | 8.78E-24 | postive |
| ATF4 | AC127024.5 | 0.415728181 | 2.34E-22 | postive |
| HELLS | AC127024.5 | 0.454646347 | 6.32E-27 | postive |
| ISCU | AC127024.5 | 0.434700463 | 1.65E-24 | postive |
| ZNF419 | AC127024.5 | 0.468189043 | 1.17E-28 | postive |
| TUBE1 | AC127024.5 | 0.527901532 | 2.70E-37 | postive |
| ELAVL1 | AC127024.5 | 0.453277557 | 9.37E-27 | postive |
| STMN1 | AC127024.5 | 0.446601726 | 6.23E-26 | postive |
| GABPB1 | AC127024.5 | 0.453400959 | 9.05E-27 | postive |
| PHKG2 | AC127024.5 | 0.48536584 | 5.69E-31 | postive |
| TAZ | AC127024.5 | 0.429704448 | 6.26E-24 | postive |
| MIOX | AC078881.1 | 0.411128257 | 7.44E-22 | postive |
| ZEB1 | STARD4-AS1 | 0.421817206 | 4.94E-23 | postive |
| KRAS | AC084819.1 | 0.690536964 | 3.25E-72 | postive |
| GLS2 | AC008443.4 | 0.536306074 | 1.18E-38 | postive |
| MIOX | AC008443.4 | 0.403878013 | 4.43E-21 | postive |
| GPX4 | AC084824.6 | 0.502676722 | 1.94E-33 | postive |
| CISD1 | AC084824.6 | 0.405337876 | 3.11E-21 | postive |
| ATF4 | AC084824.6 | 0.475193867 | 1.38E-29 | postive |
| ISCU | AC084824.6 | 0.539499867 | 3.51E-39 | postive |
| ACSF2 | AC084824.6 | 0.412632513 | 5.11E-22 | postive |
| STMN1 | AC084824.6 | 0.463362641 | 4.95E-28 | postive |
| NOX1 | AC084824.6 | 0.494660844 | 2.81E-32 | postive |
| PHKG2 | AC084824.6 | 0.556335159 | 4.74E-42 | postive |
| SOCS1 | AC084824.6 | 0.457482661 | 2.78E-27 | postive |
| MYB | AC084824.6 | 0.476580375 | 9.00E-30 | postive |
| EGLN2 | AC084824.6 | 0.46168994 | 8.11E-28 | postive |
| TAZ | AC084824.6 | 0.538668945 | 4.82E-39 | postive |
| GPX4 | MAFG-DT | 0.480104565 | 3.00E-30 | postive |
| AIFM2 | MAFG-DT | 0.412257892 | 5.61E-22 | postive |
| ELAVL1 | MAFG-DT | 0.428436113 | 8.76E-24 | postive |
| PHKG2 | MAFG-DT | 0.47138871 | 4.43E-29 | postive |
| MAP1LC3A | MAFG-DT | 0.472808493 | 2.88E-29 | postive |
| PEBP1 | MAFG-DT | 0.418973707 | 1.03E-22 | postive |
| SLC7A11 | WNT5A-AS1 | 0.582954718 | 6.14E-47 | postive |
| AKR1C1 | WNT5A-AS1 | 0.457834763 | 2.51E-27 | postive |
| AKR1C3 | WNT5A-AS1 | 0.463957754 | 4.15E-28 | postive |
| NQO1 | WNT5A-AS1 | 0.452581366 | 1.14E-26 | postive |
| TXNRD1 | WNT5A-AS1 | 0.582258279 | 8.36E-47 | postive |
| SRXN1 | WNT5A-AS1 | 0.719634668 | 4.40E-81 | postive |
| FTL | WNT5A-AS1 | 0.431821723 | 3.56E-24 | postive |
| MAFG | WNT5A-AS1 | 0.464196511 | 3.86E-28 | postive |
| G6PD | WNT5A-AS1 | 0.610036316 | 2.15E-52 | postive |
| PGD | WNT5A-AS1 | 0.596495516 | 1.33E-49 | postive |
| ABCC1 | WNT5A-AS1 | 0.633489097 | 1.45E-57 | postive |
| IDH1 | WNT5A-AS1 | 0.451052259 | 1.77E-26 | postive |
| HELLS | AC060780.1 | 0.546102712 | 2.75E-40 | postive |
| FADS2 | AC060780.1 | 0.439285901 | 4.73E-25 | postive |
| KLHL24 | AC060780.1 | 0.508937921 | 2.29E-34 | postive |
| TUBE1 | AC060780.1 | 0.561062609 | 6.91E-43 | postive |
| ELAVL1 | AC060780.1 | 0.441652645 | 2.47E-25 | postive |
| SP1 | AC060780.1 | 0.435155968 | 1.46E-24 | postive |
| GABPB1 | AC060780.1 | 0.431288446 | 4.11E-24 | postive |
| GLS2 | AC060780.1 | 0.542523046 | 1.10E-39 | postive |
| ULK2 | AC060780.1 | 0.403647198 | 4.69E-21 | postive |
| MYB | AC060780.1 | 0.42996986 | 5.84E-24 | postive |
| MAPK8 | AC060780.1 | 0.44239107 | 2.01E-25 | postive |
| ACVR1B | AC060780.1 | 0.458573581 | 2.02E-27 | postive |
| LPIN1 | AC060780.1 | 0.43900267 | 5.11E-25 | postive |
| ATM | AC060780.1 | 0.426576844 | 1.43E-23 | postive |
| YY1AP1 | AC060780.1 | 0.478870971 | 4.42E-30 | postive |
| SIRT1 | AC060780.1 | 0.482491271 | 1.42E-30 | postive |
| NFE2L2 | LINC01521 | 0.457810951 | 2.53E-27 | postive |
| FADS2 | LINC01521 | 0.454547392 | 6.51E-27 | postive |
| BRD4 | LINC01521 | 0.533840758 | 2.98E-38 | postive |
| NF2 | LINC01521 | 0.400466104 | 1.01E-20 | postive |
| KLHL24 | LINC01521 | 0.618663798 | 3.03E-54 | postive |
| ZFP69B | LINC01521 | 0.455528625 | 4.90E-27 | postive |
| TUBE1 | LINC01521 | 0.406950549 | 2.09E-21 | postive |
| SETD1B | LINC01521 | 0.469648914 | 7.52E-29 | postive |
| TFRC | LINC01521 | 0.451602263 | 1.51E-26 | postive |
| MAFG | LINC01521 | 0.429169921 | 7.21E-24 | postive |
| EIF2AK4 | LINC01521 | 0.406873806 | 2.13E-21 | postive |
| IREB2 | LINC01521 | 0.509413543 | 1.95E-34 | postive |
| ELAVL1 | LINC01521 | 0.463718076 | 4.45E-28 | postive |
| SP1 | LINC01521 | 0.504135441 | 1.19E-33 | postive |
| PIK3CA | LINC01521 | 0.531258336 | 7.81E-38 | postive |
| LPCAT3 | LINC01521 | 0.464915322 | 3.12E-28 | postive |
| GLS2 | LINC01521 | 0.409675007 | 1.07E-21 | postive |
| ABCC1 | LINC01521 | 0.467951179 | 1.26E-28 | postive |
| ACVR1B | LINC01521 | 0.420313751 | 7.27E-23 | postive |
| LPIN1 | LINC01521 | 0.413212193 | 4.42E-22 | postive |
| ATM | LINC01521 | 0.493023825 | 4.80E-32 | postive |
| YY1AP1 | LINC01521 | 0.402895455 | 5.63E-21 | postive |
| IDH1 | LINC01521 | 0.407350963 | 1.90E-21 | postive |
| SIRT1 | LINC01521 | 0.422344365 | 4.31E-23 | postive |
| KLHL24 | ZEB1-AS1 | 0.452523347 | 1.16E-26 | postive |
| GABARAPL1 | ZEB1-AS1 | 0.417008276 | 1.69E-22 | postive |
| CISD1 | LIPE-AS1 | 0.419834859 | 8.22E-23 | postive |
| ANO6 | LIPE-AS1 | -0.400959349 | 8.99E-21 | negative |
| EGLN2 | LIPE-AS1 | 0.417771475 | 1.39E-22 | postive |
| FANCD2 | LINC00909 | 0.412190592 | 5.71E-22 | postive |
| TP53 | LINC00909 | 0.406684101 | 2.23E-21 | postive |
| HELLS | LINC00909 | 0.479514848 | 3.61E-30 | postive |
| ISCU | LINC00909 | 0.479272764 | 3.90E-30 | postive |
| STMN1 | LINC00909 | 0.504072375 | 1.21E-33 | postive |
| NOX1 | LINC00909 | 0.481282034 | 2.07E-30 | postive |
| MYB | LINC00909 | 0.449932505 | 2.43E-26 | postive |
| NGB | AL355601.1 | 0.469032225 | 9.06E-29 | postive |
| ALOX5 | AC002091.1 | 0.449330663 | 2.89E-26 | postive |
| CYBB | AC002091.1 | 0.429036031 | 7.47E-24 | postive |
| HELLS | TBC1D8-AS1 | 0.44900711 | 3.16E-26 | postive |
| KLHL24 | TBC1D8-AS1 | 0.470867493 | 5.20E-29 | postive |
| TUBE1 | TBC1D8-AS1 | 0.419457374 | 9.06E-23 | postive |
| ELAVL1 | TBC1D8-AS1 | 0.428478841 | 8.66E-24 | postive |
| GLS2 | TBC1D8-AS1 | 0.411776287 | 6.33E-22 | postive |
| KEAP1 | TBC1D8-AS1 | 0.413120512 | 4.52E-22 | postive |
| YY1AP1 | TBC1D8-AS1 | 0.430103601 | 5.63E-24 | postive |
| NOX4 | LINC01050 | 0.451398998 | 1.60E-26 | postive |
| SNX4 | LINC02035 | 0.506605911 | 5.11E-34 | postive |
| ACVR1B | LINC02035 | 0.433403064 | 2.33E-24 | postive |
| NNMT | AC093673.1 | 0.400607148 | 9.78E-21 | postive |
| ACVR1B | AC093110.1 | 0.415066968 | 2.77E-22 | postive |
| ATM | AC093110.1 | 0.402488 | 6.21E-21 | postive |
| ISCU | AP003774.2 | 0.477098324 | 7.67E-30 | postive |
| ALOX5 | AP003774.2 | 0.416228522 | 2.06E-22 | postive |
| FLT3 | AP003774.2 | 0.5702806 | 1.48E-44 | postive |
| IFNG | AP003774.2 | 0.514849339 | 2.93E-35 | postive |
| MIOX | AC106875.1 | 0.453428814 | 8.97E-27 | postive |
| TUBE1 | AC068888.1 | 0.534989078 | 1.94E-38 | postive |
| ELAVL1 | AC068888.1 | 0.441119171 | 2.86E-25 | postive |
| GABPB1 | AC068888.1 | 0.418611501 | 1.13E-22 | postive |
| GLS2 | AC068888.1 | 0.41952894 | 8.90E-23 | postive |
| GABARAPL1 | AC068888.1 | 0.433068046 | 2.55E-24 | postive |
| YY1AP1 | AC068888.1 | 0.443206854 | 1.61E-25 | postive |
| SRC | NORAD | 0.495339531 | 2.24E-32 | postive |
| NFS1 | NORAD | 0.64406477 | 4.80E-60 | postive |
| NFE2L2 | BX293535.1 | 0.427167416 | 1.22E-23 | postive |
| GPX2 | BX293535.1 | 0.412502725 | 5.28E-22 | postive |
| BID | BX293535.1 | 0.435934097 | 1.18E-24 | postive |
| FH | EXOSC10-AS1 | 0.402145467 | 6.75E-21 | postive |
| NOX1 | EXOSC10-AS1 | 0.410893068 | 7.89E-22 | postive |
| PHKG2 | EXOSC10-AS1 | 0.435192976 | 1.44E-24 | postive |
| PEBP1 | EXOSC10-AS1 | 0.415867235 | 2.26E-22 | postive |
| TUBE1 | AC020558.2 | 0.547331809 | 1.70E-40 | postive |
| TAZ | AC020558.2 | 0.441038265 | 2.92E-25 | postive |
| AIFM2 | PCCA-DT | 0.408969376 | 1.27E-21 | postive |
| ELAVL1 | PCCA-DT | 0.432651219 | 2.85E-24 | postive |
| NOX1 | PCCA-DT | 0.414091854 | 3.54E-22 | postive |
| ATG4D | PCCA-DT | 0.432025732 | 3.37E-24 | postive |
| BID | PCCA-DT | 0.53546873 | 1.62E-38 | postive |
| YY1AP1 | PCCA-DT | 0.416180466 | 2.09E-22 | postive |
| HELLS | AC005332.3 | 0.42715793 | 1.23E-23 | postive |
| ISCU | AC005332.3 | 0.476199398 | 1.01E-29 | postive |
| STMN1 | AC005332.3 | 0.499908042 | 4.92E-33 | postive |
| NOX1 | AC005332.3 | 0.453942631 | 7.74E-27 | postive |
| PHKG2 | AC005332.3 | 0.417069347 | 1.67E-22 | postive |
| PEBP1 | AC005332.3 | 0.432201555 | 3.22E-24 | postive |
| TAZ | AC005332.3 | 0.417783167 | 1.39E-22 | postive |
| WIPI2 | AP001065.3 | 0.406091373 | 2.58E-21 | postive |
| GPX4 | AC006942.1 | 0.468713588 | 9.98E-29 | postive |
| ISCU | AC006942.1 | 0.459320141 | 1.63E-27 | postive |
| PHKG2 | AC006942.1 | 0.499669831 | 5.33E-33 | postive |
| SOCS1 | AC006942.1 | 0.435307527 | 1.40E-24 | postive |
| EGLN2 | AC006942.1 | 0.459126251 | 1.72E-27 | postive |
| TAZ | AC006942.1 | 0.454488892 | 6.62E-27 | postive |
| GPX4 | SNHG32 | 0.444856789 | 1.01E-25 | postive |
| ATF4 | SNHG32 | 0.46592443 | 2.31E-28 | postive |
| ISCU | SNHG32 | 0.406839215 | 2.15E-21 | postive |
| STMN1 | SNHG32 | 0.42026723 | 7.36E-23 | postive |
| TAZ | AC118553.1 | 0.427798784 | 1.04E-23 | postive |
| GPX4 | AC073896.4 | 0.607221282 | 8.39E-52 | postive |
| CISD1 | AC073896.4 | 0.429815413 | 6.08E-24 | postive |
| ATF4 | AC073896.4 | 0.408891666 | 1.30E-21 | postive |
| ISCU | AC073896.4 | 0.416133402 | 2.11E-22 | postive |
| HIF1A | AC073896.4 | -0.416417618 | 1.97E-22 | negative |
| CHMP6 | AC073896.4 | 0.453938078 | 7.75E-27 | postive |
| RPL8 | AC073896.4 | 0.420298332 | 7.30E-23 | postive |
| SLC2A8 | AC073896.4 | 0.509350264 | 1.99E-34 | postive |
| ACSF2 | AC073896.4 | 0.4103902 | 8.94E-22 | postive |
| STMN1 | AC073896.4 | 0.401473296 | 7.94E-21 | postive |
| NOX1 | AC073896.4 | 0.472696514 | 2.98E-29 | postive |
| HRAS | AC073896.4 | 0.449058279 | 3.12E-26 | postive |
| PHKG2 | AC073896.4 | 0.715766148 | 7.70E-80 | postive |
| MAP1LC3A | AC073896.4 | 0.532808965 | 4.39E-38 | postive |
| PEBP1 | AC073896.4 | 0.453634383 | 8.46E-27 | postive |
| ANO6 | AC073896.4 | -0.416478209 | 1.94E-22 | negative |
| EGLN2 | AC073896.4 | 0.696586429 | 5.70E-74 | postive |
| TAZ | AC073896.4 | 0.5379 | 6.46E-39 | postive |
| MTDH | AC073896.4 | -0.444210179 | 1.22E-25 | negative |
| GPX4 | AC025265.1 | 0.454348094 | 6.89E-27 | postive |
| ISCU | AC025265.1 | 0.444215092 | 1.21E-25 | postive |
| TUBE1 | AC025265.1 | 0.482823587 | 1.28E-30 | postive |
| ELAVL1 | AC025265.1 | 0.432967758 | 2.62E-24 | postive |
| STMN1 | AC025265.1 | 0.435182524 | 1.44E-24 | postive |
| NOX1 | AC025265.1 | 0.403109583 | 5.34E-21 | postive |
| PHKG2 | AC025265.1 | 0.41062186 | 8.44E-22 | postive |
| SOCS1 | AC025265.1 | 0.458128889 | 2.30E-27 | postive |
| MYB | AC025265.1 | 0.520055351 | 4.63E-36 | postive |
| ATF4 | SCAMP1-AS1 | 0.403643245 | 4.70E-21 | postive |
| GPX4 | AC011445.2 | 0.436629081 | 9.76E-25 | postive |
| RPL8 | AC011445.2 | 0.506129047 | 6.01E-34 | postive |
| SLC2A8 | AC011445.2 | 0.421326885 | 5.60E-23 | postive |
| PHKG2 | AC011445.2 | 0.495315897 | 2.26E-32 | postive |
| EGLN2 | AC011445.2 | 0.603069059 | 6.11E-51 | postive |
| HELLS | AC024075.2 | 0.505679697 | 7.01E-34 | postive |
| ISCU | AC024075.2 | 0.503146247 | 1.66E-33 | postive |
| ELAVL1 | AC024075.2 | 0.424780179 | 2.29E-23 | postive |
| STMN1 | AC024075.2 | 0.437144444 | 8.49E-25 | postive |
| NOX1 | AC024075.2 | 0.476576977 | 9.01E-30 | postive |
| PHKG2 | AC024075.2 | 0.428849407 | 7.85E-24 | postive |
| ATG4D | AC024075.2 | 0.470690328 | 5.48E-29 | postive |
| MYB | AC024075.2 | 0.478245565 | 5.37E-30 | postive |
| TAZ | AC024075.2 | 0.411779087 | 6.32E-22 | postive |
| GABPB1 | AC083843.3 | 0.407277571 | 1.93E-21 | postive |
| EMC2 | AC083843.3 | 0.41545177 | 2.51E-22 | postive |
| FANCD2 | AL356481.3 | 0.44199736 | 2.24E-25 | postive |
| HELLS | AL356481.3 | 0.545982175 | 2.88E-40 | postive |
| STMN1 | AL356481.3 | 0.490283955 | 1.17E-31 | postive |
| MAPK8 | AL356481.3 | 0.427412444 | 1.15E-23 | postive |
| ISCU | AC026367.3 | 0.466507367 | 1.94E-28 | postive |
| GOT1 | AC026367.3 | 0.410522671 | 8.65E-22 | postive |
| PHKG2 | AL596094.1 | 0.455690567 | 4.68E-27 | postive |
| TUBE1 | AC090589.3 | 0.434856645 | 1.58E-24 | postive |
| HELLS | AC008982.2 | 0.42269897 | 3.93E-23 | postive |
| SETD1B | AC008982.2 | 0.449996287 | 2.39E-26 | postive |
| SP1 | AC008982.2 | 0.435994515 | 1.16E-24 | postive |
| TUBE1 | ZNF32-AS2 | 0.471665555 | 4.08E-29 | postive |
| GABPB1 | ZNF32-AS2 | 0.431089303 | 4.33E-24 | postive |
| MAPK8 | ZNF32-AS2 | 0.43074968 | 4.74E-24 | postive |
| DDIT3 | U62317.4 | 0.488926133 | 1.82E-31 | postive |
| HRAS | U62317.4 | 0.405628337 | 2.89E-21 | postive |
| EGLN2 | U62317.4 | 0.480388447 | 2.75E-30 | postive |
| TUBE1 | LINC02604 | 0.439257879 | 4.77E-25 | postive |
| TAZ | LINC02604 | 0.420774701 | 6.46E-23 | postive |
| FANCD2 | AL596202.1 | 0.49899588 | 6.68E-33 | postive |
| HELLS | AL596202.1 | 0.58188709 | 9.84E-47 | postive |
| FADS2 | AL596202.1 | 0.420754847 | 6.49E-23 | postive |
| ISCU | AL596202.1 | 0.406667108 | 2.24E-21 | postive |
| TUBE1 | AL596202.1 | 0.598383795 | 5.54E-50 | postive |
| HMGB1 | AL596202.1 | 0.430931335 | 4.52E-24 | postive |
| ELAVL1 | AL596202.1 | 0.544058195 | 6.09E-40 | postive |
| STMN1 | AL596202.1 | 0.511838436 | 8.40E-35 | postive |
| GABPB1 | AL596202.1 | 0.468953482 | 9.28E-29 | postive |
| NOX1 | AL596202.1 | 0.411574645 | 6.66E-22 | postive |
| GLS2 | AL596202.1 | 0.441227217 | 2.78E-25 | postive |
| ATG4D | AL596202.1 | 0.406773147 | 2.19E-21 | postive |
| MYB | AL596202.1 | 0.455368176 | 5.13E-27 | postive |
| MIOX | LINC02244 | 0.47965771 | 3.45E-30 | postive |
| CAPG | TP53TG1 | 0.420925813 | 6.21E-23 | postive |
| NOX1 | TP53TG1 | 0.400268813 | 1.06E-20 | postive |
| EGLN2 | AC108047.1 | 0.413872219 | 3.74E-22 | postive |
| PHKG2 | AC027575.3 | 0.424123279 | 2.71E-23 | postive |
| BRD4 | U91328.1 | 0.476097972 | 1.05E-29 | postive |
| KLHL24 | U91328.1 | 0.425489389 | 1.90E-23 | postive |
| TUBE1 | U91328.1 | 0.431565539 | 3.82E-24 | postive |
| ELAVL1 | U91328.1 | 0.426541182 | 1.44E-23 | postive |
| YY1AP1 | U91328.1 | 0.408110382 | 1.57E-21 | postive |
| PHKG2 | AC130343.2 | 0.437286221 | 8.17E-25 | postive |
| EGLN2 | AC130343.2 | 0.451107607 | 1.74E-26 | postive |
| PHKG2 | AL590560.3 | 0.505991881 | 6.30E-34 | postive |
| TAZ | AL590560.3 | 0.416173254 | 2.09E-22 | postive |
| MYB | Z92544.1 | 0.444719969 | 1.05E-25 | postive |
| PHKG2 | AP002748.4 | 0.45832884 | 2.17E-27 | postive |
| MAP1LC3A | AP002748.4 | 0.406285139 | 2.46E-21 | postive |
| MIOX | AC108134.3 | 0.487933616 | 2.50E-31 | postive |
| GABARAPL2 | AC009113.1 | 0.413466626 | 4.14E-22 | postive |
| SOCS1 | PCAT19 | 0.429471189 | 6.66E-24 | postive |
| NNMT | AC012636.1 | 0.415935754 | 2.22E-22 | postive |
| NOX4 | AC012636.1 | 0.401689787 | 7.54E-21 | postive |
| ZEB1 | AC012636.1 | 0.614210985 | 2.78E-53 | postive |
| PRKAA2 | AC012636.1 | 0.585851006 | 1.70E-47 | postive |
| ISCU | CD27-AS1 | 0.413954287 | 3.67E-22 | postive |
| TUBE1 | CD27-AS1 | 0.427188675 | 1.22E-23 | postive |
| GABPB1 | CD27-AS1 | 0.425033993 | 2.14E-23 | postive |
| GABARAPL1 | CD27-AS1 | 0.451678237 | 1.48E-26 | postive |
| TAZ | CD27-AS1 | 0.459614729 | 1.49E-27 | postive |
| SLC2A8 | HYI-AS1 | 0.406724313 | 2.21E-21 | postive |
| HRAS | HYI-AS1 | 0.558495917 | 1.97E-42 | postive |
| PHKG2 | HYI-AS1 | 0.593956796 | 4.31E-49 | postive |
| MAPK1 | HYI-AS1 | -0.405487832 | 2.99E-21 | negative |
| ANO6 | HYI-AS1 | -0.411050587 | 7.58E-22 | negative |
| EGLN2 | HYI-AS1 | 0.643322727 | 7.22E-60 | postive |
| TAZ | HYI-AS1 | 0.411626934 | 6.57E-22 | postive |
| SLC40A1 | AL133371.2 | 0.51620609 | 1.82E-35 | postive |
| ENPP2 | AL133371.2 | 0.556285055 | 4.83E-42 | postive |
| HERPUD1 | AL133371.2 | 0.404619873 | 3.70E-21 | postive |
| ALOX5 | AL133371.2 | 0.602040684 | 9.94E-51 | postive |
| CYBB | AL133371.2 | 0.653727574 | 2.13E-62 | postive |
| FLT3 | AL133371.2 | 0.643872684 | 5.34E-60 | postive |
| TLR4 | AL133371.2 | 0.524407091 | 9.65E-37 | postive |
| HSPB1 | AC015912.3 | 0.432034463 | 3.37E-24 | postive |
| MTOR | AC015912.3 | -0.410605381 | 8.47E-22 | negative |
| RPL8 | AC015912.3 | 0.54999854 | 5.95E-41 | postive |
| HRAS | AC015912.3 | 0.429666638 | 6.32E-24 | postive |
| PHKG2 | AC015912.3 | 0.535924454 | 1.36E-38 | postive |
| ANO6 | AC015912.3 | -0.406931962 | 2.10E-21 | negative |
| EGLN2 | AC015912.3 | 0.595761457 | 1.87E-49 | postive |
| TAZ | AC015912.3 | 0.442998098 | 1.70E-25 | postive |
| PHKG2 | AC007497.1 | 0.404214469 | 4.09E-21 | postive |
| RPL8 | AP006621.3 | 0.41803551 | 1.30E-22 | postive |
| PHKG2 | AP006621.3 | 0.684330657 | 1.86E-70 | postive |
| EGLN2 | AP006621.3 | 0.561585362 | 5.58E-43 | postive |
| TAZ | AP006621.3 | 0.518411779 | 8.32E-36 | postive |
| LONP1 | AP006621.3 | 0.400166488 | 1.09E-20 | postive |
| RPL8 | ATP2A1-AS1 | 0.421103735 | 5.93E-23 | postive |
| SLC2A8 | ATP2A1-AS1 | 0.424386507 | 2.53E-23 | postive |
| STMN1 | ATP2A1-AS1 | 0.465467787 | 2.64E-28 | postive |
| PHKG2 | ATP2A1-AS1 | 0.586087367 | 1.53E-47 | postive |
| PEBP1 | ATP2A1-AS1 | 0.424850231 | 2.25E-23 | postive |
| EGLN2 | ATP2A1-AS1 | 0.475379539 | 1.30E-29 | postive |
| TAZ | ATP2A1-AS1 | 0.483164432 | 1.15E-30 | postive |
| PHKG2 | AC005476.2 | 0.401618213 | 7.67E-21 | postive |
| HELLS | AC013403.2 | 0.427047554 | 1.26E-23 | postive |
| ISCU | LINC01089 | 0.433760854 | 2.12E-24 | postive |
| SLC2A8 | LINC01089 | 0.431835445 | 3.55E-24 | postive |
| STMN1 | LINC01089 | 0.496610117 | 1.48E-32 | postive |
| PHKG2 | LINC01089 | 0.587610601 | 7.71E-48 | postive |
| PEBP1 | LINC01089 | 0.460523073 | 1.14E-27 | postive |
| SOCS1 | LINC01089 | 0.442693416 | 1.85E-25 | postive |
| MYB | LINC01089 | 0.482184722 | 1.56E-30 | postive |
| EGLN2 | LINC01089 | 0.43917102 | 4.88E-25 | postive |
| TAZ | LINC01089 | 0.487207224 | 3.16E-31 | postive |
| MAPK8 | PINK1-AS | 0.420859677 | 6.32E-23 | postive |
| ATM | PINK1-AS | 0.412778096 | 4.93E-22 | postive |
| ATF4 | AC004241.3 | 0.498415152 | 8.10E-33 | postive |
| PHKG2 | AC004241.3 | 0.495797084 | 1.93E-32 | postive |
| BID | LINC01752 | 0.418255205 | 1.23E-22 | postive |
| PHKG2 | AC002310.1 | 0.493447262 | 4.18E-32 | postive |
| TAZ | AC002310.1 | 0.441667214 | 2.46E-25 | postive |
| HIC1 | HECW2-AS1 | 0.41914721 | 9.81E-23 | postive |
| NOX4 | HECW2-AS1 | 0.489547754 | 1.49E-31 | postive |
| PHKG2 | AL355385.1 | 0.542091959 | 1.30E-39 | postive |
| ENPP2 | AC104083.1 | 0.421729837 | 5.05E-23 | postive |
| HIC1 | AC104083.1 | 0.613348593 | 4.25E-53 | postive |
| NOX4 | AC104083.1 | 0.571399674 | 9.22E-45 | postive |
| ZEB1 | AC104083.1 | 0.670656718 | 9.75E-67 | postive |
| TLR4 | AC104083.1 | 0.448544258 | 3.60E-26 | postive |
| EIF2S1 | KTN1-AS1 | 0.53197386 | 5.99E-38 | postive |
| GPX4 | AC087239.1 | 0.43416587 | 1.90E-24 | postive |
| PHKG2 | AC087239.1 | 0.578910513 | 3.63E-46 | postive |
| EGLN2 | AC087239.1 | 0.531401373 | 7.41E-38 | postive |
| TAZ | AC087239.1 | 0.456727709 | 3.46E-27 | postive |
| ATM | UGDH-AS1 | 0.446868267 | 5.78E-26 | postive |
| TUBE1 | AC006305.2 | 0.441970772 | 2.26E-25 | postive |
| SLC2A6 | VIM-AS1 | 0.411105697 | 7.48E-22 | postive |
| HELLS | AC004908.1 | 0.486030658 | 4.60E-31 | postive |
| TUBE1 | AC004908.1 | 0.443900905 | 1.32E-25 | postive |
| SP1 | AC004908.1 | 0.41898909 | 1.02E-22 | postive |
| GABPB1 | AC004908.1 | 0.412059332 | 5.90E-22 | postive |
| GLS2 | AC004908.1 | 0.460520206 | 1.14E-27 | postive |
| PHKG2 | AL590617.2 | 0.581790035 | 1.03E-46 | postive |
| EGLN2 | AL590617.2 | 0.542005652 | 1.34E-39 | postive |
| FANCD2 | DLEU2 | 0.411315822 | 7.10E-22 | postive |
| HELLS | DLEU2 | 0.47473505 | 1.59E-29 | postive |
| HMGB1 | DLEU2 | 0.531030101 | 8.50E-38 | postive |
| AURKA | DLEU2 | 0.450196745 | 2.26E-26 | postive |
| MIOX | AC092916.1 | 0.445994109 | 7.38E-26 | postive |
| HELLS | AL122125.1 | 0.423822041 | 2.94E-23 | postive |
| ISCU | AL122125.1 | 0.403215924 | 5.21E-21 | postive |
| PHKG2 | AL122125.1 | 0.408600558 | 1.39E-21 | postive |
| TAZ | AL122125.1 | 0.429322859 | 6.93E-24 | postive |
| ATF4 | AP003352.1 | 0.462698096 | 6.02E-28 | postive |
| RPL8 | AP003352.1 | 0.513698719 | 4.39E-35 | postive |
| PHKG2 | AP003352.1 | 0.590815031 | 1.81E-48 | postive |
| EGLN2 | AP003352.1 | 0.495065601 | 2.46E-32 | postive |
| TAZ | AP003352.1 | 0.563969818 | 2.08E-43 | postive |
| GPX4 | CAHM | 0.438460052 | 5.93E-25 | postive |
| PHKG2 | CAHM | 0.49440271 | 3.05E-32 | postive |
| EGLN2 | CAHM | 0.421929959 | 4.79E-23 | postive |
| TAZ | CAHM | 0.45578774 | 4.55E-27 | postive |
| PHKG2 | RTCA-AS1 | 0.47715225 | 7.54E-30 | postive |
| EGLN2 | RTCA-AS1 | 0.492954606 | 4.91E-32 | postive |
| NFE2L2 | AC073046.1 | 0.4268169 | 1.34E-23 | postive |
| MTOR | AC073046.1 | 0.45578288 | 4.55E-27 | postive |
| BRD4 | AC073046.1 | 0.484681439 | 7.08E-31 | postive |
| SETD1B | AC073046.1 | 0.613446898 | 4.05E-53 | postive |
| EIF2AK4 | AC073046.1 | 0.407951343 | 1.64E-21 | postive |
| IREB2 | AC073046.1 | 0.593734394 | 4.77E-49 | postive |
| SP1 | AC073046.1 | 0.538332672 | 5.48E-39 | postive |
| PIK3CA | AC073046.1 | 0.43747842 | 7.75E-25 | postive |
| LPCAT3 | AC073046.1 | 0.53659772 | 1.06E-38 | postive |
| SLC38A1 | AC073046.1 | 0.443478088 | 1.49E-25 | postive |
| MAPK1 | AC073046.1 | 0.439730246 | 4.19E-25 | postive |
| ATM | AC073046.1 | 0.460493386 | 1.15E-27 | postive |
| GPX4 | AL355353.2 | 0.415874471 | 2.26E-22 | postive |
| RPL8 | AL355353.2 | 0.474728434 | 1.59E-29 | postive |
| SLC2A8 | AL355353.2 | 0.432348044 | 3.10E-24 | postive |
| PHKG2 | AL355353.2 | 0.600593036 | 1.97E-50 | postive |
| EGLN2 | AL355353.2 | 0.695138783 | 1.51E-73 | postive |
| TAZ | AL355353.2 | 0.469595506 | 7.64E-29 | postive |
| NNMT | AC007998.3 | 0.428047089 | 9.71E-24 | postive |
| HIC1 | AC007998.3 | 0.46130603 | 9.08E-28 | postive |
| NOX4 | AC007998.3 | 0.454861408 | 5.94E-27 | postive |
| ZEB1 | AC007998.3 | 0.414655669 | 3.07E-22 | postive |
| CHMP6 | AL022341.1 | 0.466396065 | 2.00E-28 | postive |
| PHKG2 | AC012306.2 | 0.416622784 | 1.87E-22 | postive |
| MUC1 | AC073389.1 | 0.4052367 | 3.18E-21 | postive |
| PHKG2 | AC073389.1 | 0.443969653 | 1.30E-25 | postive |
| HELLS | AL603839.2 | 0.550857124 | 4.24E-41 | postive |
| ELAVL1 | AL603839.2 | 0.418853488 | 1.06E-22 | postive |
| STMN1 | AL603839.2 | 0.534848097 | 2.04E-38 | postive |
| GABPB1 | AL603839.2 | 0.415000008 | 2.82E-22 | postive |
| NOX1 | AL603839.2 | 0.404541847 | 3.77E-21 | postive |
| GLS2 | AL603839.2 | 0.404430792 | 3.88E-21 | postive |
| MAPK8 | AL603839.2 | 0.426071748 | 1.63E-23 | postive |
| KLHL24 | AC006206.2 | 0.441909568 | 2.30E-25 | postive |
| LPCAT3 | AC006206.2 | 0.480691517 | 2.50E-30 | postive |
| ACVR1B | AC006206.2 | 0.4056339 | 2.89E-21 | postive |
| FANCD2 | AC145207.5 | 0.421853278 | 4.89E-23 | postive |
| HELLS | AC145207.5 | 0.579419202 | 2.91E-46 | postive |
| ISCU | AC145207.5 | 0.403901651 | 4.41E-21 | postive |
| ELAVL1 | AC145207.5 | 0.461202896 | 9.36E-28 | postive |
| STMN1 | AC145207.5 | 0.469103044 | 8.87E-29 | postive |
| NOX1 | AC145207.5 | 0.50127378 | 3.11E-33 | postive |
| MYB | AC145207.5 | 0.403410331 | 4.97E-21 | postive |
| HELLS | AC015849.3 | 0.458986054 | 1.79E-27 | postive |
| TUBE1 | AC015849.3 | 0.412891438 | 4.79E-22 | postive |
| SETD1B | AC015849.3 | 0.419636391 | 8.65E-23 | postive |
| SP1 | AC015849.3 | 0.462248027 | 6.88E-28 | postive |
| ACVR1B | AC015849.3 | 0.460165582 | 1.27E-27 | postive |
| KLHL24 | TRAM2-AS1 | 0.408222327 | 1.53E-21 | postive |
| TUBE1 | TRAM2-AS1 | 0.418530359 | 1.15E-22 | postive |
| GABPB1 | TRAM2-AS1 | 0.422395708 | 4.25E-23 | postive |
| ATF4 | NUP50-DT | 0.407707026 | 1.74E-21 | postive |
| SELENOS | NUP50-DT | 0.437987914 | 6.75E-25 | postive |
| PHKG2 | NUP50-DT | 0.454355533 | 6.88E-27 | postive |
| EGLN2 | NUP50-DT | 0.427681543 | 1.07E-23 | postive |
| TAZ | NUP50-DT | 0.411484368 | 6.81E-22 | postive |
| GPX4 | AC008764.6 | 0.454357691 | 6.87E-27 | postive |
| CISD1 | AC008764.6 | 0.406481607 | 2.35E-21 | postive |
| FANCD2 | AC008764.6 | 0.401658465 | 7.59E-21 | postive |
| ATF4 | AC008764.6 | 0.438313552 | 6.17E-25 | postive |
| HELLS | AC008764.6 | 0.500961763 | 3.46E-33 | postive |
| ISCU | AC008764.6 | 0.556111301 | 5.18E-42 | postive |
| STMN1 | AC008764.6 | 0.551769905 | 2.95E-41 | postive |
| NOX1 | AC008764.6 | 0.50922417 | 2.08E-34 | postive |
| PHKG2 | AC008764.6 | 0.540552244 | 2.35E-39 | postive |
| ATG4D | AC008764.6 | 0.487437072 | 2.93E-31 | postive |
| SOCS1 | AC008764.6 | 0.446784565 | 5.92E-26 | postive |
| MYB | AC008764.6 | 0.44600315 | 7.37E-26 | postive |
| EGLN2 | AC008764.6 | 0.420417058 | 7.08E-23 | postive |
| TAZ | AC008764.6 | 0.530481648 | 1.04E-37 | postive |
| EMC2 | AP002907.1 | 0.422386106 | 4.26E-23 | postive |
| MIOX | AC128709.2 | 0.466568713 | 1.90E-28 | postive |
| FANCD2 | AC011477.2 | 0.404044545 | 4.26E-21 | postive |
| HELLS | AC011477.2 | 0.404529308 | 3.78E-21 | postive |
| KLHL24 | AC011477.2 | 0.42524613 | 2.03E-23 | postive |
| ELAVL1 | AC011477.2 | 0.41515723 | 2.71E-22 | postive |
| SP1 | AC011477.2 | 0.489541886 | 1.49E-31 | postive |
| GLS2 | AC011477.2 | 0.427896719 | 1.01E-23 | postive |
| MYB | AC011477.2 | 0.431000836 | 4.44E-24 | postive |
| ATM | AC011477.2 | 0.461891916 | 7.64E-28 | postive |
| PML | USP30-AS1 | 0.523697061 | 1.25E-36 | postive |
| CAPG | USP30-AS1 | 0.409471976 | 1.12E-21 | postive |
| IFNG | USP30-AS1 | 0.657692275 | 2.17E-63 | postive |
| HELLS | AC114810.1 | 0.457161027 | 3.05E-27 | postive |
| ISCU | AC114810.1 | 0.457688412 | 2.62E-27 | postive |
| NOX1 | AC114810.1 | 0.442003087 | 2.24E-25 | postive |
| GLS2 | AC114810.1 | 0.495632944 | 2.04E-32 | postive |
| GOT1 | AC114810.1 | 0.408645864 | 1.38E-21 | postive |
| STAT3 | AL731577.2 | 0.476373869 | 9.60E-30 | postive |
| MTOR | AL731577.2 | 0.516930532 | 1.41E-35 | postive |
| SETD1B | AL731577.2 | 0.513853879 | 4.16E-35 | postive |
| IREB2 | AL731577.2 | 0.480975392 | 2.28E-30 | postive |
| SP1 | AL731577.2 | 0.475600522 | 1.22E-29 | postive |
| HRAS | AL731577.2 | -0.481051925 | 2.23E-30 | negative |
| NCOA4 | AL731577.2 | 0.442666811 | 1.87E-25 | postive |
| PHKG2 | AL731577.2 | -0.450856844 | 1.87E-26 | negative |
| MAPK1 | AL731577.2 | 0.461629967 | 8.26E-28 | postive |
| ACVR1B | AL731577.2 | 0.407452335 | 1.85E-21 | postive |
| EPAS1 | AL731577.2 | 0.425352412 | 1.97E-23 | postive |
| SIRT1 | AL731577.2 | 0.528314619 | 2.32E-37 | postive |
| STMN1 | AC011450.1 | 0.409366061 | 1.15E-21 | postive |
| GLS2 | AC011450.1 | 0.402677365 | 5.94E-21 | postive |
| TUBE1 | GARS1-DT | 0.407923627 | 1.65E-21 | postive |
| WIPI2 | GARS1-DT | 0.401910363 | 7.15E-21 | postive |
| TAZ | GARS1-DT | 0.418491285 | 1.16E-22 | postive |
| HIC1 | AP003071.4 | 0.485311638 | 5.79E-31 | postive |
| ZEB1 | AP003071.4 | 0.406183245 | 2.53E-21 | postive |
| ALOX12B | AC138207.5 | 0.406232001 | 2.50E-21 | postive |
| HELLS | AC022306.2 | 0.418417308 | 1.18E-22 | postive |
| ISCU | AC022306.2 | 0.401841688 | 7.27E-21 | postive |
| STMN1 | AC022306.2 | 0.402567157 | 6.10E-21 | postive |
| NOX1 | AC022306.2 | 0.490688772 | 1.03E-31 | postive |
| GPX4 | AC109347.2 | 0.432275129 | 3.16E-24 | postive |
| HELLS | AC109347.2 | 0.401135432 | 8.61E-21 | postive |
| ISCU | AC109347.2 | 0.496883679 | 1.35E-32 | postive |
| STMN1 | AC109347.2 | 0.544424324 | 5.28E-40 | postive |
| NOX1 | AC109347.2 | 0.584089627 | 3.72E-47 | postive |
| ATG4D | AC109347.2 | 0.430426437 | 5.17E-24 | postive |
| TAZ | AC109347.2 | 0.402580572 | 6.08E-21 | postive |
| CISD2 | AC099850.2 | 0.51313744 | 5.34E-35 | postive |
| STMN1 | AC099850.2 | 0.449479244 | 2.77E-26 | postive |
| GABPB1 | AC090517.2 | 0.434233789 | 1.87E-24 | postive |
| ISCU | AC104825.1 | 0.524126261 | 1.07E-36 | postive |
| NOX1 | AC104825.1 | 0.431619512 | 3.76E-24 | postive |
| ATG4D | AC104825.1 | 0.447109601 | 5.40E-26 | postive |
| PHKG2 | AL135910.1 | 0.422383997 | 4.26E-23 | postive |
| MYB | AC084824.5 | 0.459395548 | 1.59E-27 | postive |
| TUBE1 | AC008870.2 | 0.426290597 | 1.54E-23 | postive |
| PHKG2 | AC008870.2 | 0.545774413 | 3.13E-40 | postive |
| TAZ | AC008870.2 | 0.474244096 | 1.85E-29 | postive |
| PHKG2 | AL031963.3 | 0.471187311 | 4.71E-29 | postive |
| KLHL24 | NNT-AS1 | 0.446575678 | 6.27E-26 | postive |
| PRKAA1 | NNT-AS1 | 0.504185563 | 1.17E-33 | postive |
| SIRT1 | NNT-AS1 | 0.438873929 | 5.30E-25 | postive |
| KRAS | AP005482.2 | 0.439481962 | 4.48E-25 | postive |
| NOX4 | LINC01929 | 0.543695994 | 7.00E-40 | postive |
| EGLN2 | CEBPA-DT | 0.421583476 | 5.24E-23 | postive |
| HELLS | SAP30-DT | 0.467230348 | 1.56E-28 | postive |
| CISD2 | SAP30-DT | 0.421204881 | 5.78E-23 | postive |
| ISCU | SAP30-DT | 0.44513671 | 9.38E-26 | postive |
| HMGB1 | SAP30-DT | 0.409707146 | 1.06E-21 | postive |
| STMN1 | SAP30-DT | 0.584551387 | 3.03E-47 | postive |
| NOX1 | SAP30-DT | 0.40264471 | 5.98E-21 | postive |
| PHKG2 | AC073195.2 | 0.442358277 | 2.03E-25 | postive |
| TAZ | AC073195.2 | 0.442175776 | 2.14E-25 | postive |
| GPX4 | AP000254.2 | 0.438294316 | 6.21E-25 | postive |
| HELLS | AP000254.2 | 0.408240748 | 1.52E-21 | postive |
| ISCU | AP000254.2 | 0.584689765 | 2.85E-47 | postive |
| HMGB1 | AP000254.2 | 0.413192413 | 4.44E-22 | postive |
| STMN1 | AP000254.2 | 0.488985754 | 1.78E-31 | postive |
| NOX1 | AP000254.2 | 0.551642844 | 3.10E-41 | postive |
| PHKG2 | AP000254.2 | 0.464144251 | 3.92E-28 | postive |
| ATG4D | AP000254.2 | 0.408491346 | 1.43E-21 | postive |
| PEBP1 | AP000254.2 | 0.430485147 | 5.09E-24 | postive |
| SOCS1 | AP000254.2 | 0.433332612 | 2.38E-24 | postive |
| MYB | AP000254.2 | 0.420670292 | 6.64E-23 | postive |
| TAZ | AP000254.2 | 0.404955476 | 3.41E-21 | postive |
| HELLS | AC007406.5 | 0.490732849 | 1.01E-31 | postive |
| KLHL24 | AC007406.5 | 0.429458815 | 6.68E-24 | postive |
| SP1 | AC007406.5 | 0.484251329 | 8.11E-31 | postive |
| LPCAT3 | AC007406.5 | 0.534199093 | 2.61E-38 | postive |
| MAPK8 | AC007406.5 | 0.460988975 | 9.97E-28 | postive |
| ACVR1B | AC007406.5 | 0.433935274 | 2.02E-24 | postive |
| SIRT1 | AC007406.5 | 0.445293884 | 8.98E-26 | postive |
| STMN1 | AC025048.2 | 0.489072062 | 1.73E-31 | postive |
| GPX4 | AC010226.1 | 0.41481646 | 2.95E-22 | postive |
| FANCD2 | AC010226.1 | 0.40744262 | 1.85E-21 | postive |
| HELLS | AC010226.1 | 0.431527828 | 3.85E-24 | postive |
| ISCU | AC010226.1 | 0.527279443 | 3.39E-37 | postive |
| STMN1 | AC010226.1 | 0.469961822 | 6.84E-29 | postive |
| MAP1LC3A | AC010226.1 | 0.410926785 | 7.82E-22 | postive |
| SOCS1 | AC010226.1 | 0.462794428 | 5.85E-28 | postive |
| MYB | AC010226.1 | 0.465095255 | 2.95E-28 | postive |
| SETD1B | AC018521.6 | 0.434989616 | 1.52E-24 | postive |
| SP1 | AC018521.6 | 0.451538045 | 1.54E-26 | postive |
| ACVR1B | AC018521.6 | 0.40173708 | 7.45E-21 | postive |
| TAZ | AL359513.1 | 0.478157413 | 5.52E-30 | postive |
| AKR1C1 | DLGAP1-AS2 | 0.417375217 | 1.54E-22 | postive |
| AKR1C3 | DLGAP1-AS2 | 0.445346003 | 8.85E-26 | postive |
| GCLC | DLGAP1-AS2 | 0.403261036 | 5.15E-21 | postive |
| AIFM2 | DLGAP1-AS2 | 0.572601111 | 5.52E-45 | postive |
| TXNRD1 | DLGAP1-AS2 | 0.451338851 | 1.63E-26 | postive |
| GPX2 | DLGAP1-AS2 | 0.502690365 | 1.93E-33 | postive |
| ASNS | DLGAP1-AS2 | 0.408317957 | 1.49E-21 | postive |
| TUBE1 | DLGAP1-AS2 | 0.489615767 | 1.45E-31 | postive |
| STAT3 | LINC02605 | 0.443838522 | 1.35E-25 | postive |
| GCH1 | LINC02605 | 0.527908422 | 2.69E-37 | postive |
| AGPAT3 | LINC02605 | 0.406864984 | 2.14E-21 | postive |
| MAPK8 | LINC02605 | 0.429804892 | 6.10E-24 | postive |
| FANCD2 | POLH-AS1 | 0.463063515 | 5.41E-28 | postive |
| HELLS | POLH-AS1 | 0.459686249 | 1.46E-27 | postive |
| ELAVL1 | POLH-AS1 | 0.423775632 | 2.97E-23 | postive |
| STMN1 | POLH-AS1 | 0.460114773 | 1.29E-27 | postive |
| PHKG2 | AC135178.6 | 0.526330198 | 4.79E-37 | postive |
| MAP1LC3A | AC135178.6 | 0.431057032 | 4.37E-24 | postive |
| FANCD2 | CKMT2-AS1 | 0.419711222 | 8.49E-23 | postive |
| HELLS | CKMT2-AS1 | 0.496863223 | 1.36E-32 | postive |
| ISCU | CKMT2-AS1 | 0.425039554 | 2.14E-23 | postive |
| ELAVL1 | CKMT2-AS1 | 0.41867453 | 1.11E-22 | postive |
| STMN1 | CKMT2-AS1 | 0.502544287 | 2.03E-33 | postive |
| GABPB1 | CKMT2-AS1 | 0.40855843 | 1.41E-21 | postive |
| GLS2 | CKMT2-AS1 | 0.440816941 | 3.11E-25 | postive |
| HELLS | AL358472.2 | 0.425376797 | 1.96E-23 | postive |
| ISCU | AL358472.2 | 0.432609057 | 2.89E-24 | postive |
| TUBE1 | AL358472.2 | 0.484285258 | 8.03E-31 | postive |
| ELAVL1 | AL358472.2 | 0.410731578 | 8.21E-22 | postive |
| STMN1 | AL358472.2 | 0.499357722 | 5.92E-33 | postive |
| GABPB1 | AL358472.2 | 0.428919391 | 7.71E-24 | postive |
| NOX1 | AL358472.2 | 0.460863423 | 1.03E-27 | postive |
| PHKG2 | AL358472.2 | 0.482760651 | 1.30E-30 | postive |
| PEBP1 | AL358472.2 | 0.409657303 | 1.07E-21 | postive |
| YY1AP1 | AL358472.2 | 0.484953251 | 6.49E-31 | postive |
| TAZ | AL358472.2 | 0.477420579 | 6.94E-30 | postive |
| NOX1 | LINC01290 | 0.406739713 | 2.20E-21 | postive |
| ACVR1B | ATP1B3-AS1 | 0.421496662 | 5.36E-23 | postive |
| STMN1 | ERICH6-AS1 | 0.428949993 | 7.65E-24 | postive |
| GPX4 | AC005261.3 | 0.42785211 | 1.02E-23 | postive |
| ISCU | AC005261.3 | 0.422034551 | 4.67E-23 | postive |
| ZNF419 | AC005261.3 | 0.545896928 | 2.98E-40 | postive |
| NOX1 | AC005261.3 | 0.483230519 | 1.12E-30 | postive |
| PHKG2 | AC005261.3 | 0.472072632 | 3.60E-29 | postive |
| EGLN2 | AC005261.3 | 0.416104283 | 2.13E-22 | postive |
| TAZ | AC005261.3 | 0.441943559 | 2.28E-25 | postive |
| STMN1 | AL512408.1 | 0.431688259 | 3.69E-24 | postive |
| YY1AP1 | AL512408.1 | 0.404082112 | 4.22E-21 | postive |
| HELLS | LINC00173 | 0.404146781 | 4.15E-21 | postive |
| MYB | LINC00173 | 0.446677325 | 6.10E-26 | postive |
| FANCD2 | AC027097.1 | 0.500149347 | 4.54E-33 | postive |
| TP53 | AC027097.1 | 0.454649084 | 6.32E-27 | postive |
| HELLS | AC027097.1 | 0.558865668 | 1.70E-42 | postive |
| ENPP2 | AC027097.1 | 0.408198802 | 1.54E-21 | postive |
| ISCU | AC027097.1 | 0.466069503 | 2.21E-28 | postive |
| HMGB1 | AC027097.1 | 0.452760244 | 1.09E-26 | postive |
| STMN1 | AC027097.1 | 0.474057421 | 1.96E-29 | postive |
| FLT3 | AC027097.1 | 0.522003894 | 2.30E-36 | postive |
| GLS2 | AC027097.1 | 0.496713828 | 1.43E-32 | postive |
| MYB | AC027097.1 | 0.475567532 | 1.23E-29 | postive |
| MAPK8 | AC027097.1 | 0.41739749 | 1.53E-22 | postive |
| ATM | AC027097.1 | 0.46223902 | 6.90E-28 | postive |
| SIRT1 | AC027097.1 | 0.401254296 | 8.37E-21 | postive |
| FANCD2 | AC037459.2 | 0.454084634 | 7.43E-27 | postive |
| HELLS | AC037459.2 | 0.491763781 | 7.24E-32 | postive |
| SETD1B | AC037459.2 | 0.431397002 | 3.99E-24 | postive |
| IREB2 | AC037459.2 | 0.406904828 | 2.12E-21 | postive |
| SP1 | AC037459.2 | 0.528520216 | 2.15E-37 | postive |
| ACVR1B | AC037459.2 | 0.426565845 | 1.43E-23 | postive |
| ATM | AC037459.2 | 0.446033093 | 7.30E-26 | postive |
| SLC2A8 | AL445222.2 | 0.435994023 | 1.16E-24 | postive |
| STMN1 | AL445222.2 | 0.403748817 | 4.58E-21 | postive |
| PHKG2 | AL445222.2 | 0.442072591 | 2.20E-25 | postive |
| TAZ | AL445222.2 | 0.432541245 | 2.94E-24 | postive |
| PHKG2 | AC040977.1 | 0.617371851 | 5.78E-54 | postive |
| GLS2 | LINC00885 | 0.408369637 | 1.47E-21 | postive |
| GABARAPL1 | LINC00885 | 0.413928685 | 3.69E-22 | postive |
| TAZ | MIR17HG | 0.507207228 | 4.16E-34 | postive |
| TUBE1 | GABPB1-AS1 | 0.493011846 | 4.82E-32 | postive |
| GABPB1 | GABPB1-AS1 | 0.493612839 | 3.96E-32 | postive |
| FANCD2 | AL079303.1 | 0.457675807 | 2.63E-27 | postive |
| HELLS | AL079303.1 | 0.46086984 | 1.03E-27 | postive |
| STMN1 | AL079303.1 | 0.4911581 | 8.82E-32 | postive |
| NOX1 | AL079303.1 | 0.421221435 | 5.76E-23 | postive |
| GLS2 | AL079303.1 | 0.403315586 | 5.08E-21 | postive |
| GOT1 | AL079303.1 | 0.42056617 | 6.82E-23 | postive |
| STMN1 | DCST1-AS1 | 0.436831651 | 9.24E-25 | postive |
| NOX1 | DCST1-AS1 | 0.494426325 | 3.03E-32 | postive |
| PHKG2 | DCST1-AS1 | 0.420652444 | 6.67E-23 | postive |
| TAZ | DCST1-AS1 | 0.462371151 | 6.63E-28 | postive |
| PHKG2 | LINC02166 | 0.493098214 | 4.68E-32 | postive |
| ZNF419 | AL136295.6 | 0.4061735 | 2.53E-21 | postive |
| PHKG2 | AL136295.6 | 0.564503849 | 1.67E-43 | postive |
| EGLN2 | AL136295.6 | 0.452370818 | 1.22E-26 | postive |
| TAZ | AL136295.6 | 0.49676805 | 1.40E-32 | postive |
| GPX4 | AC107982.3 | 0.51715207 | 1.30E-35 | postive |
| ISCU | AC107982.3 | 0.473081793 | 2.64E-29 | postive |
| MAP1LC3A | AC107982.3 | 0.493536938 | 4.06E-32 | postive |
| SOCS1 | AC107982.3 | 0.421809524 | 4.95E-23 | postive |
| CHMP6 | AL031432.3 | 0.405431645 | 3.04E-21 | postive |
| PHKG2 | AL031432.3 | 0.51623627 | 1.80E-35 | postive |
| EGLN2 | AL031432.3 | 0.416744024 | 1.81E-22 | postive |
| TAZ | AL031432.3 | 0.404863776 | 3.49E-21 | postive |
| FADS2 | AC080013.1 | 0.452710545 | 1.10E-26 | postive |
| YY1AP1 | AC080013.1 | 0.408746373 | 1.34E-21 | postive |
| NNMT | AC069360.1 | 0.416834966 | 1.77E-22 | postive |
| NFE2L2 | AF165147.1 | 0.449704399 | 2.60E-26 | postive |
| NFE2L2 | AL031667.3 | 0.409409962 | 1.14E-21 | postive |
| HELLS | ANKRD10-IT1 | 0.427054537 | 1.26E-23 | postive |
| TUBE1 | ANKRD10-IT1 | 0.414490249 | 3.20E-22 | postive |
| LPIN1 | ANKRD10-IT1 | 0.420602065 | 6.75E-23 | postive |
| ATM | ANKRD10-IT1 | 0.427384622 | 1.16E-23 | postive |
| MT3 | PCOTH | 0.461143783 | 9.53E-28 | postive |
| PEBP1 | PCOTH | 0.454276907 | 7.03E-27 | postive |
| CDO1 | PCOTH | 0.470999204 | 4.99E-29 | postive |
| FANCD2 | MIR9-3HG | 0.46174468 | 7.98E-28 | postive |
| TP53 | MIR9-3HG | 0.414744565 | 3.00E-22 | postive |
| HELLS | MIR9-3HG | 0.509119406 | 2.15E-34 | postive |
| ISCU | MIR9-3HG | 0.44461831 | 1.08E-25 | postive |
| STMN1 | MIR9-3HG | 0.503714034 | 1.37E-33 | postive |
| RRM2 | MIR9-3HG | 0.415309708 | 2.60E-22 | postive |
| NOX1 | MIR9-3HG | 0.508671786 | 2.51E-34 | postive |
| GLS2 | MIR9-3HG | 0.54011206 | 2.78E-39 | postive |
| GOT1 | MIR9-3HG | 0.415696042 | 2.36E-22 | postive |
| ATG3 | MIR9-3HG | 0.467782743 | 1.32E-28 | postive |
| ATG4D | MIR9-3HG | 0.42046889 | 6.99E-23 | postive |
| CDKN2A | MIR9-3HG | 0.569540838 | 2.03E-44 | postive |
| MAPK8 | MIR9-3HG | 0.400304321 | 1.05E-20 | postive |
| YY1AP1 | MIR9-3HG | 0.4004509 | 1.02E-20 | postive |
| FANCD2 | LNCTAM34A | 0.41150887 | 6.77E-22 | postive |
| HELLS | LNCTAM34A | 0.419629825 | 8.67E-23 | postive |
| ISCU | LNCTAM34A | 0.44477148 | 1.04E-25 | postive |
| KLHL24 | LNCTAM34A | 0.418536152 | 1.15E-22 | postive |
| ELAVL1 | LNCTAM34A | 0.417888136 | 1.35E-22 | postive |
| STMN1 | LNCTAM34A | 0.438232892 | 6.31E-25 | postive |
| KEAP1 | LNCTAM34A | 0.479945919 | 3.16E-30 | postive |
| ATG4D | LNCTAM34A | 0.483745295 | 9.53E-31 | postive |
| PANX1 | LNCTAM34A | -0.437287992 | 8.16E-25 | negative |
| KLHL24 | AC009120.3 | 0.409345107 | 1.16E-21 | postive |
| SETD1B | AC009120.3 | 0.450253109 | 2.22E-26 | postive |
| SP1 | AC009120.3 | 0.473481742 | 2.34E-29 | postive |
| GLS2 | AC009120.3 | 0.460893364 | 1.03E-27 | postive |
| SNX4 | AC009120.3 | 0.402523268 | 6.16E-21 | postive |
| ACVR1B | AC009120.3 | 0.441302506 | 2.72E-25 | postive |
| PEBP1 | AC004812.2 | 0.406087172 | 2.59E-21 | postive |
| KEAP1 | LINC00963 | 0.424159813 | 2.69E-23 | postive |
| TUBE1 | AP005329.2 | 0.428499444 | 8.61E-24 | postive |
| KRAS | AP005329.2 | 0.412847492 | 4.84E-22 | postive |
| MIOX | AP005329.2 | 0.426198533 | 1.58E-23 | postive |
| CHMP6 | AP003419.3 | 0.429550722 | 6.52E-24 | postive |
| PHKG2 | AP003419.3 | 0.647521549 | 7.07E-61 | postive |
| MAP1LC3A | AP003419.3 | 0.524555679 | 9.15E-37 | postive |
| EGLN2 | AP003419.3 | 0.490675354 | 1.03E-31 | postive |
| TAZ | AP003419.3 | 0.4697661 | 7.26E-29 | postive |
| PLIN4 | AC053503.3 | 0.749212502 | 2.47E-91 | postive |
| PRKAA2 | AC053503.3 | 0.604462207 | 3.15E-51 | postive |
| ALOX12B | AC007728.3 | 0.501088683 | 3.31E-33 | postive |
| ALOXE3 | AC007728.3 | 0.417054409 | 1.67E-22 | postive |
| RPL8 | AP001505.1 | 0.50829751 | 2.86E-34 | postive |
| SLC2A8 | AP001505.1 | 0.450754909 | 1.93E-26 | postive |
| NOX1 | AP001505.1 | 0.535467228 | 1.62E-38 | postive |
| HRAS | AP001505.1 | 0.405172509 | 3.23E-21 | postive |
| PHKG2 | AP001505.1 | 0.552151747 | 2.53E-41 | postive |
| EGLN2 | AP001505.1 | 0.600774241 | 1.81E-50 | postive |
| NNMT | MIR1-1HG | 0.427723002 | 1.06E-23 | postive |
| PLIN4 | MIR1-1HG | 0.787748334 | 4.52E-107 | postive |
| PRKAA2 | MIR1-1HG | 0.654303398 | 1.53E-62 | postive |
| ISCU | AC091563.1 | 0.419866337 | 8.16E-23 | postive |
| PLIN4 | AC091563.1 | 0.625079529 | 1.17E-55 | postive |
| PRKAA2 | AC091563.1 | 0.538016045 | 6.18E-39 | postive |
| TUBE1 | AC073842.2 | 0.44436118 | 1.17E-25 | postive |
| YY1AP1 | AC073842.2 | 0.463017953 | 5.48E-28 | postive |
| GPX4 | AC108673.3 | 0.482366208 | 1.47E-30 | postive |
| ATF4 | AC108673.3 | 0.438332668 | 6.14E-25 | postive |
| SLC2A8 | AC108673.3 | 0.451108414 | 1.74E-26 | postive |
| NOX1 | AC108673.3 | 0.509136355 | 2.14E-34 | postive |
| ACSL4 | AC108673.3 | -0.400322798 | 1.05E-20 | negative |
| PHKG2 | AC108673.3 | 0.699606196 | 7.29E-75 | postive |
| MAP1LC3A | AC108673.3 | 0.437658003 | 7.38E-25 | postive |
| PEBP1 | AC108673.3 | 0.441334328 | 2.70E-25 | postive |
| ANO6 | AC108673.3 | -0.501247471 | 3.14E-33 | negative |
| EGLN2 | AC108673.3 | 0.584466987 | 3.14E-47 | postive |
| TAZ | AC108673.3 | 0.562396228 | 3.99E-43 | postive |
| MTDH | AC108673.3 | -0.416304169 | 2.02E-22 | negative |
| HELLS | AL137058.2 | 0.438867056 | 5.31E-25 | postive |
| ISCU | AL391069.2 | 0.426112443 | 1.61E-23 | postive |
| STMN1 | AL391069.2 | 0.450357507 | 2.16E-26 | postive |
| PEBP1 | AL391069.2 | 0.443928573 | 1.31E-25 | postive |
| MYB | AL391069.2 | 0.47099814 | 4.99E-29 | postive |
| TP53 | AC087752.3 | 0.407835786 | 1.68E-21 | postive |
| HELLS | AC087752.3 | 0.438522326 | 5.83E-25 | postive |
| ISCU | AC087752.3 | 0.409072216 | 1.24E-21 | postive |
| ELAVL1 | AC087752.3 | 0.424669253 | 2.35E-23 | postive |
| STMN1 | AC087752.3 | 0.435958453 | 1.17E-24 | postive |
| GLS2 | AC087752.3 | 0.418988834 | 1.02E-22 | postive |
| MYB | AC087752.3 | 0.431843834 | 3.54E-24 | postive |
| PSAT1 | LINC01932 | 0.411577318 | 6.65E-22 | postive |
| TUBE1 | LINC01932 | 0.425674084 | 1.81E-23 | postive |
| ALOX12B | AL359979.1 | 0.439523304 | 4.43E-25 | postive |
| STAT3 | EPB41L4A-DT | 0.422307317 | 4.35E-23 | postive |
| ISCU | EPB41L4A-DT | 0.452798164 | 1.08E-26 | postive |
| GCH1 | EPB41L4A-DT | 0.42811149 | 9.54E-24 | postive |
| AGPAT3 | EPB41L4A-DT | 0.404624265 | 3.70E-21 | postive |
| SP1 | EPB41L4A-DT | 0.423027673 | 3.61E-23 | postive |
| DUOX2 | EPB41L4A-DT | 0.418442362 | 1.17E-22 | postive |
| GLS2 | EPB41L4A-DT | 0.497666907 | 1.04E-32 | postive |
| ATG4D | EPB41L4A-DT | 0.419605837 | 8.72E-23 | postive |
| SETD1B | AC010761.3 | 0.440251819 | 3.63E-25 | postive |
| SP1 | AC010761.3 | 0.416325809 | 2.01E-22 | postive |
| ACVR1B | AC010761.3 | 0.418005851 | 1.31E-22 | postive |
| GPX4 | AC004801.6 | 0.474929656 | 1.50E-29 | postive |
| CISD1 | AC004801.6 | 0.460523949 | 1.14E-27 | postive |
| FANCD2 | AC004801.6 | 0.467262318 | 1.54E-28 | postive |
| HELLS | AC004801.6 | 0.476384795 | 9.56E-30 | postive |
| ISCU | AC004801.6 | 0.587205806 | 9.24E-48 | postive |
| HMGB1 | AC004801.6 | 0.473762948 | 2.15E-29 | postive |
| STMN1 | AC004801.6 | 0.507421213 | 3.86E-34 | postive |
| NOX1 | AC004801.6 | 0.56999907 | 1.67E-44 | postive |
| CARS1 | AC004801.6 | 0.462934689 | 5.62E-28 | postive |
| ATG4D | AC004801.6 | 0.405198216 | 3.21E-21 | postive |
| CDKN2A | AC004801.6 | 0.476931139 | 8.07E-30 | postive |
| SOCS1 | AC004801.6 | 0.418892231 | 1.05E-22 | postive |
| FANCD2 | FGD5-AS1 | 0.652629683 | 3.98E-62 | postive |
| TP53 | FGD5-AS1 | 0.433957163 | 2.01E-24 | postive |
| HELLS | FGD5-AS1 | 0.591774313 | 1.17E-48 | postive |
| GCH1 | FGD5-AS1 | 0.444037044 | 1.28E-25 | postive |
| KLHL24 | FGD5-AS1 | 0.470713411 | 5.45E-29 | postive |
| ARRDC3 | FGD5-AS1 | 0.43229972 | 3.14E-24 | postive |
| IREB2 | FGD5-AS1 | 0.446720668 | 6.02E-26 | postive |
| ELAVL1 | FGD5-AS1 | 0.42850672 | 8.60E-24 | postive |
| SP1 | FGD5-AS1 | 0.643018857 | 8.53E-60 | postive |
| STMN1 | FGD5-AS1 | 0.437685533 | 7.33E-25 | postive |
| GABPB1 | FGD5-AS1 | 0.42584722 | 1.73E-23 | postive |
| GLS2 | FGD5-AS1 | 0.522869784 | 1.68E-36 | postive |
| ATG7 | FGD5-AS1 | 0.462953218 | 5.59E-28 | postive |
| SNX4 | FGD5-AS1 | 0.534550006 | 2.29E-38 | postive |
| MAPK8 | FGD5-AS1 | 0.56916328 | 2.38E-44 | postive |
| ACVR1B | FGD5-AS1 | 0.435288873 | 1.40E-24 | postive |
| ATM | FGD5-AS1 | 0.480953559 | 2.30E-30 | postive |
| SIRT1 | FGD5-AS1 | 0.537190694 | 8.45E-39 | postive |
| ISCU | AC016957.2 | 0.402990212 | 5.50E-21 | postive |
| AKR1C1 | AC010280.1 | 0.537831842 | 6.63E-39 | postive |
| AKR1C2 | AC010280.1 | 0.495291146 | 2.28E-32 | postive |
| AKR1C3 | AC010280.1 | 0.530858649 | 9.06E-38 | postive |
| GPX2 | AC010280.1 | 0.495973442 | 1.82E-32 | postive |
| TFRC | AC010280.1 | 0.407009887 | 2.06E-21 | postive |
| ABCC1 | AC010280.1 | 0.401234549 | 8.41E-21 | postive |
| IDH1 | AC010280.1 | 0.406228977 | 2.50E-21 | postive |
| NFE2L2 | AC010980.2 | 0.428039401 | 9.73E-24 | postive |
| TUBE1 | AC010980.2 | 0.437168527 | 8.43E-25 | postive |
| ELAVL1 | AC010980.2 | 0.460373251 | 1.20E-27 | postive |
| MYB | AC010980.2 | 0.444524796 | 1.11E-25 | postive |
| MT3 | SEMA3B-AS1 | 0.459267634 | 1.65E-27 | postive |
| CDO1 | SEMA3B-AS1 | 0.515669651 | 2.20E-35 | postive |
| ISCU | AC110792.3 | 0.413614027 | 3.99E-22 | postive |
| NOX1 | AC110792.3 | 0.447891831 | 4.33E-26 | postive |
| PHKG2 | AC110792.3 | 0.438063584 | 6.61E-25 | postive |
| TUBE1 | OBSCN-AS1 | 0.459567797 | 1.51E-27 | postive |
| GABPB1 | OBSCN-AS1 | 0.401386623 | 8.11E-21 | postive |
| YY1AP1 | OBSCN-AS1 | 0.430643885 | 4.88E-24 | postive |
| PHKG2 | AC010997.4 | 0.443983058 | 1.29E-25 | postive |
| TAZ | AC010997.4 | 0.455164548 | 5.45E-27 | postive |
| STMN1 | AL021807.1 | 0.447452694 | 4.90E-26 | postive |
| CDKN2A | AL021807.1 | 0.445675028 | 8.07E-26 | postive |
| TUBE1 | AC090739.1 | 0.445623835 | 8.19E-26 | postive |
| GPX4 | Z93930.2 | 0.432113429 | 3.30E-24 | postive |
| PHKG2 | Z93930.2 | 0.421285391 | 5.66E-23 | postive |
| EGLN2 | Z93930.2 | 0.434518826 | 1.73E-24 | postive |
| NFE2L2 | AC005586.1 | 0.494287341 | 3.17E-32 | postive |
| BRD4 | AC005586.1 | 0.403049165 | 5.42E-21 | postive |
| SP1 | AC005586.1 | 0.417401159 | 1.53E-22 | postive |
| GLS2 | AC005586.1 | 0.458557381 | 2.03E-27 | postive |
| KEAP1 | AC005586.1 | 0.405417728 | 3.05E-21 | postive |
| ATG4D | AC005586.1 | 0.400089704 | 1.11E-20 | postive |
| YY1AP1 | AC005586.1 | 0.437099408 | 8.59E-25 | postive |
| MIOX | AC010789.1 | 0.402558078 | 6.11E-21 | postive |
| PHKG2 | ENO1-AS1 | 0.489815865 | 1.36E-31 | postive |
| TAZ | ENO1-AS1 | 0.419425228 | 9.14E-23 | postive |
| BRD4 | AC108010.1 | 0.461207395 | 9.35E-28 | postive |
| SETD1B | AC108010.1 | 0.636259128 | 3.32E-58 | postive |
| IREB2 | AC108010.1 | 0.49951536 | 5.61E-33 | postive |
| SP1 | AC108010.1 | 0.574505213 | 2.44E-45 | postive |
| PIK3CA | AC108010.1 | 0.432670414 | 2.84E-24 | postive |
| ACVR1B | AC108010.1 | 0.449124828 | 3.06E-26 | postive |
| ATM | AC108010.1 | 0.494471233 | 2.99E-32 | postive |
| YY1AP1 | AC108010.1 | 0.40717738 | 1.98E-21 | postive |
| SIRT1 | AC108010.1 | 0.421649883 | 5.15E-23 | postive |
| ATM | AC004771.2 | 0.513458499 | 4.77E-35 | postive |
| ALOX12B | AC103974.1 | 0.465458606 | 2.65E-28 | postive |
| ALOXE3 | AC103974.1 | 0.422542295 | 4.09E-23 | postive |
| NFE2L2 | AL133243.2 | 0.501978211 | 2.46E-33 | postive |
| KLHL24 | AL133243.2 | 0.517056968 | 1.35E-35 | postive |
| TUBE1 | AL133243.2 | 0.534769451 | 2.11E-38 | postive |
| ELAVL1 | AL133243.2 | 0.42378384 | 2.96E-23 | postive |
| GABPB1 | AL133243.2 | 0.436913377 | 9.04E-25 | postive |
| PIK3CA | AL133243.2 | 0.442908463 | 1.74E-25 | postive |
| GABARAPL1 | AL133243.2 | 0.432055417 | 3.35E-24 | postive |
| BID | AL133243.2 | 0.418464866 | 1.17E-22 | postive |
| LPIN1 | AL133243.2 | 0.417926642 | 1.34E-22 | postive |
| YY1AP1 | AL133243.2 | 0.400827226 | 9.28E-21 | postive |
| NFE2L2 | DTX2P1-UPK3BP1-PMS2P11 | 0.402842459 | 5.70E-21 | postive |
| HELLS | DTX2P1-UPK3BP1-PMS2P11 | 0.476346202 | 9.68E-30 | postive |
| ISCU | DTX2P1-UPK3BP1-PMS2P11 | 0.402035318 | 6.93E-21 | postive |
| BRD4 | DTX2P1-UPK3BP1-PMS2P11 | 0.404897232 | 3.46E-21 | postive |
| ELAVL1 | DTX2P1-UPK3BP1-PMS2P11 | 0.423578942 | 3.13E-23 | postive |
| SP1 | DTX2P1-UPK3BP1-PMS2P11 | 0.433877476 | 2.05E-24 | postive |
| GLS2 | DTX2P1-UPK3BP1-PMS2P11 | 0.506536591 | 5.23E-34 | postive |
| KEAP1 | DTX2P1-UPK3BP1-PMS2P11 | 0.408943346 | 1.28E-21 | postive |
| ATG4D | DTX2P1-UPK3BP1-PMS2P11 | 0.454386953 | 6.81E-27 | postive |
| MAPK8 | DTX2P1-UPK3BP1-PMS2P11 | 0.404437545 | 3.87E-21 | postive |
| YY1AP1 | DTX2P1-UPK3BP1-PMS2P11 | 0.473840903 | 2.10E-29 | postive |
| NFE2L2 | LINC02820 | 0.435094081 | 1.48E-24 | postive |
| KLHL24 | LINC02820 | 0.417886577 | 1.35E-22 | postive |
| TUBE1 | LINC02820 | 0.401416776 | 8.05E-21 | postive |
| TUBE1 | LINC00899 | 0.431562266 | 3.82E-24 | postive |
| PHKG2 | LINC00899 | 0.40657317 | 2.30E-21 | postive |
| TAZ | LINC00899 | 0.404749483 | 3.59E-21 | postive |
| FANCD2 | AL157392.3 | 0.412441225 | 5.36E-22 | postive |
| HELLS | AL157392.3 | 0.569881273 | 1.75E-44 | postive |
| TUBE1 | AL157392.3 | 0.423868659 | 2.90E-23 | postive |
| ELAVL1 | AL157392.3 | 0.407095449 | 2.02E-21 | postive |
| SP1 | AL157392.3 | 0.499833033 | 5.05E-33 | postive |
| STMN1 | AL157392.3 | 0.4570614 | 3.14E-27 | postive |
| GLS2 | AL157392.3 | 0.496954395 | 1.32E-32 | postive |
| MYB | AL157392.3 | 0.443100689 | 1.65E-25 | postive |
| MAPK8 | AL157392.3 | 0.537099102 | 8.75E-39 | postive |
| ACVR1B | AL157392.3 | 0.438909607 | 5.25E-25 | postive |
| YY1AP1 | AL157392.3 | 0.43428898 | 1.84E-24 | postive |
| SIRT1 | AL157392.3 | 0.422324721 | 4.33E-23 | postive |
| ALOXE3 | C20orf197 | 0.410571165 | 8.55E-22 | postive |
| GLS2 | AL035258.1 | 0.45185973 | 1.41E-26 | postive |
| CHMP6 | AC138207.2 | 0.455547916 | 4.87E-27 | postive |
| PHKG2 | AC138207.2 | 0.484839781 | 6.73E-31 | postive |
| MAP1LC3A | AC138207.2 | 0.452357336 | 1.22E-26 | postive |
| EGLN2 | AC138207.2 | 0.44061248 | 3.29E-25 | postive |
| PML | LINC02100 | 0.421733701 | 5.04E-23 | postive |
| GPX4 | RPARP-AS1 | 0.471165675 | 4.75E-29 | postive |
| CISD1 | RPARP-AS1 | 0.435777022 | 1.23E-24 | postive |
| ATF4 | RPARP-AS1 | 0.436211398 | 1.09E-24 | postive |
| HELLS | RPARP-AS1 | 0.45659457 | 3.60E-27 | postive |
| FH | RPARP-AS1 | 0.442915362 | 1.74E-25 | postive |
| ISCU | RPARP-AS1 | 0.494724905 | 2.75E-32 | postive |
| PRDX6 | RPARP-AS1 | 0.436690895 | 9.60E-25 | postive |
| SLC2A8 | RPARP-AS1 | 0.471194498 | 4.70E-29 | postive |
| STMN1 | RPARP-AS1 | 0.536359853 | 1.16E-38 | postive |
| NOX1 | RPARP-AS1 | 0.506028884 | 6.22E-34 | postive |
| PHKG2 | RPARP-AS1 | 0.638432594 | 1.03E-58 | postive |
| MAP1LC3A | RPARP-AS1 | 0.418583834 | 1.13E-22 | postive |
| PEBP1 | RPARP-AS1 | 0.595913018 | 1.75E-49 | postive |
| MYB | RPARP-AS1 | 0.412027117 | 5.94E-22 | postive |
| ANO6 | RPARP-AS1 | -0.450952461 | 1.82E-26 | negative |
| EGLN2 | RPARP-AS1 | 0.568634436 | 2.97E-44 | postive |
| TAZ | RPARP-AS1 | 0.596642281 | 1.25E-49 | postive |
| PANX1 | RPARP-AS1 | -0.422340053 | 4.31E-23 | negative |
| LONP1 | RPARP-AS1 | 0.40562738 | 2.89E-21 | postive |
| GABPB1 | AC006449.2 | 0.412412789 | 5.40E-22 | postive |
| GPX4 | YTHDF3-AS1 | 0.47804194 | 5.72E-30 | postive |
| RPL8 | YTHDF3-AS1 | 0.533185991 | 3.81E-38 | postive |
| SLC2A8 | YTHDF3-AS1 | 0.465584192 | 2.55E-28 | postive |
| PHKG2 | YTHDF3-AS1 | 0.529131655 | 1.72E-37 | postive |
| EGLN2 | YTHDF3-AS1 | 0.656495431 | 4.34E-63 | postive |
| TAZ | YTHDF3-AS1 | 0.481551332 | 1.91E-30 | postive |
| PHKG2 | AL355001.2 | 0.412942148 | 4.73E-22 | postive |
| TAZ | AL355001.2 | 0.412302351 | 5.55E-22 | postive |
| HELLS | AC004943.2 | 0.428571047 | 8.45E-24 | postive |
| GPX4 | SNHG30 | 0.409409695 | 1.14E-21 | postive |
| PRDX6 | SNHG30 | 0.423052975 | 3.58E-23 | postive |
| SLC2A8 | SNHG30 | 0.409224333 | 1.19E-21 | postive |
| ELAVL1 | SNHG30 | 0.450621722 | 2.00E-26 | postive |
| STMN1 | SNHG30 | 0.407048225 | 2.04E-21 | postive |
| GABPB1 | SNHG30 | 0.412270718 | 5.59E-22 | postive |
| PHKG2 | SNHG30 | 0.524692675 | 8.70E-37 | postive |
| PEBP1 | SNHG30 | 0.447611158 | 4.69E-26 | postive |
| TAZ | SNHG30 | 0.480940459 | 2.31E-30 | postive |
| LONP1 | SNHG30 | 0.413410294 | 4.20E-22 | postive |
| HRAS | LINC01730 | 0.406086246 | 2.59E-21 | postive |
| ATP5MC3 | AC010894.2 | 0.490174835 | 1.21E-31 | postive |
| TAZ | AC010894.2 | 0.40965255 | 1.07E-21 | postive |
| PHKG2 | AC005696.3 | 0.474606241 | 1.66E-29 | postive |
| PHKG2 | AC253536.6 | 0.472131214 | 3.54E-29 | postive |
| TAZ | AC253536.6 | 0.41142096 | 6.92E-22 | postive |
| KLHL24 | AC106798.1 | 0.401161132 | 8.56E-21 | postive |
| PIK3CA | AC106798.1 | 0.418961314 | 1.03E-22 | postive |
| TUBE1 | Z83843.1 | 0.457518598 | 2.75E-27 | postive |
| GPX4 | AL139246.3 | 0.525710684 | 6.01E-37 | postive |
| CISD1 | AL139246.3 | 0.400353495 | 1.04E-20 | postive |
| ISCU | AL139246.3 | 0.532204626 | 5.49E-38 | postive |
| MAP1LC3A | AL139246.3 | 0.434584276 | 1.70E-24 | postive |
| SOCS1 | AL139246.3 | 0.566696657 | 6.70E-44 | postive |
| MYB | AL139246.3 | 0.447331561 | 5.07E-26 | postive |
| ALOX12B | AC011483.1 | 0.466094909 | 2.19E-28 | postive |
| ALOXE3 | AC011483.1 | 0.49679887 | 1.39E-32 | postive |
| FANCD2 | AC012467.2 | 0.46242163 | 6.54E-28 | postive |
| TP53 | AC012467.2 | 0.41419389 | 3.45E-22 | postive |
| HELLS | AC012467.2 | 0.574455941 | 2.50E-45 | postive |
| FADS2 | AC012467.2 | 0.430154085 | 5.56E-24 | postive |
| ISCU | AC012467.2 | 0.49627006 | 1.65E-32 | postive |
| KLHL24 | AC012467.2 | 0.401483328 | 7.92E-21 | postive |
| ELAVL1 | AC012467.2 | 0.472642925 | 3.02E-29 | postive |
| SP1 | AC012467.2 | 0.472751737 | 2.93E-29 | postive |
| STMN1 | AC012467.2 | 0.474874214 | 1.52E-29 | postive |
| NOX1 | AC012467.2 | 0.415380099 | 2.56E-22 | postive |
| GLS2 | AC012467.2 | 0.499780896 | 5.14E-33 | postive |
| PEBP1 | AC012467.2 | 0.401660271 | 7.59E-21 | postive |
| MYB | AC012467.2 | 0.46901391 | 9.11E-29 | postive |
| MAPK8 | AC012467.2 | 0.447518705 | 4.81E-26 | postive |
| LPIN1 | AC012467.2 | 0.424355391 | 2.56E-23 | postive |
| YY1AP1 | AC012467.2 | 0.431247011 | 4.15E-24 | postive |
| SIRT1 | AC012467.2 | 0.444340309 | 1.17E-25 | postive |
| NFE2L2 | OR2A1-AS1 | 0.404107412 | 4.19E-21 | postive |
| ISCU | AC005332.4 | 0.402768695 | 5.81E-21 | postive |
| TUBE1 | AC005332.4 | 0.543169533 | 8.59E-40 | postive |
| ELAVL1 | AC005332.4 | 0.480629458 | 2.55E-30 | postive |
| STMN1 | AC005332.4 | 0.433502242 | 2.27E-24 | postive |
| GABPB1 | AC005332.4 | 0.451798349 | 1.43E-26 | postive |
| GLS2 | AC005332.4 | 0.405709562 | 2.84E-21 | postive |
| ATG4D | AC005332.4 | 0.42293731 | 3.69E-23 | postive |
| PEBP1 | AC005332.4 | 0.422965663 | 3.67E-23 | postive |
| CHMP6 | AC092171.5 | 0.494669275 | 2.80E-32 | postive |
| PHKG2 | AC092171.5 | 0.586113056 | 1.51E-47 | postive |
| MAP1LC3A | AC092171.5 | 0.405294366 | 3.14E-21 | postive |
| WIPI2 | AC092171.5 | 0.505967102 | 6.36E-34 | postive |
| EGLN2 | AC092171.5 | 0.444222182 | 1.21E-25 | postive |
| TAZ | AC092171.5 | 0.42277181 | 3.86E-23 | postive |
| GPX4 | AL035461.3 | 0.49987135 | 4.98E-33 | postive |
| FANCD2 | AL035461.3 | 0.418305637 | 1.22E-22 | postive |
| ATF4 | AL035461.3 | 0.407765738 | 1.71E-21 | postive |
| HELLS | AL035461.3 | 0.414503984 | 3.19E-22 | postive |
| ISCU | AL035461.3 | 0.472373518 | 3.28E-29 | postive |
| SLC2A8 | AL035461.3 | 0.503537862 | 1.45E-33 | postive |
| STMN1 | AL035461.3 | 0.58661759 | 1.20E-47 | postive |
| NOX1 | AL035461.3 | 0.526969511 | 3.79E-37 | postive |
| PHKG2 | AL035461.3 | 0.544136732 | 5.91E-40 | postive |
| PEBP1 | AL035461.3 | 0.43651681 | 1.01E-24 | postive |
| SOCS1 | AL035461.3 | 0.483648706 | 9.82E-31 | postive |
| MYB | AL035461.3 | 0.419747663 | 8.41E-23 | postive |
| EGLN2 | AL035461.3 | 0.531407435 | 7.39E-38 | postive |
| TAZ | AL035461.3 | 0.497475274 | 1.11E-32 | postive |
| PHKG2 | AC011498.6 | 0.460273067 | 1.23E-27 | postive |
| TAZ | AC011498.6 | 0.424060134 | 2.76E-23 | postive |
| TUBE1 | AL356299.3 | 0.40455047 | 3.76E-21 | postive |
| NNMT | AC021087.4 | 0.46762715 | 1.38E-28 | postive |
| PLIN4 | AC021087.4 | 0.696603096 | 5.63E-74 | postive |
| PRKAA2 | AC021087.4 | 0.607943822 | 5.92E-52 | postive |
| ATF4 | AC010973.2 | 0.400947821 | 9.01E-21 | postive |
| PHKG2 | AC010973.2 | 0.546669032 | 2.20E-40 | postive |
| TAZ | AC010973.2 | 0.544675962 | 4.79E-40 | postive |
| GPX4 | AC069307.1 | 0.441945244 | 2.28E-25 | postive |
| ATF4 | AC069307.1 | 0.413296191 | 4.33E-22 | postive |
| NOX1 | AC069307.1 | 0.45252771 | 1.16E-26 | postive |
| PHKG2 | AC069307.1 | 0.488744174 | 1.93E-31 | postive |
| EGLN2 | AC069307.1 | 0.413113695 | 4.53E-22 | postive |
| TAZ | AC069307.1 | 0.413194456 | 4.44E-22 | postive |
| OTUB1 | AP003068.1 | 0.572265789 | 6.37E-45 | postive |
| HRAS | AP003068.1 | 0.474860115 | 1.53E-29 | postive |
| GPX4 | FOXD3-AS1 | 0.522104579 | 2.22E-36 | postive |
| CISD1 | FOXD3-AS1 | 0.535991679 | 1.33E-38 | postive |
| ISCU | FOXD3-AS1 | 0.526938905 | 3.84E-37 | postive |
| AIFM2 | FOXD3-AS1 | 0.437851111 | 7.00E-25 | postive |
| MT3 | FOXD3-AS1 | 0.40825437 | 1.52E-21 | postive |
| STMN1 | FOXD3-AS1 | 0.500845614 | 3.60E-33 | postive |
| GLS2 | FOXD3-AS1 | 0.451528508 | 1.55E-26 | postive |
| PHKG2 | FOXD3-AS1 | 0.40676359 | 2.19E-21 | postive |
| MAP1LC3A | FOXD3-AS1 | 0.54524355 | 3.84E-40 | postive |
| PEBP1 | FOXD3-AS1 | 0.500859014 | 3.58E-33 | postive |
| MIOX | FOXD3-AS1 | 0.442795223 | 1.80E-25 | postive |
| NFE2L2 | AC008124.1 | 0.424464408 | 2.48E-23 | postive |
| HELLS | AC008124.1 | 0.441371713 | 2.67E-25 | postive |
| ISCU | AC008124.1 | 0.408987681 | 1.27E-21 | postive |
| KLHL24 | AC008124.1 | 0.502665473 | 1.95E-33 | postive |
| TUBE1 | AC008124.1 | 0.429869274 | 5.99E-24 | postive |
| ELAVL1 | AC008124.1 | 0.41455596 | 3.15E-22 | postive |
| SP1 | AC008124.1 | 0.403818479 | 4.50E-21 | postive |
| GABPB1 | AC008124.1 | 0.518976475 | 6.80E-36 | postive |
| GLS2 | AC008124.1 | 0.49609649 | 1.75E-32 | postive |
| ATG4D | AC008124.1 | 0.40233031 | 6.46E-21 | postive |
| YY1AP1 | AC008124.1 | 0.402722011 | 5.87E-21 | postive |
| SIRT1 | AC008124.1 | 0.461084169 | 9.70E-28 | postive |
| SELENOS | SNHG21 | 0.405165764 | 3.24E-21 | postive |
| TUBE1 | SNHG21 | 0.478799165 | 4.52E-30 | postive |
| GABPB1 | SNHG21 | 0.450313308 | 2.18E-26 | postive |
| PEBP1 | SNHG21 | 0.44946404 | 2.78E-26 | postive |
| TAZ | SNHG21 | 0.408971827 | 1.27E-21 | postive |
| GPX4 | AC016394.2 | 0.459061942 | 1.76E-27 | postive |
| CISD1 | AC016394.2 | 0.503822158 | 1.32E-33 | postive |
| ATF4 | AC016394.2 | 0.444877906 | 1.01E-25 | postive |
| HELLS | AC016394.2 | 0.475633757 | 1.21E-29 | postive |
| CISD2 | AC016394.2 | 0.400136316 | 1.10E-20 | postive |
| ISCU | AC016394.2 | 0.474739734 | 1.59E-29 | postive |
| HMGB1 | AC016394.2 | 0.455801597 | 4.53E-27 | postive |
| STMN1 | AC016394.2 | 0.598352605 | 5.62E-50 | postive |
| NOX1 | AC016394.2 | 0.48609795 | 4.50E-31 | postive |
| PHKG2 | AC016394.2 | 0.545522583 | 3.45E-40 | postive |
| PEBP1 | AC016394.2 | 0.419654421 | 8.61E-23 | postive |
| TAZ | AC016394.2 | 0.56878584 | 2.79E-44 | postive |
| CISD1 | AC021028.1 | 0.447374328 | 5.01E-26 | postive |
| ISCU | AC021028.1 | 0.410205494 | 9.36E-22 | postive |
| BRD4 | AC006001.2 | 0.404179755 | 4.12E-21 | postive |
| STMN1 | AC100814.2 | 0.452248581 | 1.26E-26 | postive |
| NOX1 | AC100814.2 | 0.495183042 | 2.36E-32 | postive |
| TAZ | AC100814.2 | 0.422940022 | 3.69E-23 | postive |
| CISD1 | COLCA1 | 0.403576911 | 4.77E-21 | postive |
| HELLS | COLCA1 | 0.429174954 | 7.20E-24 | postive |
| ISCU | COLCA1 | 0.448258613 | 3.91E-26 | postive |
| GLS2 | COLCA1 | 0.580749733 | 1.62E-46 | postive |
| MAPK8 | COLCA1 | 0.455656456 | 4.72E-27 | postive |
| TUBE1 | ATXN1-AS1 | 0.431291254 | 4.11E-24 | postive |
| PIK3CA | ATXN1-AS1 | 0.402133888 | 6.77E-21 | postive |
| HELLS | AL355075.2 | 0.43531803 | 1.39E-24 | postive |
| TUBE1 | AL355075.2 | 0.411765675 | 6.35E-22 | postive |
| PEBP1 | AC110285.2 | 0.468018799 | 1.23E-28 | postive |
| TUBE1 | AC005104.1 | 0.458483961 | 2.08E-27 | postive |
| ELAVL1 | AL391244.1 | 0.428170737 | 9.39E-24 | postive |
| BID | AL391244.1 | 0.44356528 | 1.45E-25 | postive |
| YY1AP1 | AL391244.1 | 0.412655242 | 5.08E-22 | postive |
| SETD1B | NPTN-IT1 | 0.406039663 | 2.62E-21 | postive |
| SP1 | NPTN-IT1 | 0.415704655 | 2.36E-22 | postive |
| ACVR1B | NPTN-IT1 | 0.426424467 | 1.49E-23 | postive |
| ATM | NPTN-IT1 | 0.459654556 | 1.48E-27 | postive |
| PHKG2 | HOXB-AS1 | 0.407195107 | 1.97E-21 | postive |
| ENPP2 | AL109741.1 | 0.570903324 | 1.14E-44 | postive |
| CYBB | AL109741.1 | 0.410524755 | 8.65E-22 | postive |
| FLT3 | AL109741.1 | 0.543417918 | 7.80E-40 | postive |
| ZEB1 | AL109741.1 | 0.55939598 | 1.37E-42 | postive |
| TLR4 | AL109741.1 | 0.499882128 | 4.97E-33 | postive |
| ATM | AL109741.1 | 0.440465263 | 3.42E-25 | postive |
| TUBE1 | ELOA-AS1 | 0.428152235 | 9.44E-24 | postive |
| STMN1 | ELOA-AS1 | 0.421767234 | 5.00E-23 | postive |
| MYB | MIR3142HG | 0.568419268 | 3.25E-44 | postive |
| ALOX12B | AC004816.1 | 0.412936111 | 4.73E-22 | postive |
| ALOXE3 | AC004816.1 | 0.429486808 | 6.63E-24 | postive |
| SLC2A8 | AL441992.2 | 0.444979178 | 9.81E-26 | postive |
| STMN1 | AL441992.2 | 0.400917449 | 9.08E-21 | postive |
| ISCU | AC005840.4 | 0.485189727 | 6.02E-31 | postive |
| NOX1 | AC005840.4 | 0.464838236 | 3.19E-28 | postive |
| SOCS1 | AC005840.4 | 0.406280002 | 2.47E-21 | postive |
| MAPK1 | OGFRP1 | 0.403164191 | 5.27E-21 | postive |
| TXNRD1 | TM4SF1-AS1 | 0.446191069 | 6.99E-26 | postive |
| GPX2 | TM4SF1-AS1 | 0.52282159 | 1.71E-36 | postive |
| HIC1 | AL135925.1 | 0.423892284 | 2.88E-23 | postive |
| PHKG2 | AC009309.2 | 0.431374162 | 4.02E-24 | postive |
| EGLN2 | AC009309.2 | 0.400978123 | 8.95E-21 | postive |
| TAZ | AC009309.2 | 0.427890229 | 1.01E-23 | postive |
| CEBPG | AC083801.2 | 0.556831141 | 3.88E-42 | postive |
| PRKAA1 | AC025171.1 | 0.4800736 | 3.03E-30 | postive |
| GPX4 | AC106897.2 | 0.473744939 | 2.16E-29 | postive |
| MUC1 | AC106897.2 | 0.460799062 | 1.05E-27 | postive |
| SLC7A11 | LINC01564 | 0.461018674 | 9.89E-28 | postive |
| AKR1C1 | LINC01564 | 0.699133899 | 1.01E-74 | postive |
| AKR1C2 | LINC01564 | 0.640962948 | 2.62E-59 | postive |
| AKR1C3 | LINC01564 | 0.686720022 | 3.96E-71 | postive |
| GCLC | LINC01564 | 0.695786639 | 9.78E-74 | postive |
| NFE2L2 | LINC01564 | 0.433132205 | 2.51E-24 | postive |
| NQO1 | LINC01564 | 0.574810054 | 2.14E-45 | postive |
| FTH1 | LINC01564 | 0.446630613 | 6.18E-26 | postive |
| PRDX6 | LINC01564 | 0.483279514 | 1.10E-30 | postive |
| AIFM2 | LINC01564 | 0.411332085 | 7.07E-22 | postive |
| TXNRD1 | LINC01564 | 0.657859996 | 1.97E-63 | postive |
| SRXN1 | LINC01564 | 0.485703313 | 5.11E-31 | postive |
| GPX2 | LINC01564 | 0.736361827 | 1.04E-86 | postive |
| ASNS | LINC01564 | 0.440921294 | 3.02E-25 | postive |
| FTL | LINC01564 | 0.445873388 | 7.64E-26 | postive |
| MAFG | LINC01564 | 0.520309349 | 4.23E-36 | postive |
| PRDX1 | LINC01564 | 0.50226526 | 2.23E-33 | postive |
| G6PD | LINC01564 | 0.462206855 | 6.96E-28 | postive |
| PGD | LINC01564 | 0.570649757 | 1.27E-44 | postive |
| ABCC1 | LINC01564 | 0.483302104 | 1.10E-30 | postive |
| IDH1 | LINC01564 | 0.515013093 | 2.77E-35 | postive |
| ISCU | ZNF710-AS1 | 0.427585454 | 1.10E-23 | postive |
| PLIN4 | ZNF710-AS1 | 0.584150821 | 3.62E-47 | postive |
| PRKAA2 | ZNF710-AS1 | 0.468850452 | 9.57E-29 | postive |
| ATF4 | SNHG5 | 0.455384767 | 5.11E-27 | postive |
| RPL8 | SNHG5 | 0.461123639 | 9.59E-28 | postive |
| GPX4 | AC026333.4 | 0.426096006 | 1.62E-23 | postive |
| CISD1 | AC026333.4 | 0.402116282 | 6.80E-21 | postive |
| FANCD2 | AC026333.4 | 0.486070159 | 4.54E-31 | postive |
| HELLS | AC026333.4 | 0.55723022 | 3.30E-42 | postive |
| ISCU | AC026333.4 | 0.578118851 | 5.13E-46 | postive |
| HMGB1 | AC026333.4 | 0.414603757 | 3.11E-22 | postive |
| STMN1 | AC026333.4 | 0.549630976 | 6.89E-41 | postive |
| NOX1 | AC026333.4 | 0.579483674 | 2.83E-46 | postive |
| PHKG2 | AC026333.4 | 0.429738535 | 6.20E-24 | postive |
| ATG4D | AC026333.4 | 0.41087053 | 7.93E-22 | postive |
| CDKN2A | AC026333.4 | 0.424886869 | 2.22E-23 | postive |
| SOCS1 | AC026333.4 | 0.426044075 | 1.64E-23 | postive |
| MYB | AC026333.4 | 0.493302501 | 4.38E-32 | postive |
| TAZ | AC026333.4 | 0.448911004 | 3.25E-26 | postive |
| PHKG2 | ZNF433-AS1 | 0.439237865 | 4.79E-25 | postive |
| MAP1LC3A | AL596442.2 | 0.428119015 | 9.52E-24 | postive |
| WIPI2 | AL596442.2 | 0.403063126 | 5.41E-21 | postive |
| TUBE1 | SNHG4 | 0.403295592 | 5.11E-21 | postive |
| CS | SNHG4 | 0.469945233 | 6.88E-29 | postive |
| HELLS | AP005482.4 | 0.400011145 | 1.13E-20 | postive |
| ISCU | AP005482.4 | 0.444736802 | 1.05E-25 | postive |
| TUBE1 | AP005482.4 | 0.438562676 | 5.77E-25 | postive |
| ELAVL1 | AP005482.4 | 0.437361168 | 8.00E-25 | postive |
| STMN1 | AP005482.4 | 0.404003629 | 4.30E-21 | postive |
| NOX1 | AP005482.4 | 0.436580224 | 9.89E-25 | postive |
| KRAS | AP005482.4 | 0.4291683 | 7.22E-24 | postive |
| ATG4D | AP005482.4 | 0.450891219 | 1.85E-26 | postive |
| CHMP6 | AC010531.6 | 0.411287483 | 7.15E-22 | postive |
| PHKG2 | AC010531.6 | 0.55928811 | 1.43E-42 | postive |
| EGLN2 | AC010531.6 | 0.428846935 | 7.86E-24 | postive |
| TAZ | AC010531.6 | 0.43851964 | 5.84E-25 | postive |
| CAV1 | LINC00707 | 0.427430246 | 1.14E-23 | postive |
| WIPI2 | AC092171.3 | 0.463277403 | 5.07E-28 | postive |
| ALOX12B | OVOL1-AS1 | 0.573503853 | 3.76E-45 | postive |
| ALOXE3 | OVOL1-AS1 | 0.458576784 | 2.02E-27 | postive |
| ATF4 | AP001160.1 | 0.481384788 | 2.01E-30 | postive |
| RPL8 | AP001160.1 | 0.428883736 | 7.78E-24 | postive |
| PHKG2 | AP001160.1 | 0.646918042 | 9.89E-61 | postive |
| EGLN2 | AP001160.1 | 0.571222722 | 9.94E-45 | postive |
| TAZ | AP001160.1 | 0.612609057 | 6.11E-53 | postive |
| SLC40A1 | AL365361.1 | 0.46515233 | 2.91E-28 | postive |
| ENPP2 | AL365361.1 | 0.553926579 | 1.25E-41 | postive |
| HERPUD1 | AL365361.1 | 0.597151326 | 9.84E-50 | postive |
| ALOX5 | AL365361.1 | 0.458362155 | 2.15E-27 | postive |
| CYBB | AL365361.1 | 0.516406878 | 1.69E-35 | postive |
| FLT3 | AL365361.1 | 0.795421878 | 1.34E-110 | postive |
| IFNG | AL365361.1 | 0.426989086 | 1.28E-23 | postive |
| ATM | AL365361.1 | 0.485166795 | 6.06E-31 | postive |
| TUBE1 | DNAJC3-DT | 0.401638082 | 7.63E-21 | postive |
| PHKG2 | AC093249.6 | 0.506903244 | 4.61E-34 | postive |
| PEBP1 | AC093249.6 | 0.459653355 | 1.48E-27 | postive |
| CAV1 | LINP1 | 0.565687853 | 1.02E-43 | postive |
| ANO6 | AC073569.2 | 0.463256457 | 5.11E-28 | postive |
| NFE2L2 | AC009506.2 | 0.490632637 | 1.05E-31 | postive |
| FADS2 | AC009506.2 | 0.404074602 | 4.23E-21 | postive |
| BRD4 | AC009506.2 | 0.402203808 | 6.66E-21 | postive |
| GPX2 | AC009506.2 | 0.417410149 | 1.53E-22 | postive |
| KLHL24 | AC009506.2 | 0.45234169 | 1.23E-26 | postive |
| TUBE1 | AC009506.2 | 0.452481311 | 1.18E-26 | postive |
| ELAVL1 | AC009506.2 | 0.464646019 | 3.38E-28 | postive |
| GABPB1 | AC009506.2 | 0.45601011 | 4.26E-27 | postive |
| GLS2 | AC009506.2 | 0.414945435 | 2.86E-22 | postive |
| KEAP1 | AC009506.2 | 0.402019471 | 6.96E-21 | postive |
| GABARAPL1 | AC009506.2 | 0.488269917 | 2.24E-31 | postive |
| BID | AC009506.2 | 0.449779458 | 2.54E-26 | postive |
| YY1AP1 | AC009506.2 | 0.43293539 | 2.65E-24 | postive |
| GPX4 | AC005790.1 | 0.486198553 | 4.36E-31 | postive |
| ISCU | AC005790.1 | 0.405716712 | 2.83E-21 | postive |
| MAP1LC3A | AC005790.1 | 0.458122596 | 2.31E-27 | postive |
| SOCS1 | AC005790.1 | 0.45374122 | 8.20E-27 | postive |
| PRKAA1 | AC114956.1 | 0.411214041 | 7.28E-22 | postive |
| PANX1 | MIR31HG | 0.443265538 | 1.58E-25 | postive |
| STAT3 | AC098851.1 | 0.405400057 | 3.06E-21 | postive |
| SP1 | AC098851.1 | 0.405782367 | 2.79E-21 | postive |
| MAPK8 | AC098851.1 | 0.437778992 | 7.14E-25 | postive |
| FANCD2 | AC232271.1 | 0.432464616 | 3.00E-24 | postive |
| HELLS | AC232271.1 | 0.572297647 | 6.29E-45 | postive |
| ISCU | AC232271.1 | 0.45514298 | 5.48E-27 | postive |
| TUBE1 | AC232271.1 | 0.444258658 | 1.20E-25 | postive |
| ELAVL1 | AC232271.1 | 0.410084758 | 9.65E-22 | postive |
| STMN1 | AC232271.1 | 0.503811614 | 1.32E-33 | postive |
| GABPB1 | AC232271.1 | 0.444426171 | 1.14E-25 | postive |
| NOX1 | AC232271.1 | 0.43262197 | 2.88E-24 | postive |
| GLS2 | AC232271.1 | 0.478349512 | 5.20E-30 | postive |
| PHKG2 | AC232271.1 | 0.40630396 | 2.45E-21 | postive |
| ATG4D | AC232271.1 | 0.439134936 | 4.93E-25 | postive |
| MYB | AC232271.1 | 0.441170026 | 2.82E-25 | postive |
| TAZ | AC232271.1 | 0.447035324 | 5.51E-26 | postive |
| FANCD2 | AC019171.1 | 0.438930136 | 5.22E-25 | postive |
| HELLS | AC019171.1 | 0.501701947 | 2.70E-33 | postive |
| STMN1 | AC019171.1 | 0.635167533 | 5.95E-58 | postive |
| NOX1 | AC019171.1 | 0.48227657 | 1.52E-30 | postive |
| GLS2 | AC019171.1 | 0.441742478 | 2.41E-25 | postive |
| CDKN2A | AC019171.1 | 0.494347078 | 3.11E-32 | postive |
| TUBE1 | OCIAD1-AS1 | 0.401084383 | 8.72E-21 | postive |
| GPX4 | LINC01534 | 0.418658691 | 1.11E-22 | postive |
| ISCU | LINC01534 | 0.469238176 | 8.52E-29 | postive |
| STMN1 | LINC01534 | 0.46312723 | 5.30E-28 | postive |
| PHKG2 | LINC01534 | 0.432012657 | 3.39E-24 | postive |
| PEBP1 | LINC01534 | 0.412746127 | 4.97E-22 | postive |
| FANCD2 | AC084018.1 | 0.419547849 | 8.85E-23 | postive |
| HELLS | AC084018.1 | 0.488951891 | 1.80E-31 | postive |
| TUBE1 | AC084018.1 | 0.520699161 | 3.68E-36 | postive |
| SP1 | AC084018.1 | 0.425677786 | 1.81E-23 | postive |
| GLS2 | AC084018.1 | 0.443397865 | 1.52E-25 | postive |
| MYB | AC084018.1 | 0.558509502 | 1.96E-42 | postive |
| ACVR1B | AC084018.1 | 0.443277938 | 1.57E-25 | postive |
| LPIN1 | AC084018.1 | 0.419959949 | 7.96E-23 | postive |
| ATM | AC084018.1 | 0.400499504 | 1.00E-20 | postive |
| HELLS | LINC00893 | 0.414325203 | 3.34E-22 | postive |
| TUBE1 | LINC00893 | 0.4891377 | 1.70E-31 | postive |
| TAZ | LINC00893 | 0.466212778 | 2.12E-28 | postive |
| HSPB1 | FOXN3-AS1 | 0.453857071 | 7.94E-27 | postive |
| RPL8 | FOXN3-AS1 | 0.428980799 | 7.58E-24 | postive |
| EGLN2 | FOXN3-AS1 | 0.585481516 | 2.00E-47 | postive |
| CHMP6 | HDAC4-AS1 | 0.435727928 | 1.25E-24 | postive |
| SLC2A8 | HDAC4-AS1 | 0.448010095 | 4.19E-26 | postive |
| PHKG2 | HDAC4-AS1 | 0.635118891 | 6.11E-58 | postive |
| PEBP1 | HDAC4-AS1 | 0.420918855 | 6.22E-23 | postive |
| EGLN2 | HDAC4-AS1 | 0.477532284 | 6.70E-30 | postive |
| TAZ | HDAC4-AS1 | 0.505471158 | 7.53E-34 | postive |
| SP1 | C2CD4D-AS1 | 0.43517367 | 1.45E-24 | postive |
| GLS2 | C2CD4D-AS1 | 0.456001227 | 4.28E-27 | postive |
| YY1AP1 | C2CD4D-AS1 | 0.400136255 | 1.10E-20 | postive |
| TUBE1 | AL050341.2 | 0.438975994 | 5.15E-25 | postive |
| GABPB1 | AL050341.2 | 0.42813184 | 9.49E-24 | postive |
| GABARAPL2 | AL050341.2 | 0.401640241 | 7.63E-21 | postive |
| TMBIM4 | UBR5-AS1 | 0.411362837 | 7.02E-22 | postive |
| EMC2 | UBR5-AS1 | 0.611087634 | 1.29E-52 | postive |
| STMN1 | TFAP2A-AS1 | 0.427540932 | 1.11E-23 | postive |
| PHKG2 | TFAP2A-AS1 | 0.480970659 | 2.29E-30 | postive |
| BRD4 | ACVR2B-AS1 | 0.409753928 | 1.05E-21 | postive |
| KLHL24 | ACVR2B-AS1 | 0.506022525 | 6.24E-34 | postive |
| TUBE1 | ACVR2B-AS1 | 0.489896068 | 1.33E-31 | postive |
| ELAVL1 | ACVR2B-AS1 | 0.483406231 | 1.06E-30 | postive |
| GABPB1 | ACVR2B-AS1 | 0.456791988 | 3.40E-27 | postive |
| GLS2 | ACVR2B-AS1 | 0.460279331 | 1.23E-27 | postive |
| GABARAPL1 | ACVR2B-AS1 | 0.473648714 | 2.22E-29 | postive |
| WIPI1 | ACVR2B-AS1 | 0.414636378 | 3.09E-22 | postive |
| SNX4 | ACVR2B-AS1 | 0.434906323 | 1.56E-24 | postive |
| LPIN1 | ACVR2B-AS1 | 0.409245478 | 1.19E-21 | postive |
| YY1AP1 | ACVR2B-AS1 | 0.46220141 | 6.98E-28 | postive |
| EGLN2 | AC018816.1 | 0.428158149 | 9.43E-24 | postive |
| HIC1 | AC009093.1 | 0.608771207 | 3.97E-52 | postive |
| NOX4 | AC009093.1 | 0.687444152 | 2.47E-71 | postive |
| ZEB1 | AC009093.1 | 0.57495865 | 2.01E-45 | postive |
| TUBE1 | AP002449.1 | 0.462477541 | 6.43E-28 | postive |
| KRAS | AP002449.1 | 0.506820372 | 4.75E-34 | postive |
| PEBP1 | PRRT3-AS1 | 0.436064127 | 1.14E-24 | postive |
| GPX4 | AC010331.1 | 0.486235885 | 4.31E-31 | postive |
| ISCU | AC010331.1 | 0.403773511 | 4.55E-21 | postive |
| SLC2A8 | AC010331.1 | 0.461850695 | 7.74E-28 | postive |
| ACSF2 | AC010331.1 | 0.402934929 | 5.58E-21 | postive |
| STMN1 | AC010331.1 | 0.400088371 | 1.11E-20 | postive |
| PHKG2 | AC010331.1 | 0.569883193 | 1.75E-44 | postive |
| SOCS1 | AC010331.1 | 0.413592732 | 4.01E-22 | postive |
| EGLN2 | AC010331.1 | 0.580358259 | 1.93E-46 | postive |
| TAZ | AC010331.1 | 0.536603243 | 1.06E-38 | postive |
| RPL8 | AC112491.1 | 0.477461116 | 6.85E-30 | postive |
| EGLN2 | AC112491.1 | 0.444000564 | 1.29E-25 | postive |
| TUBE1 | AC007292.1 | 0.442940876 | 1.73E-25 | postive |
| ELAVL1 | AC007292.1 | 0.483829818 | 9.28E-31 | postive |
| NOX1 | AC007292.1 | 0.430938604 | 4.51E-24 | postive |
| PHKG2 | AC007292.1 | 0.572369034 | 6.10E-45 | postive |
| ATG4D | AC007292.1 | 0.43488427 | 1.57E-24 | postive |
| PEBP1 | AC007292.1 | 0.400459846 | 1.01E-20 | postive |
| SOCS1 | AC007292.1 | 0.465642955 | 2.51E-28 | postive |
| TAZ | AC007292.1 | 0.439513703 | 4.45E-25 | postive |
| ISCU | AC048341.2 | 0.458786577 | 1.90E-27 | postive |
| STMN1 | AC048341.2 | 0.410843264 | 7.99E-22 | postive |
| PHKG2 | AC048341.2 | 0.460484644 | 1.16E-27 | postive |
| MYB | AC048341.2 | 0.434095914 | 1.94E-24 | postive |
| TAZ | AC048341.2 | 0.441970463 | 2.26E-25 | postive |
| HELLS | AC102953.2 | 0.40469421 | 3.64E-21 | postive |
| GPX4 | AC027644.3 | 0.42438572 | 2.53E-23 | postive |
| RPL8 | AC027644.3 | 0.411349804 | 7.04E-22 | postive |
| PHKG2 | AC027644.3 | 0.556893406 | 3.78E-42 | postive |
| WIPI2 | AC027644.3 | 0.403616434 | 4.73E-21 | postive |
| EGLN2 | AC027644.3 | 0.623232561 | 3.00E-55 | postive |
| TAZ | AC027644.3 | 0.467262204 | 1.54E-28 | postive |
| GPX4 | AC024060.2 | 0.520046938 | 4.64E-36 | postive |
| CISD1 | AC024060.2 | 0.431899569 | 3.49E-24 | postive |
| FANCD2 | AC024060.2 | 0.495813781 | 1.92E-32 | postive |
| ATF4 | AC024060.2 | 0.467921982 | 1.27E-28 | postive |
| HELLS | AC024060.2 | 0.446847402 | 5.81E-26 | postive |
| ISCU | AC024060.2 | 0.596283096 | 1.47E-49 | postive |
| HMGB1 | AC024060.2 | 0.456432801 | 3.77E-27 | postive |
| STMN1 | AC024060.2 | 0.610443464 | 1.76E-52 | postive |
| NOX1 | AC024060.2 | 0.575244721 | 1.78E-45 | postive |
| PHKG2 | AC024060.2 | 0.546082378 | 2.77E-40 | postive |
| MAP1LC3A | AC024060.2 | 0.431057821 | 4.37E-24 | postive |
| PEBP1 | AC024060.2 | 0.480171157 | 2.94E-30 | postive |
| SOCS1 | AC024060.2 | 0.43432158 | 1.82E-24 | postive |
| MYB | AC024060.2 | 0.43723436 | 8.28E-25 | postive |
| EGLN2 | AC024060.2 | 0.404284874 | 4.02E-21 | postive |
| TAZ | AC024060.2 | 0.52262573 | 1.84E-36 | postive |
| GPX4 | MHENCR | 0.40166573 | 7.58E-21 | postive |
| RPL8 | MHENCR | 0.454568361 | 6.47E-27 | postive |
| SLC2A8 | MHENCR | 0.425202145 | 2.05E-23 | postive |
| PHKG2 | MHENCR | 0.644528269 | 3.72E-60 | postive |
| PEBP1 | MHENCR | 0.470464686 | 5.87E-29 | postive |
| ANO6 | MHENCR | -0.428785176 | 7.99E-24 | negative |
| EGLN2 | MHENCR | 0.511066709 | 1.10E-34 | postive |
| TAZ | MHENCR | 0.600217202 | 2.35E-50 | postive |
| HIC1 | RASSF8-AS1 | 0.421290155 | 5.66E-23 | postive |
| NOX4 | RASSF8-AS1 | 0.403647257 | 4.69E-21 | postive |
| ZEB1 | RASSF8-AS1 | 0.454131725 | 7.33E-27 | postive |
| SOCS1 | AC055854.1 | 0.416268279 | 2.04E-22 | postive |
| GPX4 | AC008915.3 | 0.476763267 | 8.51E-30 | postive |
| CISD1 | AC008915.3 | 0.409157621 | 1.21E-21 | postive |
| CHMP6 | AC008915.3 | 0.489176621 | 1.68E-31 | postive |
| PHKG2 | AC008915.3 | 0.606637655 | 1.11E-51 | postive |
| MAP1LC3A | AC008915.3 | 0.508917917 | 2.31E-34 | postive |
| EGLN2 | AC008915.3 | 0.470407227 | 5.98E-29 | postive |
| SP1 | AC098487.1 | -0.400338515 | 1.04E-20 | negative |
| CHMP6 | LINC02361 | 0.40568874 | 2.85E-21 | postive |
| PHKG2 | LINC02361 | 0.435775552 | 1.23E-24 | postive |
| PANX1 | NOP14-AS1 | 0.428648632 | 8.28E-24 | postive |
| PHKG2 | SNHG19 | 0.573718965 | 3.43E-45 | postive |
| MAP1LC3A | SNHG19 | 0.410642784 | 8.40E-22 | postive |
| EGLN2 | SNHG19 | 0.445898616 | 7.58E-26 | postive |
| TAZ | SNHG19 | 0.404652713 | 3.67E-21 | postive |
| STMN1 | AC011297.1 | 0.444840295 | 1.02E-25 | postive |
| GPX4 | AC016773.2 | 0.450130919 | 2.30E-26 | postive |
| ATF4 | AC016773.2 | 0.439049107 | 5.05E-25 | postive |
| ISCU | AC016773.2 | 0.489380179 | 1.57E-31 | postive |
| SLC2A8 | AC016773.2 | 0.432109638 | 3.30E-24 | postive |
| STMN1 | AC016773.2 | 0.520491813 | 3.96E-36 | postive |
| NOX1 | AC016773.2 | 0.510326876 | 1.42E-34 | postive |
| PHKG2 | AC016773.2 | 0.585385557 | 2.09E-47 | postive |
| SOCS1 | AC016773.2 | 0.430880864 | 4.58E-24 | postive |
| EGLN2 | AC016773.2 | 0.44001541 | 3.87E-25 | postive |
| TAZ | AC016773.2 | 0.581795958 | 1.02E-46 | postive |
| ELAVL1 | AC008966.1 | 0.418113117 | 1.28E-22 | postive |
| ATF4 | LINC01311 | 0.456516594 | 3.68E-27 | postive |
| RPL8 | LINC01311 | 0.409486287 | 1.12E-21 | postive |
| PHKG2 | LINC01311 | 0.582240911 | 8.42E-47 | postive |
| EGLN2 | LINC01311 | 0.485385628 | 5.66E-31 | postive |
| TAZ | LINC01311 | 0.574875581 | 2.08E-45 | postive |
| ALOX5 | AC018755.4 | 0.405910038 | 2.70E-21 | postive |
| FLT3 | AC018755.4 | 0.42170866 | 5.08E-23 | postive |
| SETD1B | AC090198.1 | 0.401460099 | 7.97E-21 | postive |
| SP1 | AC090198.1 | 0.419194479 | 9.69E-23 | postive |
| ACVR1B | AC090198.1 | 0.410997596 | 7.69E-22 | postive |
| SIRT1 | AC090198.1 | 0.424561927 | 2.42E-23 | postive |
| CDKN2A | CDKN2B-AS1 | 0.434820653 | 1.59E-24 | postive |
| HELLS | AC004803.1 | 0.418250951 | 1.23E-22 | postive |
| LPCAT3 | AC004803.1 | 0.438207285 | 6.36E-25 | postive |
| MYB | AC004803.1 | 0.492820985 | 5.13E-32 | postive |
| NFE2L2 | AC128709.3 | 0.447719044 | 4.55E-26 | postive |
| GPX4 | AC026979.2 | 0.443906645 | 1.32E-25 | postive |
| RPL8 | AC026979.2 | 0.499787018 | 5.13E-33 | postive |
| SLC2A8 | AC026979.2 | 0.491099177 | 8.99E-32 | postive |
| PHKG2 | AC026979.2 | 0.607533605 | 7.22E-52 | postive |
| EGLN2 | AC026979.2 | 0.646677578 | 1.13E-60 | postive |
| TAZ | AC026979.2 | 0.475468952 | 1.27E-29 | postive |
| LONP1 | AC026979.2 | 0.422845213 | 3.78E-23 | postive |
| ASNS | AF131216.3 | 0.416181244 | 2.09E-22 | postive |
| PCK2 | AF131216.3 | 0.405559936 | 2.94E-21 | postive |
| PSAT1 | AF131216.3 | 0.438916981 | 5.23E-25 | postive |
| FANCD2 | AF131215.5 | 0.493950616 | 3.54E-32 | postive |
| TP53 | AF131215.5 | 0.498931416 | 6.82E-33 | postive |
| HELLS | AF131215.5 | 0.565207912 | 1.25E-43 | postive |
| STAT3 | AF131215.5 | 0.412755973 | 4.95E-22 | postive |
| AGPAT3 | AF131215.5 | 0.405468915 | 3.01E-21 | postive |
| SETD1B | AF131215.5 | 0.426080357 | 1.63E-23 | postive |
| SP1 | AF131215.5 | 0.590509971 | 2.08E-48 | postive |
| FLT3 | AF131215.5 | 0.41837626 | 1.19E-22 | postive |
| GLS2 | AF131215.5 | 0.564147107 | 1.94E-43 | postive |
| MYB | AF131215.5 | 0.418851857 | 1.06E-22 | postive |
| MAPK8 | AF131215.5 | 0.458837262 | 1.87E-27 | postive |
| ACVR1B | AF131215.5 | 0.425516072 | 1.89E-23 | postive |
| ATM | AF131215.5 | 0.433673258 | 2.17E-24 | postive |
| SIRT1 | AF131215.5 | 0.438527998 | 5.82E-25 | postive |
| ISCU | AC090948.1 | 0.502555313 | 2.02E-33 | postive |
| NOX1 | AC090948.1 | 0.407650153 | 1.76E-21 | postive |
| IFNG | AC090948.1 | 0.400997914 | 8.91E-21 | postive |
| CD44 | AL133330.1 | 0.48189152 | 1.71E-30 | postive |
| FANCD2 | MIR924HG | 0.518116065 | 9.24E-36 | postive |
| TP53 | MIR924HG | 0.441010517 | 2.95E-25 | postive |
| HELLS | MIR924HG | 0.638594362 | 9.47E-59 | postive |
| GCH1 | MIR924HG | 0.431114824 | 4.30E-24 | postive |
| ELAVL1 | MIR924HG | 0.415161266 | 2.70E-22 | postive |
| STMN1 | MIR924HG | 0.569570417 | 2.00E-44 | postive |
| GLS2 | MIR924HG | 0.508500766 | 2.67E-34 | postive |
| CDKN2A | MIR924HG | 0.419417134 | 9.15E-23 | postive |
| MAPK8 | MIR924HG | 0.468565401 | 1.04E-28 | postive |
| ATF4 | AL031186.1 | 0.401112301 | 8.66E-21 | postive |
| ZNF419 | AL031186.1 | 0.411168678 | 7.36E-22 | postive |
| TUBE1 | AL031186.1 | 0.518305967 | 8.64E-36 | postive |
| MYB | AL031186.1 | 0.406224955 | 2.50E-21 | postive |
| TAZ | AL031186.1 | 0.421533829 | 5.31E-23 | postive |
| BRD4 | LOXL1-AS1 | 0.427594373 | 1.09E-23 | postive |
| KLHL24 | LOXL1-AS1 | 0.453861581 | 7.93E-27 | postive |
| TUBE1 | LOXL1-AS1 | 0.454904001 | 5.87E-27 | postive |
| ELAVL1 | LOXL1-AS1 | 0.419424929 | 9.14E-23 | postive |
| GABPB1 | LOXL1-AS1 | 0.419826743 | 8.24E-23 | postive |
| GABARAPL1 | LOXL1-AS1 | 0.456967307 | 3.23E-27 | postive |
| GPX4 | ENTPD3-AS1 | 0.51722756 | 1.27E-35 | postive |
| HSPB1 | ENTPD3-AS1 | 0.44050143 | 3.39E-25 | postive |
| CISD1 | ENTPD3-AS1 | 0.44488623 | 1.01E-25 | postive |
| HIF1A | ENTPD3-AS1 | -0.414540782 | 3.16E-22 | negative |
| RPL8 | ENTPD3-AS1 | 0.471447204 | 4.36E-29 | postive |
| SLC2A8 | ENTPD3-AS1 | 0.441379793 | 2.66E-25 | postive |
| NOX1 | ENTPD3-AS1 | 0.433619982 | 2.20E-24 | postive |
| HRAS | ENTPD3-AS1 | 0.409918745 | 1.01E-21 | postive |
| PHKG2 | ENTPD3-AS1 | 0.591245651 | 1.49E-48 | postive |
| ANO6 | ENTPD3-AS1 | -0.466954379 | 1.69E-28 | negative |
| EGLN2 | ENTPD3-AS1 | 0.68814296 | 1.57E-71 | postive |
| TAZ | ENTPD3-AS1 | 0.509872811 | 1.66E-34 | postive |
| AKR1C1 | COA6-AS1 | 0.414115186 | 3.52E-22 | postive |
| AKR1C3 | COA6-AS1 | 0.430827266 | 4.65E-24 | postive |
| PRDX6 | COA6-AS1 | 0.44658381 | 6.26E-26 | postive |
| GPX2 | COA6-AS1 | 0.489581956 | 1.47E-31 | postive |
| PRDX1 | COA6-AS1 | 0.456270208 | 3.96E-27 | postive |
| PHKG2 | COA6-AS1 | 0.470938867 | 5.08E-29 | postive |
| BID | COA6-AS1 | 0.429440922 | 6.71E-24 | postive |
| TAZ | COA6-AS1 | 0.444188876 | 1.22E-25 | postive |
| LONP1 | COA6-AS1 | 0.407187769 | 1.97E-21 | postive |
| TUBE1 | AC114730.3 | 0.41913082 | 9.85E-23 | postive |
| PHKG2 | CD2BP2-DT | 0.442581772 | 1.91E-25 | postive |
| FANCD2 | AC091057.1 | 0.614152495 | 2.86E-53 | postive |
| HELLS | AC091057.1 | 0.701909924 | 1.49E-75 | postive |
| GCH1 | AC091057.1 | 0.451050867 | 1.77E-26 | postive |
| SETD1B | AC091057.1 | 0.419819263 | 8.26E-23 | postive |
| HMGB1 | AC091057.1 | 0.42672659 | 1.37E-23 | postive |
| ELAVL1 | AC091057.1 | 0.492321572 | 6.04E-32 | postive |
| SP1 | AC091057.1 | 0.548670947 | 1.01E-40 | postive |
| STMN1 | AC091057.1 | 0.535942176 | 1.35E-38 | postive |
| RRM2 | AC091057.1 | 0.5862026 | 1.45E-47 | postive |
| GABPB1 | AC091057.1 | 0.469548616 | 7.75E-29 | postive |
| AURKA | AC091057.1 | 0.441600284 | 2.50E-25 | postive |
| GLS2 | AC091057.1 | 0.500834707 | 3.61E-33 | postive |
| ATG3 | AC091057.1 | 0.43560782 | 1.29E-24 | postive |
| SNX4 | AC091057.1 | 0.475616547 | 1.21E-29 | postive |
| MAPK8 | AC091057.1 | 0.467665833 | 1.37E-28 | postive |
| MAPK9 | AC091057.1 | 0.422334173 | 4.32E-23 | postive |
| ACVR1B | AC091057.1 | 0.415459686 | 2.51E-22 | postive |
| YY1AP1 | AC091057.1 | 0.474623127 | 1.65E-29 | postive |
| SIRT1 | AC091057.1 | 0.422115139 | 4.57E-23 | postive |
| TUBE1 | AC009237.14 | 0.417750056 | 1.40E-22 | postive |
| RPL8 | MIR4435-2HG | 0.455502807 | 4.94E-27 | postive |
| NNMT | MIR4435-2HG | 0.498386774 | 8.18E-33 | postive |
| EGLN2 | MIR4435-2HG | 0.489294694 | 1.61E-31 | postive |
| FANCD2 | AL391001.1 | 0.42881996 | 7.91E-24 | postive |
| HELLS | AL391001.1 | 0.475967621 | 1.09E-29 | postive |
| STMN1 | AL391001.1 | 0.490853054 | 9.74E-32 | postive |
| GLS2 | AL391001.1 | 0.416447213 | 1.95E-22 | postive |
| KLHL24 | AC022916.2 | 0.402726448 | 5.87E-21 | postive |
| ELAVL1 | AC022916.2 | 0.407932182 | 1.64E-21 | postive |
| HELLS | ZNF436-AS1 | 0.534571966 | 2.27E-38 | postive |
| ISCU | ZNF436-AS1 | 0.519254536 | 6.16E-36 | postive |
| TUBE1 | ZNF436-AS1 | 0.470888875 | 5.16E-29 | postive |
| ELAVL1 | ZNF436-AS1 | 0.416251146 | 2.05E-22 | postive |
| STMN1 | ZNF436-AS1 | 0.512830494 | 5.95E-35 | postive |
| NOX1 | ZNF436-AS1 | 0.419303628 | 9.42E-23 | postive |
| GLS2 | ZNF436-AS1 | 0.474836602 | 1.54E-29 | postive |
| ATG4D | ZNF436-AS1 | 0.424430342 | 2.51E-23 | postive |
| MYB | ZNF436-AS1 | 0.532122205 | 5.67E-38 | postive |
| MAPK8 | ZNF436-AS1 | 0.410102326 | 9.60E-22 | postive |
| TAZ | ZNF436-AS1 | 0.403105096 | 5.35E-21 | postive |
| FANCD2 | AC007066.2 | 0.429455149 | 6.69E-24 | postive |
| HELLS | AC007066.2 | 0.490690493 | 1.03E-31 | postive |
| ISCU | AC007066.2 | 0.411632615 | 6.56E-22 | postive |
| TUBE1 | AC007066.2 | 0.454000371 | 7.62E-27 | postive |
| SLC2A8 | AC007066.2 | 0.405055339 | 3.33E-21 | postive |
| ELAVL1 | AC007066.2 | 0.440700328 | 3.21E-25 | postive |
| STMN1 | AC007066.2 | 0.500542834 | 3.98E-33 | postive |
| GABPB1 | AC007066.2 | 0.419680523 | 8.56E-23 | postive |
| PHKG2 | AC007066.2 | 0.422690755 | 3.94E-23 | postive |
| ATG3 | AC007066.2 | 0.408249767 | 1.52E-21 | postive |
| TAZ | AC007066.2 | 0.402227708 | 6.62E-21 | postive |
| EGLN2 | AC084809.2 | 0.494280755 | 3.18E-32 | postive |
| EMC2 | OTUD6B-AS1 | 0.56410606 | 1.97E-43 | postive |
| PRKAA1 | OTUD6B-AS1 | 0.428134915 | 9.48E-24 | postive |
| MTDH | OTUD6B-AS1 | 0.42020194 | 7.48E-23 | postive |
| TP53 | BHLHE40-AS1 | 0.407320817 | 1.91E-21 | postive |
| HELLS | BHLHE40-AS1 | 0.42478068 | 2.29E-23 | postive |
| GCH1 | BHLHE40-AS1 | 0.435649603 | 1.27E-24 | postive |
| ATG7 | BHLHE40-AS1 | 0.419175849 | 9.74E-23 | postive |
| MAPK8 | BHLHE40-AS1 | 0.415898857 | 2.24E-22 | postive |
| IFNG | BHLHE40-AS1 | 0.430041494 | 5.73E-24 | postive |
| CAV1 | LINC00911 | 0.408398058 | 1.46E-21 | postive |
| HELLS | AL450998.2 | 0.400741228 | 9.47E-21 | postive |
| STMN1 | AL450998.2 | 0.515302862 | 2.50E-35 | postive |
| ZFP69B | AL031985.3 | 0.797621632 | 1.22E-111 | postive |
| TUBE1 | AL031985.3 | 0.441879478 | 2.32E-25 | postive |
| ELAVL1 | AL031985.3 | 0.427131316 | 1.24E-23 | postive |
| MYB | AL031985.3 | 0.415206164 | 2.67E-22 | postive |
| ATM | AL031985.3 | 0.450042697 | 2.36E-26 | postive |
| GPX4 | AC019131.2 | 0.457574198 | 2.71E-27 | postive |
| HELLS | AC019131.2 | 0.427419023 | 1.15E-23 | postive |
| ISCU | AC019131.2 | 0.531377019 | 7.47E-38 | postive |
| STMN1 | AC019131.2 | 0.465080296 | 2.97E-28 | postive |
| NOX1 | AC019131.2 | 0.572382637 | 6.06E-45 | postive |
| GLS2 | AC019131.2 | 0.448679651 | 3.47E-26 | postive |
| ATG4D | AC019131.2 | 0.468037699 | 1.22E-28 | postive |
| MAP1LC3A | AC019131.2 | 0.42295983 | 3.67E-23 | postive |
| ZNF419 | ZNF528-AS1 | 0.505766217 | 6.81E-34 | postive |
| TAZ | AC016394.3 | 0.43605353 | 1.14E-24 | postive |
| CEBPG | AC133919.2 | 0.421618565 | 5.20E-23 | postive |
| CISD1 | AC015722.2 | 0.44273363 | 1.83E-25 | postive |
| TUBE1 | STAG3L5P-PVRIG2P-PILRB | 0.428972731 | 7.60E-24 | postive |
| PHKG2 | STAG3L5P-PVRIG2P-PILRB | 0.459984382 | 1.34E-27 | postive |
| TAZ | STAG3L5P-PVRIG2P-PILRB | 0.47539597 | 1.30E-29 | postive |
| GPX4 | LINC01023 | 0.479615472 | 3.50E-30 | postive |
| RB1 | LINC01023 | -0.424295135 | 2.60E-23 | negative |
| HSPB1 | LINC01023 | 0.484030643 | 8.70E-31 | postive |
| CHMP6 | LINC01023 | 0.43484336 | 1.58E-24 | postive |
| RPL8 | LINC01023 | 0.477168481 | 7.50E-30 | postive |
| SLC2A8 | LINC01023 | 0.432596135 | 2.90E-24 | postive |
| NRAS | LINC01023 | -0.401114324 | 8.66E-21 | negative |
| PHKG2 | LINC01023 | 0.600626466 | 1.94E-50 | postive |
| ANO6 | LINC01023 | -0.451372992 | 1.62E-26 | negative |
| EGLN2 | LINC01023 | 0.69296906 | 6.47E-73 | postive |
| TAZ | LINC01023 | 0.534479227 | 2.35E-38 | postive |
| LONP1 | LINC01023 | 0.409816493 | 1.03E-21 | postive |
| TUBE1 | AL022328.2 | 0.465014903 | 3.03E-28 | postive |
| PHKG2 | AL022328.2 | 0.481078775 | 2.21E-30 | postive |
| TAZ | AL022328.2 | 0.502646531 | 1.96E-33 | postive |
| CHMP6 | AP001363.2 | 0.414753878 | 3.00E-22 | postive |
| MAPK9 | AC008906.1 | 0.457597909 | 2.69E-27 | postive |
| GPX4 | IDH1-AS1 | 0.404620282 | 3.70E-21 | postive |
| PHKG2 | IDH1-AS1 | 0.497693875 | 1.03E-32 | postive |
| EGLN2 | IDH1-AS1 | 0.486105541 | 4.49E-31 | postive |
| TAZ | IDH1-AS1 | 0.446450655 | 6.50E-26 | postive |
| FANCD2 | AC100861.1 | 0.531152422 | 8.12E-38 | postive |
| TP53 | AC100861.1 | 0.438763533 | 5.46E-25 | postive |
| HELLS | AC100861.1 | 0.554019568 | 1.20E-41 | postive |
| ISCU | AC100861.1 | 0.404327014 | 3.98E-21 | postive |
| GCH1 | AC100861.1 | 0.41287224 | 4.81E-22 | postive |
| ELAVL1 | AC100861.1 | 0.449031466 | 3.14E-26 | postive |
| SP1 | AC100861.1 | 0.428538384 | 8.53E-24 | postive |
| CS | AC100861.1 | 0.415323574 | 2.60E-22 | postive |
| GLS2 | AC100861.1 | 0.574560591 | 2.39E-45 | postive |
| MAPK8 | AC100861.1 | 0.429269797 | 7.03E-24 | postive |
| ISCU | ZNF460-AS1 | 0.487908909 | 2.52E-31 | postive |
| ZNF419 | ZNF460-AS1 | 0.440255641 | 3.63E-25 | postive |
| NOX1 | ZNF460-AS1 | 0.469949574 | 6.87E-29 | postive |
| PHKG2 | ZNF460-AS1 | 0.489619478 | 1.45E-31 | postive |
| TAZ | ZNF460-AS1 | 0.468453185 | 1.08E-28 | postive |
| GPX4 | AL133215.2 | 0.415659863 | 2.38E-22 | postive |
| HELLS | AL133215.2 | 0.45584897 | 4.47E-27 | postive |
| ISCU | AL133215.2 | 0.434424679 | 1.77E-24 | postive |
| SLC2A8 | AL133215.2 | 0.467284189 | 1.53E-28 | postive |
| HMGB1 | AL133215.2 | 0.412771 | 4.93E-22 | postive |
| STMN1 | AL133215.2 | 0.596014214 | 1.67E-49 | postive |
| NOX1 | AL133215.2 | 0.462038066 | 7.32E-28 | postive |
| PHKG2 | AL133215.2 | 0.491605515 | 7.62E-32 | postive |
| PEBP1 | AL133215.2 | 0.461672572 | 8.15E-28 | postive |
| EGLN2 | AL133215.2 | 0.403132246 | 5.32E-21 | postive |
| TAZ | AL133215.2 | 0.523097222 | 1.55E-36 | postive |
| PHKG2 | LINC02846 | 0.400584822 | 9.84E-21 | postive |
| ELAVL1 | AC008649.1 | 0.405001448 | 3.37E-21 | postive |
| SOCS1 | AC008649.1 | 0.449503164 | 2.75E-26 | postive |
| STMN1 | TNKS2-AS1 | 0.453535059 | 8.70E-27 | postive |
| PHKG2 | TNKS2-AS1 | 0.422673625 | 3.96E-23 | postive |
| TAZ | TNKS2-AS1 | 0.453999705 | 7.62E-27 | postive |
| AIFM2 | AC004034.1 | 0.404074011 | 4.23E-21 | postive |
| MT3 | AC004034.1 | 0.472979326 | 2.73E-29 | postive |
| CDO1 | AC004034.1 | 0.427074387 | 1.25E-23 | postive |
| MIOX | AC004034.1 | 0.430240151 | 5.43E-24 | postive |
| NOX1 | AC107214.2 | 0.447964136 | 4.25E-26 | postive |
| GPX4 | LINC01006 | 0.438972095 | 5.16E-25 | postive |
| FANCD2 | LINC01006 | 0.431470612 | 3.91E-24 | postive |
| TP53 | LINC01006 | 0.402106267 | 6.82E-21 | postive |
| HELLS | LINC01006 | 0.492828395 | 5.12E-32 | postive |
| ISCU | LINC01006 | 0.419085455 | 9.97E-23 | postive |
| ELAVL1 | LINC01006 | 0.40616423 | 2.54E-21 | postive |
| STMN1 | LINC01006 | 0.587216103 | 9.20E-48 | postive |
| NOX1 | LINC01006 | 0.425282897 | 2.01E-23 | postive |
| GLS2 | LINC01006 | 0.493142461 | 4.62E-32 | postive |
| MAP1LC3A | LINC01006 | 0.46073867 | 1.07E-27 | postive |
| CDKN2A | LINC01006 | 0.418403858 | 1.19E-22 | postive |
| YY1AP1 | LINC01006 | 0.425935067 | 1.69E-23 | postive |
| LPCAT3 | AC006064.2 | 0.533978473 | 2.83E-38 | postive |
| RPL8 | AC055822.1 | 0.422298211 | 4.36E-23 | postive |
| ACSF2 | AC055822.1 | 0.419906078 | 8.08E-23 | postive |
| HRAS | AC055822.1 | 0.40336289 | 5.03E-21 | postive |
| STAT3 | AC090587.2 | 0.422383473 | 4.26E-23 | postive |
| SETD1B | AC090587.2 | 0.537283986 | 8.16E-39 | postive |
| IREB2 | AC090587.2 | 0.415290012 | 2.62E-22 | postive |
| SP1 | AC090587.2 | 0.466105364 | 2.19E-28 | postive |
| PRKAA1 | C5orf34-AS1 | 0.404562409 | 3.75E-21 | postive |
| PML | TTLL11-IT1 | 0.44713891 | 5.36E-26 | postive |
| CAV1 | TTLL11-IT1 | 0.513307874 | 5.03E-35 | postive |
| ENPP2 | AC015922.2 | 0.400320785 | 1.05E-20 | postive |
| ZEB1 | AC015922.2 | 0.435039259 | 1.50E-24 | postive |
| TF | AC103563.7 | 0.46034679 | 1.20E-27 | postive |
| FANCD2 | AL355488.1 | 0.561299177 | 6.27E-43 | postive |
| HELLS | AL355488.1 | 0.640135505 | 4.11E-59 | postive |
| ISCU | AL355488.1 | 0.512939584 | 5.72E-35 | postive |
| TSC22D3 | AL355488.1 | 0.400690667 | 9.59E-21 | postive |
| TUBE1 | AL355488.1 | 0.426557565 | 1.44E-23 | postive |
| HMGB1 | AL355488.1 | 0.457986494 | 2.40E-27 | postive |
| ELAVL1 | AL355488.1 | 0.411396502 | 6.96E-22 | postive |
| STMN1 | AL355488.1 | 0.615613246 | 1.39E-53 | postive |
| GABPB1 | AL355488.1 | 0.423961442 | 2.83E-23 | postive |
| NOX1 | AL355488.1 | 0.479031284 | 4.20E-30 | postive |
| GLS2 | AL355488.1 | 0.470153621 | 6.45E-29 | postive |
| PHKG2 | AL355488.1 | 0.406252525 | 2.48E-21 | postive |
| ATG3 | AL355488.1 | 0.408233306 | 1.53E-21 | postive |
| ATG4D | AL355488.1 | 0.401656262 | 7.60E-21 | postive |
| SOCS1 | AL355488.1 | 0.404866837 | 3.49E-21 | postive |
| MYB | AL355488.1 | 0.518533288 | 7.97E-36 | postive |
| TAZ | AL355488.1 | 0.436909333 | 9.05E-25 | postive |
| ATF4 | AL021707.6 | 0.462432562 | 6.52E-28 | postive |
| RPL8 | AL021707.6 | 0.445379181 | 8.77E-26 | postive |
| SLC2A8 | AL021707.6 | 0.481822738 | 1.75E-30 | postive |
| CAPG | AL021707.6 | 0.427261045 | 1.19E-23 | postive |
| NOX1 | AL021707.6 | 0.468297928 | 1.13E-28 | postive |
| HRAS | AL021707.6 | 0.498291784 | 8.44E-33 | postive |
| PHKG2 | AL021707.6 | 0.628216826 | 2.31E-56 | postive |
| ANO6 | AL021707.6 | -0.440210574 | 3.67E-25 | negative |
| EGLN2 | AL021707.6 | 0.689110795 | 8.30E-72 | postive |
| TAZ | AL021707.6 | 0.504156588 | 1.18E-33 | postive |
| PML | AL596244.1 | 0.469390486 | 8.13E-29 | postive |
| CAV1 | AL596244.1 | 0.454069091 | 7.47E-27 | postive |
| MYB | AC005674.2 | 0.439365172 | 4.63E-25 | postive |
| GPX4 | IRF1-AS1 | 0.433914667 | 2.03E-24 | postive |
| CISD1 | IRF1-AS1 | 0.46093205 | 1.01E-27 | postive |
| ISCU | IRF1-AS1 | 0.621071061 | 8.99E-55 | postive |
| GCH1 | IRF1-AS1 | 0.423872759 | 2.90E-23 | postive |
| FLT3 | IRF1-AS1 | 0.436275197 | 1.07E-24 | postive |
| CARS1 | IRF1-AS1 | 0.476065446 | 1.06E-29 | postive |
| ATG7 | IRF1-AS1 | 0.433691551 | 2.16E-24 | postive |
| SOCS1 | IRF1-AS1 | 0.499939197 | 4.87E-33 | postive |
| IFNG | IRF1-AS1 | 0.561675613 | 5.37E-43 | postive |
| FANCD2 | AL021878.2 | 0.417956022 | 1.33E-22 | postive |
| HELLS | AL021878.2 | 0.443702539 | 1.40E-25 | postive |
| BRD4 | AL021878.2 | 0.403125467 | 5.32E-21 | postive |
| TUBE1 | AL021878.2 | 0.513592491 | 4.55E-35 | postive |
| GLS2 | AL021878.2 | 0.407447539 | 1.85E-21 | postive |
| PHKG2 | AC135050.3 | 0.539254602 | 3.86E-39 | postive |
| TAZ | AC135050.3 | 0.405037465 | 3.34E-21 | postive |
| NOS2 | AL354754.1 | 0.462429389 | 6.52E-28 | postive |
| HELLS | AC016877.3 | 0.439658888 | 4.27E-25 | postive |
| STMN1 | AC016877.3 | 0.505500857 | 7.45E-34 | postive |
| FANCD2 | AC025171.2 | 0.40323197 | 5.19E-21 | postive |
| HELLS | AC025171.2 | 0.413822996 | 3.79E-22 | postive |
| ISCU | AC025171.2 | 0.410485816 | 8.73E-22 | postive |
| SETD1B | AC025171.2 | 0.402781934 | 5.79E-21 | postive |
| SP1 | AC025171.2 | 0.480521461 | 2.63E-30 | postive |
| GLS2 | AC025171.2 | 0.419363697 | 9.28E-23 | postive |
| MAPK8 | AC025171.2 | 0.402629841 | 6.00E-21 | postive |
| HELLS | AL390728.6 | 0.430690815 | 4.82E-24 | postive |
| ISCU | AL390728.6 | 0.448740927 | 3.41E-26 | postive |
| TUBE1 | AL390728.6 | 0.485525417 | 5.41E-31 | postive |
| HMGB1 | AL390728.6 | 0.457481552 | 2.78E-27 | postive |
| STMN1 | AL390728.6 | 0.44256092 | 1.92E-25 | postive |
| GABPB1 | AL390728.6 | 0.419243907 | 9.57E-23 | postive |
| NOX1 | AL390728.6 | 0.451667617 | 1.49E-26 | postive |
| PHKG2 | AL390728.6 | 0.419066785 | 1.00E-22 | postive |
| TAZ | AL390728.6 | 0.446825067 | 5.85E-26 | postive |
| NNMT | AC068506.1 | 0.473964439 | 2.02E-29 | postive |
| PLIN4 | AC068506.1 | 0.631118759 | 5.07E-57 | postive |
| PRKAA2 | AC068506.1 | 0.866736913 | 7.33E-153 | postive |
| GPX4 | AC008622.2 | 0.417092073 | 1.66E-22 | postive |
| ISCU | AC008622.2 | 0.411550502 | 6.70E-22 | postive |
| PRDX6 | AC008622.2 | 0.407352543 | 1.90E-21 | postive |
| NOX1 | AC008622.2 | 0.503150828 | 1.65E-33 | postive |
| SLC1A5 | AC008622.2 | 0.557416757 | 3.06E-42 | postive |
| PHKG2 | AC008622.2 | 0.496076194 | 1.76E-32 | postive |
| EGLN2 | AC008622.2 | 0.433107547 | 2.53E-24 | postive |
| TAZ | AC008622.2 | 0.49021497 | 1.20E-31 | postive |
| ISCU | AL353708.3 | 0.436387664 | 1.04E-24 | postive |
| STMN1 | AL353708.3 | 0.482821255 | 1.28E-30 | postive |
| NOX1 | AL353708.3 | 0.479963167 | 3.14E-30 | postive |
| PHKG2 | AL353708.3 | 0.480311725 | 2.81E-30 | postive |
| TAZ | AL353708.3 | 0.461203765 | 9.36E-28 | postive |
| SLC7A11 | AC145207.8 | 0.486321401 | 4.19E-31 | postive |
| AKR1C1 | AC145207.8 | 0.496409548 | 1.58E-32 | postive |
| AKR1C3 | AC145207.8 | 0.517588453 | 1.11E-35 | postive |
| GCLC | AC145207.8 | 0.440081173 | 3.81E-25 | postive |
| NQO1 | AC145207.8 | 0.422788568 | 3.84E-23 | postive |
| PRDX6 | AC145207.8 | 0.402152589 | 6.74E-21 | postive |
| TXNRD1 | AC145207.8 | 0.662470812 | 1.32E-64 | postive |
| SRXN1 | AC145207.8 | 0.641987629 | 1.50E-59 | postive |
| GPX2 | AC145207.8 | 0.521278814 | 2.99E-36 | postive |
| FTL | AC145207.8 | 0.438044807 | 6.64E-25 | postive |
| MAFG | AC145207.8 | 0.618401998 | 3.45E-54 | postive |
| G6PD | AC145207.8 | 0.57744034 | 6.88E-46 | postive |
| PGD | AC145207.8 | 0.54854728 | 1.06E-40 | postive |
| ABCC1 | AC145207.8 | 0.644380366 | 4.03E-60 | postive |
| IDH1 | AC145207.8 | 0.489167351 | 1.68E-31 | postive |
| SLC7A11 | AC005722.4 | 0.400121774 | 1.10E-20 | postive |
| AKR1C1 | AC005722.4 | 0.633089569 | 1.79E-57 | postive |
| AKR1C2 | AC005722.4 | 0.635433788 | 5.16E-58 | postive |
| AKR1C3 | AC005722.4 | 0.6151431 | 1.75E-53 | postive |
| GCLC | AC005722.4 | 0.562440004 | 3.92E-43 | postive |
| NQO1 | AC005722.4 | 0.518140386 | 9.16E-36 | postive |
| TXNRD1 | AC005722.4 | 0.548256401 | 1.18E-40 | postive |
| SRXN1 | AC005722.4 | 0.547324358 | 1.71E-40 | postive |
| GPX2 | AC005722.4 | 0.622428977 | 4.52E-55 | postive |
| FTL | AC005722.4 | 0.414579336 | 3.13E-22 | postive |
| MAFG | AC005722.4 | 0.538786975 | 4.61E-39 | postive |
| PRDX1 | AC005722.4 | 0.508790828 | 2.41E-34 | postive |
| G6PD | AC005722.4 | 0.569229963 | 2.31E-44 | postive |
| PGD | AC005722.4 | 0.626508512 | 5.58E-56 | postive |
| ABCC1 | AC005722.4 | 0.587969698 | 6.56E-48 | postive |
| IDH1 | AC005722.4 | 0.538573383 | 5.00E-39 | postive |
| ATF4 | AC010761.1 | 0.43398291 | 2.00E-24 | postive |
| TUBE1 | AC010761.1 | 0.409986664 | 9.88E-22 | postive |
| PHKG2 | AC010761.1 | 0.456538729 | 3.66E-27 | postive |
| TAZ | AC010761.1 | 0.459333749 | 1.62E-27 | postive |
| PHKG2 | ATXN2-AS | 0.459087989 | 1.74E-27 | postive |
| PEBP1 | ATXN2-AS | 0.446732013 | 6.00E-26 | postive |
| ATF4 | AL021707.8 | 0.460385314 | 1.19E-27 | postive |
| PHKG2 | AL021707.8 | 0.461798021 | 7.86E-28 | postive |
| EGLN2 | AL021707.8 | 0.404180033 | 4.12E-21 | postive |
| TAZ | AL021707.8 | 0.487931007 | 2.50E-31 | postive |
| PHKG2 | AL162274.2 | 0.530887873 | 8.96E-38 | postive |
| TAZ | AL162274.2 | 0.526695126 | 4.20E-37 | postive |
| HELLS | AC093227.1 | 0.40727676 | 1.93E-21 | postive |
| TUBE1 | AC093227.1 | 0.448569376 | 3.58E-26 | postive |
| GPX4 | CAMTA1-DT | 0.420037191 | 7.81E-23 | postive |
| STMN1 | CAMTA1-DT | 0.418968049 | 1.03E-22 | postive |
| PHKG2 | CAMTA1-DT | 0.600516808 | 2.04E-50 | postive |
| PEBP1 | CAMTA1-DT | 0.403955361 | 4.35E-21 | postive |
| ANO6 | CAMTA1-DT | -0.406568082 | 2.30E-21 | negative |
| EGLN2 | CAMTA1-DT | 0.446824081 | 5.85E-26 | postive |
| TAZ | CAMTA1-DT | 0.523892442 | 1.16E-36 | postive |
| TAZ | AC084125.4 | 0.406922937 | 2.11E-21 | postive |
| HELLS | EIF3J-DT | 0.495549958 | 2.09E-32 | postive |
| TUBE1 | EIF3J-DT | 0.4233753 | 3.30E-23 | postive |
| STMN1 | EIF3J-DT | 0.449839815 | 2.50E-26 | postive |
| GABPB1 | EIF3J-DT | 0.573644589 | 3.54E-45 | postive |
| NOX1 | EIF3J-DT | 0.405389501 | 3.07E-21 | postive |
| GLS2 | EIF3J-DT | 0.410419248 | 8.88E-22 | postive |
| GABARAPL2 | EIF3J-DT | 0.405077161 | 3.31E-21 | postive |
| PEBP1 | EIF3J-DT | 0.449362081 | 2.86E-26 | postive |
| TAZ | EIF3J-DT | 0.41354351 | 4.06E-22 | postive |
| PHKG2 | AC022167.2 | 0.41224315 | 5.63E-22 | postive |
| CISD1 | AC025171.5 | 0.414209643 | 3.44E-22 | postive |
| ISCU | AC025171.5 | 0.532591753 | 4.76E-38 | postive |
| ACSF2 | AC025171.5 | 0.400666787 | 9.64E-21 | postive |
| NOX1 | AC025171.5 | 0.400934807 | 9.04E-21 | postive |
| PHKG2 | AC004130.2 | 0.429673993 | 6.31E-24 | postive |
| WIPI2 | AC004130.2 | 0.440643276 | 3.26E-25 | postive |
| BID | AC004130.2 | 0.409010192 | 1.26E-21 | postive |
| TAZ | AC004130.2 | 0.477645292 | 6.47E-30 | postive |
| GPX4 | AL451165.2 | 0.472230692 | 3.43E-29 | postive |
| RPL8 | AL451165.2 | 0.42906725 | 7.41E-24 | postive |
| SLC2A8 | AL451165.2 | 0.431087768 | 4.33E-24 | postive |
| PHKG2 | AL451165.2 | 0.649646091 | 2.15E-61 | postive |
| GABARAPL2 | AL451165.2 | 0.400692444 | 9.58E-21 | postive |
| PEBP1 | AL451165.2 | 0.440458005 | 3.43E-25 | postive |
| ANO6 | AL451165.2 | -0.4300776 | 5.67E-24 | negative |
| EGLN2 | AL451165.2 | 0.54972954 | 6.62E-41 | postive |
| TAZ | AL451165.2 | 0.536992562 | 9.11E-39 | postive |
| DDIT4 | AC104695.4 | 0.427976138 | 9.89E-24 | postive |
| HILPDA | AC104695.4 | 0.409304007 | 1.17E-21 | postive |
| MYB | AC093620.1 | 0.521803493 | 2.47E-36 | postive |
| PLIN4 | C10orf71-AS1 | 0.809469459 | 1.80E-117 | postive |
| PRKAA2 | C10orf71-AS1 | 0.623831606 | 2.21E-55 | postive |
| ATF4 | ZKSCAN2-DT | 0.40980702 | 1.03E-21 | postive |
| HELLS | ZKSCAN2-DT | 0.444123748 | 1.24E-25 | postive |
| ISCU | ZKSCAN2-DT | 0.480084936 | 3.02E-30 | postive |
| ZNF419 | ZKSCAN2-DT | 0.427068616 | 1.26E-23 | postive |
| TUBE1 | ZKSCAN2-DT | 0.463886891 | 4.23E-28 | postive |
| STMN1 | ZKSCAN2-DT | 0.469698329 | 7.41E-29 | postive |
| NOX1 | ZKSCAN2-DT | 0.450515572 | 2.06E-26 | postive |
| PHKG2 | ZKSCAN2-DT | 0.610172129 | 2.01E-52 | postive |
| ATG4D | ZKSCAN2-DT | 0.424903301 | 2.21E-23 | postive |
| SOCS1 | ZKSCAN2-DT | 0.403756918 | 4.57E-21 | postive |
| MYB | ZKSCAN2-DT | 0.492670193 | 5.39E-32 | postive |
| TAZ | ZKSCAN2-DT | 0.539788636 | 3.15E-39 | postive |
| KLHL24 | USP27X-AS1 | 0.527137992 | 3.57E-37 | postive |
| ELAVL1 | USP27X-AS1 | 0.430747865 | 4.74E-24 | postive |
| SP1 | USP27X-AS1 | 0.40533975 | 3.11E-21 | postive |
| GLS2 | USP27X-AS1 | 0.434818155 | 1.59E-24 | postive |
| SNX4 | USP27X-AS1 | 0.436298014 | 1.07E-24 | postive |
| YY1AP1 | USP27X-AS1 | 0.406740751 | 2.20E-21 | postive |
| SIRT1 | USP27X-AS1 | 0.401008234 | 8.88E-21 | postive |
| NNMT | AL499627.1 | 0.433704429 | 2.15E-24 | postive |
| PLIN4 | AL499627.1 | 0.654916832 | 1.08E-62 | postive |
| ZEB1 | AL499627.1 | 0.417844005 | 1.37E-22 | postive |
| PRKAA2 | AL499627.1 | 0.642191098 | 1.34E-59 | postive |
| FANCD2 | OLMALINC | 0.438378111 | 6.07E-25 | postive |
| HELLS | OLMALINC | 0.567118791 | 5.61E-44 | postive |
| FADS2 | OLMALINC | 0.571439727 | 9.06E-45 | postive |
| KLHL24 | OLMALINC | 0.400288706 | 1.06E-20 | postive |
| TUBE1 | OLMALINC | 0.437531693 | 7.64E-25 | postive |
| HMGB1 | OLMALINC | 0.413463953 | 4.15E-22 | postive |
| ELAVL1 | OLMALINC | 0.500811111 | 3.64E-33 | postive |
| STMN1 | OLMALINC | 0.502896579 | 1.80E-33 | postive |
| GABPB1 | OLMALINC | 0.422055833 | 4.64E-23 | postive |
| GLS2 | OLMALINC | 0.535206411 | 1.79E-38 | postive |
| ATG4D | OLMALINC | 0.445833549 | 7.72E-26 | postive |
| PEBP1 | OLMALINC | 0.423853005 | 2.91E-23 | postive |
| YY1AP1 | OLMALINC | 0.408158266 | 1.55E-21 | postive |
| GPX4 | TNFRSF14-AS1 | 0.420645178 | 6.68E-23 | postive |
| CISD1 | TNFRSF14-AS1 | 0.434273213 | 1.85E-24 | postive |
| ISCU | TNFRSF14-AS1 | 0.631373564 | 4.43E-57 | postive |
| ACSF2 | TNFRSF14-AS1 | 0.417436297 | 1.52E-22 | postive |
| CARS1 | TNFRSF14-AS1 | 0.408862436 | 1.31E-21 | postive |
| ATG4D | TNFRSF14-AS1 | 0.405354498 | 3.09E-21 | postive |
| SOCS1 | TNFRSF14-AS1 | 0.410498412 | 8.70E-22 | postive |
| IFNG | TNFRSF14-AS1 | 0.41088507 | 7.90E-22 | postive |
| PHKG2 | DICER1-AS1 | 0.458307985 | 2.19E-27 | postive |
| TAZ | DICER1-AS1 | 0.477289402 | 7.23E-30 | postive |
| FANCD2 | AC127024.4 | 0.47408746 | 1.94E-29 | postive |
| HELLS | AC127024.4 | 0.551625417 | 3.12E-41 | postive |
| TUBE1 | AC127024.4 | 0.505398073 | 7.72E-34 | postive |
| SP1 | AC127024.4 | 0.406485988 | 2.35E-21 | postive |
| GABPB1 | AC127024.4 | 0.425580445 | 1.86E-23 | postive |
| GLS2 | AC127024.4 | 0.427162229 | 1.23E-23 | postive |
| SNX4 | AC127024.4 | 0.408021322 | 1.61E-21 | postive |
| ACVR1B | AC127024.4 | 0.457518208 | 2.75E-27 | postive |
| LPIN1 | AC127024.4 | 0.500172221 | 4.51E-33 | postive |
| SLC40A1 | AL136084.3 | 0.415649964 | 2.39E-22 | postive |
| ENPP2 | AL136084.3 | 0.516437281 | 1.67E-35 | postive |
| HIC1 | AL136084.3 | 0.412315224 | 5.53E-22 | postive |
| CYBB | AL136084.3 | 0.477593539 | 6.57E-30 | postive |
| ZEB1 | AL136084.3 | 0.620836096 | 1.01E-54 | postive |
| TLR4 | AL136084.3 | 0.595444154 | 2.17E-49 | postive |
| GPX4 | AC145423.2 | 0.431787065 | 3.60E-24 | postive |
| ATF4 | AC145423.2 | 0.422958485 | 3.67E-23 | postive |
| SLC2A8 | AC145423.2 | 0.472385731 | 3.27E-29 | postive |
| PHKG2 | AC145423.2 | 0.640956211 | 2.63E-59 | postive |
| PEBP1 | AC145423.2 | 0.465313161 | 2.77E-28 | postive |
| EGLN2 | AC145423.2 | 0.600004912 | 2.59E-50 | postive |
| TAZ | AC145423.2 | 0.519790349 | 5.09E-36 | postive |
| STMN1 | AC083798.2 | 0.417526306 | 1.48E-22 | postive |
| ATG3 | AC083798.2 | 0.404483588 | 3.83E-21 | postive |
| PML | AL157871.2 | 0.417755913 | 1.40E-22 | postive |
| IFNG | AL157871.2 | 0.41544651 | 2.52E-22 | postive |
| SLC40A1 | AC093278.2 | 0.547483888 | 1.60E-40 | postive |
| ENPP2 | AC093278.2 | 0.65521966 | 9.05E-63 | postive |
| HERPUD1 | AC093278.2 | 0.431275925 | 4.12E-24 | postive |
| ALOX5 | AC093278.2 | 0.541956323 | 1.37E-39 | postive |
| CYBB | AC093278.2 | 0.615949261 | 1.17E-53 | postive |
| FLT3 | AC093278.2 | 0.759588543 | 2.82E-95 | postive |
| ZEB1 | AC093278.2 | 0.477586679 | 6.59E-30 | postive |
| TLR4 | AC093278.2 | 0.528116537 | 2.49E-37 | postive |
| ATM | AC093278.2 | 0.400884736 | 9.15E-21 | postive |
| GPX4 | AC012510.1 | 0.438753154 | 5.47E-25 | postive |
| RPL8 | AC012510.1 | 0.458329831 | 2.17E-27 | postive |
| SLC2A8 | AC012510.1 | 0.463723691 | 4.44E-28 | postive |
| HRAS | AC012510.1 | 0.521060396 | 3.23E-36 | postive |
| PHKG2 | AC012510.1 | 0.640342824 | 3.68E-59 | postive |
| EGLN2 | AC012510.1 | 0.697568399 | 2.93E-74 | postive |
| TAZ | AC012510.1 | 0.433950459 | 2.01E-24 | postive |
| ZFP69B | AL512353.1 | 0.409232035 | 1.19E-21 | postive |
| TUBE1 | AC090772.3 | 0.405410608 | 3.05E-21 | postive |
| MYB | AC020907.4 | 0.518144059 | 9.15E-36 | postive |
| TP53 | BDNF-AS | 0.422278789 | 4.38E-23 | postive |
| HELLS | BDNF-AS | 0.407213206 | 1.96E-21 | postive |
| ISCU | BDNF-AS | 0.412727607 | 4.99E-22 | postive |
| ELAVL1 | BDNF-AS | 0.415037672 | 2.79E-22 | postive |
| GLS2 | BDNF-AS | 0.484603368 | 7.25E-31 | postive |
| ATG4D | BDNF-AS | 0.404975145 | 3.39E-21 | postive |
| MAPK8 | BDNF-AS | 0.427516839 | 1.12E-23 | postive |
| YY1AP1 | BDNF-AS | 0.445376361 | 8.78E-26 | postive |
| LPCAT3 | LINC02449 | 0.426532073 | 1.45E-23 | postive |
| HELLS | LINC01355 | 0.463055283 | 5.42E-28 | postive |
| TUBE1 | LINC01355 | 0.423001104 | 3.63E-23 | postive |
| STMN1 | LINC01355 | 0.437148159 | 8.48E-25 | postive |
| MYB | ZNF22-AS1 | 0.501044159 | 3.36E-33 | postive |
| HERPUD1 | AC012181.2 | 0.651862825 | 6.15E-62 | postive |
| SP1 | AC105206.2 | 0.432304204 | 3.13E-24 | postive |
| GLS2 | AC105206.2 | 0.408671702 | 1.37E-21 | postive |
| ZEB1 | AC105206.2 | 0.403292155 | 5.11E-21 | postive |
| ATM | AC105206.2 | 0.449639163 | 2.64E-26 | postive |
| ALOX12B | AC129492.1 | 0.687522572 | 2.35E-71 | postive |
| ALOXE3 | AC129492.1 | 0.63243665 | 2.53E-57 | postive |
| HELLS | AL356481.1 | 0.426567209 | 1.43E-23 | postive |
| ISCU | AL356481.1 | 0.439890641 | 4.01E-25 | postive |
| STMN1 | AL356481.1 | 0.428241641 | 9.22E-24 | postive |
| ISCU | AATBC | 0.465227828 | 2.84E-28 | postive |
| AGPAT3 | AATBC | 0.413167006 | 4.47E-22 | postive |
| GLS2 | AATBC | 0.474279339 | 1.83E-29 | postive |
| ATG4D | AATBC | 0.481221505 | 2.11E-30 | postive |
| HELLS | AC079907.1 | 0.433369464 | 2.35E-24 | postive |
| TUBE1 | AC079907.1 | 0.459532871 | 1.53E-27 | postive |
| TAZ | AC079907.1 | 0.422511942 | 4.12E-23 | postive |
| MT3 | SCGB1B2P | 0.583936543 | 3.98E-47 | postive |
| CDO1 | SCGB1B2P | 0.654928177 | 1.07E-62 | postive |
| GPX4 | AL359091.3 | 0.430272593 | 5.38E-24 | postive |
| PHKG2 | AL359091.3 | 0.547580399 | 1.54E-40 | postive |
| MAP1LC3A | AL359091.3 | 0.435287169 | 1.40E-24 | postive |
| SOCS1 | AL359091.3 | 0.410860929 | 7.95E-22 | postive |
| TAZ | AL359091.3 | 0.422878896 | 3.75E-23 | postive |
| CISD1 | AL022316.1 | 0.409771734 | 1.04E-21 | postive |
| ISCU | AL022316.1 | 0.426179748 | 1.59E-23 | postive |
| CARS1 | AL022316.1 | 0.404676946 | 3.65E-21 | postive |
| PHKG2 | Z69706.1 | 0.433585324 | 2.22E-24 | postive |
| MYB | Z69706.1 | 0.523870091 | 1.17E-36 | postive |
| MUC1 | FOXP4-AS1 | 0.499715788 | 5.25E-33 | postive |
| GPX4 | AL133520.1 | 0.452551182 | 1.15E-26 | postive |
| ISCU | AL133520.1 | 0.401381956 | 8.12E-21 | postive |
| STMN1 | AL133520.1 | 0.443634962 | 1.43E-25 | postive |
| NOX1 | AL133520.1 | 0.475281675 | 1.34E-29 | postive |
| PHKG2 | AL133520.1 | 0.440754464 | 3.16E-25 | postive |
| PEBP1 | AL133520.1 | 0.402735153 | 5.85E-21 | postive |
| KRAS | AP001029.1 | 0.450350492 | 2.16E-26 | postive |
| TSC22D3 | LINC01480 | 0.503912123 | 1.28E-33 | postive |
| HERPUD1 | LINC01480 | 0.422495006 | 4.14E-23 | postive |
| FLT3 | LINC01480 | 0.440918809 | 3.02E-25 | postive |
| GPX4 | AL035071.1 | 0.479862516 | 3.24E-30 | postive |
| MAP1LC3A | AL035071.1 | 0.481917809 | 1.70E-30 | postive |
| SOCS1 | AL035071.1 | 0.45152707 | 1.55E-26 | postive |
| HELLS | AC091185.1 | 0.444877086 | 1.01E-25 | postive |
| SP1 | AC091185.1 | 0.410447146 | 8.81E-22 | postive |
| MAPK8 | AC091185.1 | 0.402020578 | 6.96E-21 | postive |
| GPX4 | ARRDC1-AS1 | 0.433810351 | 2.09E-24 | postive |
| CHMP6 | ARRDC1-AS1 | 0.427013164 | 1.27E-23 | postive |
| SLC2A8 | ARRDC1-AS1 | 0.510382681 | 1.39E-34 | postive |
| STMN1 | ARRDC1-AS1 | 0.452264658 | 1.25E-26 | postive |
| NOX1 | ARRDC1-AS1 | 0.404750409 | 3.59E-21 | postive |
| PHKG2 | ARRDC1-AS1 | 0.61499332 | 1.89E-53 | postive |
| EGLN2 | ARRDC1-AS1 | 0.458044426 | 2.36E-27 | postive |
| TAZ | ARRDC1-AS1 | 0.474473142 | 1.73E-29 | postive |
| CAV1 | TENM3-AS1 | 0.4092885 | 1.18E-21 | postive |
| NFE2L2 | SOX21-AS1 | 0.523945642 | 1.14E-36 | postive |
| BRD4 | SOX21-AS1 | 0.458033906 | 2.37E-27 | postive |
| KLHL24 | SOX21-AS1 | 0.483978442 | 8.85E-31 | postive |
| SETD1B | SOX21-AS1 | 0.442031889 | 2.22E-25 | postive |
| IREB2 | SOX21-AS1 | 0.412882134 | 4.80E-22 | postive |
| ELAVL1 | SOX21-AS1 | 0.479895035 | 3.21E-30 | postive |
| SP1 | SOX21-AS1 | 0.500062355 | 4.68E-33 | postive |
| GLS2 | SOX21-AS1 | 0.447999366 | 4.20E-26 | postive |
| YY1AP1 | SOX21-AS1 | 0.466782183 | 1.78E-28 | postive |
| GPX4 | DDX11-AS1 | 0.529553234 | 1.47E-37 | postive |
| CISD1 | DDX11-AS1 | 0.430433139 | 5.16E-24 | postive |
| FANCD2 | DDX11-AS1 | 0.498437361 | 8.04E-33 | postive |
| HELLS | DDX11-AS1 | 0.526665063 | 4.24E-37 | postive |
| FADS2 | DDX11-AS1 | 0.459219124 | 1.68E-27 | postive |
| ISCU | DDX11-AS1 | 0.489620938 | 1.45E-31 | postive |
| HMGB1 | DDX11-AS1 | 0.431425958 | 3.96E-24 | postive |
| ELAVL1 | DDX11-AS1 | 0.420022116 | 7.84E-23 | postive |
| STMN1 | DDX11-AS1 | 0.554355843 | 1.05E-41 | postive |
| NOX1 | DDX11-AS1 | 0.418151347 | 1.27E-22 | postive |
| GLS2 | DDX11-AS1 | 0.452624324 | 1.13E-26 | postive |
| ATG4D | DDX11-AS1 | 0.410775516 | 8.12E-22 | postive |
| MYB | DDX11-AS1 | 0.418529629 | 1.15E-22 | postive |
| TAZ | DDX11-AS1 | 0.434112155 | 1.93E-24 | postive |
| HIC1 | AC147067.2 | 0.438810628 | 5.39E-25 | postive |
| NFE2L2 | APTR | 0.412523578 | 5.25E-22 | postive |
| ATF4 | APTR | 0.450604807 | 2.01E-26 | postive |
| HELLS | APTR | 0.409031764 | 1.25E-21 | postive |
| ISCU | APTR | 0.490407739 | 1.12E-31 | postive |
| ASNS | APTR | 0.4513966 | 1.60E-26 | postive |
| PSAT1 | APTR | 0.440480053 | 3.41E-25 | postive |
| TUBE1 | APTR | 0.518644495 | 7.66E-36 | postive |
| ELAVL1 | APTR | 0.46472569 | 3.30E-28 | postive |
| STMN1 | APTR | 0.426790964 | 1.35E-23 | postive |
| GABPB1 | APTR | 0.477466312 | 6.84E-30 | postive |
| NOX1 | APTR | 0.44708927 | 5.43E-26 | postive |
| TFR2 | APTR | 0.445530173 | 8.41E-26 | postive |
| GLS2 | APTR | 0.428597715 | 8.39E-24 | postive |
| KEAP1 | APTR | 0.457401667 | 2.85E-27 | postive |
| PHKG2 | APTR | 0.419904225 | 8.08E-23 | postive |
| ATG4D | APTR | 0.513529879 | 4.66E-35 | postive |
| BID | APTR | 0.487275143 | 3.09E-31 | postive |
| YY1AP1 | APTR | 0.458898123 | 1.84E-27 | postive |
| PANX1 | APTR | -0.41442842 | 3.25E-22 | negative |
| ALOX12B | LINC02621 | 0.409119765 | 1.23E-21 | postive |
| ENPP2 | AL731567.1 | 0.412754246 | 4.95E-22 | postive |
| ALOX5 | AL731567.1 | 0.681943294 | 8.57E-70 | postive |
| CYBB | AL731567.1 | 0.401172905 | 8.54E-21 | postive |
| FLT3 | AL731567.1 | 0.423988744 | 2.81E-23 | postive |
| PHKG2 | AC020910.5 | 0.413595734 | 4.01E-22 | postive |
| GLS2 | AP002026.1 | 0.479737056 | 3.37E-30 | postive |
| TAZ | Z95115.1 | 0.411243602 | 7.23E-22 | postive |
| ZNF419 | AC011477.1 | 0.424043076 | 2.77E-23 | postive |
| FH | AC008946.1 | 0.41001034 | 9.82E-22 | postive |
| SLC2A8 | AC008946.1 | 0.468784087 | 9.77E-29 | postive |
| PHKG2 | AC008946.1 | 0.533739113 | 3.10E-38 | postive |
| PEBP1 | AC008946.1 | 0.450458221 | 2.10E-26 | postive |
| EGLN2 | AC008946.1 | 0.408480517 | 1.44E-21 | postive |
| TAZ | AC008946.1 | 0.441758209 | 2.40E-25 | postive |
| LONP1 | AC008946.1 | 0.412768565 | 4.94E-22 | postive |
| ALOX12B | AC007182.1 | 0.545458078 | 3.53E-40 | postive |
| ALOXE3 | AC007182.1 | 0.530978168 | 8.67E-38 | postive |
| GPX2 | AL589986.2 | 0.413082067 | 4.56E-22 | postive |
| NFE2L2 | AC079807.1 | 0.480554675 | 2.61E-30 | postive |
| BRD4 | AC079807.1 | 0.491906094 | 6.91E-32 | postive |
| SETD1B | AC079807.1 | 0.453997964 | 7.62E-27 | postive |
| IREB2 | AC079807.1 | 0.461393894 | 8.85E-28 | postive |
| SP1 | AC079807.1 | 0.401278042 | 8.32E-21 | postive |
| HELLS | LINC01475 | 0.493283487 | 4.41E-32 | postive |
| ISCU | LINC01475 | 0.46804654 | 1.22E-28 | postive |
| GLS2 | LINC01475 | 0.520896519 | 3.42E-36 | postive |
| MAPK8 | LINC01475 | 0.438302971 | 6.19E-25 | postive |
| PHKG2 | TBX2-AS1 | 0.42840651 | 8.83E-24 | postive |
| MAP1LC3A | TBX2-AS1 | 0.431386229 | 4.00E-24 | postive |
| ATG3 | ZBTB11-AS1 | 0.516015659 | 1.94E-35 | postive |
| SNX4 | ZBTB11-AS1 | 0.462317197 | 6.74E-28 | postive |
| GPX2 | EDIL3-DT | 0.425432252 | 1.93E-23 | postive |
| HIC1 | AP001434.1 | 0.44886138 | 3.30E-26 | postive |
| NOX4 | AP001434.1 | 0.443175061 | 1.62E-25 | postive |
| ZEB1 | AP001434.1 | 0.427342387 | 1.17E-23 | postive |
| FANCD2 | AC104532.2 | 0.44021516 | 3.67E-25 | postive |
| HELLS | AC104532.2 | 0.487120883 | 3.25E-31 | postive |
| TUBE1 | AC104532.2 | 0.452771403 | 1.08E-26 | postive |
| MYB | AC104532.2 | 0.479062533 | 4.16E-30 | postive |
| LPIN1 | AC104532.2 | 0.416472409 | 1.94E-22 | postive |
| YY1AP1 | AC104532.2 | 0.457237865 | 2.99E-27 | postive |
| TP53 | AC017100.1 | 0.407180224 | 1.98E-21 | postive |
| HELLS | AC017100.1 | 0.44757673 | 4.74E-26 | postive |
| FADS2 | AC017100.1 | 0.403769989 | 4.55E-21 | postive |
| ISCU | AC017100.1 | 0.403439865 | 4.93E-21 | postive |
| GLS2 | AC017100.1 | 0.487316886 | 3.05E-31 | postive |
| MAP1LC3A | AC017100.1 | 0.420675443 | 6.63E-23 | postive |
| MAPK8 | AC017100.1 | 0.42108282 | 5.97E-23 | postive |
| GPX4 | AL358075.2 | 0.410003992 | 9.84E-22 | postive |
| PHKG2 | AL358075.2 | 0.431559739 | 3.82E-24 | postive |
| NCF2 | SMIM25 | 0.407712684 | 1.73E-21 | postive |
| ALOX5 | SMIM25 | 0.657829625 | 2.00E-63 | postive |
| CYBB | SMIM25 | 0.638883906 | 8.10E-59 | postive |
| TLR4 | SMIM25 | 0.462218662 | 6.94E-28 | postive |
| HIC1 | MIR100HG | 0.460723083 | 1.08E-27 | postive |
| NOX4 | MIR100HG | 0.456737484 | 3.45E-27 | postive |
| ZEB1 | MIR100HG | 0.450089721 | 2.33E-26 | postive |
| GPX4 | EMC1-AS1 | 0.449660595 | 2.63E-26 | postive |
| STMN1 | EMC1-AS1 | 0.42267916 | 3.95E-23 | postive |
| NFE2L2 | PAXIP1-AS1 | 0.493861882 | 3.65E-32 | postive |
| BRD4 | PAXIP1-AS1 | 0.415256174 | 2.64E-22 | postive |
| GPX2 | PAXIP1-AS1 | 0.462312538 | 6.75E-28 | postive |
| PSAT1 | PAXIP1-AS1 | 0.43267081 | 2.84E-24 | postive |
| KLHL24 | PAXIP1-AS1 | 0.406602718 | 2.28E-21 | postive |
| TUBE1 | PAXIP1-AS1 | 0.428268764 | 9.15E-24 | postive |
| ELAVL1 | PAXIP1-AS1 | 0.458215951 | 2.25E-27 | postive |
| GABPB1 | PAXIP1-AS1 | 0.44567557 | 8.07E-26 | postive |
| KEAP1 | PAXIP1-AS1 | 0.408427507 | 1.45E-21 | postive |
| ATG4D | PAXIP1-AS1 | 0.466043346 | 2.23E-28 | postive |
| GABARAPL1 | PAXIP1-AS1 | 0.446222128 | 6.93E-26 | postive |
| YY1AP1 | PAXIP1-AS1 | 0.408485693 | 1.43E-21 | postive |
| TAZ | PAXIP1-AS1 | 0.400980948 | 8.94E-21 | postive |
| ENPP2 | AC243960.1 | 0.516017293 | 1.94E-35 | postive |
| ISCU | AC243960.1 | 0.405882393 | 2.72E-21 | postive |
| HERPUD1 | AC243960.1 | 0.448698957 | 3.45E-26 | postive |
| ALOX5 | AC243960.1 | 0.456976581 | 3.22E-27 | postive |
| FLT3 | AC243960.1 | 0.688975597 | 9.07E-72 | postive |
| IFNG | AC243960.1 | 0.453112567 | 9.83E-27 | postive |
| CAV1 | ITGB1-DT | 0.40355301 | 4.80E-21 | postive |
| HELLS | LINC01560 | 0.465177605 | 2.88E-28 | postive |
| KLHL24 | LINC01560 | 0.541671427 | 1.53E-39 | postive |
| TUBE1 | LINC01560 | 0.423093319 | 3.55E-23 | postive |
| ELAVL1 | LINC01560 | 0.447210674 | 5.25E-26 | postive |
| GABPB1 | LINC01560 | 0.493514477 | 4.09E-32 | postive |
| GLS2 | LINC01560 | 0.418489482 | 1.16E-22 | postive |
| SNX4 | LINC01560 | 0.408229577 | 1.53E-21 | postive |
| ATF4 | AC103691.1 | 0.407652677 | 1.76E-21 | postive |
| CHMP6 | AC103691.1 | 0.435249115 | 1.42E-24 | postive |
| HRAS | AC103691.1 | 0.501124293 | 3.28E-33 | postive |
| PHKG2 | AC103691.1 | 0.628728252 | 1.77E-56 | postive |
| ANO6 | AC103691.1 | -0.411684698 | 6.48E-22 | negative |
| EGLN2 | AC103691.1 | 0.612271858 | 7.21E-53 | postive |
| TAZ | AC103691.1 | 0.518672094 | 7.58E-36 | postive |
| BID | LINC01770 | 0.488527784 | 2.07E-31 | postive |
| MIOX | LINC01770 | 0.493334149 | 4.34E-32 | postive |
| SP1 | AC022150.4 | 0.439187834 | 4.86E-25 | postive |
| GPX4 | MYLK-AS1 | 0.436748917 | 9.45E-25 | postive |
| PHKG2 | MYLK-AS1 | 0.484874865 | 6.65E-31 | postive |
| HELLS | AL022322.1 | 0.437274271 | 8.19E-25 | postive |
| TUBE1 | AL022322.1 | 0.451127811 | 1.73E-26 | postive |
| STMN1 | AL022322.1 | 0.413612323 | 3.99E-22 | postive |
| PHKG2 | AL022322.1 | 0.435397556 | 1.36E-24 | postive |
| MYB | AL022322.1 | 0.438121358 | 6.51E-25 | postive |
| ATG4D | AC112484.3 | 0.417195099 | 1.61E-22 | postive |
| AKR1C1 | AC004990.1 | 0.424699803 | 2.34E-23 | postive |
| AKR1C2 | AC004990.1 | 0.420937268 | 6.19E-23 | postive |
| GCLC | AC004990.1 | 0.409440123 | 1.13E-21 | postive |
| NFE2L2 | AC004990.1 | 0.510210381 | 1.48E-34 | postive |
| BRD4 | AC004990.1 | 0.400255383 | 1.06E-20 | postive |
| TXNRD1 | AC004990.1 | 0.419855397 | 8.18E-23 | postive |
| GPX2 | AC004990.1 | 0.496396882 | 1.58E-32 | postive |
| MAFG | AC004990.1 | 0.444962364 | 9.85E-26 | postive |
| PGD | AC004990.1 | 0.441810838 | 2.36E-25 | postive |
| ABCC1 | AC004990.1 | 0.439570214 | 4.38E-25 | postive |
| IDH1 | AC004990.1 | 0.420405408 | 7.10E-23 | postive |
| SLC40A1 | TRBV11-2 | 0.424358529 | 2.55E-23 | postive |
| ENPP2 | TRBV11-2 | 0.480182281 | 2.93E-30 | postive |
| CYBB | TRBV11-2 | 0.524291632 | 1.01E-36 | postive |
| FLT3 | TRBV11-2 | 0.618714426 | 2.95E-54 | postive |
| IFNG | TRBV11-2 | 0.495340528 | 2.24E-32 | postive |
| ISCU | IL10RB-DT | 0.421169427 | 5.83E-23 | postive |
| BID | IL10RB-DT | 0.406339509 | 2.43E-21 | postive |
| NGB | MAFA-AS1 | 0.449475461 | 2.77E-26 | postive |
| TAZ | CNNM3-DT | 0.433488536 | 2.28E-24 | postive |
| GLS2 | AL355803.1 | 0.424859551 | 2.24E-23 | postive |
| GPX4 | AC022007.1 | 0.502790697 | 1.87E-33 | postive |
| CISD1 | AC022007.1 | 0.419289813 | 9.46E-23 | postive |
| FANCD2 | AC022007.1 | 0.528475254 | 2.18E-37 | postive |
| ATF4 | AC022007.1 | 0.413843859 | 3.77E-22 | postive |
| FH | AC022007.1 | 0.402895004 | 5.63E-21 | postive |
| ISCU | AC022007.1 | 0.435803935 | 1.22E-24 | postive |
| STMN1 | AC022007.1 | 0.620456574 | 1.23E-54 | postive |
| NOX1 | AC022007.1 | 0.563580129 | 2.45E-43 | postive |
| PHKG2 | AC022007.1 | 0.507497407 | 3.76E-34 | postive |
| PEBP1 | AC022007.1 | 0.41578401 | 2.31E-22 | postive |
| EGLN2 | AC022007.1 | 0.435968189 | 1.17E-24 | postive |
| TAZ | AC022007.1 | 0.480166153 | 2.95E-30 | postive |
| KLHL24 | AC009271.1 | 0.429504817 | 6.60E-24 | postive |
| HELLS | TMEM9B-AS1 | 0.44387278 | 1.33E-25 | postive |
| GLS2 | TMEM9B-AS1 | 0.435063801 | 1.49E-24 | postive |
| MYB | TMEM9B-AS1 | 0.485277941 | 5.85E-31 | postive |
| ATM | TMEM9B-AS1 | 0.410381664 | 8.96E-22 | postive |
| HIC1 | AC134312.5 | 0.617200416 | 6.29E-54 | postive |
| NOX4 | AC134312.5 | 0.675523237 | 4.87E-68 | postive |
| ZEB1 | AC134312.5 | 0.488969072 | 1.79E-31 | postive |
| PHKG2 | TOLLIP-AS1 | 0.490063223 | 1.26E-31 | postive |
| TAZ | TOLLIP-AS1 | 0.456450174 | 3.75E-27 | postive |
| TP53 | FAM111A-DT | 0.406045951 | 2.61E-21 | postive |
| HELLS | FAM111A-DT | 0.481445206 | 1.97E-30 | postive |
| ISCU | FAM111A-DT | 0.471710764 | 4.02E-29 | postive |
| AGPAT3 | FAM111A-DT | 0.409778454 | 1.04E-21 | postive |
| SP1 | FAM111A-DT | 0.414262233 | 3.39E-22 | postive |
| STMN1 | FAM111A-DT | 0.44756271 | 4.75E-26 | postive |
| GLS2 | FAM111A-DT | 0.4135338 | 4.07E-22 | postive |
| MYB | FAM111A-DT | 0.418894292 | 1.05E-22 | postive |
| MAPK8 | FAM111A-DT | 0.452546636 | 1.16E-26 | postive |
| ATM | FAM111A-DT | 0.408780757 | 1.33E-21 | postive |
| YY1AP1 | FAM111A-DT | 0.403644426 | 4.69E-21 | postive |
| CHMP6 | AL022328.4 | 0.407849993 | 1.68E-21 | postive |
| PHKG2 | AL022328.4 | 0.520290084 | 4.26E-36 | postive |
| GPX4 | AC108134.4 | 0.414892473 | 2.89E-22 | postive |
| MUC1 | AC108134.4 | 0.503879438 | 1.29E-33 | postive |
| PHKG2 | AC108134.4 | 0.544432056 | 5.27E-40 | postive |
| BRD4 | AC135050.5 | 0.447005388 | 5.56E-26 | postive |
| TUBE1 | AC135050.5 | 0.456070749 | 4.19E-27 | postive |
| SETD1B | AC135050.5 | 0.457893375 | 2.47E-27 | postive |
| IREB2 | AC135050.5 | 0.424416652 | 2.51E-23 | postive |
| SP1 | AC135050.5 | 0.466705429 | 1.83E-28 | postive |
| ACVR1B | AC135050.5 | 0.470846543 | 5.23E-29 | postive |
| LPIN1 | AC135050.5 | 0.404394077 | 3.91E-21 | postive |
| ATM | AC135050.5 | 0.431405252 | 3.98E-24 | postive |
| NFE2L2 | AL354993.2 | 0.467201967 | 1.57E-28 | postive |
| KLHL24 | AL354993.2 | 0.602625093 | 7.54E-51 | postive |
| PIK3CA | AL354993.2 | 0.462635632 | 6.14E-28 | postive |
| LPCAT3 | AL354993.2 | 0.402930464 | 5.58E-21 | postive |
| GLS2 | AL354993.2 | 0.403561071 | 4.79E-21 | postive |
| SIRT1 | AL354993.2 | 0.418620641 | 1.12E-22 | postive |
| HELLS | RBM26-AS1 | 0.453148436 | 9.73E-27 | postive |
| ISCU | AC121761.2 | 0.405772806 | 2.79E-21 | postive |
| FANCD2 | AC116366.2 | 0.422077322 | 4.62E-23 | postive |
| TP53 | AC116366.2 | 0.436284972 | 1.07E-24 | postive |
| HELLS | AC116366.2 | 0.497831939 | 9.84E-33 | postive |
| ENPP2 | AC116366.2 | 0.42225038 | 4.41E-23 | postive |
| ISCU | AC116366.2 | 0.424398065 | 2.53E-23 | postive |
| GCH1 | AC116366.2 | 0.489272697 | 1.62E-31 | postive |
| AGPAT3 | AC116366.2 | 0.418525261 | 1.15E-22 | postive |
| SP1 | AC116366.2 | 0.479784953 | 3.32E-30 | postive |
| FLT3 | AC116366.2 | 0.496204755 | 1.69E-32 | postive |
| MAPK8 | AC116366.2 | 0.448241792 | 3.93E-26 | postive |
| IFNG | AC116366.2 | 0.43444374 | 1.76E-24 | postive |
| STAT3 | AC108449.2 | 0.47701097 | 7.88E-30 | postive |
| MTOR | AC108449.2 | 0.485744948 | 5.04E-31 | postive |
| BRD4 | AC108449.2 | 0.433000137 | 2.60E-24 | postive |
| KLHL24 | AC108449.2 | 0.434955314 | 1.54E-24 | postive |
| ARRDC3 | AC108449.2 | 0.416723924 | 1.82E-22 | postive |
| SETD1B | AC108449.2 | 0.571870608 | 7.54E-45 | postive |
| IREB2 | AC108449.2 | 0.535427611 | 1.64E-38 | postive |
| SP1 | AC108449.2 | 0.703736134 | 4.20E-76 | postive |
| CS | AC108449.2 | 0.408152069 | 1.56E-21 | postive |
| PIK3CA | AC108449.2 | 0.438050956 | 6.63E-25 | postive |
| LPCAT3 | AC108449.2 | 0.407234157 | 1.95E-21 | postive |
| GLS2 | AC108449.2 | 0.416441292 | 1.96E-22 | postive |
| NCOA4 | AC108449.2 | 0.458225702 | 2.24E-27 | postive |
| MAPK8 | AC108449.2 | 0.412870661 | 4.81E-22 | postive |
| ACVR1B | AC108449.2 | 0.504022155 | 1.23E-33 | postive |
| ATM | AC108449.2 | 0.596687321 | 1.22E-49 | postive |
| SIRT1 | AC108449.2 | 0.54322237 | 8.41E-40 | postive |
| PHKG2 | BRWD1-AS2 | 0.501121737 | 3.28E-33 | postive |
| WIPI2 | BRWD1-AS2 | 0.420970028 | 6.14E-23 | postive |
| FANCD2 | MIR600HG | 0.460948891 | 1.01E-27 | postive |
| HELLS | MIR600HG | 0.539150995 | 4.01E-39 | postive |
| FADS2 | MIR600HG | 0.447006893 | 5.56E-26 | postive |
| BRD4 | MIR600HG | 0.404789648 | 3.55E-21 | postive |
| KLHL24 | MIR600HG | 0.407337935 | 1.90E-21 | postive |
| TUBE1 | MIR600HG | 0.411155801 | 7.39E-22 | postive |
| ELAVL1 | MIR600HG | 0.4859515 | 4.72E-31 | postive |
| SP1 | MIR600HG | 0.490811335 | 9.87E-32 | postive |
| STMN1 | MIR600HG | 0.429403612 | 6.78E-24 | postive |
| GLS2 | MIR600HG | 0.544773912 | 4.61E-40 | postive |
| KEAP1 | MIR600HG | 0.456921473 | 3.27E-27 | postive |
| ATG4D | MIR600HG | 0.495495261 | 2.13E-32 | postive |
| SNX4 | MIR600HG | 0.440237054 | 3.65E-25 | postive |
| LPIN1 | MIR600HG | 0.404909253 | 3.45E-21 | postive |
| YY1AP1 | MIR600HG | 0.48665722 | 3.77E-31 | postive |
| GPX2 | AC009403.1 | 0.472809181 | 2.87E-29 | postive |
| TAZ | AC009403.1 | 0.446027053 | 7.32E-26 | postive |
| NFE2L2 | KIAA1671-AS1 | 0.400068136 | 1.11E-20 | postive |
| PLIN4 | KIAA1671-AS1 | 0.544086795 | 6.02E-40 | postive |
| WIPI1 | KIAA1671-AS1 | 0.430754553 | 4.74E-24 | postive |
| PRKAA2 | KIAA1671-AS1 | 0.544452798 | 5.22E-40 | postive |
| GPX4 | AC098484.2 | 0.517149414 | 1.30E-35 | postive |
| ATF4 | AC098484.2 | 0.425159242 | 2.07E-23 | postive |
| ISCU | AC098484.2 | 0.49467768 | 2.79E-32 | postive |
| SLC2A8 | AC098484.2 | 0.447055741 | 5.48E-26 | postive |
| STMN1 | AC098484.2 | 0.53531737 | 1.71E-38 | postive |
| NOX1 | AC098484.2 | 0.511492725 | 9.47E-35 | postive |
| PHKG2 | AC098484.2 | 0.547542263 | 1.57E-40 | postive |
| ATG4D | AC098484.2 | 0.408985266 | 1.27E-21 | postive |
| PEBP1 | AC098484.2 | 0.400603354 | 9.79E-21 | postive |
| MYB | AC098484.2 | 0.409843087 | 1.02E-21 | postive |
| EGLN2 | AC098484.2 | 0.489090598 | 1.72E-31 | postive |
| TAZ | AC098484.2 | 0.45082414 | 1.89E-26 | postive |
| HELLS | AL158212.3 | 0.484779891 | 6.86E-31 | postive |
| SP1 | AL158212.3 | 0.47787008 | 6.03E-30 | postive |
| MAPK8 | AL158212.3 | 0.489094816 | 1.72E-31 | postive |
| PHKG2 | AC010359.2 | 0.486187403 | 4.38E-31 | postive |
| TAZ | AC010359.2 | 0.425424174 | 1.93E-23 | postive |
| PHKG2 | AL117379.1 | 0.578885148 | 3.67E-46 | postive |
| EGLN2 | AL117379.1 | 0.446057975 | 7.25E-26 | postive |
| TAZ | AL117379.1 | 0.53079042 | 9.29E-38 | postive |
| ISCU | MIR155HG | 0.401124752 | 8.64E-21 | postive |
| FLT3 | MIR155HG | 0.446536647 | 6.34E-26 | postive |
| IFNG | MIR155HG | 0.625643161 | 8.72E-56 | postive |
| NNMT | LANCL1-AS1 | 0.478942188 | 4.32E-30 | postive |
| PLIN4 | LANCL1-AS1 | 0.720143252 | 3.01E-81 | postive |
| WIPI1 | LANCL1-AS1 | 0.425852347 | 1.73E-23 | postive |
| ZEB1 | LANCL1-AS1 | 0.463664236 | 4.52E-28 | postive |
| PRKAA2 | LANCL1-AS1 | 0.766235252 | 6.56E-98 | postive |
| NFE2L2 | ODC1-DT | 0.431885294 | 3.50E-24 | postive |
| FADS2 | ODC1-DT | 0.513078213 | 5.45E-35 | postive |
| TXNRD1 | ODC1-DT | 0.420921967 | 6.22E-23 | postive |
| ELAVL1 | ODC1-DT | 0.450754779 | 1.93E-26 | postive |
| GLS2 | ODC1-DT | 0.417933158 | 1.34E-22 | postive |
| ABCC1 | ODC1-DT | 0.426358088 | 1.51E-23 | postive |
| LPIN1 | ODC1-DT | 0.435392199 | 1.37E-24 | postive |
| HELLS | AC137932.1 | 0.420263381 | 7.37E-23 | postive |
| MAPK8 | AC137932.1 | 0.428727297 | 8.11E-24 | postive |
| HELLS | AC079610.1 | 0.433382878 | 2.35E-24 | postive |
| STMN1 | AC079610.1 | 0.416291694 | 2.03E-22 | postive |
| SLC2A3 | TGFB2-AS1 | 0.515374947 | 2.44E-35 | postive |
| NOX4 | TGFB2-AS1 | 0.478841587 | 4.46E-30 | postive |
| MYB | AC016735.1 | 0.412814897 | 4.88E-22 | postive |
| GPX4 | AL031716.1 | 0.458533226 | 2.05E-27 | postive |
| HELLS | AL031716.1 | 0.431951414 | 3.44E-24 | postive |
| ISCU | AL031716.1 | 0.498603361 | 7.61E-33 | postive |
| STMN1 | AL031716.1 | 0.431044004 | 4.39E-24 | postive |
| NOX1 | AL031716.1 | 0.452482955 | 1.18E-26 | postive |
| PHKG2 | AL031716.1 | 0.464319956 | 3.72E-28 | postive |
| MAP1LC3A | AL031716.1 | 0.456837056 | 3.36E-27 | postive |
| SOCS1 | AL031716.1 | 0.503097643 | 1.68E-33 | postive |
| MYB | AL031716.1 | 0.521821592 | 2.46E-36 | postive |
| ATF4 | AL136295.2 | 0.422793203 | 3.83E-23 | postive |
| ISCU | AL136295.2 | 0.436264244 | 1.08E-24 | postive |
| NOX1 | AL136295.2 | 0.414820501 | 2.95E-22 | postive |
| PHKG2 | AL136295.2 | 0.502578509 | 2.01E-33 | postive |
| EGLN2 | AL136295.2 | 0.407973378 | 1.63E-21 | postive |
| TAZ | AL136295.2 | 0.446260038 | 6.85E-26 | postive |
| AKR1C1 | AC089983.1 | 0.456369144 | 3.84E-27 | postive |
| AKR1C3 | AC089983.1 | 0.506874329 | 4.66E-34 | postive |
| TXNRD1 | AC089983.1 | 0.520305029 | 4.23E-36 | postive |
| SRXN1 | AC089983.1 | 0.403941425 | 4.37E-21 | postive |
| GPX2 | AC089983.1 | 0.406050524 | 2.61E-21 | postive |
| GPX4 | AL844908.2 | 0.409702835 | 1.06E-21 | postive |
| PHKG2 | AL844908.2 | 0.520271068 | 4.28E-36 | postive |
| TUBE1 | AL928654.2 | 0.410443734 | 8.82E-22 | postive |
| PEBP1 | AC018645.3 | 0.414889678 | 2.90E-22 | postive |
| TAZ | AF196972.1 | 0.414442786 | 3.24E-22 | postive |
| KLHL24 | YEATS2-AS1 | 0.466129415 | 2.17E-28 | postive |
| TUBE1 | YEATS2-AS1 | 0.427139755 | 1.23E-23 | postive |
| PIK3CA | YEATS2-AS1 | 0.430766417 | 4.72E-24 | postive |
| SNX4 | YEATS2-AS1 | 0.408468328 | 1.44E-21 | postive |
| ACVR1B | YEATS2-AS1 | 0.401860909 | 7.23E-21 | postive |
| PML | DANCR | -0.416004521 | 2.18E-22 | negative |
| ASNS | DANCR | 0.440977645 | 2.97E-25 | postive |
| ELAVL1 | DANCR | 0.500949264 | 3.47E-33 | postive |
| STMN1 | DANCR | 0.453206017 | 9.57E-27 | postive |
| PRDX1 | DANCR | 0.420344523 | 7.22E-23 | postive |
| KEAP1 | DANCR | 0.40262672 | 6.01E-21 | postive |
| ATG4D | DANCR | 0.420986606 | 6.12E-23 | postive |
| BID | DANCR | 0.422236929 | 4.43E-23 | postive |
| PEBP1 | DANCR | 0.52555945 | 6.35E-37 | postive |
| ATG13 | AC018716.1 | 0.400522424 | 9.98E-21 | postive |
| GPX4 | AC011462.4 | 0.415668352 | 2.38E-22 | postive |
| STMN1 | AC011462.4 | 0.401924773 | 7.12E-21 | postive |
| PHKG2 | AC011462.4 | 0.569063951 | 2.48E-44 | postive |
| MAP1LC3A | AC011462.4 | 0.427927006 | 1.00E-23 | postive |
| EGLN2 | AC011462.4 | 0.453937868 | 7.75E-27 | postive |
| TAZ | AC011462.4 | 0.433472629 | 2.29E-24 | postive |
| STMN1 | AL359715.3 | 0.446268045 | 6.84E-26 | postive |
| GPX4 | AC010542.6 | 0.415131342 | 2.72E-22 | postive |
| ISCU | AC010542.6 | 0.40748158 | 1.84E-21 | postive |
| SLC2A8 | AC010542.6 | 0.402468212 | 6.24E-21 | postive |
| STMN1 | AC010542.6 | 0.520822991 | 3.52E-36 | postive |
| NOX1 | AC010542.6 | 0.465522873 | 2.60E-28 | postive |
| PHKG2 | AC010542.6 | 0.61395638 | 3.15E-53 | postive |
| EGLN2 | AC010542.6 | 0.447333327 | 5.07E-26 | postive |
| TAZ | AC010542.6 | 0.587974309 | 6.54E-48 | postive |
| CHAC1 | LINC02560 | 0.445723837 | 7.96E-26 | postive |
| DUOX1 | LINC02560 | 0.434589364 | 1.70E-24 | postive |
| ALOX12B | LINC02560 | 0.675987472 | 3.65E-68 | postive |
| ALOXE3 | LINC02560 | 0.522948131 | 1.64E-36 | postive |
| MAPK3 | LINC02560 | 0.505603614 | 7.19E-34 | postive |
| MIOX | LINC02525 | 0.495723963 | 1.98E-32 | postive |
| CHMP5 | B4GALT1-AS1 | 0.46203624 | 7.32E-28 | postive |
| SLC7A11 | AC010731.2 | 0.476067648 | 1.06E-29 | postive |
| AKR1C1 | AC010731.2 | 0.455247344 | 5.32E-27 | postive |
| AKR1C3 | AC010731.2 | 0.485795037 | 4.96E-31 | postive |
| GCLC | AC010731.2 | 0.431517137 | 3.87E-24 | postive |
| TXNRD1 | AC010731.2 | 0.610934369 | 1.39E-52 | postive |
| SRXN1 | AC010731.2 | 0.548849161 | 9.37E-41 | postive |
| GPX2 | AC010731.2 | 0.465369341 | 2.72E-28 | postive |
| TFRC | AC010731.2 | 0.411269853 | 7.18E-22 | postive |
| MAFG | AC010731.2 | 0.48981439 | 1.36E-31 | postive |
| G6PD | AC010731.2 | 0.422854389 | 3.77E-23 | postive |
| PGD | AC010731.2 | 0.47845814 | 5.02E-30 | postive |
| ABCC1 | AC010731.2 | 0.608650781 | 4.21E-52 | postive |
| IDH1 | AC010731.2 | 0.479428265 | 3.71E-30 | postive |
| SLC7A11 | LINC00942 | 0.46046478 | 1.16E-27 | postive |
| NQO1 | LINC00942 | 0.452382332 | 1.21E-26 | postive |
| TXNRD1 | LINC00942 | 0.53512149 | 1.84E-38 | postive |
| SRXN1 | LINC00942 | 0.484708268 | 7.02E-31 | postive |
| MAFG | LINC00942 | 0.442499331 | 1.95E-25 | postive |
| G6PD | LINC00942 | 0.464953603 | 3.08E-28 | postive |
| PGD | LINC00942 | 0.504460437 | 1.06E-33 | postive |
| ABCC1 | LINC00942 | 0.416201892 | 2.08E-22 | postive |
| ALOX12B | AC244205.1 | 0.405165181 | 3.24E-21 | postive |
| IREB2 | TRAPPC12-AS1 | 0.414655247 | 3.07E-22 | postive |
| SP1 | TRAPPC12-AS1 | 0.464688513 | 3.34E-28 | postive |
| ACVR1B | TRAPPC12-AS1 | 0.415308607 | 2.61E-22 | postive |
| ZNF419 | AC010326.3 | 0.598104261 | 6.31E-50 | postive |
| PHKG2 | AC010326.3 | 0.449550595 | 2.71E-26 | postive |
| EGLN2 | AC010326.3 | 0.424330508 | 2.57E-23 | postive |
| PHKG2 | AC004908.2 | 0.400308656 | 1.05E-20 | postive |
| FANCD2 | NFYC-AS1 | 0.414951569 | 2.85E-22 | postive |
| HELLS | NFYC-AS1 | 0.554620763 | 9.44E-42 | postive |
| KLHL24 | NFYC-AS1 | 0.406237009 | 2.49E-21 | postive |
| TUBE1 | NFYC-AS1 | 0.543275901 | 8.24E-40 | postive |
| ELAVL1 | NFYC-AS1 | 0.411598787 | 6.62E-22 | postive |
| SP1 | NFYC-AS1 | 0.422516374 | 4.12E-23 | postive |
| STMN1 | NFYC-AS1 | 0.416091867 | 2.14E-22 | postive |
| GABPB1 | NFYC-AS1 | 0.48403593 | 8.69E-31 | postive |
| GLS2 | NFYC-AS1 | 0.519365467 | 5.92E-36 | postive |
| SNX4 | NFYC-AS1 | 0.405627089 | 2.89E-21 | postive |
| MAPK8 | NFYC-AS1 | 0.425572982 | 1.86E-23 | postive |
| ACVR1B | NFYC-AS1 | 0.441716946 | 2.43E-25 | postive |
| YY1AP1 | NFYC-AS1 | 0.403914337 | 4.40E-21 | postive |
| SIRT1 | NFYC-AS1 | 0.405558143 | 2.94E-21 | postive |
| GPX4 | AC024896.1 | 0.431486129 | 3.90E-24 | postive |
| CISD1 | AC024896.1 | 0.422354736 | 4.30E-23 | postive |
| FANCD2 | AC024896.1 | 0.420739021 | 6.52E-23 | postive |
| TP53 | AC024896.1 | 0.402617176 | 6.02E-21 | postive |
| HELLS | AC024896.1 | 0.548338998 | 1.15E-40 | postive |
| ISCU | AC024896.1 | 0.60334685 | 5.35E-51 | postive |
| STMN1 | AC024896.1 | 0.418988921 | 1.02E-22 | postive |
| NOX1 | AC024896.1 | 0.475054403 | 1.44E-29 | postive |
| SOCS1 | AC024896.1 | 0.411883736 | 6.16E-22 | postive |
| MYB | AC024896.1 | 0.537163032 | 8.54E-39 | postive |
| ATM | AP001318.2 | 0.427389347 | 1.15E-23 | postive |
| ELAVL1 | KDM4A-AS1 | 0.412839334 | 4.85E-22 | postive |
| GLS2 | KDM4A-AS1 | 0.412927026 | 4.74E-22 | postive |
| GPX4 | AC099343.3 | 0.405044021 | 3.34E-21 | postive |
| CISD1 | AC099343.3 | 0.420258388 | 7.38E-23 | postive |
| ATF4 | AC099343.3 | 0.403167898 | 5.27E-21 | postive |
| ISCU | AC099343.3 | 0.607729168 | 6.57E-52 | postive |
| STMN1 | AC099343.3 | 0.40618941 | 2.52E-21 | postive |
| NOX1 | AC099343.3 | 0.440226141 | 3.66E-25 | postive |
| PHKG2 | AC099343.3 | 0.420644498 | 6.68E-23 | postive |
| ATG4D | AC099343.3 | 0.410707781 | 8.26E-22 | postive |
| SOCS1 | AC099343.3 | 0.435257607 | 1.42E-24 | postive |
| HIC1 | AL096865.1 | 0.417020829 | 1.69E-22 | postive |
| STMN1 | AC005076.2 | 0.426406837 | 1.49E-23 | postive |
| NOX1 | AC005076.2 | 0.409182856 | 1.21E-21 | postive |
| PHKG2 | AC005076.2 | 0.464027222 | 4.06E-28 | postive |
| PEBP1 | AC005076.2 | 0.405258421 | 3.17E-21 | postive |
| TAZ | AC005076.2 | 0.481685402 | 1.83E-30 | postive |
| MYB | LINC02613 | 0.418767542 | 1.08E-22 | postive |
| CISD1 | SNHG12 | 0.405585145 | 2.92E-21 | postive |
| ATF4 | SNHG12 | 0.482056245 | 1.63E-30 | postive |
| FH | SNHG12 | 0.400624279 | 9.74E-21 | postive |
| ISCU | SNHG12 | 0.476680412 | 8.73E-30 | postive |
| STMN1 | SNHG12 | 0.446366346 | 6.65E-26 | postive |
| NOX1 | SNHG12 | 0.422785958 | 3.84E-23 | postive |
| PHKG2 | SNHG12 | 0.509701757 | 1.76E-34 | postive |
| TAZ | SNHG12 | 0.589521156 | 3.26E-48 | postive |
| FANCD2 | PTOV1-AS1 | 0.409015996 | 1.26E-21 | postive |
| HELLS | PTOV1-AS1 | 0.450340685 | 2.17E-26 | postive |
| ISCU | PTOV1-AS1 | 0.570637073 | 1.27E-44 | postive |
| STMN1 | PTOV1-AS1 | 0.543485732 | 7.60E-40 | postive |
| NOX1 | PTOV1-AS1 | 0.489942342 | 1.31E-31 | postive |
| CDKN2A | PTOV1-AS1 | 0.439006859 | 5.11E-25 | postive |
| KLHL24 | KCNJ2-AS1 | 0.449917056 | 2.44E-26 | postive |
| GABARAPL1 | KCNJ2-AS1 | 0.408870419 | 1.30E-21 | postive |
| GPX4 | TTC28-AS1 | 0.445243028 | 9.11E-26 | postive |
| ATF4 | TTC28-AS1 | 0.450904399 | 1.85E-26 | postive |
| ISCU | TTC28-AS1 | 0.431867715 | 3.52E-24 | postive |
| TUBE1 | TTC28-AS1 | 0.457185516 | 3.03E-27 | postive |
| STMN1 | TTC28-AS1 | 0.459756337 | 1.43E-27 | postive |
| NOX1 | TTC28-AS1 | 0.425031885 | 2.14E-23 | postive |
| PHKG2 | TTC28-AS1 | 0.461405307 | 8.82E-28 | postive |
| ATG4D | TTC28-AS1 | 0.414107322 | 3.53E-22 | postive |
| BID | TTC28-AS1 | 0.429166093 | 7.22E-24 | postive |
| TAZ | TTC28-AS1 | 0.441046688 | 2.92E-25 | postive |
| PANX1 | TTC28-AS1 | -0.407076963 | 2.03E-21 | negative |
| TP53 | AL391121.1 | 0.41885714 | 1.06E-22 | postive |
| HELLS | AL391121.1 | 0.434132645 | 1.92E-24 | postive |
| STAT3 | AL391121.1 | 0.451970467 | 1.36E-26 | postive |
| AGPAT3 | AL391121.1 | 0.401010655 | 8.88E-21 | postive |
| SP1 | AL391121.1 | 0.490060525 | 1.26E-31 | postive |
| GLS2 | AL391121.1 | 0.540917965 | 2.04E-39 | postive |
| NCOA4 | AL391121.1 | 0.472267446 | 3.39E-29 | postive |
| MAPK8 | AL391121.1 | 0.586863117 | 1.08E-47 | postive |
| SIRT1 | AL391121.1 | 0.511687102 | 8.86E-35 | postive |
| ISCU | AL645933.3 | 0.415800636 | 2.30E-22 | postive |
| PHKG2 | AL162724.2 | 0.436596554 | 9.85E-25 | postive |
| AIFM2 | AC114488.1 | 0.419241805 | 9.58E-23 | postive |
| MT3 | AC114488.1 | 0.441971517 | 2.26E-25 | postive |
| CDO1 | AC114488.1 | 0.609136158 | 3.32E-52 | postive |
| HIC1 | LINC02544 | 0.453912665 | 7.81E-27 | postive |
| NOX4 | LINC02544 | 0.483181943 | 1.14E-30 | postive |
| SOCS1 | AP001062.1 | 0.444948554 | 9.89E-26 | postive |
| MYB | AP001062.1 | 0.489398668 | 1.56E-31 | postive |
| HELLS | AL354733.3 | 0.452544303 | 1.16E-26 | postive |
| SP1 | AL354733.3 | 0.461796301 | 7.86E-28 | postive |
| ACVR1B | AL354733.3 | 0.431523712 | 3.86E-24 | postive |
| CAV1 | LINC00941 | 0.422965394 | 3.67E-23 | postive |
| IFNG | PSMB8-AS1 | 0.476021507 | 1.07E-29 | postive |
| PHKG2 | SNHG9 | 0.596860826 | 1.13E-49 | postive |
| EGLN2 | SNHG9 | 0.406668002 | 2.24E-21 | postive |
| MTOR | Z68871.1 | 0.480722777 | 2.47E-30 | postive |
| BRD4 | Z68871.1 | 0.42908832 | 7.37E-24 | postive |
| LAMP2 | Z68871.1 | 0.458344233 | 2.16E-27 | postive |
| KLHL24 | Z68871.1 | 0.418580312 | 1.13E-22 | postive |
| SETD1B | Z68871.1 | 0.514715057 | 3.07E-35 | postive |
| IREB2 | Z68871.1 | 0.480677043 | 2.51E-30 | postive |
| SP1 | Z68871.1 | 0.563439352 | 2.60E-43 | postive |
| CS | Z68871.1 | 0.403783033 | 4.54E-21 | postive |
| PIK3CA | Z68871.1 | 0.458408178 | 2.12E-27 | postive |
| LPCAT3 | Z68871.1 | 0.468575133 | 1.04E-28 | postive |
| MAPK8 | Z68871.1 | 0.43352286 | 2.26E-24 | postive |
| ACVR1B | Z68871.1 | 0.431725078 | 3.66E-24 | postive |
| ATM | Z68871.1 | 0.487526423 | 2.85E-31 | postive |
| SIRT1 | Z68871.1 | 0.42706767 | 1.26E-23 | postive |
| TAZ | LINC01431 | 0.426792577 | 1.35E-23 | postive |
| NFE2L2 | AL512625.2 | 0.454053785 | 7.50E-27 | postive |
| BRD4 | AL512625.2 | 0.43018792 | 5.51E-24 | postive |
| KLHL24 | AL512625.2 | 0.439769783 | 4.14E-25 | postive |
| MAFG | AL512625.2 | 0.42416073 | 2.69E-23 | postive |
| PIK3CA | AL512625.2 | 0.404337063 | 3.97E-21 | postive |
| ABCC1 | AL512625.2 | 0.407292849 | 1.92E-21 | postive |
| HERPUD1 | DTNB-AS1 | 0.649045293 | 3.01E-61 | postive |
| FLT3 | DTNB-AS1 | 0.4983444 | 8.30E-33 | postive |
| GPX4 | AC100810.1 | 0.45792344 | 2.45E-27 | postive |
| PHKG2 | AC100810.1 | 0.455795661 | 4.54E-27 | postive |
| MAP1LC3A | AC100810.1 | 0.400337798 | 1.04E-20 | postive |
| TAZ | AC100810.1 | 0.431405121 | 3.98E-24 | postive |
| PTGS2 | DELEC1 | 0.449838807 | 2.50E-26 | postive |
| GPX4 | AC004069.1 | 0.414062924 | 3.57E-22 | postive |
| STMN1 | AC004069.1 | 0.453833788 | 7.99E-27 | postive |
| NOX1 | AC004069.1 | 0.538351993 | 5.44E-39 | postive |
| PHKG2 | AC004069.1 | 0.445967634 | 7.44E-26 | postive |
| PEBP1 | AC004069.1 | 0.452237949 | 1.26E-26 | postive |
| TAZ | AC004069.1 | 0.515762641 | 2.13E-35 | postive |
| BRD4 | AC245060.2 | 0.418119218 | 1.28E-22 | postive |
| SP1 | AC245060.2 | 0.459335953 | 1.62E-27 | postive |
| ACVR1B | AC245060.2 | 0.40536761 | 3.08E-21 | postive |
| ATM | AC245060.2 | 0.470932718 | 5.09E-29 | postive |
| CISD1 | AC034213.1 | 0.435766545 | 1.23E-24 | postive |
| CARS1 | AC034213.1 | 0.463699766 | 4.48E-28 | postive |
| STMN1 | TTTY14 | 0.442157803 | 2.15E-25 | postive |
| GABPB1 | AC144652.1 | 0.419798338 | 8.30E-23 | postive |
| CHMP6 | AL132712.1 | 0.418517445 | 1.15E-22 | postive |
| PHKG2 | AL132712.1 | 0.426988043 | 1.28E-23 | postive |
| STMN1 | CCNT2-AS1 | 0.43407294 | 1.95E-24 | postive |
| ELAVL1 | AC024575.2 | 0.432921882 | 2.65E-24 | postive |
| KEAP1 | AC024575.2 | 0.446926536 | 5.69E-26 | postive |
| ATG4D | AC024575.2 | 0.458204903 | 2.25E-27 | postive |
| MYB | AC024575.2 | 0.417779586 | 1.39E-22 | postive |
| ISCU | AL662844.4 | 0.517182054 | 1.29E-35 | postive |
| PLIN4 | AL662844.4 | 0.492659493 | 5.41E-32 | postive |
| FLT3 | AL662844.4 | 0.411018585 | 7.65E-22 | postive |
| PRKAA2 | AL662844.4 | 0.545823413 | 3.07E-40 | postive |
| HELLS | AC092119.2 | 0.475571505 | 1.23E-29 | postive |
| ISCU | AC092119.2 | 0.526799518 | 4.04E-37 | postive |
| STMN1 | AC092119.2 | 0.462348466 | 6.68E-28 | postive |
| NOX1 | AC092119.2 | 0.41689914 | 1.74E-22 | postive |
| PHKG2 | AC092119.2 | 0.477581163 | 6.60E-30 | postive |
| MYB | AC092119.2 | 0.427603425 | 1.09E-23 | postive |
| TAZ | AC092119.2 | 0.464844712 | 3.18E-28 | postive |
| GPX2 | AL357079.1 | 0.421409154 | 5.48E-23 | postive |
| HIC1 | AC080038.2 | 0.467583361 | 1.40E-28 | postive |
| HELLS | AC120114.1 | 0.43647915 | 1.02E-24 | postive |
| FADS2 | AC120114.1 | 0.451525238 | 1.55E-26 | postive |
| BRD4 | AC120114.1 | 0.451403523 | 1.60E-26 | postive |
| KLHL24 | AC120114.1 | 0.46185839 | 7.72E-28 | postive |
| SP1 | AC120114.1 | 0.530606526 | 9.95E-38 | postive |
| PIK3CA | AC120114.1 | 0.463248302 | 5.12E-28 | postive |
| ACVR1B | AC120114.1 | 0.441837794 | 2.35E-25 | postive |
| ATM | AC120114.1 | 0.474542732 | 1.69E-29 | postive |
| YY1AP1 | AC120114.1 | 0.427065776 | 1.26E-23 | postive |
| SIRT1 | AC120114.1 | 0.41153289 | 6.73E-22 | postive |
| PHKG2 | AL359881.1 | 0.443829333 | 1.35E-25 | postive |
| GPX4 | LINC00339 | 0.453251955 | 9.44E-27 | postive |
| HELLS | LINC00339 | 0.402363004 | 6.40E-21 | postive |
| ISCU | LINC00339 | 0.480585925 | 2.58E-30 | postive |
| STMN1 | LINC00339 | 0.543121937 | 8.75E-40 | postive |
| NOX1 | LINC00339 | 0.400952228 | 9.00E-21 | postive |
| PHKG2 | LINC00339 | 0.407088534 | 2.02E-21 | postive |
| TAZ | LINC00339 | 0.431787523 | 3.60E-24 | postive |
| MT3 | AC104958.2 | 0.560534786 | 8.58E-43 | postive |
| CDO1 | AC104958.2 | 0.656502053 | 4.32E-63 | postive |
| HELLS | THAP9-AS1 | 0.451536048 | 1.54E-26 | postive |
| CISD2 | THAP9-AS1 | 0.437484732 | 7.74E-25 | postive |
| TUBE1 | THAP9-AS1 | 0.417737435 | 1.41E-22 | postive |
| ELAVL1 | THAP9-AS1 | 0.43309721 | 2.53E-24 | postive |
| STMN1 | THAP9-AS1 | 0.478956944 | 4.30E-30 | postive |
| NOX1 | THAP9-AS1 | 0.470823609 | 5.27E-29 | postive |
| ATG3 | THAP9-AS1 | 0.4275514 | 1.11E-23 | postive |
| GPX4 | SNHG11 | 0.424905716 | 2.21E-23 | postive |
| ATF4 | SNHG11 | 0.43832802 | 6.15E-25 | postive |
| NFS1 | SNHG11 | 0.45039578 | 2.13E-26 | postive |
| CHMP6 | SNHG11 | 0.455183802 | 5.42E-27 | postive |
| SLC2A8 | SNHG11 | 0.444495214 | 1.12E-25 | postive |
| PHKG2 | SNHG11 | 0.611720235 | 9.44E-53 | postive |
| EGLN2 | SNHG11 | 0.505113989 | 8.50E-34 | postive |
| TAZ | SNHG11 | 0.563233791 | 2.83E-43 | postive |
| HELLS | PSPC1-AS2 | 0.450263035 | 2.22E-26 | postive |
| HMGB1 | PSPC1-AS2 | 0.461503733 | 8.57E-28 | postive |
| STMN1 | PSPC1-AS2 | 0.400163407 | 1.09E-20 | postive |
| MYB | PSPC1-AS2 | 0.445191172 | 9.24E-26 | postive |
| TUBE1 | AL031600.1 | 0.415828562 | 2.28E-22 | postive |
| GPX4 | AC011374.2 | 0.431825683 | 3.56E-24 | postive |
| CISD1 | AC011374.2 | 0.466487757 | 1.95E-28 | postive |
| ISCU | AC011374.2 | 0.434889941 | 1.56E-24 | postive |
| NOX1 | AC011374.2 | 0.499910662 | 4.92E-33 | postive |
| PHKG2 | AC011374.2 | 0.40965447 | 1.07E-21 | postive |
| FANCD2 | AC097534.1 | 0.509452221 | 1.92E-34 | postive |
| TP53 | AC097534.1 | 0.467658449 | 1.37E-28 | postive |
| HELLS | AC097534.1 | 0.632498644 | 2.45E-57 | postive |
| ISCU | AC097534.1 | 0.423194226 | 3.46E-23 | postive |
| SP1 | AC097534.1 | 0.4680847 | 1.21E-28 | postive |
| STMN1 | AC097534.1 | 0.500684056 | 3.80E-33 | postive |
| GLS2 | AC097534.1 | 0.509384631 | 1.97E-34 | postive |
| MYB | AC097534.1 | 0.445260171 | 9.07E-26 | postive |
| MAPK8 | AC097534.1 | 0.477259006 | 7.29E-30 | postive |
| AKR1C1 | AC092115.4 | 0.452558871 | 1.15E-26 | postive |
| AKR1C2 | AC092115.4 | 0.466455503 | 1.97E-28 | postive |
| AKR1C3 | AC092115.4 | 0.474297867 | 1.82E-29 | postive |
| NQO1 | AC092115.4 | 0.53528657 | 1.73E-38 | postive |
| AIFM2 | AC092115.4 | 0.439855976 | 4.05E-25 | postive |
| TXNRD1 | AC092115.4 | 0.450005219 | 2.38E-26 | postive |
| GPX2 | AC092115.4 | 0.577632293 | 6.33E-46 | postive |
| ASNS | AC092115.4 | 0.416647964 | 1.86E-22 | postive |
| PRDX1 | AC092115.4 | 0.457306914 | 2.93E-27 | postive |
| G6PD | AC092115.4 | 0.425838499 | 1.73E-23 | postive |
| TAZ | HMGA1P4 | 0.403549619 | 4.80E-21 | postive |
| GPX4 | AC007114.1 | 0.419021549 | 1.01E-22 | postive |
| CISD1 | AC007114.1 | 0.406427089 | 2.38E-21 | postive |
| NOX1 | AC007114.1 | 0.422143756 | 4.54E-23 | postive |
| PHKG2 | AC007114.1 | 0.413382322 | 4.23E-22 | postive |
| TAZ | AC007114.1 | 0.451491079 | 1.56E-26 | postive |
| NFE2L2 | AC079848.1 | 0.418551623 | 1.14E-22 | postive |
| KLHL24 | AC079848.1 | 0.508928113 | 2.30E-34 | postive |
| SNX4 | AC079848.1 | 0.49652724 | 1.52E-32 | postive |
| ACVR1B | AC079848.1 | 0.4169674 | 1.71E-22 | postive |
| YY1AP1 | AC079848.1 | 0.470690142 | 5.48E-29 | postive |
| PROM2 | STEAP3-AS1 | 0.45330123 | 9.31E-27 | postive |
| STEAP3 | STEAP3-AS1 | 0.477335526 | 7.12E-30 | postive |
| CHMP6 | AL121832.2 | 0.482462068 | 1.43E-30 | postive |
| RPL8 | AL121832.2 | 0.419854876 | 8.18E-23 | postive |
| SLC2A8 | AL121832.2 | 0.416790694 | 1.79E-22 | postive |
| PHKG2 | AL121832.2 | 0.589865866 | 2.78E-48 | postive |
| EGLN2 | AL121832.2 | 0.534143988 | 2.66E-38 | postive |
| TAZ | AL121832.2 | 0.473333691 | 2.45E-29 | postive |
| MAFG | AC008443.3 | 0.461163124 | 9.48E-28 | postive |
| PHKG2 | AL732292.2 | 0.427479205 | 1.13E-23 | postive |
| EGLN2 | AL732292.2 | 0.424301633 | 2.59E-23 | postive |
| CDO1 | AL136962.1 | 0.499487059 | 5.67E-33 | postive |
| MUC1 | AC004233.2 | 0.444390669 | 1.16E-25 | postive |
| TUBE1 | AC002128.1 | 0.442460435 | 1.97E-25 | postive |
| AKR1C1 | SAMD12-AS1 | 0.45567736 | 4.70E-27 | postive |
| AKR1C3 | SAMD12-AS1 | 0.434153934 | 1.91E-24 | postive |
| GCLC | SAMD12-AS1 | 0.531604898 | 6.87E-38 | postive |
| NFE2L2 | SAMD12-AS1 | 0.511793107 | 8.53E-35 | postive |
| TXNRD1 | SAMD12-AS1 | 0.641616236 | 1.84E-59 | postive |
| SRXN1 | SAMD12-AS1 | 0.51730651 | 1.23E-35 | postive |
| GPX2 | SAMD12-AS1 | 0.581412607 | 1.21E-46 | postive |
| ASNS | SAMD12-AS1 | 0.512355067 | 7.02E-35 | postive |
| PCK2 | SAMD12-AS1 | 0.418854 | 1.06E-22 | postive |
| PSAT1 | SAMD12-AS1 | 0.494274265 | 3.19E-32 | postive |
| TUBE1 | SAMD12-AS1 | 0.423862315 | 2.91E-23 | postive |
| FTL | SAMD12-AS1 | 0.415645188 | 2.39E-22 | postive |
| TFRC | SAMD12-AS1 | 0.442771149 | 1.81E-25 | postive |
| MAFG | SAMD12-AS1 | 0.44535711 | 8.82E-26 | postive |
| G6PD | SAMD12-AS1 | 0.425502816 | 1.89E-23 | postive |
| PGD | SAMD12-AS1 | 0.47613431 | 1.03E-29 | postive |
| KEAP1 | SAMD12-AS1 | 0.411893579 | 6.15E-22 | postive |
| GABARAPL1 | SAMD12-AS1 | 0.506401396 | 5.48E-34 | postive |
| ABCC1 | SAMD12-AS1 | 0.543031213 | 9.06E-40 | postive |
| IDH1 | SAMD12-AS1 | 0.487591344 | 2.79E-31 | postive |
| YY1AP1 | AC093627.4 | 0.456656345 | 3.54E-27 | postive |
| FANCD2 | AC007996.1 | 0.410899844 | 7.88E-22 | postive |
| HELLS | AC007996.1 | 0.493454746 | 4.17E-32 | postive |
| ELAVL1 | AC007996.1 | 0.501788442 | 2.62E-33 | postive |
| GLS2 | AC007996.1 | 0.546576179 | 2.29E-40 | postive |
| MYB | AC007996.1 | 0.424744836 | 2.31E-23 | postive |
| CHMP6 | AL118558.3 | 0.409651702 | 1.07E-21 | postive |
| PHKG2 | AL118558.3 | 0.456877276 | 3.32E-27 | postive |
| EGLN2 | AL118558.3 | 0.423843134 | 2.92E-23 | postive |
| TAZ | AL118558.3 | 0.419672829 | 8.57E-23 | postive |
| ZNF419 | AC006213.4 | 0.546068346 | 2.79E-40 | postive |
| MYB | CEROX1 | 0.501911187 | 2.51E-33 | postive |
| GABPB1 | LOH12CR2 | 0.402733105 | 5.86E-21 | postive |
| GABARAPL1 | LOH12CR2 | 0.421394585 | 5.51E-23 | postive |
| HELLS | AC022098.1 | 0.493376649 | 4.28E-32 | postive |
| STMN1 | AC022098.1 | 0.479000777 | 4.24E-30 | postive |
| GABPB1 | AC022098.1 | 0.40849315 | 1.43E-21 | postive |
| PHKG2 | AC022098.1 | 0.42161623 | 5.20E-23 | postive |
| TAZ | AC022098.1 | 0.456982131 | 3.22E-27 | postive |
| GPX4 | AC139768.1 | 0.406224556 | 2.50E-21 | postive |
| ISCU | AC139768.1 | 0.476255103 | 9.96E-30 | postive |
| STMN1 | AC139768.1 | 0.437554344 | 7.59E-25 | postive |
| GABPB1 | AC139768.1 | 0.404295354 | 4.01E-21 | postive |
| PEBP1 | AC139768.1 | 0.448808619 | 3.35E-26 | postive |
| NNMT | LINC00702 | 0.569178592 | 2.36E-44 | postive |
| PLIN4 | LINC00702 | 0.655243591 | 8.92E-63 | postive |
| ZEB1 | LINC00702 | 0.558449661 | 2.01E-42 | postive |
| PRKAA2 | LINC00702 | 0.758378619 | 8.32E-95 | postive |
| FANCD2 | ASB16-AS1 | 0.515342916 | 2.46E-35 | postive |
| TP53 | ASB16-AS1 | 0.414170177 | 3.47E-22 | postive |
| HELLS | ASB16-AS1 | 0.548704832 | 9.92E-41 | postive |
| ISCU | ASB16-AS1 | 0.5307926 | 9.28E-38 | postive |
| TUBE1 | ASB16-AS1 | 0.410599988 | 8.49E-22 | postive |
| ELAVL1 | ASB16-AS1 | 0.510342663 | 1.41E-34 | postive |
| STMN1 | ASB16-AS1 | 0.533814312 | 3.01E-38 | postive |
| NOX1 | ASB16-AS1 | 0.506906937 | 4.61E-34 | postive |
| GLS2 | ASB16-AS1 | 0.448432515 | 3.72E-26 | postive |
| KEAP1 | ASB16-AS1 | 0.424415091 | 2.52E-23 | postive |
| PHKG2 | ASB16-AS1 | 0.428521699 | 8.56E-24 | postive |
| ATG3 | ASB16-AS1 | 0.430335445 | 5.30E-24 | postive |
| ATG4D | ASB16-AS1 | 0.469324801 | 8.30E-29 | postive |
| MYB | ASB16-AS1 | 0.491552668 | 7.76E-32 | postive |
| YY1AP1 | ASB16-AS1 | 0.447742225 | 4.52E-26 | postive |
| TAZ | ASB16-AS1 | 0.442656957 | 1.87E-25 | postive |
| HELLS | LENG8-AS1 | 0.413487765 | 4.12E-22 | postive |
| ZNF419 | LENG8-AS1 | 0.436105844 | 1.13E-24 | postive |
| TUBE1 | LENG8-AS1 | 0.438451589 | 5.95E-25 | postive |
| GABPB1 | LENG8-AS1 | 0.424533316 | 2.44E-23 | postive |
| ALOX12B | SNHG28 | 0.592578008 | 8.10E-49 | postive |
| ALOXE3 | SNHG28 | 0.448818595 | 3.34E-26 | postive |
| PHKG2 | PARD3-AS1 | 0.402509515 | 6.18E-21 | postive |
| SP1 | AC007342.4 | 0.402356538 | 6.41E-21 | postive |
| LPCAT3 | AC007342.4 | 0.407465133 | 1.84E-21 | postive |
| YY1AP1 | AC007342.4 | 0.401314818 | 8.25E-21 | postive |
| SLC40A1 | AC090559.1 | 0.60287423 | 6.70E-51 | postive |
| ENPP2 | AC090559.1 | 0.666165475 | 1.47E-65 | postive |
| ALOX5 | AC090559.1 | 0.747220534 | 1.34E-90 | postive |
| CYBB | AC090559.1 | 0.861013135 | 1.24E-148 | postive |
| FLT3 | AC090559.1 | 0.608645607 | 4.22E-52 | postive |
| ZEB1 | AC090559.1 | 0.593176237 | 6.16E-49 | postive |
| TLR4 | AC090559.1 | 0.757619148 | 1.64E-94 | postive |
| ATM | AC090559.1 | 0.465205596 | 2.86E-28 | postive |
| NOS2 | RHOXF1-AS1 | 0.482903619 | 1.24E-30 | postive |
| NOX1 | RHOXF1-AS1 | 0.412053747 | 5.90E-22 | postive |
| ATM | SNHG14 | 0.405089756 | 3.30E-21 | postive |
| GPX2 | AL591501.1 | 0.441998343 | 2.24E-25 | postive |
| ISCU | AC015726.1 | 0.408717371 | 1.35E-21 | postive |
| GLS2 | LINC01376 | 0.404390507 | 3.91E-21 | postive |
| TUBE1 | AL021707.7 | 0.4088395 | 1.31E-21 | postive |
| PHKG2 | AL021707.7 | 0.413653332 | 3.95E-22 | postive |
| FANCD2 | AC005696.1 | 0.450475534 | 2.09E-26 | postive |
| HELLS | AC005696.1 | 0.498966194 | 6.74E-33 | postive |
| ISCU | AC005696.1 | 0.495669125 | 2.01E-32 | postive |
| ELAVL1 | AC005696.1 | 0.419334209 | 9.35E-23 | postive |
| STMN1 | AC005696.1 | 0.486368425 | 4.13E-31 | postive |
| NOX1 | AC005696.1 | 0.406819825 | 2.16E-21 | postive |
| GLS2 | AC005696.1 | 0.514722931 | 3.06E-35 | postive |
| GOT1 | AC005696.1 | 0.573890279 | 3.18E-45 | postive |
| KEAP1 | AC005696.1 | 0.406958493 | 2.09E-21 | postive |
| ATG3 | AC005696.1 | 0.442332173 | 2.05E-25 | postive |
| ATG4D | AC005696.1 | 0.553123156 | 1.72E-41 | postive |
| PHKG2 | STARD7-AS1 | 0.505464556 | 7.54E-34 | postive |
| MAP1LC3A | STARD7-AS1 | 0.414237908 | 3.41E-22 | postive |
| TAZ | STARD7-AS1 | 0.468050408 | 1.22E-28 | postive |
| HELLS | AL109811.2 | 0.400899403 | 9.12E-21 | postive |
| ISCU | AL109811.2 | 0.478886621 | 4.39E-30 | postive |
| STMN1 | AL109811.2 | 0.443471962 | 1.49E-25 | postive |
| NOX1 | AL109811.2 | 0.410293691 | 9.16E-22 | postive |
| PHKG2 | AL109811.2 | 0.461720273 | 8.04E-28 | postive |
| MYB | AL109811.2 | 0.457859579 | 2.49E-27 | postive |
| TAZ | AL109811.2 | 0.427566775 | 1.10E-23 | postive |
| GPX4 | SCAT2 | 0.426059259 | 1.64E-23 | postive |
| CISD1 | SCAT2 | 0.406993761 | 2.07E-21 | postive |
| FANCD2 | SCAT2 | 0.42973755 | 6.21E-24 | postive |
| ATF4 | SCAT2 | 0.490835656 | 9.79E-32 | postive |
| HELLS | SCAT2 | 0.473083053 | 2.64E-29 | postive |
| ISCU | SCAT2 | 0.545740121 | 3.17E-40 | postive |
| SLC2A8 | SCAT2 | 0.408182575 | 1.54E-21 | postive |
| HMGB1 | SCAT2 | 0.466795462 | 1.78E-28 | postive |
| STMN1 | SCAT2 | 0.575875698 | 1.35E-45 | postive |
| NOX1 | SCAT2 | 0.534808094 | 2.08E-38 | postive |
| PHKG2 | SCAT2 | 0.553114413 | 1.73E-41 | postive |
| ATG4D | SCAT2 | 0.433343861 | 2.37E-24 | postive |
| SOCS1 | SCAT2 | 0.41734604 | 1.55E-22 | postive |
| TAZ | SCAT2 | 0.594505335 | 3.35E-49 | postive |
| FANCD2 | SREBF2-AS1 | 0.47566552 | 1.19E-29 | postive |
| ATF4 | SREBF2-AS1 | 0.504917948 | 9.09E-34 | postive |
| HELLS | SREBF2-AS1 | 0.510498496 | 1.34E-34 | postive |
| ISCU | SREBF2-AS1 | 0.544055758 | 6.09E-40 | postive |
| TUBE1 | SREBF2-AS1 | 0.452702509 | 1.11E-26 | postive |
| ELAVL1 | SREBF2-AS1 | 0.493093525 | 4.69E-32 | postive |
| STMN1 | SREBF2-AS1 | 0.473452656 | 2.36E-29 | postive |
| NOX1 | SREBF2-AS1 | 0.462631705 | 6.14E-28 | postive |
| GLS2 | SREBF2-AS1 | 0.440718386 | 3.19E-25 | postive |
| KEAP1 | SREBF2-AS1 | 0.410487865 | 8.73E-22 | postive |
| PHKG2 | SREBF2-AS1 | 0.41084783 | 7.98E-22 | postive |
| ATG4D | SREBF2-AS1 | 0.483485198 | 1.03E-30 | postive |
| YY1AP1 | SREBF2-AS1 | 0.408948792 | 1.28E-21 | postive |
| TAZ | SREBF2-AS1 | 0.431119672 | 4.30E-24 | postive |
| PANX1 | SREBF2-AS1 | -0.416417409 | 1.97E-22 | negative |
| GPX4 | LINC01315 | 0.569385447 | 2.16E-44 | postive |
| CISD1 | LINC01315 | 0.498690928 | 7.39E-33 | postive |
| ISCU | LINC01315 | 0.509757239 | 1.73E-34 | postive |
| GLS2 | LINC01315 | 0.402930998 | 5.58E-21 | postive |
| CARS1 | LINC01315 | 0.436698008 | 9.58E-25 | postive |
| MAP1LC3A | LINC01315 | 0.440831507 | 3.10E-25 | postive |
| CDKN2A | LINC01315 | 0.458857307 | 1.86E-27 | postive |
| SOCS1 | LINC01315 | 0.430867118 | 4.60E-24 | postive |
| MYB | AC022509.2 | 0.455644484 | 4.74E-27 | postive |
| ELAVL1 | AC006449.5 | 0.41990252 | 8.08E-23 | postive |
| STMN1 | AC006449.5 | 0.407605265 | 1.78E-21 | postive |
| GABPB1 | AC006449.5 | 0.416203765 | 2.08E-22 | postive |
| MYB | HOTAIR | 0.574521074 | 2.43E-45 | postive |
| NFE2L2 | LINC01876 | 0.431283967 | 4.11E-24 | postive |
| TUBE1 | LINC01876 | 0.423121578 | 3.52E-23 | postive |
| CAV1 | HS1BP3-IT1 | 0.450446093 | 2.10E-26 | postive |
| PANX1 | HS1BP3-IT1 | 0.405523212 | 2.97E-21 | postive |
| HIC1 | AC112721.2 | 0.505590626 | 7.23E-34 | postive |
| NOX4 | AC112721.2 | 0.54884838 | 9.37E-41 | postive |
| ZEB1 | AC112721.2 | 0.576608569 | 9.87E-46 | postive |
| KLHL24 | AC121757.1 | 0.410770321 | 8.13E-22 | postive |
| SP1 | AC121757.1 | 0.473186102 | 2.56E-29 | postive |
| ACVR1B | AC121757.1 | 0.463766709 | 4.39E-28 | postive |
| YY1AP1 | AC121757.1 | 0.444113899 | 1.25E-25 | postive |
| ISCU | AL513320.1 | 0.501121494 | 3.28E-33 | postive |
| ACSF2 | AL513320.1 | 0.410364779 | 9.00E-22 | postive |
| STMN1 | AL513320.1 | 0.413523373 | 4.09E-22 | postive |
| PHKG2 | AL513320.1 | 0.491064523 | 9.09E-32 | postive |
| TAZ | AL513320.1 | 0.481731261 | 1.80E-30 | postive |
| GPX4 | AC139530.1 | 0.445466738 | 8.56E-26 | postive |
| CHMP6 | AC139530.1 | 0.600917013 | 1.69E-50 | postive |
| SLC2A8 | AC139530.1 | 0.490763681 | 1.00E-31 | postive |
| STMN1 | AC139530.1 | 0.425547268 | 1.87E-23 | postive |
| PHKG2 | AC139530.1 | 0.686676123 | 4.07E-71 | postive |
| PEBP1 | AC139530.1 | 0.468760389 | 9.84E-29 | postive |
| ANO6 | AC139530.1 | -0.439303753 | 4.71E-25 | negative |
| EGLN2 | AC139530.1 | 0.550702992 | 4.51E-41 | postive |
| TAZ | AC139530.1 | 0.544396884 | 5.34E-40 | postive |
| LONP1 | AC139530.1 | 0.410801498 | 8.07E-22 | postive |
| TAZ | AC087741.1 | 0.422473786 | 4.17E-23 | postive |
| PHKG2 | AL451085.3 | 0.611504872 | 1.05E-52 | postive |
| MAP1LC3A | AL451085.3 | 0.484357938 | 7.84E-31 | postive |
| WIPI2 | AL451085.3 | 0.436616014 | 9.80E-25 | postive |
| EGLN2 | AL451085.3 | 0.401139194 | 8.61E-21 | postive |
| TAZ | AL451085.3 | 0.462796674 | 5.85E-28 | postive |
| BID | AL138760.1 | 0.423814923 | 2.94E-23 | postive |
| MIOX | AL138760.1 | 0.411268126 | 7.18E-22 | postive |
| FANCD2 | AC109454.2 | 0.414823656 | 2.94E-22 | postive |
| HELLS | AC109454.2 | 0.439516298 | 4.44E-25 | postive |
| STMN1 | AP000251.1 | 0.542170854 | 1.26E-39 | postive |
| NOX1 | AP000251.1 | 0.437005402 | 8.82E-25 | postive |
| PHKG2 | AP000251.1 | 0.540385133 | 2.50E-39 | postive |
| PEBP1 | AP000251.1 | 0.403088952 | 5.37E-21 | postive |
| TAZ | AP000251.1 | 0.470010351 | 6.74E-29 | postive |
| AKR1C1 | LINC01508 | 0.420916056 | 6.23E-23 | postive |
| AKR1C3 | LINC01508 | 0.522406709 | 1.99E-36 | postive |
| NQO1 | LINC01508 | 0.464537528 | 3.49E-28 | postive |
| TXNRD1 | LINC01508 | 0.53655416 | 1.07E-38 | postive |
| SRXN1 | LINC01508 | 0.445380526 | 8.77E-26 | postive |
| GPX2 | LINC01508 | 0.504428843 | 1.07E-33 | postive |
| G6PD | LINC01508 | 0.470503976 | 5.80E-29 | postive |
| PGD | LINC01508 | 0.414553646 | 3.15E-22 | postive |
| GPX4 | AL392172.1 | 0.595737767 | 1.89E-49 | postive |
| ATF4 | AL392172.1 | 0.422504131 | 4.13E-23 | postive |
| FH | AL392172.1 | 0.470569902 | 5.69E-29 | postive |
| ISCU | AL392172.1 | 0.476597591 | 8.95E-30 | postive |
| PRDX6 | AL392172.1 | 0.495191721 | 2.36E-32 | postive |
| STMN1 | AL392172.1 | 0.523904732 | 1.16E-36 | postive |
| NOX1 | AL392172.1 | 0.461261935 | 9.20E-28 | postive |
| PHKG2 | AL392172.1 | 0.535485349 | 1.61E-38 | postive |
| ATG4D | AL392172.1 | 0.406758139 | 2.19E-21 | postive |
| MAP1LC3A | AL392172.1 | 0.42348704 | 3.20E-23 | postive |
| PEBP1 | AL392172.1 | 0.401881489 | 7.20E-21 | postive |
| EGLN2 | AL392172.1 | 0.452081695 | 1.32E-26 | postive |
| TAZ | AL392172.1 | 0.46530795 | 2.77E-28 | postive |
| PANX1 | AL392172.1 | -0.404518512 | 3.79E-21 | negative |
| LONP1 | AL392172.1 | 0.42746879 | 1.13E-23 | postive |
| ALOX12B | LINC01094 | 0.484035648 | 8.69E-31 | postive |
| ALOXE3 | LINC01094 | 0.405863588 | 2.73E-21 | postive |
| FANCD2 | SNHG1 | 0.413013834 | 4.64E-22 | postive |
| ATF4 | SNHG1 | 0.53118353 | 8.03E-38 | postive |
| HELLS | SNHG1 | 0.497060777 | 1.27E-32 | postive |
| FH | SNHG1 | 0.422680007 | 3.95E-23 | postive |
| ISCU | SNHG1 | 0.41621042 | 2.07E-22 | postive |
| TUBE1 | SNHG1 | 0.496283333 | 1.64E-32 | postive |
| HMGB1 | SNHG1 | 0.481840027 | 1.74E-30 | postive |
| STMN1 | SNHG1 | 0.464685999 | 3.34E-28 | postive |
| GABPB1 | SNHG1 | 0.444244791 | 1.20E-25 | postive |
| PHKG2 | SNHG1 | 0.40759338 | 1.79E-21 | postive |
| ATG4D | SNHG1 | 0.400191969 | 1.08E-20 | postive |
| TAZ | SNHG1 | 0.504645493 | 9.97E-34 | postive |
| FLT3 | LINC00926 | 0.458984968 | 1.80E-27 | postive |
| MYB | LINC00926 | 0.463237967 | 5.13E-28 | postive |
| TUBE1 | AL353796.1 | 0.426026893 | 1.65E-23 | postive |
| GPX4 | AC105942.1 | 0.437264694 | 8.22E-25 | postive |
| CISD1 | AC105942.1 | 0.542776165 | 9.99E-40 | postive |
| ISCU | AC105942.1 | 0.456369074 | 3.84E-27 | postive |
| GLS2 | AC105942.1 | 0.419445649 | 9.09E-23 | postive |
| CARS1 | AC105942.1 | 0.435385904 | 1.37E-24 | postive |
| MAP1LC3A | AC105942.1 | 0.464168912 | 3.89E-28 | postive |
| FANCD2 | PCBP1-AS1 | 0.426177593 | 1.59E-23 | postive |
| HELLS | PCBP1-AS1 | 0.452816276 | 1.07E-26 | postive |
| ISCU | PCBP1-AS1 | 0.442712752 | 1.84E-25 | postive |
| TUBE1 | PCBP1-AS1 | 0.514520495 | 3.29E-35 | postive |
| ELAVL1 | PCBP1-AS1 | 0.411316214 | 7.10E-22 | postive |
| STMN1 | PCBP1-AS1 | 0.4238746 | 2.90E-23 | postive |
| GABPB1 | PCBP1-AS1 | 0.44317689 | 1.62E-25 | postive |
| NOX1 | PCBP1-AS1 | 0.418772838 | 1.08E-22 | postive |
| GLS2 | PCBP1-AS1 | 0.420589151 | 6.78E-23 | postive |
| ATG4D | PCBP1-AS1 | 0.415666091 | 2.38E-22 | postive |
| MYB | PCBP1-AS1 | 0.569640397 | 1.94E-44 | postive |
| LPIN1 | PCBP1-AS1 | 0.406977729 | 2.08E-21 | postive |
| LPCAT3 | C1RL-AS1 | 0.436616739 | 9.80E-25 | postive |
| TAZ | AC002553.2 | 0.436776057 | 9.38E-25 | postive |
| KLHL24 | EIF2AK3-DT | 0.490550986 | 1.07E-31 | postive |
| TUBE1 | EIF2AK3-DT | 0.42078778 | 6.44E-23 | postive |
| GABPB1 | EIF2AK3-DT | 0.42004362 | 7.80E-23 | postive |
| HSPB1 | MIR200CHG | 0.411638933 | 6.55E-22 | postive |
| RPL8 | MIR200CHG | 0.447560802 | 4.76E-26 | postive |
| HRAS | MIR200CHG | 0.414984297 | 2.83E-22 | postive |
| PHKG2 | MIR200CHG | 0.435990578 | 1.16E-24 | postive |
| EGLN2 | MIR200CHG | 0.521902482 | 2.39E-36 | postive |
| PHKG2 | AL139287.1 | 0.511467591 | 9.56E-35 | postive |
| TAZ | AL139287.1 | 0.402343082 | 6.44E-21 | postive |
| MUC1 | CPNE8-AS1 | 0.456538744 | 3.66E-27 | postive |
| KLHL24 | AL133367.1 | 0.421399949 | 5.50E-23 | postive |
| HIC1 | LINC01711 | 0.483796047 | 9.38E-31 | postive |
| PHKG2 | AC068338.3 | 0.486341563 | 4.17E-31 | postive |
| PEBP1 | AC068338.3 | 0.471658948 | 4.08E-29 | postive |
| EGLN2 | AC068338.3 | 0.453852855 | 7.94E-27 | postive |
| TAZ | AC068338.3 | 0.455724298 | 4.63E-27 | postive |
| PEBP1 | AC022706.1 | 0.40356567 | 4.78E-21 | postive |
| GPX4 | AC008669.1 | 0.414025295 | 3.60E-22 | postive |
| FANCD2 | AC008669.1 | 0.422294647 | 4.36E-23 | postive |
| TP53 | AC008669.1 | 0.441814294 | 2.36E-25 | postive |
| HELLS | AC008669.1 | 0.527201132 | 3.49E-37 | postive |
| ISCU | AC008669.1 | 0.569451434 | 2.10E-44 | postive |
| STMN1 | AC008669.1 | 0.475552634 | 1.24E-29 | postive |
| NOX1 | AC008669.1 | 0.472017789 | 3.66E-29 | postive |
| GLS2 | AC008669.1 | 0.549107182 | 8.47E-41 | postive |
| ATG4D | AC008669.1 | 0.427815401 | 1.03E-23 | postive |
| MAPK8 | AC008669.1 | 0.481553084 | 1.90E-30 | postive |
| ALOX12B | LINC01527 | 0.521063315 | 3.23E-36 | postive |
| ALOXE3 | LINC01527 | 0.510644719 | 1.27E-34 | postive |
| FANCD2 | LINC00852 | 0.516146671 | 1.86E-35 | postive |
| HELLS | LINC00852 | 0.513860797 | 4.15E-35 | postive |
| ISCU | LINC00852 | 0.486581203 | 3.86E-31 | postive |
| STMN1 | LINC00852 | 0.400248134 | 1.07E-20 | postive |
| NOX1 | LINC00852 | 0.415333106 | 2.59E-22 | postive |
| MYB | LINC00852 | 0.419610489 | 8.71E-23 | postive |
| PRDX6 | LINC01063 | 0.41607028 | 2.15E-22 | postive |
| PHKG2 | LINC01063 | 0.421713711 | 5.07E-23 | postive |
| EGLN2 | LINC01063 | 0.403400342 | 4.98E-21 | postive |
| TAZ | LINC01063 | 0.438588802 | 5.73E-25 | postive |
| ALOX5 | AC002091.2 | 0.408027613 | 1.60E-21 | postive |
| FLT3 | AC002091.2 | 0.401799319 | 7.34E-21 | postive |
| CHMP6 | AL136304.1 | 0.415336535 | 2.59E-22 | postive |
| PHKG2 | AL136304.1 | 0.564455599 | 1.70E-43 | postive |
| AIFM2 | AC099568.2 | 0.456659049 | 3.53E-27 | postive |
| ELAVL1 | AC099568.2 | 0.40737674 | 1.88E-21 | postive |
| PEBP1 | AC099568.2 | 0.461644716 | 8.22E-28 | postive |
| MIOX | AC099568.2 | 0.427781273 | 1.04E-23 | postive |
| GPX4 | CRNDE | 0.420469111 | 6.99E-23 | postive |
| ATF4 | CRNDE | 0.448816231 | 3.34E-26 | postive |
| FH | CRNDE | 0.458550818 | 2.04E-27 | postive |
| PRDX6 | CRNDE | 0.438415658 | 6.00E-25 | postive |
| HMGB1 | CRNDE | 0.401376718 | 8.13E-21 | postive |
| STMN1 | CRNDE | 0.554801855 | 8.78E-42 | postive |
| NOX1 | CRNDE | 0.478426339 | 5.07E-30 | postive |
| PHKG2 | CRNDE | 0.562461884 | 3.89E-43 | postive |
| PEBP1 | CRNDE | 0.464801771 | 3.23E-28 | postive |
| TAZ | CRNDE | 0.503425979 | 1.51E-33 | postive |
| PANX1 | CRNDE | -0.440520265 | 3.37E-25 | negative |
| FANCD2 | TMPO-AS1 | 0.423747635 | 2.99E-23 | postive |
| HELLS | TMPO-AS1 | 0.449644147 | 2.64E-26 | postive |
| HMGB1 | TMPO-AS1 | 0.400251692 | 1.07E-20 | postive |
| STMN1 | TMPO-AS1 | 0.545068379 | 4.11E-40 | postive |
| SLC2A6 | AL138724.2 | 0.416331876 | 2.01E-22 | postive |
| PML | NRIR | 0.447115234 | 5.39E-26 | postive |
| ISCU | LINC01011 | 0.416452326 | 1.95E-22 | postive |
| TAZ | LINC01011 | 0.403818752 | 4.50E-21 | postive |
| KRAS | AP005899.1 | 0.401681352 | 7.55E-21 | postive |
| ISCU | AC063948.1 | 0.418470871 | 1.17E-22 | postive |
| STMN1 | AC063948.1 | 0.473490854 | 2.33E-29 | postive |
| GPX4 | AC067838.1 | 0.521007453 | 3.29E-36 | postive |
| CISD1 | AC067838.1 | 0.439306026 | 4.71E-25 | postive |
| ATF4 | AC067838.1 | 0.407115057 | 2.01E-21 | postive |
| ISCU | AC067838.1 | 0.485989348 | 4.66E-31 | postive |
| STMN1 | AC067838.1 | 0.446486146 | 6.43E-26 | postive |
| NOX1 | AC067838.1 | 0.503593719 | 1.42E-33 | postive |
| PHKG2 | AC067838.1 | 0.495039052 | 2.48E-32 | postive |
| MAP1LC3A | AC067838.1 | 0.483940756 | 8.95E-31 | postive |
| PEBP1 | AC067838.1 | 0.48197561 | 1.67E-30 | postive |
| SOCS1 | AC067838.1 | 0.503662457 | 1.39E-33 | postive |
| EGLN2 | AC067838.1 | 0.419170523 | 9.75E-23 | postive |
| TAZ | AC067838.1 | 0.460643244 | 1.10E-27 | postive |
| GPX4 | AP002360.2 | 0.411961321 | 6.04E-22 | postive |
| PHKG2 | AP002360.2 | 0.537535741 | 7.41E-39 | postive |
| MAP1LC3A | AP002360.2 | 0.4459661 | 7.44E-26 | postive |
| TAZ | AP002360.2 | 0.415662096 | 2.38E-22 | postive |
| CAV1 | LINC02551 | 0.455245211 | 5.32E-27 | postive |
| ATF4 | SNHG20 | 0.438979787 | 5.15E-25 | postive |
| ISCU | SNHG20 | 0.430299685 | 5.35E-24 | postive |
| ZNF419 | SNHG20 | 0.412322042 | 5.52E-22 | postive |
| TUBE1 | SNHG20 | 0.484829764 | 6.75E-31 | postive |
| ELAVL1 | SNHG20 | 0.491350292 | 8.28E-32 | postive |
| STMN1 | SNHG20 | 0.447952329 | 4.26E-26 | postive |
| GABPB1 | SNHG20 | 0.40677977 | 2.18E-21 | postive |
| NOX1 | SNHG20 | 0.413351698 | 4.27E-22 | postive |
| PHKG2 | SNHG20 | 0.519839972 | 5.00E-36 | postive |
| ATG4D | SNHG20 | 0.430947433 | 4.50E-24 | postive |
| PEBP1 | SNHG20 | 0.46934503 | 8.25E-29 | postive |
| MYB | SNHG20 | 0.414978149 | 2.83E-22 | postive |
| TAZ | SNHG20 | 0.491014647 | 9.24E-32 | postive |
| KLHL24 | AC013652.1 | 0.448651265 | 3.50E-26 | postive |
| HELLS | AC127502.2 | 0.46140136 | 8.83E-28 | postive |
| HELLS | AL031673.1 | 0.505799013 | 6.73E-34 | postive |
| STMN1 | AL031673.1 | 0.514729747 | 3.06E-35 | postive |
| NOX1 | AL031673.1 | 0.548888274 | 9.23E-41 | postive |
| PHKG2 | AL031673.1 | 0.468932778 | 9.34E-29 | postive |
| TAZ | AL031673.1 | 0.50616145 | 5.95E-34 | postive |
| GPX4 | AC090425.2 | 0.538959976 | 4.32E-39 | postive |
| ISCU | AC090425.2 | 0.484183807 | 8.29E-31 | postive |
| SLC2A8 | AC090425.2 | 0.448332141 | 3.83E-26 | postive |
| ELAVL1 | AC090425.2 | 0.414044056 | 3.58E-22 | postive |
| STMN1 | AC090425.2 | 0.495467576 | 2.15E-32 | postive |
| NOX1 | AC090425.2 | 0.479530092 | 3.59E-30 | postive |
| PHKG2 | AC090425.2 | 0.532116123 | 5.68E-38 | postive |
| ATG4D | AC090425.2 | 0.489615931 | 1.45E-31 | postive |
| CDKN2A | AC090425.2 | 0.401399832 | 8.08E-21 | postive |
| SOCS1 | AC090425.2 | 0.413225097 | 4.40E-22 | postive |
| EGLN2 | AC090425.2 | 0.436982957 | 8.87E-25 | postive |
| TAZ | AC090425.2 | 0.413097562 | 4.55E-22 | postive |
| PANX1 | AC090425.2 | -0.413187684 | 4.44E-22 | negative |
| HIC1 | NKILA | 0.420454579 | 7.01E-23 | postive |
| CHAC1 | AC005392.2 | 0.403674531 | 4.66E-21 | postive |
| DUOX1 | AC005392.2 | 0.437304007 | 8.13E-25 | postive |
| ALOX12B | AC005392.2 | 0.462958249 | 5.58E-28 | postive |
| HELLS | ADNP-AS1 | 0.428730202 | 8.10E-24 | postive |
| ELAVL1 | ADNP-AS1 | 0.400386493 | 1.03E-20 | postive |
| STMN1 | ADNP-AS1 | 0.437934595 | 6.85E-25 | postive |
| NOX1 | ADNP-AS1 | 0.431430851 | 3.96E-24 | postive |
| TAZ | ADNP-AS1 | 0.40757004 | 1.80E-21 | postive |
| NFS1 | AL139289.1 | 0.407532475 | 1.81E-21 | postive |
| RPL8 | AL139289.1 | 0.436029838 | 1.15E-24 | postive |
| TAZ | AL139289.1 | 0.44409982 | 1.25E-25 | postive |
| HELLS | AC139256.3 | 0.411375308 | 6.99E-22 | postive |
| BRD4 | AC139256.3 | 0.4258134 | 1.75E-23 | postive |
| TUBE1 | AC139256.3 | 0.542138365 | 1.28E-39 | postive |
| ELAVL1 | AC139256.3 | 0.468964817 | 9.25E-29 | postive |
| GABPB1 | AC139256.3 | 0.442337783 | 2.04E-25 | postive |
| LPIN1 | AC139256.3 | 0.419234837 | 9.59E-23 | postive |
| MIOX | AC128709.1 | 0.465297329 | 2.78E-28 | postive |
| MIOX | AC090515.5 | 0.452526709 | 1.16E-26 | postive |
| SLC7A11 | MSC-AS1 | 0.420786198 | 6.44E-23 | postive |
| FTH1 | MSC-AS1 | 0.428617771 | 8.35E-24 | postive |
| NFE2L2 | AC019080.1 | 0.747253147 | 1.31E-90 | postive |
| KLHL24 | AC019080.1 | 0.473903157 | 2.06E-29 | postive |
| KEAP1 | AC019080.1 | 0.403338859 | 5.06E-21 | postive |
| ATG4D | AC019080.1 | 0.403586002 | 4.76E-21 | postive |
| IDH1 | AC019080.1 | 0.416301371 | 2.03E-22 | postive |
| FH | AL162258.2 | 0.452984703 | 1.02E-26 | postive |
| TAZ | AL162258.2 | 0.412798579 | 4.90E-22 | postive |
| CAV1 | AC099850.4 | 0.465200461 | 2.86E-28 | postive |
| EIF2AK4 | AC099850.4 | 0.454718002 | 6.19E-27 | postive |
| RRM2 | AC099850.4 | 0.483156335 | 1.15E-30 | postive |
| AURKA | AC099850.4 | 0.559864429 | 1.13E-42 | postive |
| NRAS | AC099850.4 | 0.478909015 | 4.36E-30 | postive |
| PHKG2 | AC106820.3 | 0.467396051 | 1.48E-28 | postive |
| MAP1LC3A | AC106820.3 | 0.434468346 | 1.75E-24 | postive |
| MIOX | AC068473.3 | 0.475971758 | 1.09E-29 | postive |
| FANCD2 | NBR2 | 0.423193998 | 3.46E-23 | postive |
| HELLS | NBR2 | 0.441709026 | 2.43E-25 | postive |
| ISCU | NBR2 | 0.412344732 | 5.49E-22 | postive |
| KLHL24 | NBR2 | 0.419568497 | 8.81E-23 | postive |
| TUBE1 | NBR2 | 0.437318225 | 8.10E-25 | postive |
| ELAVL1 | NBR2 | 0.442774284 | 1.81E-25 | postive |
| GLS2 | NBR2 | 0.433620055 | 2.20E-24 | postive |
| KEAP1 | NBR2 | 0.424573728 | 2.41E-23 | postive |
| ATG4D | NBR2 | 0.455931491 | 4.36E-27 | postive |
| YY1AP1 | NBR2 | 0.408549022 | 1.41E-21 | postive |
| PHKG2 | AC007938.3 | 0.471422335 | 4.39E-29 | postive |
| ISCU | AL353622.1 | 0.442898401 | 1.75E-25 | postive |
| PHKG2 | AL353622.1 | 0.455506992 | 4.93E-27 | postive |
| MYB | AL353622.1 | 0.411126875 | 7.44E-22 | postive |
| TAZ | AL353622.1 | 0.439268726 | 4.75E-25 | postive |
| TF | AC008406.3 | 0.461032747 | 9.85E-28 | postive |
| STMN1 | AC009061.2 | 0.406043404 | 2.61E-21 | postive |
| PHKG2 | AC009061.2 | 0.493916029 | 3.58E-32 | postive |
| GABARAPL2 | AC009061.2 | 0.401452468 | 7.98E-21 | postive |
| PEBP1 | AC009061.2 | 0.464829432 | 3.20E-28 | postive |
| TAZ | AC009061.2 | 0.444340315 | 1.17E-25 | postive |
| ENPP2 | MAGI2-AS3 | 0.503394057 | 1.52E-33 | postive |
| HIC1 | MAGI2-AS3 | 0.646209685 | 1.47E-60 | postive |
| NOX4 | MAGI2-AS3 | 0.65156077 | 7.29E-62 | postive |
| ZEB1 | MAGI2-AS3 | 0.760385995 | 1.38E-95 | postive |
| TLR4 | MAGI2-AS3 | 0.424270362 | 2.61E-23 | postive |
| TUBE1 | AC007566.1 | 0.400973786 | 8.96E-21 | postive |
| TUBE1 | AL031670.1 | 0.46200102 | 7.40E-28 | postive |
| ACVR1B | AL031670.1 | 0.444130667 | 1.24E-25 | postive |
| RPL8 | LINC02878 | 0.432506068 | 2.97E-24 | postive |
| PHKG2 | LINC02878 | 0.468520171 | 1.06E-28 | postive |
| EGLN2 | LINC02878 | 0.480134001 | 2.97E-30 | postive |
| TAZ | LINC02878 | 0.427135673 | 1.23E-23 | postive |
| FANCD2 | ATP1A1-AS1 | 0.403968239 | 4.34E-21 | postive |
| HELLS | ATP1A1-AS1 | 0.470196654 | 6.37E-29 | postive |
| STMN1 | ATP1A1-AS1 | 0.446217145 | 6.94E-26 | postive |
| GABPB1 | ATP1A1-AS1 | 0.406238933 | 2.49E-21 | postive |
| GLS2 | ATP1A1-AS1 | 0.437214838 | 8.33E-25 | postive |
| ULK2 | ATP1A1-AS1 | 0.402540872 | 6.13E-21 | postive |
| MAPK8 | ATP1A1-AS1 | 0.410117688 | 9.57E-22 | postive |
| ATF4 | AL359921.2 | 0.401347434 | 8.19E-21 | postive |
| TUBE1 | AL359921.2 | 0.444292358 | 1.19E-25 | postive |
| ELAVL1 | AL359921.2 | 0.414372173 | 3.30E-22 | postive |
| STMN1 | AL359921.2 | 0.418255659 | 1.23E-22 | postive |
| NOX1 | AL359921.2 | 0.423172024 | 3.48E-23 | postive |
| PHKG2 | AL359921.2 | 0.463595163 | 4.62E-28 | postive |
| TAZ | AL359921.2 | 0.432280369 | 3.15E-24 | postive |
| GPX4 | AL163051.1 | 0.412799196 | 4.90E-22 | postive |
| RPL8 | AL163051.1 | 0.441734396 | 2.41E-25 | postive |
| SLC2A8 | AL163051.1 | 0.429793365 | 6.12E-24 | postive |
| PHKG2 | AL163051.1 | 0.551791299 | 2.93E-41 | postive |
| EGLN2 | AL163051.1 | 0.530813231 | 9.21E-38 | postive |
| TAZ | AL163051.1 | 0.455669763 | 4.71E-27 | postive |
| LONP1 | AL163051.1 | 0.406257466 | 2.48E-21 | postive |
| SETD1B | AL109614.1 | 0.40043392 | 1.02E-20 | postive |
| SP1 | AL109614.1 | 0.431187717 | 4.22E-24 | postive |
| ACVR1B | AL109614.1 | 0.470466324 | 5.87E-29 | postive |
| HELLS | AC007485.1 | 0.440231396 | 3.65E-25 | postive |
| STMN1 | AC007485.1 | 0.43347402 | 2.29E-24 | postive |
| PHKG2 | AC106782.5 | 0.555420998 | 6.85E-42 | postive |
| TAZ | AC106782.5 | 0.447645571 | 4.64E-26 | postive |
| PHKG2 | AC005911.1 | 0.448838503 | 3.32E-26 | postive |
| TAZ | AC005911.1 | 0.44042412 | 3.46E-25 | postive |
| ATF3 | AL021578.1 | 0.412543391 | 5.22E-22 | postive |
| ATF4 | AC010969.2 | 0.406284229 | 2.46E-21 | postive |
| NOX1 | AC010969.2 | 0.481815073 | 1.75E-30 | postive |
| HRAS | AC010969.2 | 0.406511677 | 2.33E-21 | postive |
| PHKG2 | AC010969.2 | 0.540302007 | 2.59E-39 | postive |
| EGLN2 | AC010969.2 | 0.52192299 | 2.37E-36 | postive |
| TAZ | AC010969.2 | 0.42989492 | 5.95E-24 | postive |
| HELLS | ACAP2-IT1 | 0.474188624 | 1.88E-29 | postive |
| GCH1 | ACAP2-IT1 | 0.405941253 | 2.68E-21 | postive |
| NOS2 | ACAP2-IT1 | 0.429123503 | 7.30E-24 | postive |
| KLHL24 | ACAP2-IT1 | 0.535297436 | 1.73E-38 | postive |
| TUBE1 | ACAP2-IT1 | 0.44533833 | 8.87E-26 | postive |
| SETD1B | ACAP2-IT1 | 0.406592902 | 2.28E-21 | postive |
| SP1 | ACAP2-IT1 | 0.484085065 | 8.55E-31 | postive |
| PIK3CA | ACAP2-IT1 | 0.450230318 | 2.24E-26 | postive |
| GLS2 | ACAP2-IT1 | 0.487420156 | 2.95E-31 | postive |
| SNX4 | ACAP2-IT1 | 0.427714718 | 1.06E-23 | postive |
| ACVR1B | ACAP2-IT1 | 0.4148264 | 2.94E-22 | postive |
| YY1AP1 | ACAP2-IT1 | 0.432080451 | 3.33E-24 | postive |
| SIRT1 | ACAP2-IT1 | 0.419100436 | 9.93E-23 | postive |
| FANCD2 | THUMPD3-AS1 | 0.603969818 | 3.98E-51 | postive |
| ATF4 | THUMPD3-AS1 | 0.440986298 | 2.97E-25 | postive |
| HELLS | THUMPD3-AS1 | 0.478942604 | 4.32E-30 | postive |
| ISCU | THUMPD3-AS1 | 0.439382907 | 4.61E-25 | postive |
| TUBE1 | THUMPD3-AS1 | 0.400553365 | 9.91E-21 | postive |
| HMGB1 | THUMPD3-AS1 | 0.40947433 | 1.12E-21 | postive |
| STMN1 | THUMPD3-AS1 | 0.534135728 | 2.67E-38 | postive |
| NOX1 | THUMPD3-AS1 | 0.416466588 | 1.94E-22 | postive |
| PHKG2 | THUMPD3-AS1 | 0.414780266 | 2.98E-22 | postive |
| PEBP1 | THUMPD3-AS1 | 0.419326802 | 9.37E-23 | postive |
| TAZ | THUMPD3-AS1 | 0.455890046 | 4.42E-27 | postive |
| GPX4 | ZNF687-AS1 | 0.421703418 | 5.08E-23 | postive |
| PHKG2 | ZNF687-AS1 | 0.443041153 | 1.68E-25 | postive |
| GPX4 | AL109936.3 | 0.428635376 | 8.31E-24 | postive |
| HELLS | AL109936.3 | 0.436430531 | 1.03E-24 | postive |
| ISCU | AL109936.3 | 0.529282237 | 1.62E-37 | postive |
| STMN1 | AL109936.3 | 0.530487087 | 1.04E-37 | postive |
| NOX1 | AL109936.3 | 0.578755748 | 3.88E-46 | postive |
| GOT1 | AL109936.3 | 0.492389674 | 5.90E-32 | postive |
| KEAP1 | AL109936.3 | 0.406793403 | 2.17E-21 | postive |
| PHKG2 | AL109936.3 | 0.495894934 | 1.87E-32 | postive |
| ATG3 | AL109936.3 | 0.448893156 | 3.27E-26 | postive |
| ATG4D | AL109936.3 | 0.494593166 | 2.87E-32 | postive |
| CDKN2A | AL109936.3 | 0.583686052 | 4.44E-47 | postive |
| TAZ | AL109936.3 | 0.411811841 | 6.27E-22 | postive |
| MAPK9 | AC008393.1 | 0.405194156 | 3.22E-21 | postive |
| OTUB1 | PPP1R14B-AS1 | 0.475267093 | 1.35E-29 | postive |
| AGPAT3 | PPP1R14B-AS1 | -0.431763038 | 3.62E-24 | negative |
| SETD1B | PPP1R14B-AS1 | -0.401824695 | 7.30E-21 | negative |
| SP1 | PPP1R14B-AS1 | -0.433713618 | 2.15E-24 | negative |
| MAPK9 | PPP1R14B-AS1 | -0.409017955 | 1.26E-21 | negative |
| SIRT1 | PPP1R14B-AS1 | -0.40120254 | 8.48E-21 | negative |
| FANCD2 | AC107027.3 | 0.405664646 | 2.87E-21 | postive |
| TP53 | AC107027.3 | 0.416522799 | 1.92E-22 | postive |
| HELLS | AC107027.3 | 0.447503644 | 4.83E-26 | postive |
| AGPAT3 | AC107027.3 | 0.438041812 | 6.65E-25 | postive |
| ELAVL1 | AC107027.3 | 0.462056626 | 7.28E-28 | postive |
| SP1 | AC107027.3 | 0.516689241 | 1.53E-35 | postive |
| CS | AC107027.3 | 0.410661537 | 8.36E-22 | postive |
| GLS2 | AC107027.3 | 0.444033261 | 1.28E-25 | postive |
| ATG3 | AC107027.3 | 0.509480244 | 1.90E-34 | postive |
| SNX4 | AC107027.3 | 0.5399491 | 2.96E-39 | postive |
| MAPK8 | AC107027.3 | 0.422741632 | 3.89E-23 | postive |
| YY1AP1 | AC107027.3 | 0.407058672 | 2.04E-21 | postive |
| GPX4 | AC074032.1 | 0.505305344 | 7.96E-34 | postive |
| CISD1 | AC074032.1 | 0.453898393 | 7.84E-27 | postive |
| ISCU | AC074032.1 | 0.49148383 | 7.93E-32 | postive |
| NOX1 | AC074032.1 | 0.495798684 | 1.93E-32 | postive |
| PHKG2 | AC074032.1 | 0.506407617 | 5.47E-34 | postive |
| EGLN2 | AC074032.1 | 0.463788426 | 4.36E-28 | postive |
| TAZ | AC074032.1 | 0.424053523 | 2.76E-23 | postive |
| KLHL24 | ARHGAP31-AS1 | 0.507255602 | 4.09E-34 | postive |
| GABARAPL1 | ARHGAP31-AS1 | 0.43411479 | 1.93E-24 | postive |
| SNX4 | ARHGAP31-AS1 | 0.407355334 | 1.89E-21 | postive |
| HELLS | AC011481.1 | 0.412591884 | 5.16E-22 | postive |
| ISCU | AC011481.1 | 0.436251504 | 1.08E-24 | postive |
| STMN1 | AC011481.1 | 0.42138556 | 5.52E-23 | postive |
| PHKG2 | AC011481.1 | 0.479215008 | 3.97E-30 | postive |
| MYB | AC011481.1 | 0.447745616 | 4.52E-26 | postive |
| TAZ | AC011481.1 | 0.461392419 | 8.86E-28 | postive |
| TUBE1 | FMR1-IT1 | 0.405955977 | 2.67E-21 | postive |
| SETD1B | FMR1-IT1 | 0.400128659 | 1.10E-20 | postive |
| SP1 | FMR1-IT1 | 0.433302492 | 2.40E-24 | postive |
| ACVR1B | FMR1-IT1 | 0.430821426 | 4.65E-24 | postive |
| TAZ | LINC01144 | 0.421424028 | 5.46E-23 | postive |
| IFNG | LINC02446 | 0.766722785 | 4.17E-98 | postive |
| GPX4 | AC016876.2 | 0.556696785 | 4.09E-42 | postive |
| CISD1 | AC016876.2 | 0.439571414 | 4.38E-25 | postive |
| FANCD2 | AC016876.2 | 0.438684567 | 5.58E-25 | postive |
| TP53 | AC016876.2 | 0.417568652 | 1.47E-22 | postive |
| HELLS | AC016876.2 | 0.411952719 | 6.06E-22 | postive |
| ISCU | AC016876.2 | 0.448707173 | 3.44E-26 | postive |
| STMN1 | AC016876.2 | 0.568754046 | 2.82E-44 | postive |
| NOX1 | AC016876.2 | 0.459353289 | 1.61E-27 | postive |
| GLS2 | AC016876.2 | 0.438720397 | 5.52E-25 | postive |
| PHKG2 | AC016876.2 | 0.43546136 | 1.34E-24 | postive |
| MAP1LC3A | AC016876.2 | 0.468790875 | 9.75E-29 | postive |
| CDKN2A | AC016876.2 | 0.560933655 | 7.29E-43 | postive |
| TAZ | AC016876.2 | 0.429227767 | 7.10E-24 | postive |
| GPX4 | AF131215.6 | 0.459955793 | 1.35E-27 | postive |
| FANCD2 | AF131215.6 | 0.471914389 | 3.78E-29 | postive |
| TP53 | AF131215.6 | 0.408373221 | 1.47E-21 | postive |
| HELLS | AF131215.6 | 0.486956781 | 3.42E-31 | postive |
| ISCU | AF131215.6 | 0.563613274 | 2.42E-43 | postive |
| STMN1 | AF131215.6 | 0.463907078 | 4.21E-28 | postive |
| NOX1 | AF131215.6 | 0.462986926 | 5.53E-28 | postive |
| ATG4D | AF131215.6 | 0.400223358 | 1.07E-20 | postive |
| CDKN2A | AF131215.6 | 0.473903487 | 2.06E-29 | postive |
| SOCS1 | AF131215.6 | 0.519243074 | 6.19E-36 | postive |
| MYB | AF131215.6 | 0.513996647 | 3.95E-35 | postive |
| HELLS | AC124283.2 | 0.439781403 | 4.13E-25 | postive |
| GLS2 | AC124283.2 | 0.420003061 | 7.88E-23 | postive |
| KLHL24 | A2M-AS1 | 0.459164097 | 1.70E-27 | postive |
| STMN1 | AC095057.3 | 0.522716364 | 1.78E-36 | postive |
| NOX1 | AC095057.3 | 0.428012389 | 9.80E-24 | postive |
| PHKG2 | AC095057.3 | 0.462541376 | 6.31E-28 | postive |
| TAZ | AC095057.3 | 0.473911863 | 2.05E-29 | postive |
| ISCU | AL162586.1 | 0.433250054 | 2.43E-24 | postive |
| GLS2 | AL162586.1 | 0.404856522 | 3.49E-21 | postive |
| GPX4 | ZFAS1 | 0.410725082 | 8.23E-22 | postive |
| ATF4 | ZFAS1 | 0.459845501 | 1.40E-27 | postive |
| RPL8 | ZFAS1 | 0.673319019 | 1.91E-67 | postive |
| PHKG2 | ZFAS1 | 0.402960761 | 5.54E-21 | postive |
| EGLN2 | ZFAS1 | 0.433219987 | 2.45E-24 | postive |
| ALOX12B | LINC02437 | 0.420647047 | 6.67E-23 | postive |
| HRAS | AC104031.1 | 0.438623363 | 5.67E-25 | postive |
| ISCU | LINC00944 | 0.494980462 | 2.53E-32 | postive |
| SOCS1 | LINC00944 | 0.409259436 | 1.18E-21 | postive |
| GPX4 | AC109322.1 | 0.449628697 | 2.65E-26 | postive |
| RPL8 | AC109322.1 | 0.528918324 | 1.86E-37 | postive |
| SLC2A8 | AC109322.1 | 0.479482035 | 3.65E-30 | postive |
| PHKG2 | AC109322.1 | 0.520235619 | 4.34E-36 | postive |
| PEBP1 | AC109322.1 | 0.447454081 | 4.90E-26 | postive |
| EGLN2 | AC109322.1 | 0.607217314 | 8.40E-52 | postive |
| TAZ | AC109322.1 | 0.407369999 | 1.89E-21 | postive |
| LONP1 | AC109322.1 | 0.416355854 | 2.00E-22 | postive |
| FANCD2 | AC002116.2 | 0.497203725 | 1.21E-32 | postive |
| HELLS | AC002116.2 | 0.47597446 | 1.09E-29 | postive |
| HMGB1 | AC002116.2 | 0.432745546 | 2.78E-24 | postive |
| STMN1 | AC002116.2 | 0.513778083 | 4.27E-35 | postive |
| NOX1 | AC002116.2 | 0.436738981 | 9.48E-25 | postive |
| PHKG2 | AC002116.2 | 0.418128153 | 1.27E-22 | postive |
| TAZ | AC002116.2 | 0.472313551 | 3.34E-29 | postive |
| AGPAT3 | MIR193BHG | -0.400149651 | 1.09E-20 | negative |
| ELAVL1 | MIR193BHG | -0.402067412 | 6.88E-21 | negative |
| SP1 | MIR193BHG | -0.437415335 | 7.89E-25 | negative |
| HELLS | AC011815.1 | 0.440290831 | 3.59E-25 | postive |
| GLS2 | AC011815.1 | 0.401362277 | 8.16E-21 | postive |
| ISCU | AC007541.1 | 0.428417838 | 8.80E-24 | postive |
| STMN1 | AC007541.1 | 0.506445738 | 5.40E-34 | postive |
| ATM | AP003392.1 | 0.503892273 | 1.29E-33 | postive |
| KLHL24 | AC046134.2 | 0.429826151 | 6.06E-24 | postive |
| SNX4 | AC046134.2 | 0.401691237 | 7.53E-21 | postive |
| NFE2L2 | L3MBTL2-AS1 | 0.446853894 | 5.80E-26 | postive |
| PSAT1 | L3MBTL2-AS1 | 0.489349261 | 1.58E-31 | postive |
| GLS2 | L3MBTL2-AS1 | 0.402771417 | 5.80E-21 | postive |
| FANCD2 | PPP1R26-AS1 | 0.414388514 | 3.29E-22 | postive |
| HELLS | PPP1R26-AS1 | 0.554152376 | 1.14E-41 | postive |
| ISCU | PPP1R26-AS1 | 0.45221214 | 1.27E-26 | postive |
| STMN1 | PPP1R26-AS1 | 0.406992824 | 2.07E-21 | postive |
| GLS2 | PPP1R26-AS1 | 0.427031155 | 1.27E-23 | postive |
| SOCS1 | PPP1R26-AS1 | 0.437530379 | 7.64E-25 | postive |
| MYB | PPP1R26-AS1 | 0.497651447 | 1.04E-32 | postive |
| MAPK8 | PPP1R26-AS1 | 0.417391576 | 1.54E-22 | postive |
| ACSF2 | AC017104.1 | 0.450545577 | 2.04E-26 | postive |
| FANCD2 | Z94721.1 | 0.430023039 | 5.75E-24 | postive |
| HELLS | Z94721.1 | 0.564758602 | 1.50E-43 | postive |
| SP1 | Z94721.1 | 0.436975314 | 8.89E-25 | postive |
| MAPK8 | Z94721.1 | 0.44547729 | 8.53E-26 | postive |
| ACVR1B | Z94721.1 | 0.405709912 | 2.84E-21 | postive |
| BID | TRPM2-AS | 0.426728832 | 1.37E-23 | postive |
| LPCAT3 | PLBD1-AS1 | 0.41649069 | 1.93E-22 | postive |
| PLIN4 | AL592424.1 | 0.795078276 | 1.94E-110 | postive |
| WIPI1 | AL592424.1 | 0.44185337 | 2.34E-25 | postive |
| ZEB1 | AL592424.1 | 0.415639279 | 2.40E-22 | postive |
| PRKAA2 | AL592424.1 | 0.749764953 | 1.54E-91 | postive |
| TMBIM4 | PRANCR | 0.415644698 | 2.39E-22 | postive |
| PHKG2 | PRANCR | 0.462474837 | 6.43E-28 | postive |
| TAZ | PRANCR | 0.422514188 | 4.12E-23 | postive |
| GPX4 | AC009065.5 | 0.431333197 | 4.06E-24 | postive |
| CHMP6 | AC009065.5 | 0.407290014 | 1.92E-21 | postive |
| SLC2A8 | AC009065.5 | 0.464851063 | 3.18E-28 | postive |
| NOX1 | AC009065.5 | 0.49377939 | 3.75E-32 | postive |
| PHKG2 | AC009065.5 | 0.733331042 | 1.17E-85 | postive |
| MAP1LC3A | AC009065.5 | 0.447874055 | 4.35E-26 | postive |
| ANO6 | AC009065.5 | -0.424717007 | 2.33E-23 | negative |
| EGLN2 | AC009065.5 | 0.572683514 | 5.33E-45 | postive |
| TAZ | AC009065.5 | 0.615718754 | 1.32E-53 | postive |
| GCLC | AC019080.4 | 0.453226035 | 9.51E-27 | postive |
| NFE2L2 | AC019080.4 | 0.512890566 | 5.82E-35 | postive |
| MAFG | AC019080.4 | 0.423218099 | 3.43E-23 | postive |
| ABCC1 | AC019080.4 | 0.456209869 | 4.02E-27 | postive |
| PHKG2 | AC083880.1 | 0.482492665 | 1.42E-30 | postive |
| TAZ | AC083880.1 | 0.448748402 | 3.40E-26 | postive |
| RPL8 | SLC12A9-AS1 | 0.41047103 | 8.76E-22 | postive |
| EGLN2 | PRR34-AS1 | 0.477423718 | 6.93E-30 | postive |
| FANCD2 | C5orf66 | 0.410058051 | 9.71E-22 | postive |
| HELLS | C5orf66 | 0.465408305 | 2.69E-28 | postive |
| STAT3 | C5orf66 | 0.450482843 | 2.08E-26 | postive |
| GCH1 | C5orf66 | 0.46019013 | 1.26E-27 | postive |
| SP1 | C5orf66 | 0.511333399 | 1.00E-34 | postive |
| GLS2 | C5orf66 | 0.529715018 | 1.38E-37 | postive |
| GOT1 | C5orf66 | 0.409343614 | 1.16E-21 | postive |
| ATG4D | C5orf66 | 0.423619531 | 3.09E-23 | postive |
| MAPK8 | C5orf66 | 0.474158819 | 1.90E-29 | postive |
| ACVR1B | C5orf66 | 0.401323334 | 8.23E-21 | postive |
| PHKG2 | DGUOK-AS1 | 0.438700842 | 5.55E-25 | postive |
| TAZ | DGUOK-AS1 | 0.405504168 | 2.98E-21 | postive |
| ISCU | AC068620.2 | 0.42550974 | 1.89E-23 | postive |
| ZNF419 | AC068620.2 | 0.424307002 | 2.59E-23 | postive |
| STMN1 | AC068620.2 | 0.46708578 | 1.63E-28 | postive |
| NOX1 | AC068620.2 | 0.524111044 | 1.07E-36 | postive |
| PHKG2 | AC068620.2 | 0.528045124 | 2.56E-37 | postive |
| TAZ | AC068620.2 | 0.548568158 | 1.05E-40 | postive |
| ENPP2 | AC093010.2 | 0.444104753 | 1.25E-25 | postive |
| ALOX5 | AC093010.2 | 0.410255117 | 9.25E-22 | postive |
| HIC1 | AC093010.2 | 0.572252994 | 6.41E-45 | postive |
| NOX4 | AC093010.2 | 0.508551233 | 2.62E-34 | postive |
| WIPI1 | AC093010.2 | 0.463029175 | 5.46E-28 | postive |
| ZEB1 | AC093010.2 | 0.69747315 | 3.12E-74 | postive |
| PRKAA2 | AC093010.2 | 0.45588627 | 4.42E-27 | postive |
| ISCU | AC135050.6 | 0.425534747 | 1.88E-23 | postive |
| PHKG2 | AC135050.6 | 0.598878252 | 4.40E-50 | postive |
| MAP1LC3A | AC135050.6 | 0.406979584 | 2.08E-21 | postive |
| TAZ | AC135050.6 | 0.454132838 | 7.33E-27 | postive |
| PHKG2 | ST7-AS1 | 0.473732649 | 2.17E-29 | postive |
| SLC2A8 | AL023803.2 | 0.476615098 | 8.91E-30 | postive |
| STMN1 | AL023803.2 | 0.442611268 | 1.89E-25 | postive |
| NOX1 | AL023803.2 | 0.449177135 | 3.01E-26 | postive |
| PHKG2 | AL023803.2 | 0.417108707 | 1.65E-22 | postive |
| PEBP1 | AL023803.2 | 0.405014201 | 3.36E-21 | postive |
| SOCS1 | AL023803.2 | 0.452916101 | 1.04E-26 | postive |
| EGLN2 | AL023803.2 | 0.40803873 | 1.60E-21 | postive |
| TAZ | RNASEH1-AS1 | 0.41017319 | 9.44E-22 | postive |
| GPX4 | AC138696.2 | 0.473713903 | 2.18E-29 | postive |
| CHMP6 | AC138696.2 | 0.501050478 | 3.36E-33 | postive |
| RPL8 | AC138696.2 | 0.564404409 | 1.74E-43 | postive |
| SLC2A8 | AC138696.2 | 0.456422603 | 3.78E-27 | postive |
| PHKG2 | AC138696.2 | 0.6337243 | 1.28E-57 | postive |
| EGLN2 | AC138696.2 | 0.700932926 | 2.93E-75 | postive |
| TAZ | AC138696.2 | 0.541554027 | 1.60E-39 | postive |
| LONP1 | AC138696.2 | 0.41250496 | 5.27E-22 | postive |
| GPX2 | LINC02870 | 0.404949877 | 3.42E-21 | postive |
| BID | LINC02870 | 0.449516329 | 2.74E-26 | postive |
| MIOX | LINC02870 | 0.472836761 | 2.85E-29 | postive |
| ALOX15B | AC010255.2 | 0.471666026 | 4.08E-29 | postive |
| HELLS | AC004148.1 | 0.426397664 | 1.50E-23 | postive |
| ISCU | AC004148.1 | 0.402077165 | 6.86E-21 | postive |
| TUBE1 | AC004148.1 | 0.526446434 | 4.59E-37 | postive |
| STMN1 | AC004148.1 | 0.430023573 | 5.75E-24 | postive |
| NOX1 | AC004148.1 | 0.417571062 | 1.47E-22 | postive |
| PHKG2 | AC004148.1 | 0.424159093 | 2.69E-23 | postive |
| MYB | AC004148.1 | 0.43962482 | 4.31E-25 | postive |
| TAZ | AC004148.1 | 0.427616407 | 1.09E-23 | postive |
| PLIN4 | LINC01405 | 0.628801715 | 1.70E-56 | postive |
| CDO1 | LINC01405 | 0.429941122 | 5.88E-24 | postive |
| PRKAA2 | LINC01405 | 0.469673198 | 7.47E-29 | postive |
| GPX4 | AC124016.3 | 0.525238298 | 7.14E-37 | postive |
| HELLS | AC124016.3 | 0.41097625 | 7.73E-22 | postive |
| ISCU | AC124016.3 | 0.558688486 | 1.82E-42 | postive |
| STMN1 | AC124016.3 | 0.532003795 | 5.92E-38 | postive |
| NOX1 | AC124016.3 | 0.650280166 | 1.50E-61 | postive |
| PHKG2 | AC124016.3 | 0.428958121 | 7.63E-24 | postive |
| ATG4D | AC124016.3 | 0.411063878 | 7.56E-22 | postive |
| MAP1LC3A | AC124016.3 | 0.466230325 | 2.10E-28 | postive |
| CDKN2A | AC124016.3 | 0.400621034 | 9.75E-21 | postive |
| SOCS1 | AC124016.3 | 0.483426632 | 1.05E-30 | postive |
| PSAT1 | TAF1A-AS1 | 0.405212028 | 3.20E-21 | postive |
| TAZ | AC027796.4 | 0.423337366 | 3.33E-23 | postive |
| SLC40A1 | AL450326.1 | 0.429104035 | 7.34E-24 | postive |
| MTOR | AL450326.1 | 0.419691867 | 8.53E-23 | postive |
| ENPP2 | AL450326.1 | 0.41031131 | 9.12E-22 | postive |
| KLHL24 | AL450326.1 | 0.401621881 | 7.66E-21 | postive |
| SETD1B | AL450326.1 | 0.450860799 | 1.87E-26 | postive |
| IREB2 | AL450326.1 | 0.428918569 | 7.71E-24 | postive |
| SP1 | AL450326.1 | 0.49526542 | 2.30E-32 | postive |
| NCOA4 | AL450326.1 | 0.420236414 | 7.42E-23 | postive |
| ZEB1 | AL450326.1 | 0.504089224 | 1.20E-33 | postive |
| PRKAA2 | AL450326.1 | 0.408843326 | 1.31E-21 | postive |
| ATM | AL450326.1 | 0.507344487 | 3.97E-34 | postive |
| SIRT1 | AL450326.1 | 0.453243006 | 9.47E-27 | postive |
| HELLS | MIF-AS1 | 0.416210644 | 2.07E-22 | postive |
| GLS2 | MIF-AS1 | 0.488043765 | 2.41E-31 | postive |
| ENPP2 | LINC00426 | 0.510502525 | 1.34E-34 | postive |
| ISCU | LINC00426 | 0.432468408 | 3.00E-24 | postive |
| HERPUD1 | LINC00426 | 0.40561297 | 2.90E-21 | postive |
| ALOX5 | LINC00426 | 0.523882451 | 1.17E-36 | postive |
| CYBB | LINC00426 | 0.503125218 | 1.67E-33 | postive |
| FLT3 | LINC00426 | 0.742810964 | 5.38E-89 | postive |
| IFNG | LINC00426 | 0.631939398 | 3.29E-57 | postive |
| NFE2L2 | SYNPR-AS1 | 0.49713886 | 1.24E-32 | postive |
| KLHL24 | SYNPR-AS1 | 0.588126656 | 6.11E-48 | postive |
| ELAVL1 | SYNPR-AS1 | 0.414404361 | 3.27E-22 | postive |
| GLS2 | SYNPR-AS1 | 0.480750334 | 2.45E-30 | postive |
| KEAP1 | SYNPR-AS1 | 0.421760865 | 5.01E-23 | postive |
| GABARAPL1 | SYNPR-AS1 | 0.414941138 | 2.86E-22 | postive |
| FANCD2 | AC010186.3 | 0.443925372 | 1.32E-25 | postive |
| TP53 | AC010186.3 | 0.444432136 | 1.14E-25 | postive |
| HELLS | AC010186.3 | 0.519922813 | 4.85E-36 | postive |
| ISCU | AC010186.3 | 0.429948979 | 5.87E-24 | postive |
| SP1 | AC010186.3 | 0.480199323 | 2.91E-30 | postive |
| GABPB1 | AC010186.3 | 0.413596797 | 4.01E-22 | postive |
| LPCAT3 | AC010186.3 | 0.42046216 | 7.00E-23 | postive |
| GLS2 | AC010186.3 | 0.459305714 | 1.63E-27 | postive |
| MAPK8 | AC010186.3 | 0.447798689 | 4.45E-26 | postive |
| PHKG2 | AC135048.3 | 0.52324939 | 1.47E-36 | postive |
| FANCD2 | AC008763.1 | 0.440701101 | 3.21E-25 | postive |
| HELLS | AC008763.1 | 0.524146195 | 1.06E-36 | postive |
| ISCU | AC008763.1 | 0.40561332 | 2.90E-21 | postive |
| ELAVL1 | AC008763.1 | 0.422136553 | 4.55E-23 | postive |
| STMN1 | AC008763.1 | 0.533075097 | 3.97E-38 | postive |
| NOX1 | AC008763.1 | 0.424396196 | 2.53E-23 | postive |
| GLS2 | AC008763.1 | 0.53899924 | 4.25E-39 | postive |
| ATG3 | AC008763.1 | 0.401454701 | 7.98E-21 | postive |
| ATG4D | AC008763.1 | 0.497796433 | 9.95E-33 | postive |
| TAZ | AC008763.1 | 0.410100518 | 9.61E-22 | postive |
| KLHL24 | AC131235.3 | 0.465105471 | 2.95E-28 | postive |
| PIK3CA | AC131235.3 | 0.430930397 | 4.52E-24 | postive |
| DUOX2 | LINC01269 | 0.463365369 | 4.94E-28 | postive |
| GPX4 | AC091982.3 | 0.412012003 | 5.97E-22 | postive |
| ISCU | AC091982.3 | 0.418824118 | 1.07E-22 | postive |
| STMN1 | AC091982.3 | 0.425308405 | 1.99E-23 | postive |
| GOT1 | AC091982.3 | 0.408185528 | 1.54E-21 | postive |
| PHKG2 | IBA57-DT | 0.446638377 | 6.16E-26 | postive |
| GPX4 | OSER1-DT | 0.640710097 | 3.01E-59 | postive |
| CISD1 | OSER1-DT | 0.470372419 | 6.04E-29 | postive |
| ISCU | OSER1-DT | 0.512408313 | 6.89E-35 | postive |
| ACSF2 | OSER1-DT | 0.401559517 | 7.78E-21 | postive |
| STMN1 | OSER1-DT | 0.586799019 | 1.11E-47 | postive |
| NOX1 | OSER1-DT | 0.498611172 | 7.59E-33 | postive |
| MAP1LC3A | OSER1-DT | 0.404055265 | 4.25E-21 | postive |
| TAZ | OSER1-DT | 0.400905011 | 9.11E-21 | postive |
| MTOR | LINC01128 | 0.402431331 | 6.30E-21 | postive |
| HELLS | AC008764.2 | 0.485197633 | 6.00E-31 | postive |
| BRD4 | AC008764.2 | 0.446951411 | 5.65E-26 | postive |
| KLHL24 | AC008764.2 | 0.410386589 | 8.95E-22 | postive |
| SETD1B | AC008764.2 | 0.526795942 | 4.04E-37 | postive |
| IREB2 | AC008764.2 | 0.427275101 | 1.19E-23 | postive |
| SP1 | AC008764.2 | 0.62449126 | 1.58E-55 | postive |
| CS | AC008764.2 | 0.413608664 | 4.00E-22 | postive |
| GLS2 | AC008764.2 | 0.47345771 | 2.36E-29 | postive |
| MAPK8 | AC008764.2 | 0.432995667 | 2.60E-24 | postive |
| ACVR1B | AC008764.2 | 0.487564326 | 2.82E-31 | postive |
| ATM | AC008764.2 | 0.552817703 | 1.94E-41 | postive |
| SIRT1 | AC008764.2 | 0.450784316 | 1.91E-26 | postive |
| PHKG2 | ASMTL-AS1 | 0.508732691 | 2.46E-34 | postive |
| TAZ | ASMTL-AS1 | 0.492336791 | 6.01E-32 | postive |
| HELLS | AC096992.2 | 0.54413823 | 5.90E-40 | postive |
| FADS2 | AC096992.2 | 0.400481655 | 1.01E-20 | postive |
| ISCU | AC096992.2 | 0.481166151 | 2.15E-30 | postive |
| ELAVL1 | AC096992.2 | 0.446782003 | 5.92E-26 | postive |
| STMN1 | AC096992.2 | 0.448121406 | 4.06E-26 | postive |
| NOX1 | AC096992.2 | 0.406809762 | 2.17E-21 | postive |
| GLS2 | AC096992.2 | 0.501706647 | 2.69E-33 | postive |
| GOT1 | AC096992.2 | 0.414826013 | 2.94E-22 | postive |
| ATG3 | AC096992.2 | 0.479225053 | 3.95E-30 | postive |
| ATG4D | AC096992.2 | 0.52831957 | 2.31E-37 | postive |
| SNX4 | AC096992.2 | 0.400419813 | 1.02E-20 | postive |
| MAPK8 | AC096992.2 | 0.419093354 | 9.95E-23 | postive |
| STMN1 | AC091153.3 | 0.442054433 | 2.21E-25 | postive |
| TAZ | AC091153.3 | 0.403340669 | 5.05E-21 | postive |
| AKR1C1 | AC124067.2 | 0.457468888 | 2.79E-27 | postive |
| AKR1C3 | AC124067.2 | 0.476070207 | 1.05E-29 | postive |
| GPX2 | AC124067.2 | 0.531529677 | 7.06E-38 | postive |
| BID | AC124067.2 | 0.429074548 | 7.40E-24 | postive |
| NFE2L2 | LINC00519 | 0.416489623 | 1.93E-22 | postive |
| GPX2 | LINC00519 | 0.462005477 | 7.39E-28 | postive |
| ISCU | AC026367.2 | 0.412272234 | 5.59E-22 | postive |
| ISCU | AC004918.1 | 0.407053058 | 2.04E-21 | postive |
| ATF4 | GAS5 | 0.537716958 | 6.92E-39 | postive |
| FH | GAS5 | 0.450636978 | 1.99E-26 | postive |
| PRDX6 | GAS5 | 0.46546993 | 2.64E-28 | postive |
| RPL8 | GAS5 | 0.5360064 | 1.32E-38 | postive |
| TAZ | GAS5 | 0.428289761 | 9.10E-24 | postive |
| YY1AP1 | AL139011.1 | 0.4067299 | 2.21E-21 | postive |
| HERPUD1 | AC012236.1 | 0.727926984 | 8.06E-84 | postive |
| FLT3 | AC012236.1 | 0.495577445 | 2.07E-32 | postive |
| TP53 | AC027097.2 | 0.410831924 | 8.01E-22 | postive |
| ENPP2 | AC027097.2 | 0.513996792 | 3.95E-35 | postive |
| ALOX5 | AC027097.2 | 0.412955 | 4.71E-22 | postive |
| CYBB | AC027097.2 | 0.401526187 | 7.84E-21 | postive |
| FLT3 | AC027097.2 | 0.501519806 | 2.87E-33 | postive |
| ATM | AC027097.2 | 0.42772606 | 1.06E-23 | postive |
| HELLS | AC010834.3 | 0.43403482 | 1.97E-24 | postive |
| MTOR | AC010834.3 | 0.445163349 | 9.32E-26 | postive |
| KLHL24 | AC010834.3 | 0.436265928 | 1.08E-24 | postive |
| ZFP69B | AC010834.3 | 0.406360003 | 2.42E-21 | postive |
| TUBE1 | AC010834.3 | 0.40367949 | 4.65E-21 | postive |
| SETD1B | AC010834.3 | 0.505621175 | 7.15E-34 | postive |
| IREB2 | AC010834.3 | 0.413662594 | 3.94E-22 | postive |
| SP1 | AC010834.3 | 0.590535983 | 2.05E-48 | postive |
| PIK3CA | AC010834.3 | 0.421033216 | 6.04E-23 | postive |
| GLS2 | AC010834.3 | 0.412276389 | 5.58E-22 | postive |
| MAPK8 | AC010834.3 | 0.465650767 | 2.50E-28 | postive |
| PRKAA1 | AC010834.3 | 0.420534731 | 6.87E-23 | postive |
| ACVR1B | AC010834.3 | 0.509224638 | 2.08E-34 | postive |
| ATM | AC010834.3 | 0.538323216 | 5.50E-39 | postive |
| YY1AP1 | AC010834.3 | 0.413691675 | 3.92E-22 | postive |
| SIRT1 | AC010834.3 | 0.550764978 | 4.40E-41 | postive |
| GPX4 | AC002398.1 | 0.42540855 | 1.94E-23 | postive |
| PHKG2 | AC002398.1 | 0.451860392 | 1.41E-26 | postive |
| EGLN2 | AC002398.1 | 0.522658978 | 1.82E-36 | postive |
| TP53 | ZNF667-AS1 | 0.4320257 | 3.37E-24 | postive |
| HELLS | ZNF667-AS1 | 0.448266984 | 3.90E-26 | postive |
| ISCU | ZNF667-AS1 | 0.493893532 | 3.61E-32 | postive |
| STMN1 | ZNF667-AS1 | 0.401572516 | 7.75E-21 | postive |
| GLS2 | ZNF667-AS1 | 0.48394901 | 8.93E-31 | postive |
| CDKN2A | ZNF667-AS1 | 0.408162495 | 1.55E-21 | postive |
| TUBE1 | AC004253.1 | 0.437498943 | 7.71E-25 | postive |
| TUBE1 | AC027763.2 | 0.413618233 | 3.99E-22 | postive |
| PRDX6 | AL355472.3 | 0.416286801 | 2.03E-22 | postive |
| TUBE1 | AL355472.3 | 0.431380068 | 4.01E-24 | postive |
| FANCD2 | AC073896.3 | 0.496782777 | 1.39E-32 | postive |
| TP53 | AC073896.3 | 0.404314801 | 3.99E-21 | postive |
| HELLS | AC073896.3 | 0.579920963 | 2.33E-46 | postive |
| FADS2 | AC073896.3 | 0.445237873 | 9.12E-26 | postive |
| ISCU | AC073896.3 | 0.464025169 | 4.06E-28 | postive |
| ELAVL1 | AC073896.3 | 0.444910461 | 1.00E-25 | postive |
| SP1 | AC073896.3 | 0.447911222 | 4.31E-26 | postive |
| GABPB1 | AC073896.3 | 0.405345338 | 3.10E-21 | postive |
| GLS2 | AC073896.3 | 0.483408872 | 1.06E-30 | postive |
| MYB | AC073896.3 | 0.546187703 | 2.66E-40 | postive |
| NOX1 | AC137630.3 | 0.409804238 | 1.03E-21 | postive |
| PHKG2 | AC137630.3 | 0.504812002 | 9.42E-34 | postive |
| EGLN2 | AC137630.3 | 0.445468532 | 8.55E-26 | postive |
| ATM | CTBP1-DT | 0.42426967 | 2.61E-23 | postive |
| GPX4 | H1FX-AS1 | 0.442193072 | 2.13E-25 | postive |
| ISCU | H1FX-AS1 | 0.40567001 | 2.86E-21 | postive |
| STMN1 | H1FX-AS1 | 0.480991698 | 2.27E-30 | postive |
| PHKG2 | H1FX-AS1 | 0.576336313 | 1.11E-45 | postive |
| MAP1LC3A | H1FX-AS1 | 0.553871368 | 1.28E-41 | postive |
| SOCS1 | H1FX-AS1 | 0.436075055 | 1.13E-24 | postive |
| EGLN2 | H1FX-AS1 | 0.417185258 | 1.62E-22 | postive |
| TAZ | H1FX-AS1 | 0.425493373 | 1.90E-23 | postive |
| MIOX | AL049870.3 | 0.447793424 | 4.45E-26 | postive |
| SETD1B | AC122129.1 | 0.4539043 | 7.83E-27 | postive |
| SP1 | AC122129.1 | 0.462961529 | 5.57E-28 | postive |
| ACVR1B | AC122129.1 | 0.415719945 | 2.35E-22 | postive |
| ATM | AC122129.1 | 0.403119092 | 5.33E-21 | postive |
| GPX4 | AC036176.1 | 0.558013082 | 2.40E-42 | postive |
| CISD1 | AC036176.1 | 0.48559372 | 5.29E-31 | postive |
| HELLS | AC036176.1 | 0.450911207 | 1.84E-26 | postive |
| ISCU | AC036176.1 | 0.58420388 | 3.53E-47 | postive |
| STMN1 | AC036176.1 | 0.477164485 | 7.51E-30 | postive |
| NOX1 | AC036176.1 | 0.471821989 | 3.89E-29 | postive |
| GLS2 | AC036176.1 | 0.413690303 | 3.92E-22 | postive |
| MAP1LC3A | AC036176.1 | 0.468633037 | 1.02E-28 | postive |
| CDKN2A | AC036176.1 | 0.495183529 | 2.36E-32 | postive |
| SOCS1 | AC036176.1 | 0.479600478 | 3.52E-30 | postive |
| HELLS | AP000442.1 | 0.415508217 | 2.48E-22 | postive |
| HELLS | AC079414.3 | 0.529053634 | 1.77E-37 | postive |
| ISCU | AC079414.3 | 0.433471004 | 2.29E-24 | postive |
| ELAVL1 | AC079414.3 | 0.401677627 | 7.56E-21 | postive |
| STMN1 | AC079414.3 | 0.549112643 | 8.45E-41 | postive |
| NOX1 | AC079414.3 | 0.560784504 | 7.75E-43 | postive |
| GLS2 | AC079414.3 | 0.424100047 | 2.73E-23 | postive |
| PHKG2 | AC079414.3 | 0.450389562 | 2.14E-26 | postive |
| PEBP1 | AC079414.3 | 0.450310149 | 2.19E-26 | postive |
| TAZ | AC079414.3 | 0.495936152 | 1.84E-32 | postive |
| KLHL24 | AL513550.1 | 0.467607402 | 1.39E-28 | postive |
| TUBE1 | AL513550.1 | 0.525802622 | 5.81E-37 | postive |
| GABPB1 | AL513550.1 | 0.401318076 | 8.24E-21 | postive |
| PLIN4 | MIR133A1HG | 0.832764089 | 3.14E-130 | postive |
| PRKAA2 | MIR133A1HG | 0.552149779 | 2.54E-41 | postive |
| SETD1B | PAXIP1-AS2 | 0.412225626 | 5.66E-22 | postive |
| SP1 | PAXIP1-AS2 | 0.415296487 | 2.61E-22 | postive |
| ATM | PAXIP1-AS2 | 0.563069672 | 3.02E-43 | postive |
| ATF4 | AC016876.3 | 0.450811434 | 1.90E-26 | postive |
| RPL8 | AC016876.3 | 0.535976875 | 1.34E-38 | postive |
| SLC2A8 | AC016876.3 | 0.423315621 | 3.35E-23 | postive |
| HRAS | AC016876.3 | 0.503173461 | 1.64E-33 | postive |
| PHKG2 | AC016876.3 | 0.635629679 | 4.65E-58 | postive |
| MAPK1 | AC016876.3 | -0.404698434 | 3.63E-21 | negative |
| ANO6 | AC016876.3 | -0.446839597 | 5.83E-26 | negative |
| EGLN2 | AC016876.3 | 0.574542528 | 2.41E-45 | postive |
| TAZ | AC016876.3 | 0.490973381 | 9.36E-32 | postive |
| STMN1 | TYMSOS | 0.48205331 | 1.63E-30 | postive |
| NOX1 | TYMSOS | 0.481488248 | 1.94E-30 | postive |
| PHKG2 | TYMSOS | 0.420456613 | 7.01E-23 | postive |
| TAZ | TYMSOS | 0.403792667 | 4.53E-21 | postive |
| STMN1 | AL139286.1 | 0.411049333 | 7.59E-22 | postive |
| NOX1 | AL139286.1 | 0.418691429 | 1.10E-22 | postive |
| PHKG2 | AL139286.1 | 0.445356899 | 8.82E-26 | postive |
| PEBP1 | AL139286.1 | 0.413155822 | 4.48E-22 | postive |
| TAZ | AL139286.1 | 0.412601977 | 5.15E-22 | postive |
| FANCD2 | PPP3CB-AS1 | 0.413731272 | 3.88E-22 | postive |
| HELLS | PPP3CB-AS1 | 0.562788906 | 3.40E-43 | postive |
| SP1 | PPP3CB-AS1 | 0.460722209 | 1.08E-27 | postive |
| GLS2 | PPP3CB-AS1 | 0.421919077 | 4.81E-23 | postive |
| GOT1 | PPP3CB-AS1 | 0.404202285 | 4.10E-21 | postive |
| MAPK8 | PPP3CB-AS1 | 0.450524038 | 2.06E-26 | postive |
| ACVR1B | PPP3CB-AS1 | 0.41164635 | 6.54E-22 | postive |
| YY1AP1 | PPP3CB-AS1 | 0.406708161 | 2.22E-21 | postive |
| SIRT1 | PPP3CB-AS1 | 0.461229845 | 9.29E-28 | postive |
| GPX4 | RAB11B-AS1 | 0.576243745 | 1.16E-45 | postive |
| SLC2A8 | RAB11B-AS1 | 0.416815663 | 1.78E-22 | postive |
| NOX1 | RAB11B-AS1 | 0.425462286 | 1.91E-23 | postive |
| PHKG2 | RAB11B-AS1 | 0.525531405 | 6.41E-37 | postive |
| MAP1LC3A | RAB11B-AS1 | 0.492380728 | 5.92E-32 | postive |
| CDKN2A | RAB11B-AS1 | 0.427973107 | 9.90E-24 | postive |
| ANO6 | RAB11B-AS1 | -0.43498225 | 1.52E-24 | negative |
| EGLN2 | RAB11B-AS1 | 0.514776663 | 3.01E-35 | postive |
| ISCU | AC116914.2 | 0.410197599 | 9.38E-22 | postive |
| PHKG2 | AC116914.2 | 0.429868487 | 5.99E-24 | postive |
| TAZ | AC116914.2 | 0.442817952 | 1.79E-25 | postive |
| GPX4 | AC005785.1 | 0.426516546 | 1.45E-23 | postive |
| ATF4 | AC005785.1 | 0.456858835 | 3.33E-27 | postive |
| ISCU | AC005785.1 | 0.401532848 | 7.83E-21 | postive |
| ZNF419 | AC005785.1 | 0.4551568 | 5.46E-27 | postive |
| NOX1 | AC005785.1 | 0.430846385 | 4.62E-24 | postive |
| PHKG2 | AC005785.1 | 0.642168352 | 1.36E-59 | postive |
| ANO6 | AC005785.1 | -0.447389014 | 4.99E-26 | negative |
| EGLN2 | AC005785.1 | 0.599277011 | 3.65E-50 | postive |
| TAZ | AC005785.1 | 0.586133155 | 1.49E-47 | postive |
| ELAVL1 | AC022075.1 | 0.425512218 | 1.89E-23 | postive |
| MTOR | AC125807.2 | 0.437629783 | 7.44E-25 | postive |
| LPCAT3 | AC125807.2 | 0.491947288 | 6.82E-32 | postive |
| PHKG2 | AL022328.3 | 0.420841708 | 6.35E-23 | postive |
| ATF4 | SNHG15 | 0.449067619 | 3.11E-26 | postive |
| SELENOS | SNHG15 | 0.400524355 | 9.98E-21 | postive |
| RPL8 | SNHG15 | 0.54553025 | 3.44E-40 | postive |
| SLC2A8 | SNHG15 | 0.410846315 | 7.98E-22 | postive |
| HRAS | SNHG15 | 0.456180824 | 4.06E-27 | postive |
| PHKG2 | SNHG15 | 0.457111478 | 3.10E-27 | postive |
| MAPK1 | SNHG15 | -0.409682994 | 1.07E-21 | negative |
| ANO6 | SNHG15 | -0.415362928 | 2.57E-22 | negative |
| EGLN2 | SNHG15 | 0.464967091 | 3.07E-28 | postive |
| TAZ | SNHG15 | 0.462725832 | 5.97E-28 | postive |
| HELLS | AC024075.3 | 0.433558509 | 2.24E-24 | postive |
| BRD4 | AC024075.3 | 0.409436391 | 1.13E-21 | postive |
| SETD1B | AC024075.3 | 0.468311079 | 1.13E-28 | postive |
| IREB2 | AC024075.3 | 0.405898874 | 2.71E-21 | postive |
| SP1 | AC024075.3 | 0.580439346 | 1.86E-46 | postive |
| GLS2 | AC024075.3 | 0.450081909 | 2.33E-26 | postive |
| ACVR1B | AC024075.3 | 0.432170893 | 3.25E-24 | postive |
| ATM | AC024075.3 | 0.500598889 | 3.91E-33 | postive |
| SIRT1 | AC024075.3 | 0.406307457 | 2.45E-21 | postive |
| SLC7A11 | AL391427.1 | 0.447195302 | 5.27E-26 | postive |
| AKR1C1 | AL391427.1 | 0.652418444 | 4.48E-62 | postive |
| AKR1C2 | AL391427.1 | 0.734981212 | 3.14E-86 | postive |
| AKR1C3 | AL391427.1 | 0.640219535 | 3.93E-59 | postive |
| GCLC | AL391427.1 | 0.478032419 | 5.74E-30 | postive |
| NQO1 | AL391427.1 | 0.495695694 | 2.00E-32 | postive |
| TXNRD1 | AL391427.1 | 0.479507531 | 3.62E-30 | postive |
| SRXN1 | AL391427.1 | 0.449311691 | 2.90E-26 | postive |
| GPX2 | AL391427.1 | 0.506547188 | 5.21E-34 | postive |
| MAFG | AL391427.1 | 0.48153232 | 1.92E-30 | postive |
| PRDX1 | AL391427.1 | 0.444706251 | 1.06E-25 | postive |
| G6PD | AL391427.1 | 0.4139746 | 3.65E-22 | postive |
| PGD | AL391427.1 | 0.470905968 | 5.14E-29 | postive |
| ABCC1 | AL391427.1 | 0.509064953 | 2.20E-34 | postive |
| CHMP6 | AC023509.3 | 0.451587074 | 1.52E-26 | postive |
| PHKG2 | AC023509.3 | 0.551855664 | 2.85E-41 | postive |
| MAP1LC3A | AC023509.3 | 0.47627875 | 9.88E-30 | postive |
| EGLN2 | AC023509.3 | 0.501824938 | 2.59E-33 | postive |
| TAZ | AC023509.3 | 0.424975425 | 2.17E-23 | postive |
| CDO1 | AL445250.1 | 0.445103709 | 9.47E-26 | postive |
| FLT3 | ITGB2-AS1 | 0.406649107 | 2.25E-21 | postive |
| MYB | ITGB2-AS1 | 0.456822744 | 3.37E-27 | postive |
| FANCD2 | SLC25A25-AS1 | 0.497136735 | 1.24E-32 | postive |
| HELLS | SLC25A25-AS1 | 0.483754417 | 9.50E-31 | postive |
| TUBE1 | SLC25A25-AS1 | 0.48595534 | 4.71E-31 | postive |
| ELAVL1 | SLC25A25-AS1 | 0.412614186 | 5.13E-22 | postive |
| GLS2 | SLC25A25-AS1 | 0.441242648 | 2.76E-25 | postive |
| ATG4D | SLC25A25-AS1 | 0.4502939 | 2.20E-26 | postive |
| MYB | SLC25A25-AS1 | 0.480787262 | 2.42E-30 | postive |
| LPIN1 | SLC25A25-AS1 | 0.445972612 | 7.43E-26 | postive |
| GPX4 | AC090617.5 | 0.437827652 | 7.05E-25 | postive |
| YWHAE | AC090617.5 | 0.431882536 | 3.51E-24 | postive |
| PHKG2 | AC090617.5 | 0.451305194 | 1.65E-26 | postive |
| MAP1LC3A | AC090617.5 | 0.455069858 | 5.60E-27 | postive |
| PHKG2 | HOXC-AS1 | 0.44712471 | 5.38E-26 | postive |
| MAP1LC3A | HOXC-AS1 | 0.432392057 | 3.06E-24 | postive |
| ZFP69B | BOLA3-AS1 | 0.441977049 | 2.26E-25 | postive |
| PHKG2 | AC003102.1 | 0.459340088 | 1.62E-27 | postive |
| ATG3 | AC083799.1 | 0.534386104 | 2.43E-38 | postive |
| SNX4 | AC083799.1 | 0.488041703 | 2.42E-31 | postive |
| PEBP1 | AC025166.1 | 0.409582287 | 1.09E-21 | postive |
| TAZ | AC025166.1 | 0.413820681 | 3.79E-22 | postive |
| NOX4 | LINC01705 | 0.452928267 | 1.04E-26 | postive |
| ATM | CCDC84-DT | 0.571983046 | 7.19E-45 | postive |
| BECN1 | AC004596.1 | 0.40398651 | 4.32E-21 | postive |
| SLC40A1 | AC145098.1 | 0.492240419 | 6.20E-32 | postive |
| ENPP2 | AC145098.1 | 0.578039949 | 5.30E-46 | postive |
| HERPUD1 | AC145098.1 | 0.449967748 | 2.41E-26 | postive |
| ALOX5 | AC145098.1 | 0.593856072 | 4.51E-49 | postive |
| CYBB | AC145098.1 | 0.685949715 | 6.52E-71 | postive |
| FLT3 | AC145098.1 | 0.600044917 | 2.55E-50 | postive |
| TLR4 | AC145098.1 | 0.538857842 | 4.49E-39 | postive |
| ATM | AC145098.1 | 0.458037155 | 2.37E-27 | postive |
| ZNF419 | AC005261.1 | 0.510878462 | 1.17E-34 | postive |
| PHKG2 | AC005261.1 | 0.417686351 | 1.42E-22 | postive |
| ANO6 | AC005261.1 | -0.41994409 | 8.00E-23 | negative |
| GCLC | AL390755.1 | 0.41243232 | 5.37E-22 | postive |
| TXNRD1 | AL390755.1 | 0.415898859 | 2.24E-22 | postive |
| GABARAPL1 | AL390755.1 | 0.411574237 | 6.66E-22 | postive |
| ABCC1 | AL390755.1 | 0.430418647 | 5.18E-24 | postive |
| SP1 | AC244517.7 | 0.429145263 | 7.26E-24 | postive |
| NFE2L2 | AL035661.1 | 0.462833363 | 5.79E-28 | postive |
| TXNRD1 | AL035661.1 | 0.441426777 | 2.63E-25 | postive |
| SRXN1 | AL035661.1 | 0.477110526 | 7.64E-30 | postive |
| GPX2 | AL035661.1 | 0.501873172 | 2.55E-33 | postive |
| PSAT1 | AL035661.1 | 0.412692137 | 5.03E-22 | postive |
| KLHL24 | AL035661.1 | 0.474279004 | 1.83E-29 | postive |
| G6PD | AL035661.1 | 0.417721432 | 1.41E-22 | postive |
| PGD | AL035661.1 | 0.452421611 | 1.20E-26 | postive |
| GLS2 | AL035661.1 | 0.475490007 | 1.26E-29 | postive |
| KEAP1 | AL035661.1 | 0.490265962 | 1.18E-31 | postive |
| ATG4D | AL035661.1 | 0.445764845 | 7.87E-26 | postive |
| ABCC1 | AL035661.1 | 0.430078759 | 5.67E-24 | postive |
| IDH1 | AL035661.1 | 0.473351613 | 2.43E-29 | postive |
| MIOX | AC015660.2 | 0.487583121 | 2.80E-31 | postive |
| MYB | AL445471.1 | 0.497878824 | 9.69E-33 | postive |
| GPX4 | MINCR | 0.438206411 | 6.36E-25 | postive |
| RPL8 | MINCR | 0.564142517 | 1.94E-43 | postive |
| SLC2A8 | MINCR | 0.459657995 | 1.47E-27 | postive |
| PHKG2 | MINCR | 0.491954758 | 6.80E-32 | postive |
| PEBP1 | MINCR | 0.488119209 | 2.36E-31 | postive |
| EGLN2 | MINCR | 0.477482625 | 6.80E-30 | postive |
| TAZ | MINCR | 0.456281207 | 3.94E-27 | postive |
| PHKG2 | AC245100.7 | 0.414750823 | 3.00E-22 | postive |
| HMOX1 | AP000238.1 | 0.63760747 | 1.61E-58 | postive |
| ATM | AP003486.1 | 0.45150123 | 1.56E-26 | postive |
| WIPI2 | AC091729.3 | 0.479926638 | 3.17E-30 | postive |
| MUC1 | AC093001.1 | 0.622368039 | 4.66E-55 | postive |
| KLHL24 | AC026471.4 | 0.40924721 | 1.19E-21 | postive |
| TUBE1 | AC026471.4 | 0.424161728 | 2.69E-23 | postive |
| MYB | AC026471.4 | 0.403237966 | 5.18E-21 | postive |
| LPIN1 | AC026471.4 | 0.405942531 | 2.68E-21 | postive |
| YY1AP1 | AC026471.4 | 0.429105172 | 7.34E-24 | postive |
| NFE2L2 | AL359220.1 | 0.413415122 | 4.20E-22 | postive |
| NOX1 | AL604028.1 | 0.443998176 | 1.29E-25 | postive |
| PHKG2 | AL604028.1 | 0.469862904 | 7.05E-29 | postive |
| GPX4 | LINC02586 | 0.48987904 | 1.34E-31 | postive |
| CISD1 | LINC02586 | 0.423147944 | 3.50E-23 | postive |
| ISCU | LINC02586 | 0.479167395 | 4.03E-30 | postive |
| SOCS1 | LINC02586 | 0.476568349 | 9.04E-30 | postive |
| STMN1 | AC005332.5 | 0.405862084 | 2.73E-21 | postive |
| YY1AP1 | AC005332.5 | 0.441775612 | 2.39E-25 | postive |
| AIFM2 | LINC02765 | 0.437437888 | 7.84E-25 | postive |
| MT3 | LINC02765 | 0.410232903 | 9.30E-22 | postive |
| PEBP1 | LINC02765 | 0.443215834 | 1.60E-25 | postive |
| CDO1 | LINC02765 | 0.441479711 | 2.59E-25 | postive |
| MIOX | LINC02765 | 0.475153733 | 1.40E-29 | postive |
| KLHL24 | LINC00865 | 0.446942635 | 5.66E-26 | postive |
| FANCD2 | AC012073.1 | 0.586375699 | 1.34E-47 | postive |
| HELLS | AC012073.1 | 0.585970652 | 1.61E-47 | postive |
| HMGB1 | AC012073.1 | 0.43847379 | 5.91E-25 | postive |
| ELAVL1 | AC012073.1 | 0.508352745 | 2.81E-34 | postive |
| STMN1 | AC012073.1 | 0.527201822 | 3.49E-37 | postive |
| RRM2 | AC012073.1 | 0.538186488 | 5.79E-39 | postive |
| NOX1 | AC012073.1 | 0.428431717 | 8.77E-24 | postive |
| YY1AP1 | AC012073.1 | 0.408189042 | 1.54E-21 | postive |
| GPX4 | DLG5-AS1 | 0.529437544 | 1.53E-37 | postive |
| PHKG2 | DLG5-AS1 | 0.618979302 | 2.58E-54 | postive |
| MAP1LC3A | DLG5-AS1 | 0.432852235 | 2.70E-24 | postive |
| EGLN2 | DLG5-AS1 | 0.487073825 | 3.30E-31 | postive |
| TAZ | DLG5-AS1 | 0.450616915 | 2.00E-26 | postive |
| ALOX5 | SOCAR | 0.403136258 | 5.31E-21 | postive |
| CYBB | SOCAR | 0.419069849 | 1.00E-22 | postive |
| IFNG | SOCAR | 0.458857821 | 1.86E-27 | postive |
| TUBE1 | AP006621.2 | 0.4077735 | 1.71E-21 | postive |
| PHKG2 | AC012146.1 | 0.469263656 | 8.45E-29 | postive |
| MAP1LC3A | AC012146.1 | 0.456769222 | 3.42E-27 | postive |
| PHKG2 | AL157813.1 | 0.459955876 | 1.35E-27 | postive |
| GABARAPL2 | AL157813.1 | 0.403243763 | 5.17E-21 | postive |
| PHKG2 | DBH-AS1 | 0.404089411 | 4.21E-21 | postive |
| IFNG | DBH-AS1 | 0.540429149 | 2.46E-39 | postive |
| AIFM2 | AC131097.2 | 0.508760502 | 2.44E-34 | postive |
| PEBP1 | AC131097.2 | 0.41655882 | 1.90E-22 | postive |
| MIOX | AC131097.2 | 0.474052524 | 1.96E-29 | postive |
| FANCD2 | AC025176.1 | 0.541419867 | 1.68E-39 | postive |
| TP53 | AC025176.1 | 0.405361308 | 3.09E-21 | postive |
| HELLS | AC025176.1 | 0.533390613 | 3.53E-38 | postive |
| ISCU | AC025176.1 | 0.450017074 | 2.38E-26 | postive |
| STMN1 | AC025176.1 | 0.549903498 | 6.18E-41 | postive |
| NOX1 | AC025176.1 | 0.534664939 | 2.19E-38 | postive |
| MYB | AC025176.1 | 0.463635124 | 4.56E-28 | postive |

**Supplementary Table 3:** 196 DEFRlncRNA by differential expression analysis by the fresh pairing algorithm.

| LncRNA | conMean | treatMean | logFC | pValue | fdr |
| --- | --- | --- | --- | --- | --- |
| AC079336.5 | 0.073676759 | 0.246727766 | 1.743638579 | 0.000333068 | 0.000672877 |
| AL049775.1 | 0.065639028 | 0.368007084 | 2.487107757 | 3.48E-09 | 2.17E-08 |
| AC004704.1 | 0.023938076 | 0.452572213 | 4.240768909 | 3.56E-07 | 1.30E-06 |
| SLC2A1-AS1 | 0.151557771 | 0.452174116 | 1.577010587 | 5.73E-13 | 8.18E-12 |
| AC131159.1 | 0.075749598 | 0.228304904 | 1.591651721 | 1.26E-05 | 3.43E-05 |
| MNX1-AS1 | 0.086917906 | 0.410741444 | 2.240505205 | 2.78E-07 | 1.07E-06 |
| AL512274.1 | 20.18250323 | 5.526213891 | -1.868741815 | 1.31E-10 | 1.11E-09 |
| LINC02195 | 0.30193236 | 1.385191694 | 2.197788349 | 6.23E-12 | 7.24E-11 |
| MIAT | 0.156491241 | 0.970438367 | 2.632554682 | 1.29E-14 | 2.62E-13 |
| LHX1-DT | 0.012292127 | 0.364814304 | 4.891355846 | 8.12E-17 | 2.78E-15 |
| LINC01694 | 0.049415006 | 0.475339035 | 3.265935751 | 2.14E-06 | 6.87E-06 |
| AL590666.2 | 0.260007951 | 1.707629929 | 2.715367705 | 5.32E-09 | 3.15E-08 |
| LINC01614 | 0.032467522 | 2.195213921 | 6.079220463 | 7.78E-21 | 9.33E-19 |
| SLCO4A1-AS1 | 1.604012338 | 0.422780676 | -1.923703895 | 9.28E-05 | 0.000209677 |
| AC132192.2 | 0.179408958 | 0.629415506 | 1.810760793 | 4.84E-12 | 5.86E-11 |
| FOXD2-AS1 | 0.20959876 | 1.344568122 | 2.681440764 | 3.37E-26 | 4.03E-23 |
| MELTF-AS1 | 0.743938075 | 2.301486823 | 1.62931174 | 9.42E-09 | 5.18E-08 |
| AC011461.1 | 0.092096769 | 0.300511297 | 1.706196775 | 1.43E-09 | 9.63E-09 |
| AC109460.2 | 0.154669175 | 0.446897338 | 1.530757754 | 5.86E-10 | 4.33E-09 |
| LINC01139 | 0.506724188 | 1.901524471 | 1.907883903 | 0.009898505 | 0.015320942 |
| AC024592.2 | 1.344331994 | 0.223563771 | -2.588131148 | 1.03E-10 | 9.04E-10 |
| AL139089.1 | 0.14016508 | 0.463266936 | 1.724716752 | 2.29E-11 | 2.38E-10 |
| LINC01322 | 0.038893643 | 1.166655763 | 4.906700741 | 9.17E-19 | 6.46E-17 |
| AC026785.3 | 0.018304801 | 0.344117991 | 4.232609349 | 6.55E-07 | 2.29E-06 |
| PCAT7 | 0.115574302 | 0.94650843 | 3.033794703 | 1.80E-14 | 3.54E-13 |
| LINC00839 | 0.326743264 | 2.525315784 | 2.950234403 | 1.78E-07 | 7.19E-07 |
| ELFN1-AS1 | 0.030002508 | 0.87117179 | 4.859802245 | 6.00E-09 | 3.46E-08 |
| AC012065.3 | 0.090682011 | 0.74796574 | 3.044083893 | 9.81E-16 | 2.67E-14 |
| LINC01305 | 0.016896043 | 0.412361921 | 4.609153824 | 1.71E-07 | 7.00E-07 |
| LINC01943 | 0.132950969 | 0.560285923 | 2.075268957 | 6.13E-19 | 4.59E-17 |
| AC011676.1 | 0.091213988 | 0.290842882 | 1.672913009 | 1.90E-08 | 9.56E-08 |
| AC015878.1 | 2.769233101 | 0.190929583 | -3.858373935 | 5.94E-07 | 2.09E-06 |
| AC009093.2 | 0.042210596 | 0.23903049 | 2.501517566 | 2.76E-19 | 2.45E-17 |
| AC104041.1 | 0.056161839 | 0.502791129 | 3.162297118 | 7.07E-10 | 5.17E-09 |
| AC008735.2 | 0.392380637 | 1.413265444 | 1.848706706 | 4.09E-11 | 3.86E-10 |
| AC002401.4 | 0.359653209 | 1.944943067 | 2.435049544 | 1.18E-07 | 4.92E-07 |
| AC008115.3 | 0.204718457 | 0.592395065 | 1.53291844 | 4.04E-11 | 3.84E-10 |
| AC104564.3 | 0.081910765 | 0.237915562 | 1.538324662 | 9.62E-08 | 4.12E-07 |
| AL136018.1 | 0.296993627 | 1.135414972 | 1.934715793 | 9.89E-07 | 3.32E-06 |
| AL513327.1 | 0.092763569 | 0.288485426 | 1.636868198 | 2.78E-13 | 4.50E-12 |
| CASC15 | 0.11807301 | 0.366500002 | 1.634133989 | 1.25E-10 | 1.08E-09 |
| KCNMB2-AS1 | 0.352266968 | 2.179696096 | 2.629385896 | 7.08E-13 | 9.98E-12 |
| LINC01096 | 0.032469596 | 0.220154059 | 2.761352096 | 1.12E-12 | 1.55E-11 |
| AL365181.3 | 0.220337273 | 2.490361755 | 3.498569855 | 4.33E-12 | 5.29E-11 |
| SLC12A5-AS1 | 0.015808303 | 0.221778138 | 3.810362722 | 4.80E-23 | 1.15E-20 |
| AC010719.1 | 0.119459808 | 0.486673781 | 2.026429742 | 1.76E-09 | 1.17E-08 |
| Z97653.1 | 0.786794214 | 0.192987104 | -2.027481905 | 6.45E-15 | 1.47E-13 |
| AL137246.2 | 6.025001179 | 0.073148723 | -6.363985042 | 3.83E-07 | 1.39E-06 |
| AC013731.1 | 0.053086683 | 0.21643845 | 2.027534914 | 1.98E-10 | 1.62E-09 |
| C5orf66-AS1 | 11.77808904 | 3.535120782 | -1.736274073 | 3.62E-12 | 4.48E-11 |
| NCBP2-AS1 | 0.058338421 | 0.222509629 | 1.931349512 | 6.49E-10 | 4.77E-09 |
| AC026401.3 | 5.186638361 | 16.66771484 | 1.684184637 | 4.04E-22 | 6.92E-20 |
| AC093788.1 | 0.110587278 | 0.330255457 | 1.578396968 | 4.06E-10 | 3.12E-09 |
| AC026740.1 | 0.448803544 | 1.567198387 | 1.804031844 | 4.47E-11 | 4.15E-10 |
| NCK1-DT | 1.045188733 | 2.976226342 | 1.50972077 | 6.56E-16 | 1.87E-14 |
| MCCC1-AS1 | 0.080353606 | 0.280353218 | 1.802810948 | 3.52E-07 | 1.29E-06 |
| AC112236.1 | 1.005010831 | 0.338759353 | -1.568878367 | 1.38E-05 | 3.74E-05 |
| TBILA | 0.149886142 | 0.644309227 | 2.103886256 | 8.64E-15 | 1.85E-13 |
| LINC02561 | 0.060605713 | 0.646129656 | 3.414297985 | 2.33E-11 | 2.41E-10 |
| AC025154.2 | 1.187413329 | 0.122388162 | -3.278286285 | 6.61E-06 | 1.92E-05 |
| LINC02454 | 0.050391449 | 0.803105938 | 3.994339455 | 1.69E-16 | 5.52E-15 |
| AC092171.4 | 0.074070624 | 0.282832493 | 1.932974469 | 4.14E-09 | 2.54E-08 |
| AL158166.1 | 0.101294062 | 0.381337196 | 1.912517663 | 1.13E-09 | 7.85E-09 |
| AL365181.2 | 0.029400176 | 0.508709566 | 4.112945517 | 1.03E-09 | 7.32E-09 |
| STARD4-AS1 | 0.089148611 | 0.300775852 | 1.754404523 | 3.50E-13 | 5.24E-12 |
| LINC01050 | 0.032426791 | 0.252857348 | 2.963065529 | 2.53E-12 | 3.29E-11 |
| U62317.4 | 0.18403826 | 0.861365689 | 2.226620135 | 7.65E-17 | 2.78E-15 |
| LINC02244 | 0.032592812 | 0.40499255 | 3.635269653 | 2.85E-07 | 1.09E-06 |
| AC007497.1 | 0.062012713 | 0.224507759 | 1.856129398 | 3.99E-09 | 2.47E-08 |
| AL122125.1 | 0.101674897 | 0.301213666 | 1.56682369 | 6.75E-09 | 3.81E-08 |
| AC006206.2 | 0.224801368 | 0.888345988 | 1.982470866 | 1.13E-10 | 9.78E-10 |
| AP002907.1 | 0.081937545 | 0.243432092 | 1.570922794 | 4.29E-10 | 3.27E-09 |
| AC128709.2 | 0.085130847 | 0.780450229 | 3.196552739 | 6.94E-06 | 2.00E-05 |
| AP005482.2 | 0.094131896 | 0.692976149 | 2.880050134 | 2.81E-07 | 1.08E-06 |
| LINC01929 | 0.010482705 | 0.432155125 | 5.365466261 | 2.48E-22 | 4.95E-20 |
| DLGAP1-AS2 | 0.22803229 | 0.927392659 | 2.02394218 | 1.26E-13 | 2.16E-12 |
| ATP1B3-AS1 | 0.241986826 | 0.999001753 | 2.045558704 | 8.23E-12 | 9.30E-11 |
| AF165147.1 | 0.038549171 | 0.373997827 | 3.278258143 | 4.57E-08 | 2.09E-07 |
| MIR9-3HG | 0.102703781 | 1.41855042 | 3.787856233 | 3.04E-11 | 2.96E-10 |
| AC053503.3 | 1.274206299 | 0.231322286 | -2.461622702 | 0.002199093 | 0.003885713 |
| MIR1-1HG | 4.787576924 | 0.404211835 | -3.566112201 | 6.94E-05 | 0.000160535 |
| AC091563.1 | 6.461199 | 1.09062719 | -2.566643881 | 2.11E-18 | 1.26E-16 |
| AL021807.1 | 0.369597787 | 1.649749147 | 2.158218646 | 7.82E-12 | 8.93E-11 |
| AC010789.1 | 0.036179694 | 0.686533328 | 4.246077636 | 3.41E-06 | 1.05E-05 |
| LINC02820 | 0.159405569 | 0.87503945 | 2.45664603 | 0.009950069 | 0.01538088 |
| LINC02100 | 0.075732111 | 0.762552255 | 3.331859162 | 2.24E-19 | 2.24E-17 |
| AL035461.3 | 0.699630117 | 1.982414291 | 1.502594194 | 4.60E-10 | 3.47E-09 |
| FOXD3-AS1 | 0.22761119 | 1.089819199 | 2.259445421 | 6.62E-07 | 2.30E-06 |
| AC021028.1 | 0.146256094 | 0.716892057 | 2.293259165 | 2.34E-15 | 6.10E-14 |
| ZNF710-AS1 | 5.14423107 | 1.301694232 | -1.982564848 | 5.81E-14 | 1.04E-12 |
| AC026333.4 | 0.096313362 | 0.297218687 | 1.625716958 | 2.06E-05 | 5.38E-05 |
| AL596442.2 | 0.074777246 | 0.252294535 | 1.75443771 | 1.80E-11 | 1.94E-10 |
| LINC00707 | 0.572839074 | 1.890698981 | 1.722717881 | 2.29E-06 | 7.32E-06 |
| AC114956.1 | 0.090835074 | 1.200164962 | 3.723839432 | 5.27E-24 | 1.58E-21 |
| AC019171.1 | 0.080820103 | 0.679739735 | 3.072196356 | 0.023778046 | 0.033992958 |
| AC009093.1 | 0.049245832 | 0.361798652 | 2.877113507 | 3.78E-19 | 3.02E-17 |
| AC010331.1 | 0.099744922 | 0.291469308 | 1.547028676 | 5.34E-09 | 3.15E-08 |
| AC048341.2 | 0.210913631 | 1.084458352 | 2.362250404 | 9.56E-15 | 1.98E-13 |
| AC016773.2 | 0.117630109 | 0.525959404 | 2.160694059 | 2.56E-14 | 4.78E-13 |
| LINC01311 | 0.146706056 | 0.452842295 | 1.626080285 | 2.63E-12 | 3.39E-11 |
| CDKN2B-AS1 | 0.043370154 | 0.326963045 | 2.914353127 | 2.91E-12 | 3.67E-11 |
| AL031186.1 | 0.175031576 | 0.604166238 | 1.787330355 | 7.88E-11 | 7.15E-10 |
| AC114730.3 | 0.080965064 | 0.233507687 | 1.528098622 | 1.20E-06 | 3.94E-06 |
| LINC00911 | 0.020292102 | 0.327508895 | 4.01254385 | 1.06E-09 | 7.50E-09 |
| AC016394.3 | 0.322101357 | 0.913000429 | 1.503100801 | 1.42E-12 | 1.87E-11 |
| AL133215.2 | 0.167789258 | 0.481717333 | 1.521536478 | 1.80E-11 | 1.94E-10 |
| AC004034.1 | 0.132144389 | 0.520454159 | 1.977655931 | 1.55E-05 | 4.16E-05 |
| AC055822.1 | 0.375973163 | 1.084158475 | 1.527874063 | 1.25E-11 | 1.39E-10 |
| C5orf34-AS1 | 0.238100323 | 3.013935811 | 3.662007209 | 2.53E-24 | 1.01E-21 |
| TTLL11-IT1 | 0.042711254 | 0.53168813 | 3.637892113 | 3.09E-16 | 9.50E-15 |
| AC103563.7 | 1.439206603 | 0.181598143 | -2.986452356 | 7.99E-17 | 2.78E-15 |
| AL355488.1 | 0.192042323 | 0.688198471 | 1.841400397 | 3.19E-10 | 2.52E-09 |
| AC016877.3 | 0.100048881 | 0.409418026 | 2.032869593 | 5.08E-09 | 3.03E-08 |
| AC145207.8 | 0.14631856 | 0.474679191 | 1.697840022 | 0.007471637 | 0.011855657 |
| C10orf71-AS1 | 1.516174644 | 0.092312467 | -4.037766637 | 6.14E-05 | 0.000144239 |
| ZKSCAN2-DT | 0.091917311 | 0.307321677 | 1.741341034 | 1.04E-11 | 1.16E-10 |
| AL499627.1 | 1.086795037 | 0.161284942 | -2.752396228 | 0.004028032 | 0.00673024 |
| AL157871.2 | 0.072800028 | 0.442798075 | 2.604638041 | 5.83E-11 | 5.37E-10 |
| AC020907.4 | 0.080013938 | 0.25909027 | 1.695131595 | 6.07E-09 | 3.46E-08 |
| LINC01355 | 0.134767969 | 0.447363927 | 1.730971281 | 6.63E-11 | 6.07E-10 |
| FOXP4-AS1 | 0.075626655 | 0.293024085 | 1.954052535 | 4.20E-05 | 0.00010288 |
| AP001029.1 | 0.05587399 | 0.2605697 | 2.221420579 | 1.96E-05 | 5.15E-05 |
| TENM3-AS1 | 0.082642702 | 0.409906213 | 2.310334522 | 1.12E-10 | 9.78E-10 |
| DDX11-AS1 | 0.070905867 | 0.307159134 | 2.115009381 | 5.55E-15 | 1.33E-13 |
| ITGB1-DT | 0.029592297 | 0.30601971 | 3.370331001 | 3.03E-15 | 7.72E-14 |
| AL022322.1 | 0.147505528 | 0.449352433 | 1.60707839 | 0.001143404 | 0.002091294 |
| MAFA-AS1 | 0.216262253 | 0.818341397 | 1.91992096 | 2.33E-07 | 9.11E-07 |
| AL355803.1 | 0.159530568 | 0.509013069 | 1.67386981 | 2.65E-05 | 6.79E-05 |
| AC134312.5 | 0.04472852 | 0.955057431 | 4.416320568 | 1.41E-24 | 8.44E-22 |
| MIR155HG | 0.207405096 | 0.66335802 | 1.67733637 | 1.90E-09 | 1.25E-08 |
| AC016735.1 | 0.105632349 | 0.342831882 | 1.698449572 | 0.005107111 | 0.008346957 |
| AC089983.1 | 0.023883783 | 0.277471283 | 3.538235168 | 5.53E-10 | 4.11E-09 |
| AL928654.2 | 0.414423355 | 1.174280769 | 1.502600184 | 2.64E-09 | 1.71E-08 |
| YEATS2-AS1 | 0.076403554 | 0.26484662 | 1.793445451 | 6.55E-15 | 1.47E-13 |
| AC018716.1 | 0.073773722 | 0.286702685 | 1.958376478 | 8.88E-06 | 2.47E-05 |
| AC011462.4 | 0.113648334 | 0.355703487 | 1.646098577 | 2.03E-07 | 8.09E-07 |
| LINC02560 | 20.67786277 | 6.949905562 | -1.5730218 | 1.96E-08 | 9.85E-08 |
| AC010731.2 | 0.034969522 | 0.285201123 | 3.027809681 | 0.000249293 | 0.000514919 |
| LINC00942 | 0.119661257 | 1.267580579 | 3.40504943 | 0.00010459 | 0.000234203 |
| KDM4A-AS1 | 0.151950899 | 0.640102539 | 2.07469782 | 5.69E-18 | 2.84E-16 |
| LINC02544 | 0.121997573 | 0.630801153 | 2.370332849 | 1.47E-14 | 2.93E-13 |
| LINC00941 | 0.069617675 | 1.346847886 | 4.273989469 | 4.67E-21 | 6.22E-19 |
| DELEC1 | 0.069643186 | 0.424898707 | 2.609064837 | 2.89E-07 | 1.10E-06 |
| AC034213.1 | 0.04591712 | 0.574346097 | 3.644816302 | 1.01E-09 | 7.23E-09 |
| AL132712.1 | 0.576654463 | 1.949381941 | 1.757237778 | 4.77E-15 | 1.17E-13 |
| AC092119.2 | 0.044502515 | 0.219196827 | 2.300268125 | 1.05E-12 | 1.46E-11 |
| AL359881.1 | 0.063719488 | 0.268321959 | 2.074158547 | 0.00027586 | 0.000565892 |
| AL031600.1 | 0.114068316 | 0.445782244 | 1.966441031 | 1.54E-11 | 1.69E-10 |
| STEAP3-AS1 | 0.067531892 | 0.283635987 | 2.070399716 | 1.41E-08 | 7.32E-08 |
| AL591501.1 | 0.043872537 | 0.262270785 | 2.579667062 | 0.013701273 | 0.02062076 |
| SCAT2 | 0.053063839 | 0.486912378 | 3.197861211 | 1.51E-18 | 1.01E-16 |
| HOTAIR | 0.015785672 | 0.298965008 | 4.2432891 | 2.05E-18 | 1.26E-16 |
| HS1BP3-IT1 | 0.051866362 | 0.592264411 | 3.513370304 | 1.34E-15 | 3.57E-14 |
| AC112721.2 | 0.01487533 | 0.32248353 | 4.438231881 | 3.63E-18 | 2.07E-16 |
| AL513320.1 | 0.060960558 | 0.253131423 | 2.0539386 | 3.73E-11 | 3.57E-10 |
| AP000251.1 | 0.225318402 | 0.733736368 | 1.703296651 | 1.45E-05 | 3.91E-05 |
| LINC01508 | 0.045425327 | 0.259917445 | 2.516484666 | 0.006972988 | 0.011167968 |
| C1RL-AS1 | 0.224981859 | 0.708176734 | 1.654300773 | 5.66E-16 | 1.65E-14 |
| LINC01711 | 0.06080225 | 0.42586139 | 2.808187312 | 4.31E-14 | 7.94E-13 |
| LINC01063 | 0.175870806 | 0.558542726 | 1.667151622 | 2.56E-06 | 8.07E-06 |
| TMPO-AS1 | 0.353316181 | 1.284660553 | 1.862355476 | 3.37E-17 | 1.39E-15 |
| NRIR | 0.047707901 | 0.311876989 | 2.708677004 | 3.29E-13 | 5.00E-12 |
| LINC02551 | 0.041497963 | 0.356338858 | 3.10213739 | 1.94E-14 | 3.75E-13 |
| NKILA | 0.228551926 | 1.089968041 | 2.253691951 | 4.30E-15 | 1.07E-13 |
| AC005392.2 | 25.94663987 | 4.88070156 | -2.410387276 | 5.62E-08 | 2.52E-07 |
| AC128709.1 | 0.057872719 | 0.426011019 | 2.879935423 | 3.03E-05 | 7.70E-05 |
| MSC-AS1 | 0.337698889 | 1.311973563 | 1.957929311 | 2.73E-12 | 3.48E-11 |
| AC099850.4 | 2.90433139 | 10.64052708 | 1.873291635 | 8.48E-20 | 9.24E-18 |
| AC068473.3 | 0.042405169 | 0.253928774 | 2.582111839 | 4.87E-06 | 1.45E-05 |
| AL031670.1 | 0.124720746 | 0.409688703 | 1.715826646 | 2.37E-11 | 2.43E-10 |
| LINC02878 | 0.0871595 | 0.310270945 | 1.831798786 | 8.88E-16 | 2.47E-14 |
| AC011481.1 | 0.054843283 | 0.214074916 | 1.964728918 | 6.05E-09 | 3.46E-08 |
| LINC02446 | 0.353739937 | 1.409461689 | 1.994383251 | 0.003268022 | 0.00553761 |
| AC104031.1 | 0.253092872 | 1.012589813 | 2.000311092 | 4.55E-10 | 3.45E-09 |
| LINC00944 | 0.066898224 | 0.582579818 | 3.1224159 | 2.86E-19 | 2.45E-17 |
| TRPM2-AS | 0.088525076 | 0.711031355 | 3.005755092 | 2.75E-09 | 1.74E-08 |
| AL592424.1 | 1.462362282 | 0.23045222 | -2.665761196 | 0.003227806 | 0.005477212 |
| AC019080.4 | 0.08199802 | 1.089251248 | 3.73160388 | 8.20E-06 | 2.31E-05 |
| LINC02870 | 0.173474419 | 0.815580943 | 2.233105129 | 2.14E-11 | 2.24E-10 |
| LINC01405 | 4.207189054 | 0.342424709 | -3.618997934 | 0.001977172 | 0.003519542 |
| AC027796.4 | 0.31959565 | 0.912762885 | 1.51399236 | 9.56E-11 | 8.48E-10 |
| LINC01269 | 5.237450701 | 1.358473293 | -1.946878556 | 1.87E-11 | 1.99E-10 |
| LINC00519 | 0.784991822 | 5.302822662 | 2.756010974 | 1.71E-16 | 5.52E-15 |
| AC004253.1 | 0.117377526 | 0.350443943 | 1.578027481 | 4.84E-08 | 2.20E-07 |
| AL355472.3 | 0.050341092 | 0.280568916 | 2.478546766 | 8.94E-07 | 3.03E-06 |
| MIR133A1HG | 3.473337857 | 0.099351941 | -5.127630792 | 1.19E-05 | 3.27E-05 |
| TYMSOS | 0.490511743 | 1.444651564 | 1.558361989 | 1.75E-07 | 7.12E-07 |
| AL391427.1 | 0.081880769 | 0.561095316 | 2.776649311 | 6.49E-06 | 1.89E-05 |
| AL445250.1 | 0.065310316 | 0.350403266 | 2.423633419 | 6.29E-06 | 1.84E-05 |
| HOXC-AS1 | 0.061637171 | 0.554984171 | 3.170574062 | 4.74E-18 | 2.47E-16 |
| AC015660.2 | 0.039951056 | 0.521607235 | 3.706658321 | 2.31E-06 | 7.35E-06 |
| AL445471.1 | 0.042634826 | 0.214871083 | 2.33336707 | 1.20E-12 | 1.64E-11 |
| AC012073.1 | 0.416713357 | 1.511429572 | 1.858786506 | 1.60E-21 | 2.40E-19 |
| SOCAR | 0.07503426 | 0.255689317 | 1.768770507 | 6.14E-15 | 1.44E-13 |
| DBH-AS1 | 0.122636042 | 0.43407471 | 1.823560334 | 5.03E-05 | 0.000120416 |
| AC025176.1 | 0.040551818 | 0.343994817 | 3.084548326 | 6.61E-15 | 1.47E-13 |

**Supplementary Table 4:** 13,444 DEFRlncRNA pairs by differential expression analysis.

| ID |
| --- |
| AC079336.5|AL049775.1 |
| AC079336.5|AC004704.1 |
| AC079336.5|AC131159.1 |
| AC079336.5|MNX1-AS1 |
| AC079336.5|LHX1-DT |
| AC079336.5|LINC01694 |
| AC079336.5|LINC01614 |
| AC079336.5|SLCO4A1-AS1 |
| AC079336.5|AC011461.1 |
| AC079336.5|LINC01139 |
| AC079336.5|AC024592.2 |
| AC079336.5|LINC01322 |
| AC079336.5|AC026785.3 |
| AC079336.5|PCAT7 |
| AC079336.5|ELFN1-AS1 |
| AC079336.5|AC012065.3 |
| AC079336.5|LINC01305 |
| AC079336.5|AC011676.1 |
| AC079336.5|AC015878.1 |
| AC079336.5|AC009093.2 |
| AC079336.5|AC104041.1 |
| AC079336.5|AC002401.4 |
| AC079336.5|AC104564.3 |
| AC079336.5|AL136018.1 |
| AC079336.5|CASC15 |
| AC079336.5|LINC01096 |
| AC079336.5|SLC12A5-AS1 |
| AC079336.5|AC010719.1 |
| AC079336.5|Z97653.1 |
| AC079336.5|AC013731.1 |
| AC079336.5|NCBP2-AS1 |
| AC079336.5|AC093788.1 |
| AC079336.5|MCCC1-AS1 |
| AC079336.5|AC112236.1 |
| AC079336.5|LINC02561 |
| AC079336.5|LINC02454 |
| AC079336.5|AC092171.4 |
| AC079336.5|AL158166.1 |
| AC079336.5|AL365181.2 |
| AC079336.5|STARD4-AS1 |
| AC079336.5|LINC01050 |
| AC079336.5|LINC02244 |
| AC079336.5|AC007497.1 |
| AC079336.5|AL122125.1 |
| AC079336.5|AP002907.1 |
| AC079336.5|AC128709.2 |
| AC079336.5|AP005482.2 |
| AC079336.5|LINC01929 |
| AC079336.5|AF165147.1 |
| AC079336.5|MIR9-3HG |
| AC079336.5|AC053503.3 |
| AC079336.5|MIR1-1HG |
| AC079336.5|AC010789.1 |
| AC079336.5|LINC02820 |
| AC079336.5|LINC02100 |
| AC079336.5|FOXD3-AS1 |
| AC079336.5|AC026333.4 |
| AC079336.5|AL596442.2 |
| AC079336.5|LINC00707 |
| AC079336.5|AC019171.1 |
| AC079336.5|AC009093.1 |
| AC079336.5|AC010331.1 |
| AC079336.5|CDKN2B-AS1 |
| AC079336.5|AC114730.3 |
| AC079336.5|LINC00911 |
| AC079336.5|AC004034.1 |
| AC079336.5|TTLL11-IT1 |
| AC079336.5|AC103563.7 |
| AC079336.5|AC016877.3 |
| AC079336.5|AC145207.8 |
| AC079336.5|C10orf71-AS1 |
| AC079336.5|ZKSCAN2-DT |
| AC079336.5|AL499627.1 |
| AC079336.5|AL157871.2 |
| AC079336.5|AC020907.4 |
| AC079336.5|FOXP4-AS1 |
| AC079336.5|AP001029.1 |
| AC079336.5|TENM3-AS1 |
| AC079336.5|DDX11-AS1 |
| AC079336.5|ITGB1-DT |
| AC079336.5|AL022322.1 |
| AC079336.5|MAFA-AS1 |
| AC079336.5|AL355803.1 |
| AC079336.5|AC016735.1 |
| AC079336.5|AC089983.1 |
| AC079336.5|YEATS2-AS1 |
| AC079336.5|AC018716.1 |
| AC079336.5|AC011462.4 |
| AC079336.5|AC010731.2 |
| AC079336.5|LINC00942 |
| AC079336.5|LINC02544 |
| AC079336.5|LINC00941 |
| AC079336.5|DELEC1 |
| AC079336.5|AC034213.1 |
| AC079336.5|AC092119.2 |
| AC079336.5|AL359881.1 |
| AC079336.5|AL031600.1 |
| AC079336.5|STEAP3-AS1 |
| AC079336.5|AL591501.1 |
| AC079336.5|SCAT2 |
| AC079336.5|HOTAIR |
| AC079336.5|HS1BP3-IT1 |
| AC079336.5|AC112721.2 |
| AC079336.5|AL513320.1 |
| AC079336.5|AP000251.1 |
| AC079336.5|LINC01508 |
| AC079336.5|LINC01711 |
| AC079336.5|LINC01063 |
| AC079336.5|NRIR |
| AC079336.5|LINC02551 |
| AC079336.5|AC128709.1 |
| AC079336.5|AC068473.3 |
| AC079336.5|LINC02878 |
| AC079336.5|AC011481.1 |
| AC079336.5|LINC02446 |
| AC079336.5|LINC00944 |
| AC079336.5|TRPM2-AS |
| AC079336.5|AL592424.1 |
| AC079336.5|AC019080.4 |
| AC079336.5|LINC01405 |
| AC079336.5|AC004253.1 |
| AC079336.5|AL355472.3 |
| AC079336.5|AL391427.1 |
| AC079336.5|AL445250.1 |
| AC079336.5|HOXC-AS1 |
| AC079336.5|AC015660.2 |
| AC079336.5|AL445471.1 |
| AC079336.5|SOCAR |
| AC079336.5|DBH-AS1 |
| AC079336.5|AC025176.1 |
| AL049775.1|AC004704.1 |
| AL049775.1|SLC2A1-AS1 |
| AL049775.1|AC131159.1 |
| AL049775.1|MNX1-AS1 |
| AL049775.1|LINC02195 |
| AL049775.1|MIAT |
| AL049775.1|LHX1-DT |
| AL049775.1|LINC01694 |
| AL049775.1|AL590666.2 |
| AL049775.1|LINC01614 |
| AL049775.1|SLCO4A1-AS1 |
| AL049775.1|AC132192.2 |
| AL049775.1|AC011461.1 |
| AL049775.1|AC109460.2 |
| AL049775.1|LINC01139 |
| AL049775.1|AC024592.2 |
| AL049775.1|AL139089.1 |
| AL049775.1|LINC01322 |
| AL049775.1|AC026785.3 |
| AL049775.1|PCAT7 |
| AL049775.1|LINC00839 |
| AL049775.1|ELFN1-AS1 |
| AL049775.1|AC012065.3 |
| AL049775.1|LINC01305 |
| AL049775.1|LINC01943 |
| AL049775.1|AC011676.1 |
| AL049775.1|AC015878.1 |
| AL049775.1|AC009093.2 |
| AL049775.1|AC104041.1 |
| AL049775.1|AC008115.3 |
| AL049775.1|AC104564.3 |
| AL049775.1|AL136018.1 |
| AL049775.1|AL513327.1 |
| AL049775.1|CASC15 |
| AL049775.1|LINC01096 |
| AL049775.1|AL365181.3 |
| AL049775.1|SLC12A5-AS1 |
| AL049775.1|AC010719.1 |
| AL049775.1|Z97653.1 |
| AL049775.1|AC013731.1 |
| AL049775.1|C5orf66-AS1 |
| AL049775.1|NCBP2-AS1 |
| AL049775.1|AC093788.1 |
| AL049775.1|MCCC1-AS1 |
| AL049775.1|AC112236.1 |
| AL049775.1|TBILA |
| AL049775.1|LINC02561 |
| AL049775.1|AC025154.2 |
| AL049775.1|LINC02454 |
| AL049775.1|AC092171.4 |
| AL049775.1|AL158166.1 |
| AL049775.1|AL365181.2 |
| AL049775.1|STARD4-AS1 |
| AL049775.1|LINC01050 |
| AL049775.1|U62317.4 |
| AL049775.1|LINC02244 |
| AL049775.1|AC007497.1 |
| AL049775.1|AL122125.1 |
| AL049775.1|AC006206.2 |
| AL049775.1|AP002907.1 |
| AL049775.1|AC128709.2 |
| AL049775.1|AP005482.2 |
| AL049775.1|LINC01929 |
| AL049775.1|DLGAP1-AS2 |
| AL049775.1|ATP1B3-AS1 |
| AL049775.1|AF165147.1 |
| AL049775.1|MIR9-3HG |
| AL049775.1|AC053503.3 |
| AL049775.1|MIR1-1HG |
| AL049775.1|AC091563.1 |
| AL049775.1|AC010789.1 |
| AL049775.1|LINC02820 |
| AL049775.1|LINC02100 |
| AL049775.1|FOXD3-AS1 |
| AL049775.1|AC021028.1 |
| AL049775.1|AC026333.4 |
| AL049775.1|AL596442.2 |
| AL049775.1|LINC00707 |
| AL049775.1|AC019171.1 |
| AL049775.1|AC009093.1 |
| AL049775.1|AC010331.1 |
| AL049775.1|AC048341.2 |
| AL049775.1|AC016773.2 |
| AL049775.1|LINC01311 |
| AL049775.1|CDKN2B-AS1 |
| AL049775.1|AL031186.1 |
| AL049775.1|AC114730.3 |
| AL049775.1|LINC00911 |
| AL049775.1|AC016394.3 |
| AL049775.1|AL133215.2 |
| AL049775.1|AC004034.1 |
| AL049775.1|TTLL11-IT1 |
| AL049775.1|AC103563.7 |
| AL049775.1|AL355488.1 |
| AL049775.1|AC016877.3 |
| AL049775.1|AC145207.8 |
| AL049775.1|ZKSCAN2-DT |
| AL049775.1|AL499627.1 |
| AL049775.1|AL157871.2 |
| AL049775.1|AC020907.4 |
| AL049775.1|LINC01355 |
| AL049775.1|FOXP4-AS1 |
| AL049775.1|AP001029.1 |
| AL049775.1|TENM3-AS1 |
| AL049775.1|DDX11-AS1 |
| AL049775.1|ITGB1-DT |
| AL049775.1|AL022322.1 |
| AL049775.1|MAFA-AS1 |
| AL049775.1|AL355803.1 |
| AL049775.1|AC134312.5 |
| AL049775.1|MIR155HG |
| AL049775.1|AC016735.1 |
| AL049775.1|AC089983.1 |
| AL049775.1|YEATS2-AS1 |
| AL049775.1|AC018716.1 |
| AL049775.1|AC011462.4 |
| AL049775.1|AC010731.2 |
| AL049775.1|LINC00942 |
| AL049775.1|KDM4A-AS1 |
| AL049775.1|LINC02544 |
| AL049775.1|DELEC1 |
| AL049775.1|AC034213.1 |
| AL049775.1|AC092119.2 |
| AL049775.1|AL359881.1 |
| AL049775.1|AL031600.1 |
| AL049775.1|STEAP3-AS1 |
| AL049775.1|AL591501.1 |
| AL049775.1|SCAT2 |
| AL049775.1|HOTAIR |
| AL049775.1|HS1BP3-IT1 |
| AL049775.1|AC112721.2 |
| AL049775.1|AL513320.1 |
| AL049775.1|AP000251.1 |
| AL049775.1|LINC01508 |
| AL049775.1|C1RL-AS1 |
| AL049775.1|LINC01711 |
| AL049775.1|LINC01063 |
| AL049775.1|NRIR |
| AL049775.1|LINC02551 |
| AL049775.1|AC128709.1 |
| AL049775.1|AC068473.3 |
| AL049775.1|AL031670.1 |
| AL049775.1|LINC02878 |
| AL049775.1|AC011481.1 |
| AL049775.1|LINC02446 |
| AL049775.1|AC104031.1 |
| AL049775.1|LINC00944 |
| AL049775.1|TRPM2-AS |
| AL049775.1|AL592424.1 |
| AL049775.1|AC019080.4 |
| AL049775.1|LINC02870 |
| AL049775.1|LINC01405 |
| AL049775.1|AC027796.4 |
| AL049775.1|LINC01269 |
| AL049775.1|AC004253.1 |
| AL049775.1|AL355472.3 |
| AL049775.1|TYMSOS |
| AL049775.1|AL391427.1 |
| AL049775.1|AL445250.1 |
| AL049775.1|HOXC-AS1 |
| AL049775.1|AC015660.2 |
| AL049775.1|AL445471.1 |
| AL049775.1|SOCAR |
| AL049775.1|DBH-AS1 |
| AL049775.1|AC025176.1 |
| AC004704.1|AC131159.1 |
| AC004704.1|MNX1-AS1 |
| AC004704.1|LHX1-DT |
| AC004704.1|LINC01694 |
| AC004704.1|SLCO4A1-AS1 |
| AC004704.1|AC011461.1 |
| AC004704.1|AC024592.2 |
| AC004704.1|LINC01322 |
| AC004704.1|AC026785.3 |
| AC004704.1|ELFN1-AS1 |
| AC004704.1|AC012065.3 |
| AC004704.1|LINC01305 |
| AC004704.1|AC011676.1 |
| AC004704.1|AC015878.1 |
| AC004704.1|AC009093.2 |
| AC004704.1|AC104041.1 |
| AC004704.1|AC104564.3 |
| AC004704.1|LINC01096 |
| AC004704.1|SLC12A5-AS1 |
| AC004704.1|AC010719.1 |
| AC004704.1|Z97653.1 |
| AC004704.1|AL137246.2 |
| AC004704.1|AC013731.1 |
| AC004704.1|NCBP2-AS1 |
| AC004704.1|AC093788.1 |
| AC004704.1|MCCC1-AS1 |
| AC004704.1|AC112236.1 |
| AC004704.1|LINC02561 |
| AC004704.1|AC025154.2 |
| AC004704.1|LINC02454 |
| AC004704.1|AC092171.4 |
| AC004704.1|AL158166.1 |
| AC004704.1|AL365181.2 |
| AC004704.1|LINC01050 |
| AC004704.1|LINC02244 |
| AC004704.1|AC007497.1 |
| AC004704.1|AL122125.1 |
| AC004704.1|AP002907.1 |
| AC004704.1|AC128709.2 |
| AC004704.1|AP005482.2 |
| AC004704.1|LINC01929 |
| AC004704.1|AF165147.1 |
| AC004704.1|AC053503.3 |
| AC004704.1|MIR1-1HG |
| AC004704.1|AC010789.1 |
| AC004704.1|LINC02820 |
| AC004704.1|AC026333.4 |
| AC004704.1|AL596442.2 |
| AC004704.1|AC019171.1 |
| AC004704.1|AC009093.1 |
| AC004704.1|AC010331.1 |
| AC004704.1|CDKN2B-AS1 |
| AC004704.1|AC114730.3 |
| AC004704.1|LINC00911 |
| AC004704.1|AC004034.1 |
| AC004704.1|TTLL11-IT1 |
| AC004704.1|AC103563.7 |
| AC004704.1|AC016877.3 |
| AC004704.1|AC145207.8 |
| AC004704.1|C10orf71-AS1 |
| AC004704.1|ZKSCAN2-DT |
| AC004704.1|AL499627.1 |
| AC004704.1|AL157871.2 |
| AC004704.1|AC020907.4 |
| AC004704.1|FOXP4-AS1 |
| AC004704.1|AP001029.1 |
| AC004704.1|TENM3-AS1 |
| AC004704.1|DDX11-AS1 |
| AC004704.1|ITGB1-DT |
| AC004704.1|AL022322.1 |
| AC004704.1|MAFA-AS1 |
| AC004704.1|AL355803.1 |
| AC004704.1|AC016735.1 |
| AC004704.1|AC089983.1 |
| AC004704.1|YEATS2-AS1 |
| AC004704.1|AC018716.1 |
| AC004704.1|AC011462.4 |
| AC004704.1|AC010731.2 |
| AC004704.1|LINC00942 |
| AC004704.1|DELEC1 |
| AC004704.1|AC034213.1 |
| AC004704.1|AC092119.2 |
| AC004704.1|AL359881.1 |
| AC004704.1|STEAP3-AS1 |
| AC004704.1|AL591501.1 |
| AC004704.1|SCAT2 |
| AC004704.1|HOTAIR |
| AC004704.1|HS1BP3-IT1 |
| AC004704.1|AC112721.2 |
| AC004704.1|AL513320.1 |
| AC004704.1|LINC01508 |
| AC004704.1|LINC01711 |
| AC004704.1|NRIR |
| AC004704.1|LINC02551 |
| AC004704.1|AC128709.1 |
| AC004704.1|AC068473.3 |
| AC004704.1|AC011481.1 |
| AC004704.1|AL592424.1 |
| AC004704.1|AC019080.4 |
| AC004704.1|LINC01405 |
| AC004704.1|AC004253.1 |
| AC004704.1|AL355472.3 |
| AC004704.1|MIR133A1HG |
| AC004704.1|AL391427.1 |
| AC004704.1|AL445250.1 |
| AC004704.1|HOXC-AS1 |
| AC004704.1|AC015660.2 |
| AC004704.1|AL445471.1 |
| AC004704.1|SOCAR |
| AC004704.1|DBH-AS1 |
| AC004704.1|AC025176.1 |
| SLC2A1-AS1|AC131159.1 |
| SLC2A1-AS1|MNX1-AS1 |
| SLC2A1-AS1|LINC02195 |
| SLC2A1-AS1|MIAT |
| SLC2A1-AS1|LHX1-DT |
| SLC2A1-AS1|AL590666.2 |
| SLC2A1-AS1|LINC01614 |
| SLC2A1-AS1|SLCO4A1-AS1 |
| SLC2A1-AS1|AC132192.2 |
| SLC2A1-AS1|AC011461.1 |
| SLC2A1-AS1|AC109460.2 |
| SLC2A1-AS1|LINC01139 |
| SLC2A1-AS1|AC024592.2 |
| SLC2A1-AS1|AL139089.1 |
| SLC2A1-AS1|LINC01322 |
| SLC2A1-AS1|PCAT7 |
| SLC2A1-AS1|LINC00839 |
| SLC2A1-AS1|ELFN1-AS1 |
| SLC2A1-AS1|AC012065.3 |
| SLC2A1-AS1|LINC01943 |
| SLC2A1-AS1|AC011676.1 |
| SLC2A1-AS1|AC009093.2 |
| SLC2A1-AS1|AC104041.1 |
| SLC2A1-AS1|AC008735.2 |
| SLC2A1-AS1|AC002401.4 |
| SLC2A1-AS1|AC008115.3 |
| SLC2A1-AS1|AC104564.3 |
| SLC2A1-AS1|AL136018.1 |
| SLC2A1-AS1|AL513327.1 |
| SLC2A1-AS1|CASC15 |
| SLC2A1-AS1|KCNMB2-AS1 |
| SLC2A1-AS1|LINC01096 |
| SLC2A1-AS1|AL365181.3 |
| SLC2A1-AS1|SLC12A5-AS1 |
| SLC2A1-AS1|AC010719.1 |
| SLC2A1-AS1|Z97653.1 |
| SLC2A1-AS1|AC013731.1 |
| SLC2A1-AS1|C5orf66-AS1 |
| SLC2A1-AS1|NCBP2-AS1 |
| SLC2A1-AS1|AC093788.1 |
| SLC2A1-AS1|MCCC1-AS1 |
| SLC2A1-AS1|AC112236.1 |
| SLC2A1-AS1|TBILA |
| SLC2A1-AS1|LINC02561 |
| SLC2A1-AS1|LINC02454 |
| SLC2A1-AS1|AC092171.4 |
| SLC2A1-AS1|AL158166.1 |
| SLC2A1-AS1|AL365181.2 |
| SLC2A1-AS1|STARD4-AS1 |
| SLC2A1-AS1|LINC01050 |
| SLC2A1-AS1|U62317.4 |
| SLC2A1-AS1|LINC02244 |
| SLC2A1-AS1|AC007497.1 |
| SLC2A1-AS1|AL122125.1 |
| SLC2A1-AS1|AC006206.2 |
| SLC2A1-AS1|AP002907.1 |
| SLC2A1-AS1|AC128709.2 |
| SLC2A1-AS1|AP005482.2 |
| SLC2A1-AS1|LINC01929 |
| SLC2A1-AS1|DLGAP1-AS2 |
| SLC2A1-AS1|ATP1B3-AS1 |
| SLC2A1-AS1|AF165147.1 |
| SLC2A1-AS1|MIR9-3HG |
| SLC2A1-AS1|MIR1-1HG |
| SLC2A1-AS1|AC091563.1 |
| SLC2A1-AS1|AL021807.1 |
| SLC2A1-AS1|AC010789.1 |
| SLC2A1-AS1|LINC02820 |
| SLC2A1-AS1|LINC02100 |
| SLC2A1-AS1|FOXD3-AS1 |
| SLC2A1-AS1|AC021028.1 |
| SLC2A1-AS1|AC026333.4 |
| SLC2A1-AS1|LINC00707 |
| SLC2A1-AS1|AC114956.1 |
| SLC2A1-AS1|AC019171.1 |
| SLC2A1-AS1|AC009093.1 |
| SLC2A1-AS1|AC010331.1 |
| SLC2A1-AS1|AC048341.2 |
| SLC2A1-AS1|AC016773.2 |
| SLC2A1-AS1|LINC01311 |
| SLC2A1-AS1|CDKN2B-AS1 |
| SLC2A1-AS1|AL031186.1 |
| SLC2A1-AS1|AC114730.3 |
| SLC2A1-AS1|LINC00911 |
| SLC2A1-AS1|AC016394.3 |
| SLC2A1-AS1|AL133215.2 |
| SLC2A1-AS1|AC004034.1 |
| SLC2A1-AS1|AC055822.1 |
| SLC2A1-AS1|TTLL11-IT1 |
| SLC2A1-AS1|AC103563.7 |
| SLC2A1-AS1|AL355488.1 |
| SLC2A1-AS1|AC016877.3 |
| SLC2A1-AS1|AC145207.8 |
| SLC2A1-AS1|ZKSCAN2-DT |
| SLC2A1-AS1|AL157871.2 |
| SLC2A1-AS1|AC020907.4 |
| SLC2A1-AS1|LINC01355 |
| SLC2A1-AS1|FOXP4-AS1 |
| SLC2A1-AS1|AP001029.1 |
| SLC2A1-AS1|TENM3-AS1 |
| SLC2A1-AS1|DDX11-AS1 |
| SLC2A1-AS1|ITGB1-DT |
| SLC2A1-AS1|AL022322.1 |
| SLC2A1-AS1|MAFA-AS1 |
| SLC2A1-AS1|AL355803.1 |
| SLC2A1-AS1|AC134312.5 |
| SLC2A1-AS1|MIR155HG |
| SLC2A1-AS1|AC016735.1 |
| SLC2A1-AS1|AL928654.2 |
| SLC2A1-AS1|YEATS2-AS1 |
| SLC2A1-AS1|AC018716.1 |
| SLC2A1-AS1|AC011462.4 |
| SLC2A1-AS1|LINC00942 |
| SLC2A1-AS1|KDM4A-AS1 |
| SLC2A1-AS1|LINC02544 |
| SLC2A1-AS1|LINC00941 |
| SLC2A1-AS1|DELEC1 |
| SLC2A1-AS1|AC034213.1 |
| SLC2A1-AS1|AC092119.2 |
| SLC2A1-AS1|AL359881.1 |
| SLC2A1-AS1|AL031600.1 |
| SLC2A1-AS1|STEAP3-AS1 |
| SLC2A1-AS1|SCAT2 |
| SLC2A1-AS1|HS1BP3-IT1 |
| SLC2A1-AS1|AC112721.2 |
| SLC2A1-AS1|AL513320.1 |
| SLC2A1-AS1|AP000251.1 |
| SLC2A1-AS1|C1RL-AS1 |
| SLC2A1-AS1|LINC01711 |
| SLC2A1-AS1|LINC01063 |
| SLC2A1-AS1|NRIR |
| SLC2A1-AS1|LINC02551 |
| SLC2A1-AS1|NKILA |
| SLC2A1-AS1|AC005392.2 |
| SLC2A1-AS1|AC128709.1 |
| SLC2A1-AS1|MSC-AS1 |
| SLC2A1-AS1|AL031670.1 |
| SLC2A1-AS1|LINC02878 |
| SLC2A1-AS1|AC011481.1 |
| SLC2A1-AS1|LINC02446 |
| SLC2A1-AS1|AC104031.1 |
| SLC2A1-AS1|LINC00944 |
| SLC2A1-AS1|TRPM2-AS |
| SLC2A1-AS1|AL592424.1 |
| SLC2A1-AS1|AC019080.4 |
| SLC2A1-AS1|LINC02870 |
| SLC2A1-AS1|LINC01405 |
| SLC2A1-AS1|AC027796.4 |
| SLC2A1-AS1|LINC01269 |
| SLC2A1-AS1|AC004253.1 |
| SLC2A1-AS1|AL355472.3 |
| SLC2A1-AS1|AL391427.1 |
| SLC2A1-AS1|AL445250.1 |
| SLC2A1-AS1|HOXC-AS1 |
| SLC2A1-AS1|AC015660.2 |
| SLC2A1-AS1|AL445471.1 |
| SLC2A1-AS1|SOCAR |
| SLC2A1-AS1|DBH-AS1 |
| SLC2A1-AS1|AC025176.1 |
| AC131159.1|MNX1-AS1 |
| AC131159.1|MIAT |
| AC131159.1|LHX1-DT |
| AC131159.1|LINC01694 |
| AC131159.1|LINC01614 |
| AC131159.1|SLCO4A1-AS1 |
| AC131159.1|AC011461.1 |
| AC131159.1|AC109460.2 |
| AC131159.1|LINC01139 |
| AC131159.1|AC024592.2 |
| AC131159.1|AL139089.1 |
| AC131159.1|LINC01322 |
| AC131159.1|AC026785.3 |
| AC131159.1|PCAT7 |
| AC131159.1|ELFN1-AS1 |
| AC131159.1|AC012065.3 |
| AC131159.1|LINC01305 |
| AC131159.1|LINC01943 |
| AC131159.1|AC011676.1 |
| AC131159.1|AC015878.1 |
| AC131159.1|AC009093.2 |
| AC131159.1|AC104041.1 |
| AC131159.1|AC002401.4 |
| AC131159.1|AC104564.3 |
| AC131159.1|AL136018.1 |
| AC131159.1|AL513327.1 |
| AC131159.1|CASC15 |
| AC131159.1|LINC01096 |
| AC131159.1|SLC12A5-AS1 |
| AC131159.1|AC010719.1 |
| AC131159.1|Z97653.1 |
| AC131159.1|AL137246.2 |
| AC131159.1|AC013731.1 |
| AC131159.1|NCBP2-AS1 |
| AC131159.1|AC093788.1 |
| AC131159.1|MCCC1-AS1 |
| AC131159.1|AC112236.1 |
| AC131159.1|LINC02561 |
| AC131159.1|AC025154.2 |
| AC131159.1|LINC02454 |
| AC131159.1|AC092171.4 |
| AC131159.1|AL158166.1 |
| AC131159.1|AL365181.2 |
| AC131159.1|STARD4-AS1 |
| AC131159.1|LINC01050 |
| AC131159.1|LINC02244 |
| AC131159.1|AC007497.1 |
| AC131159.1|AL122125.1 |
| AC131159.1|AP002907.1 |
| AC131159.1|AC128709.2 |
| AC131159.1|AP005482.2 |
| AC131159.1|LINC01929 |
| AC131159.1|AF165147.1 |
| AC131159.1|MIR9-3HG |
| AC131159.1|AC053503.3 |
| AC131159.1|MIR1-1HG |
| AC131159.1|AC010789.1 |
| AC131159.1|LINC02820 |
| AC131159.1|LINC02100 |
| AC131159.1|FOXD3-AS1 |
| AC131159.1|AC026333.4 |
| AC131159.1|AL596442.2 |
| AC131159.1|LINC00707 |
| AC131159.1|AC019171.1 |
| AC131159.1|AC009093.1 |
| AC131159.1|AC010331.1 |
| AC131159.1|AC016773.2 |
| AC131159.1|LINC01311 |
| AC131159.1|CDKN2B-AS1 |
| AC131159.1|AC114730.3 |
| AC131159.1|LINC00911 |
| AC131159.1|AC004034.1 |
| AC131159.1|TTLL11-IT1 |
| AC131159.1|AC103563.7 |
| AC131159.1|AC016877.3 |
| AC131159.1|AC145207.8 |
| AC131159.1|C10orf71-AS1 |
| AC131159.1|ZKSCAN2-DT |
| AC131159.1|AL499627.1 |
| AC131159.1|AL157871.2 |
| AC131159.1|AC020907.4 |
| AC131159.1|LINC01355 |
| AC131159.1|FOXP4-AS1 |
| AC131159.1|AP001029.1 |
| AC131159.1|TENM3-AS1 |
| AC131159.1|DDX11-AS1 |
| AC131159.1|ITGB1-DT |
| AC131159.1|AL022322.1 |
| AC131159.1|MAFA-AS1 |
| AC131159.1|AL355803.1 |
| AC131159.1|AC134312.5 |
| AC131159.1|MIR155HG |
| AC131159.1|AC016735.1 |
| AC131159.1|AC089983.1 |
| AC131159.1|YEATS2-AS1 |
| AC131159.1|AC018716.1 |
| AC131159.1|AC011462.4 |
| AC131159.1|AC010731.2 |
| AC131159.1|LINC00942 |
| AC131159.1|LINC02544 |
| AC131159.1|LINC00941 |
| AC131159.1|DELEC1 |
| AC131159.1|AC034213.1 |
| AC131159.1|AC092119.2 |
| AC131159.1|AL359881.1 |
| AC131159.1|AL031600.1 |
| AC131159.1|STEAP3-AS1 |
| AC131159.1|AL591501.1 |
| AC131159.1|SCAT2 |
| AC131159.1|HOTAIR |
| AC131159.1|HS1BP3-IT1 |
| AC131159.1|AC112721.2 |
| AC131159.1|AL513320.1 |
| AC131159.1|LINC01508 |
| AC131159.1|LINC01711 |
| AC131159.1|LINC01063 |
| AC131159.1|NRIR |
| AC131159.1|LINC02551 |
| AC131159.1|AC128709.1 |
| AC131159.1|AC068473.3 |
| AC131159.1|AL031670.1 |
| AC131159.1|LINC02878 |
| AC131159.1|AC011481.1 |
| AC131159.1|LINC02446 |
| AC131159.1|AC104031.1 |
| AC131159.1|LINC00944 |
| AC131159.1|TRPM2-AS |
| AC131159.1|AL592424.1 |
| AC131159.1|AC019080.4 |
| AC131159.1|LINC01405 |
| AC131159.1|AC004253.1 |
| AC131159.1|AL355472.3 |
| AC131159.1|MIR133A1HG |
| AC131159.1|AL391427.1 |
| AC131159.1|AL445250.1 |
| AC131159.1|HOXC-AS1 |
| AC131159.1|AC015660.2 |
| AC131159.1|AL445471.1 |
| AC131159.1|SOCAR |
| AC131159.1|DBH-AS1 |
| AC131159.1|AC025176.1 |
| MNX1-AS1|LHX1-DT |
| MNX1-AS1|LINC01694 |
| MNX1-AS1|LINC01614 |
| MNX1-AS1|SLCO4A1-AS1 |
| MNX1-AS1|AC011461.1 |
| MNX1-AS1|AC109460.2 |
| MNX1-AS1|LINC01139 |
| MNX1-AS1|AC024592.2 |
| MNX1-AS1|AL139089.1 |
| MNX1-AS1|LINC01322 |
| MNX1-AS1|AC026785.3 |
| MNX1-AS1|ELFN1-AS1 |
| MNX1-AS1|AC012065.3 |
| MNX1-AS1|LINC01305 |
| MNX1-AS1|LINC01943 |
| MNX1-AS1|AC011676.1 |
| MNX1-AS1|AC015878.1 |
| MNX1-AS1|AC009093.2 |
| MNX1-AS1|AC104041.1 |
| MNX1-AS1|AC002401.4 |
| MNX1-AS1|AC008115.3 |
| MNX1-AS1|AC104564.3 |
| MNX1-AS1|AL136018.1 |
| MNX1-AS1|AL513327.1 |
| MNX1-AS1|CASC15 |
| MNX1-AS1|LINC01096 |
| MNX1-AS1|SLC12A5-AS1 |
| MNX1-AS1|AC010719.1 |
| MNX1-AS1|Z97653.1 |
| MNX1-AS1|AL137246.2 |
| MNX1-AS1|AC013731.1 |
| MNX1-AS1|NCBP2-AS1 |
| MNX1-AS1|AC093788.1 |
| MNX1-AS1|MCCC1-AS1 |
| MNX1-AS1|AC112236.1 |
| MNX1-AS1|LINC02561 |
| MNX1-AS1|AC025154.2 |
| MNX1-AS1|LINC02454 |
| MNX1-AS1|AC092171.4 |
| MNX1-AS1|AL158166.1 |
| MNX1-AS1|AL365181.2 |
| MNX1-AS1|STARD4-AS1 |
| MNX1-AS1|LINC01050 |
| MNX1-AS1|LINC02244 |
| MNX1-AS1|AC007497.1 |
| MNX1-AS1|AL122125.1 |
| MNX1-AS1|AP002907.1 |
| MNX1-AS1|AC128709.2 |
| MNX1-AS1|AP005482.2 |
| MNX1-AS1|LINC01929 |
| MNX1-AS1|AF165147.1 |
| MNX1-AS1|MIR9-3HG |
| MNX1-AS1|AC053503.3 |
| MNX1-AS1|MIR1-1HG |
| MNX1-AS1|AC010789.1 |
| MNX1-AS1|LINC02820 |
| MNX1-AS1|LINC02100 |
| MNX1-AS1|FOXD3-AS1 |
| MNX1-AS1|AC021028.1 |
| MNX1-AS1|AC026333.4 |
| MNX1-AS1|AL596442.2 |
| MNX1-AS1|LINC00707 |
| MNX1-AS1|AC019171.1 |
| MNX1-AS1|AC009093.1 |
| MNX1-AS1|AC010331.1 |
| MNX1-AS1|AC016773.2 |
| MNX1-AS1|LINC01311 |
| MNX1-AS1|CDKN2B-AS1 |
| MNX1-AS1|AL031186.1 |
| MNX1-AS1|AC114730.3 |
| MNX1-AS1|LINC00911 |
| MNX1-AS1|AL133215.2 |
| MNX1-AS1|AC004034.1 |
| MNX1-AS1|TTLL11-IT1 |
| MNX1-AS1|AC103563.7 |
| MNX1-AS1|AC016877.3 |
| MNX1-AS1|AC145207.8 |
| MNX1-AS1|C10orf71-AS1 |
| MNX1-AS1|ZKSCAN2-DT |
| MNX1-AS1|AL499627.1 |
| MNX1-AS1|AL157871.2 |
| MNX1-AS1|AC020907.4 |
| MNX1-AS1|LINC01355 |
| MNX1-AS1|FOXP4-AS1 |
| MNX1-AS1|AP001029.1 |
| MNX1-AS1|TENM3-AS1 |
| MNX1-AS1|DDX11-AS1 |
| MNX1-AS1|ITGB1-DT |
| MNX1-AS1|AL022322.1 |
| MNX1-AS1|MAFA-AS1 |
| MNX1-AS1|AL355803.1 |
| MNX1-AS1|AC134312.5 |
| MNX1-AS1|MIR155HG |
| MNX1-AS1|AC016735.1 |
| MNX1-AS1|AC089983.1 |
| MNX1-AS1|YEATS2-AS1 |
| MNX1-AS1|AC018716.1 |
| MNX1-AS1|AC011462.4 |
| MNX1-AS1|AC010731.2 |
| MNX1-AS1|LINC00942 |
| MNX1-AS1|LINC02544 |
| MNX1-AS1|DELEC1 |
| MNX1-AS1|AC034213.1 |
| MNX1-AS1|AC092119.2 |
| MNX1-AS1|AL359881.1 |
| MNX1-AS1|AL031600.1 |
| MNX1-AS1|STEAP3-AS1 |
| MNX1-AS1|AL591501.1 |
| MNX1-AS1|SCAT2 |
| MNX1-AS1|HOTAIR |
| MNX1-AS1|HS1BP3-IT1 |
| MNX1-AS1|AC112721.2 |
| MNX1-AS1|AL513320.1 |
| MNX1-AS1|AP000251.1 |
| MNX1-AS1|LINC01508 |
| MNX1-AS1|LINC01711 |
| MNX1-AS1|LINC01063 |
| MNX1-AS1|NRIR |
| MNX1-AS1|LINC02551 |
| MNX1-AS1|AC128709.1 |
| MNX1-AS1|AC068473.3 |
| MNX1-AS1|AL031670.1 |
| MNX1-AS1|LINC02878 |
| MNX1-AS1|AC011481.1 |
| MNX1-AS1|LINC02446 |
| MNX1-AS1|LINC00944 |
| MNX1-AS1|TRPM2-AS |
| MNX1-AS1|AL592424.1 |
| MNX1-AS1|AC019080.4 |
| MNX1-AS1|LINC01405 |
| MNX1-AS1|LINC01269 |
| MNX1-AS1|AC004253.1 |
| MNX1-AS1|AL355472.3 |
| MNX1-AS1|MIR133A1HG |
| MNX1-AS1|AL391427.1 |
| MNX1-AS1|AL445250.1 |
| MNX1-AS1|HOXC-AS1 |
| MNX1-AS1|AC015660.2 |
| MNX1-AS1|AL445471.1 |
| MNX1-AS1|SOCAR |
| MNX1-AS1|DBH-AS1 |
| MNX1-AS1|AC025176.1 |
| AL512274.1|LINC01614 |
| AL512274.1|MELTF-AS1 |
| AL512274.1|LINC00839 |
| AL512274.1|KCNMB2-AS1 |
| AL512274.1|AL365181.3 |
| AL512274.1|C5orf66-AS1 |
| AL512274.1|NCK1-DT |
| AL512274.1|C5orf34-AS1 |
| AL512274.1|LINC02560 |
| AL512274.1|AL132712.1 |
| AL512274.1|AC005392.2 |
| AL512274.1|AC099850.4 |
| AL512274.1|LINC00519 |
| LINC02195|MIAT |
| LINC02195|AL590666.2 |
| LINC02195|LINC01614 |
| LINC02195|SLCO4A1-AS1 |
| LINC02195|AC132192.2 |
| LINC02195|FOXD2-AS1 |
| LINC02195|MELTF-AS1 |
| LINC02195|AC109460.2 |
| LINC02195|LINC01139 |
| LINC02195|AL139089.1 |
| LINC02195|LINC01322 |
| LINC02195|PCAT7 |
| LINC02195|LINC00839 |
| LINC02195|ELFN1-AS1 |
| LINC02195|AC012065.3 |
| LINC02195|LINC01943 |
| LINC02195|AC104041.1 |
| LINC02195|AC008735.2 |
| LINC02195|AC002401.4 |
| LINC02195|AC008115.3 |
| LINC02195|AL136018.1 |
| LINC02195|CASC15 |
| LINC02195|KCNMB2-AS1 |
| LINC02195|AL365181.3 |
| LINC02195|AC010719.1 |
| LINC02195|C5orf66-AS1 |
| LINC02195|AC093788.1 |
| LINC02195|AC026740.1 |
| LINC02195|AC112236.1 |
| LINC02195|TBILA |
| LINC02195|LINC02561 |
| LINC02195|LINC02454 |
| LINC02195|AL158166.1 |
| LINC02195|AL365181.2 |
| LINC02195|U62317.4 |
| LINC02195|AC006206.2 |
| LINC02195|AC128709.2 |
| LINC02195|AP005482.2 |
| LINC02195|LINC01929 |
| LINC02195|DLGAP1-AS2 |
| LINC02195|ATP1B3-AS1 |
| LINC02195|MIR9-3HG |
| LINC02195|AC091563.1 |
| LINC02195|AL021807.1 |
| LINC02195|AC010789.1 |
| LINC02195|LINC02820 |
| LINC02195|LINC02100 |
| LINC02195|AL035461.3 |
| LINC02195|FOXD3-AS1 |
| LINC02195|AC021028.1 |
| LINC02195|ZNF710-AS1 |
| LINC02195|LINC00707 |
| LINC02195|AC114956.1 |
| LINC02195|AC009093.1 |
| LINC02195|AC048341.2 |
| LINC02195|AC016773.2 |
| LINC02195|LINC01311 |
| LINC02195|AL031186.1 |
| LINC02195|AC016394.3 |
| LINC02195|AL133215.2 |
| LINC02195|AC004034.1 |
| LINC02195|AC055822.1 |
| LINC02195|C5orf34-AS1 |
| LINC02195|TTLL11-IT1 |
| LINC02195|AL355488.1 |
| LINC02195|AC016877.3 |
| LINC02195|AC145207.8 |
| LINC02195|LINC01355 |
| LINC02195|TENM3-AS1 |
| LINC02195|AL022322.1 |
| LINC02195|MAFA-AS1 |
| LINC02195|AL355803.1 |
| LINC02195|AC134312.5 |
| LINC02195|MIR155HG |
| LINC02195|AL928654.2 |
| LINC02195|LINC00942 |
| LINC02195|KDM4A-AS1 |
| LINC02195|LINC02544 |
| LINC02195|LINC00941 |
| LINC02195|DELEC1 |
| LINC02195|AL132712.1 |
| LINC02195|AL031600.1 |
| LINC02195|SCAT2 |
| LINC02195|HS1BP3-IT1 |
| LINC02195|AP000251.1 |
| LINC02195|C1RL-AS1 |
| LINC02195|LINC01711 |
| LINC02195|LINC01063 |
| LINC02195|TMPO-AS1 |
| LINC02195|LINC02551 |
| LINC02195|NKILA |
| LINC02195|AC005392.2 |
| LINC02195|MSC-AS1 |
| LINC02195|AL031670.1 |
| LINC02195|LINC02446 |
| LINC02195|AC104031.1 |
| LINC02195|LINC00944 |
| LINC02195|TRPM2-AS |
| LINC02195|AC019080.4 |
| LINC02195|LINC02870 |
| LINC02195|AC027796.4 |
| LINC02195|LINC01269 |
| LINC02195|LINC00519 |
| LINC02195|AC004253.1 |
| LINC02195|TYMSOS |
| LINC02195|AL391427.1 |
| LINC02195|HOXC-AS1 |
| LINC02195|AC015660.2 |
| LINC02195|AC012073.1 |
| MIAT|LHX1-DT |
| MIAT|AL590666.2 |
| MIAT|LINC01614 |
| MIAT|SLCO4A1-AS1 |
| MIAT|AC132192.2 |
| MIAT|FOXD2-AS1 |
| MIAT|MELTF-AS1 |
| MIAT|AC011461.1 |
| MIAT|AC109460.2 |
| MIAT|LINC01139 |
| MIAT|AC024592.2 |
| MIAT|AL139089.1 |
| MIAT|LINC01322 |
| MIAT|PCAT7 |
| MIAT|LINC00839 |
| MIAT|ELFN1-AS1 |
| MIAT|AC012065.3 |
| MIAT|LINC01943 |
| MIAT|AC011676.1 |
| MIAT|AC104041.1 |
| MIAT|AC008735.2 |
| MIAT|AC002401.4 |
| MIAT|AC008115.3 |
| MIAT|AL136018.1 |
| MIAT|AL513327.1 |
| MIAT|CASC15 |
| MIAT|KCNMB2-AS1 |
| MIAT|AL365181.3 |
| MIAT|AC010719.1 |
| MIAT|Z97653.1 |
| MIAT|C5orf66-AS1 |
| MIAT|AC093788.1 |
| MIAT|AC026740.1 |
| MIAT|AC112236.1 |
| MIAT|TBILA |
| MIAT|LINC02561 |
| MIAT|LINC02454 |
| MIAT|AC092171.4 |
| MIAT|AL158166.1 |
| MIAT|AL365181.2 |
| MIAT|STARD4-AS1 |
| MIAT|U62317.4 |
| MIAT|AC007497.1 |
| MIAT|AL122125.1 |
| MIAT|AC006206.2 |
| MIAT|AP002907.1 |
| MIAT|AC128709.2 |
| MIAT|AP005482.2 |
| MIAT|LINC01929 |
| MIAT|DLGAP1-AS2 |
| MIAT|ATP1B3-AS1 |
| MIAT|AF165147.1 |
| MIAT|MIR9-3HG |
| MIAT|AC091563.1 |
| MIAT|AL021807.1 |
| MIAT|AC010789.1 |
| MIAT|LINC02820 |
| MIAT|LINC02100 |
| MIAT|FOXD3-AS1 |
| MIAT|AC021028.1 |
| MIAT|ZNF710-AS1 |
| MIAT|AC026333.4 |
| MIAT|AL596442.2 |
| MIAT|LINC00707 |
| MIAT|AC114956.1 |
| MIAT|AC009093.1 |
| MIAT|AC010331.1 |
| MIAT|AC048341.2 |
| MIAT|AC016773.2 |
| MIAT|LINC01311 |
| MIAT|CDKN2B-AS1 |
| MIAT|AL031186.1 |
| MIAT|LINC00911 |
| MIAT|AC016394.3 |
| MIAT|AL133215.2 |
| MIAT|AC004034.1 |
| MIAT|AC055822.1 |
| MIAT|TTLL11-IT1 |
| MIAT|AL355488.1 |
| MIAT|AC016877.3 |
| MIAT|AC145207.8 |
| MIAT|ZKSCAN2-DT |
| MIAT|AL157871.2 |
| MIAT|LINC01355 |
| MIAT|TENM3-AS1 |
| MIAT|DDX11-AS1 |
| MIAT|ITGB1-DT |
| MIAT|AL022322.1 |
| MIAT|MAFA-AS1 |
| MIAT|AL355803.1 |
| MIAT|AC134312.5 |
| MIAT|MIR155HG |
| MIAT|AC016735.1 |
| MIAT|AL928654.2 |
| MIAT|YEATS2-AS1 |
| MIAT|AC018716.1 |
| MIAT|AC011462.4 |
| MIAT|LINC00942 |
| MIAT|KDM4A-AS1 |
| MIAT|LINC02544 |
| MIAT|LINC00941 |
| MIAT|DELEC1 |
| MIAT|AC034213.1 |
| MIAT|AL031600.1 |
| MIAT|STEAP3-AS1 |
| MIAT|SCAT2 |
| MIAT|HS1BP3-IT1 |
| MIAT|AC112721.2 |
| MIAT|AP000251.1 |
| MIAT|C1RL-AS1 |
| MIAT|LINC01711 |
| MIAT|LINC01063 |
| MIAT|TMPO-AS1 |
| MIAT|NRIR |
| MIAT|LINC02551 |
| MIAT|NKILA |
| MIAT|AC005392.2 |
| MIAT|AC128709.1 |
| MIAT|MSC-AS1 |
| MIAT|AL031670.1 |
| MIAT|LINC02878 |
| MIAT|LINC02446 |
| MIAT|AC104031.1 |
| MIAT|LINC00944 |
| MIAT|TRPM2-AS |
| MIAT|AC019080.4 |
| MIAT|LINC02870 |
| MIAT|LINC01405 |
| MIAT|AC027796.4 |
| MIAT|LINC01269 |
| MIAT|AC004253.1 |
| MIAT|AL355472.3 |
| MIAT|TYMSOS |
| MIAT|AL391427.1 |
| MIAT|HOXC-AS1 |
| MIAT|AC015660.2 |
| MIAT|SOCAR |
| MIAT|DBH-AS1 |
| MIAT|AC025176.1 |
| LHX1-DT|LINC01694 |
| LHX1-DT|AL590666.2 |
| LHX1-DT|LINC01614 |
| LHX1-DT|SLCO4A1-AS1 |
| LHX1-DT|AC132192.2 |
| LHX1-DT|AC011461.1 |
| LHX1-DT|AC109460.2 |
| LHX1-DT|LINC01139 |
| LHX1-DT|AC024592.2 |
| LHX1-DT|AL139089.1 |
| LHX1-DT|LINC01322 |
| LHX1-DT|AC026785.3 |
| LHX1-DT|PCAT7 |
| LHX1-DT|LINC00839 |
| LHX1-DT|ELFN1-AS1 |
| LHX1-DT|AC012065.3 |
| LHX1-DT|LINC01305 |
| LHX1-DT|LINC01943 |
| LHX1-DT|AC011676.1 |
| LHX1-DT|AC015878.1 |
| LHX1-DT|AC009093.2 |
| LHX1-DT|AC104041.1 |
| LHX1-DT|AC008115.3 |
| LHX1-DT|AC104564.3 |
| LHX1-DT|AL136018.1 |
| LHX1-DT|AL513327.1 |
| LHX1-DT|CASC15 |
| LHX1-DT|LINC01096 |
| LHX1-DT|AL365181.3 |
| LHX1-DT|SLC12A5-AS1 |
| LHX1-DT|AC010719.1 |
| LHX1-DT|Z97653.1 |
| LHX1-DT|AL137246.2 |
| LHX1-DT|AC013731.1 |
| LHX1-DT|C5orf66-AS1 |
| LHX1-DT|NCBP2-AS1 |
| LHX1-DT|AC093788.1 |
| LHX1-DT|MCCC1-AS1 |
| LHX1-DT|AC112236.1 |
| LHX1-DT|TBILA |
| LHX1-DT|LINC02561 |
| LHX1-DT|AC025154.2 |
| LHX1-DT|LINC02454 |
| LHX1-DT|AC092171.4 |
| LHX1-DT|AL158166.1 |
| LHX1-DT|AL365181.2 |
| LHX1-DT|STARD4-AS1 |
| LHX1-DT|LINC01050 |
| LHX1-DT|U62317.4 |
| LHX1-DT|LINC02244 |
| LHX1-DT|AC007497.1 |
| LHX1-DT|AL122125.1 |
| LHX1-DT|AC006206.2 |
| LHX1-DT|AP002907.1 |
| LHX1-DT|AC128709.2 |
| LHX1-DT|AP005482.2 |
| LHX1-DT|LINC01929 |
| LHX1-DT|DLGAP1-AS2 |
| LHX1-DT|ATP1B3-AS1 |
| LHX1-DT|AF165147.1 |
| LHX1-DT|MIR9-3HG |
| LHX1-DT|AC053503.3 |
| LHX1-DT|MIR1-1HG |
| LHX1-DT|AC091563.1 |
| LHX1-DT|AC010789.1 |
| LHX1-DT|LINC02820 |
| LHX1-DT|LINC02100 |
| LHX1-DT|FOXD3-AS1 |
| LHX1-DT|AC021028.1 |
| LHX1-DT|AC026333.4 |
| LHX1-DT|AL596442.2 |
| LHX1-DT|AC019171.1 |
| LHX1-DT|AC009093.1 |
| LHX1-DT|AC010331.1 |
| LHX1-DT|AC048341.2 |
| LHX1-DT|AC016773.2 |
| LHX1-DT|LINC01311 |
| LHX1-DT|CDKN2B-AS1 |
| LHX1-DT|AL031186.1 |
| LHX1-DT|AC114730.3 |
| LHX1-DT|LINC00911 |
| LHX1-DT|AL133215.2 |
| LHX1-DT|AC004034.1 |
| LHX1-DT|TTLL11-IT1 |
| LHX1-DT|AC103563.7 |
| LHX1-DT|AL355488.1 |
| LHX1-DT|AC016877.3 |
| LHX1-DT|AC145207.8 |
| LHX1-DT|C10orf71-AS1 |
| LHX1-DT|ZKSCAN2-DT |
| LHX1-DT|AL499627.1 |
| LHX1-DT|AL157871.2 |
| LHX1-DT|AC020907.4 |
| LHX1-DT|LINC01355 |
| LHX1-DT|FOXP4-AS1 |
| LHX1-DT|AP001029.1 |
| LHX1-DT|TENM3-AS1 |
| LHX1-DT|DDX11-AS1 |
| LHX1-DT|ITGB1-DT |
| LHX1-DT|AL022322.1 |
| LHX1-DT|MAFA-AS1 |
| LHX1-DT|AL355803.1 |
| LHX1-DT|AC134312.5 |
| LHX1-DT|MIR155HG |
| LHX1-DT|AC016735.1 |
| LHX1-DT|AC089983.1 |
| LHX1-DT|YEATS2-AS1 |
| LHX1-DT|AC018716.1 |
| LHX1-DT|AC011462.4 |
| LHX1-DT|AC010731.2 |
| LHX1-DT|LINC00942 |
| LHX1-DT|KDM4A-AS1 |
| LHX1-DT|LINC02544 |
| LHX1-DT|DELEC1 |
| LHX1-DT|AC034213.1 |
| LHX1-DT|AC092119.2 |
| LHX1-DT|AL359881.1 |
| LHX1-DT|AL031600.1 |
| LHX1-DT|STEAP3-AS1 |
| LHX1-DT|AL591501.1 |
| LHX1-DT|SCAT2 |
| LHX1-DT|HOTAIR |
| LHX1-DT|HS1BP3-IT1 |
| LHX1-DT|AC112721.2 |
| LHX1-DT|AL513320.1 |
| LHX1-DT|AP000251.1 |
| LHX1-DT|LINC01508 |
| LHX1-DT|C1RL-AS1 |
| LHX1-DT|LINC01711 |
| LHX1-DT|LINC01063 |
| LHX1-DT|NRIR |
| LHX1-DT|LINC02551 |
| LHX1-DT|AC128709.1 |
| LHX1-DT|AC068473.3 |
| LHX1-DT|AL031670.1 |
| LHX1-DT|LINC02878 |
| LHX1-DT|AC011481.1 |
| LHX1-DT|LINC02446 |
| LHX1-DT|LINC00944 |
| LHX1-DT|TRPM2-AS |
| LHX1-DT|AL592424.1 |
| LHX1-DT|AC019080.4 |
| LHX1-DT|LINC02870 |
| LHX1-DT|LINC01405 |
| LHX1-DT|AC027796.4 |
| LHX1-DT|LINC01269 |
| LHX1-DT|AC004253.1 |
| LHX1-DT|AL355472.3 |
| LHX1-DT|MIR133A1HG |
| LHX1-DT|AL391427.1 |
| LHX1-DT|AL445250.1 |
| LHX1-DT|HOXC-AS1 |
| LHX1-DT|AC015660.2 |
| LHX1-DT|AL445471.1 |
| LHX1-DT|SOCAR |
| LHX1-DT|DBH-AS1 |
| LHX1-DT|AC025176.1 |
| LINC01694|LINC01614 |
| LINC01694|SLCO4A1-AS1 |
| LINC01694|AC011461.1 |
| LINC01694|AC109460.2 |
| LINC01694|LINC01139 |
| LINC01694|AC024592.2 |
| LINC01694|AL139089.1 |
| LINC01694|LINC01322 |
| LINC01694|AC026785.3 |
| LINC01694|ELFN1-AS1 |
| LINC01694|AC012065.3 |
| LINC01694|LINC01305 |
| LINC01694|LINC01943 |
| LINC01694|AC011676.1 |
| LINC01694|AC015878.1 |
| LINC01694|AC009093.2 |
| LINC01694|AC104041.1 |
| LINC01694|AC002401.4 |
| LINC01694|AC104564.3 |
| LINC01694|AL136018.1 |
| LINC01694|AL513327.1 |
| LINC01694|CASC15 |
| LINC01694|LINC01096 |
| LINC01694|SLC12A5-AS1 |
| LINC01694|AC010719.1 |
| LINC01694|Z97653.1 |
| LINC01694|AC013731.1 |
| LINC01694|NCBP2-AS1 |
| LINC01694|AC093788.1 |
| LINC01694|MCCC1-AS1 |
| LINC01694|AC112236.1 |
| LINC01694|LINC02561 |
| LINC01694|AC025154.2 |
| LINC01694|LINC02454 |
| LINC01694|AC092171.4 |
| LINC01694|AL158166.1 |
| LINC01694|AL365181.2 |
| LINC01694|STARD4-AS1 |
| LINC01694|LINC01050 |
| LINC01694|LINC02244 |
| LINC01694|AC007497.1 |
| LINC01694|AL122125.1 |
| LINC01694|AP002907.1 |
| LINC01694|AC128709.2 |
| LINC01694|AP005482.2 |
| LINC01694|LINC01929 |
| LINC01694|AF165147.1 |
| LINC01694|AC053503.3 |
| LINC01694|MIR1-1HG |
| LINC01694|AC010789.1 |
| LINC01694|LINC02820 |
| LINC01694|LINC02100 |
| LINC01694|FOXD3-AS1 |
| LINC01694|AC026333.4 |
| LINC01694|AL596442.2 |
| LINC01694|LINC00707 |
| LINC01694|AC019171.1 |
| LINC01694|AC009093.1 |
| LINC01694|AC010331.1 |
| LINC01694|AC016773.2 |
| LINC01694|LINC01311 |
| LINC01694|CDKN2B-AS1 |
| LINC01694|AL031186.1 |
| LINC01694|AC114730.3 |
| LINC01694|LINC00911 |
| LINC01694|AC004034.1 |
| LINC01694|TTLL11-IT1 |
| LINC01694|AC103563.7 |
| LINC01694|AC016877.3 |
| LINC01694|AC145207.8 |
| LINC01694|C10orf71-AS1 |
| LINC01694|ZKSCAN2-DT |
| LINC01694|AL499627.1 |
| LINC01694|AL157871.2 |
| LINC01694|AC020907.4 |
| LINC01694|LINC01355 |
| LINC01694|FOXP4-AS1 |
| LINC01694|AP001029.1 |
| LINC01694|TENM3-AS1 |
| LINC01694|DDX11-AS1 |
| LINC01694|ITGB1-DT |
| LINC01694|AL022322.1 |
| LINC01694|MAFA-AS1 |
| LINC01694|AL355803.1 |
| LINC01694|MIR155HG |
| LINC01694|AC016735.1 |
| LINC01694|AC089983.1 |
| LINC01694|YEATS2-AS1 |
| LINC01694|AC018716.1 |
| LINC01694|AC011462.4 |
| LINC01694|AC010731.2 |
| LINC01694|LINC00942 |
| LINC01694|LINC02544 |
| LINC01694|DELEC1 |
| LINC01694|AC034213.1 |
| LINC01694|AC092119.2 |
| LINC01694|AL359881.1 |
| LINC01694|AL031600.1 |
| LINC01694|STEAP3-AS1 |
| LINC01694|AL591501.1 |
| LINC01694|SCAT2 |
| LINC01694|HOTAIR |
| LINC01694|HS1BP3-IT1 |
| LINC01694|AC112721.2 |
| LINC01694|AL513320.1 |
| LINC01694|AP000251.1 |
| LINC01694|LINC01508 |
| LINC01694|LINC01711 |
| LINC01694|LINC01063 |
| LINC01694|NRIR |
| LINC01694|LINC02551 |
| LINC01694|AC128709.1 |
| LINC01694|AC068473.3 |
| LINC01694|AL031670.1 |
| LINC01694|LINC02878 |
| LINC01694|AC011481.1 |
| LINC01694|LINC02446 |
| LINC01694|AC104031.1 |
| LINC01694|LINC00944 |
| LINC01694|TRPM2-AS |
| LINC01694|AL592424.1 |
| LINC01694|AC019080.4 |
| LINC01694|LINC01405 |
| LINC01694|AC004253.1 |
| LINC01694|AL355472.3 |
| LINC01694|MIR133A1HG |
| LINC01694|AL391427.1 |
| LINC01694|AL445250.1 |
| LINC01694|HOXC-AS1 |
| LINC01694|AC015660.2 |
| LINC01694|AL445471.1 |
| LINC01694|SOCAR |
| LINC01694|DBH-AS1 |
| LINC01694|AC025176.1 |
| AL590666.2|LINC01614 |
| AL590666.2|SLCO4A1-AS1 |
| AL590666.2|AC132192.2 |
| AL590666.2|FOXD2-AS1 |
| AL590666.2|MELTF-AS1 |
| AL590666.2|AC011461.1 |
| AL590666.2|AC109460.2 |
| AL590666.2|LINC01139 |
| AL590666.2|AL139089.1 |
| AL590666.2|LINC01322 |
| AL590666.2|PCAT7 |
| AL590666.2|LINC00839 |
| AL590666.2|AC012065.3 |
| AL590666.2|LINC01943 |
| AL590666.2|AC011676.1 |
| AL590666.2|AC009093.2 |
| AL590666.2|AC104041.1 |
| AL590666.2|AC008735.2 |
| AL590666.2|AC002401.4 |
| AL590666.2|AC008115.3 |
| AL590666.2|AL136018.1 |
| AL590666.2|AL513327.1 |
| AL590666.2|CASC15 |
| AL590666.2|KCNMB2-AS1 |
| AL590666.2|AL365181.3 |
| AL590666.2|AC010719.1 |
| AL590666.2|Z97653.1 |
| AL590666.2|C5orf66-AS1 |
| AL590666.2|AC093788.1 |
| AL590666.2|AC026740.1 |
| AL590666.2|AC112236.1 |
| AL590666.2|TBILA |
| AL590666.2|LINC02561 |
| AL590666.2|LINC02454 |
| AL590666.2|AC092171.4 |
| AL590666.2|AL158166.1 |
| AL590666.2|STARD4-AS1 |
| AL590666.2|U62317.4 |
| AL590666.2|AL122125.1 |
| AL590666.2|AC006206.2 |
| AL590666.2|AP002907.1 |
| AL590666.2|AC128709.2 |
| AL590666.2|AP005482.2 |
| AL590666.2|LINC01929 |
| AL590666.2|DLGAP1-AS2 |
| AL590666.2|ATP1B3-AS1 |
| AL590666.2|MIR9-3HG |
| AL590666.2|MIR1-1HG |
| AL590666.2|AC091563.1 |
| AL590666.2|AL021807.1 |
| AL590666.2|AC010789.1 |
| AL590666.2|LINC02820 |
| AL590666.2|LINC02100 |
| AL590666.2|AL035461.3 |
| AL590666.2|FOXD3-AS1 |
| AL590666.2|AC021028.1 |
| AL590666.2|ZNF710-AS1 |
| AL590666.2|AC026333.4 |
| AL590666.2|LINC00707 |
| AL590666.2|AC114956.1 |
| AL590666.2|AC009093.1 |
| AL590666.2|AC010331.1 |
| AL590666.2|AC048341.2 |
| AL590666.2|AC016773.2 |
| AL590666.2|LINC01311 |
| AL590666.2|CDKN2B-AS1 |
| AL590666.2|AL031186.1 |
| AL590666.2|LINC00911 |
| AL590666.2|AC016394.3 |
| AL590666.2|AL133215.2 |
| AL590666.2|AC004034.1 |
| AL590666.2|AC055822.1 |
| AL590666.2|C5orf34-AS1 |
| AL590666.2|TTLL11-IT1 |
| AL590666.2|AL355488.1 |
| AL590666.2|AC016877.3 |
| AL590666.2|AC145207.8 |
| AL590666.2|ZKSCAN2-DT |
| AL590666.2|AL157871.2 |
| AL590666.2|AC020907.4 |
| AL590666.2|LINC01355 |
| AL590666.2|TENM3-AS1 |
| AL590666.2|DDX11-AS1 |
| AL590666.2|ITGB1-DT |
| AL590666.2|AL022322.1 |
| AL590666.2|MAFA-AS1 |
| AL590666.2|AL355803.1 |
| AL590666.2|AC134312.5 |
| AL590666.2|MIR155HG |
| AL590666.2|AL928654.2 |
| AL590666.2|YEATS2-AS1 |
| AL590666.2|AC018716.1 |
| AL590666.2|AC011462.4 |
| AL590666.2|LINC00942 |
| AL590666.2|KDM4A-AS1 |
| AL590666.2|LINC02544 |
| AL590666.2|LINC00941 |
| AL590666.2|DELEC1 |
| AL590666.2|AC034213.1 |
| AL590666.2|AL132712.1 |
| AL590666.2|AL031600.1 |
| AL590666.2|SCAT2 |
| AL590666.2|HS1BP3-IT1 |
| AL590666.2|AC112721.2 |
| AL590666.2|AL513320.1 |
| AL590666.2|AP000251.1 |
| AL590666.2|C1RL-AS1 |
| AL590666.2|LINC01711 |
| AL590666.2|LINC01063 |
| AL590666.2|TMPO-AS1 |
| AL590666.2|NRIR |
| AL590666.2|LINC02551 |
| AL590666.2|NKILA |
| AL590666.2|AC005392.2 |
| AL590666.2|MSC-AS1 |
| AL590666.2|AL031670.1 |
| AL590666.2|LINC02878 |
| AL590666.2|LINC02446 |
| AL590666.2|AC104031.1 |
| AL590666.2|LINC00944 |
| AL590666.2|TRPM2-AS |
| AL590666.2|AC019080.4 |
| AL590666.2|LINC02870 |
| AL590666.2|LINC01405 |
| AL590666.2|AC027796.4 |
| AL590666.2|LINC01269 |
| AL590666.2|AC004253.1 |
| AL590666.2|TYMSOS |
| AL590666.2|AL391427.1 |
| AL590666.2|AL445250.1 |
| AL590666.2|HOXC-AS1 |
| AL590666.2|AC015660.2 |
| AL590666.2|AC012073.1 |
| AL590666.2|SOCAR |
| AL590666.2|DBH-AS1 |
| AL590666.2|AC025176.1 |
| LINC01614|SLCO4A1-AS1 |
| LINC01614|AC132192.2 |
| LINC01614|FOXD2-AS1 |
| LINC01614|MELTF-AS1 |
| LINC01614|AC011461.1 |
| LINC01614|AC109460.2 |
| LINC01614|LINC01139 |
| LINC01614|AC024592.2 |
| LINC01614|AL139089.1 |
| LINC01614|LINC01322 |
| LINC01614|PCAT7 |
| LINC01614|LINC00839 |
| LINC01614|ELFN1-AS1 |
| LINC01614|AC012065.3 |
| LINC01614|LINC01305 |
| LINC01614|LINC01943 |
| LINC01614|AC011676.1 |
| LINC01614|AC009093.2 |
| LINC01614|AC104041.1 |
| LINC01614|AC008735.2 |
| LINC01614|AC002401.4 |
| LINC01614|AC008115.3 |
| LINC01614|AC104564.3 |
| LINC01614|AL136018.1 |
| LINC01614|AL513327.1 |
| LINC01614|CASC15 |
| LINC01614|KCNMB2-AS1 |
| LINC01614|LINC01096 |
| LINC01614|AL365181.3 |
| LINC01614|SLC12A5-AS1 |
| LINC01614|AC010719.1 |
| LINC01614|Z97653.1 |
| LINC01614|AC013731.1 |
| LINC01614|C5orf66-AS1 |
| LINC01614|NCBP2-AS1 |
| LINC01614|AC093788.1 |
| LINC01614|AC026740.1 |
| LINC01614|NCK1-DT |
| LINC01614|MCCC1-AS1 |
| LINC01614|AC112236.1 |
| LINC01614|TBILA |
| LINC01614|LINC02561 |
| LINC01614|AC025154.2 |
| LINC01614|LINC02454 |
| LINC01614|AC092171.4 |
| LINC01614|AL158166.1 |
| LINC01614|AL365181.2 |
| LINC01614|STARD4-AS1 |
| LINC01614|LINC01050 |
| LINC01614|U62317.4 |
| LINC01614|LINC02244 |
| LINC01614|AC007497.1 |
| LINC01614|AL122125.1 |
| LINC01614|AC006206.2 |
| LINC01614|AP002907.1 |
| LINC01614|AC128709.2 |
| LINC01614|AP005482.2 |
| LINC01614|DLGAP1-AS2 |
| LINC01614|ATP1B3-AS1 |
| LINC01614|AF165147.1 |
| LINC01614|MIR9-3HG |
| LINC01614|AC091563.1 |
| LINC01614|AL021807.1 |
| LINC01614|AC010789.1 |
| LINC01614|LINC02820 |
| LINC01614|LINC02100 |
| LINC01614|AL035461.3 |
| LINC01614|FOXD3-AS1 |
| LINC01614|AC021028.1 |
| LINC01614|ZNF710-AS1 |
| LINC01614|AC026333.4 |
| LINC01614|AL596442.2 |
| LINC01614|LINC00707 |
| LINC01614|AC114956.1 |
| LINC01614|AC019171.1 |
| LINC01614|AC009093.1 |
| LINC01614|AC010331.1 |
| LINC01614|AC048341.2 |
| LINC01614|AC016773.2 |
| LINC01614|LINC01311 |
| LINC01614|CDKN2B-AS1 |
| LINC01614|AL031186.1 |
| LINC01614|AC114730.3 |
| LINC01614|LINC00911 |
| LINC01614|AC016394.3 |
| LINC01614|AL133215.2 |
| LINC01614|AC004034.1 |
| LINC01614|AC055822.1 |
| LINC01614|C5orf34-AS1 |
| LINC01614|TTLL11-IT1 |
| LINC01614|AC103563.7 |
| LINC01614|AL355488.1 |
| LINC01614|AC016877.3 |
| LINC01614|AC145207.8 |
| LINC01614|ZKSCAN2-DT |
| LINC01614|AL157871.2 |
| LINC01614|AC020907.4 |
| LINC01614|LINC01355 |
| LINC01614|FOXP4-AS1 |
| LINC01614|AP001029.1 |
| LINC01614|TENM3-AS1 |
| LINC01614|DDX11-AS1 |
| LINC01614|ITGB1-DT |
| LINC01614|AL022322.1 |
| LINC01614|MAFA-AS1 |
| LINC01614|AL355803.1 |
| LINC01614|AC134312.5 |
| LINC01614|MIR155HG |
| LINC01614|AC016735.1 |
| LINC01614|AC089983.1 |
| LINC01614|AL928654.2 |
| LINC01614|YEATS2-AS1 |
| LINC01614|AC018716.1 |
| LINC01614|AC011462.4 |
| LINC01614|LINC02560 |
| LINC01614|AC010731.2 |
| LINC01614|LINC00942 |
| LINC01614|KDM4A-AS1 |
| LINC01614|LINC02544 |
| LINC01614|LINC00941 |
| LINC01614|DELEC1 |
| LINC01614|AC034213.1 |
| LINC01614|AL132712.1 |
| LINC01614|AC092119.2 |
| LINC01614|AL359881.1 |
| LINC01614|AL031600.1 |
| LINC01614|STEAP3-AS1 |
| LINC01614|AL591501.1 |
| LINC01614|SCAT2 |
| LINC01614|HOTAIR |
| LINC01614|HS1BP3-IT1 |
| LINC01614|AL513320.1 |
| LINC01614|AP000251.1 |
| LINC01614|LINC01508 |
| LINC01614|C1RL-AS1 |
| LINC01614|LINC01711 |
| LINC01614|LINC01063 |
| LINC01614|TMPO-AS1 |
| LINC01614|NRIR |
| LINC01614|LINC02551 |
| LINC01614|NKILA |
| LINC01614|AC005392.2 |
| LINC01614|AC128709.1 |
| LINC01614|MSC-AS1 |
| LINC01614|AC068473.3 |
| LINC01614|AL031670.1 |
| LINC01614|LINC02878 |
| LINC01614|AC011481.1 |
| LINC01614|LINC02446 |
| LINC01614|AC104031.1 |
| LINC01614|LINC00944 |
| LINC01614|TRPM2-AS |
| LINC01614|AL592424.1 |
| LINC01614|AC019080.4 |
| LINC01614|LINC02870 |
| LINC01614|LINC01405 |
| LINC01614|AC027796.4 |
| LINC01614|LINC01269 |
| LINC01614|LINC00519 |
| LINC01614|AC004253.1 |
| LINC01614|AL355472.3 |
| LINC01614|TYMSOS |
| LINC01614|AL391427.1 |
| LINC01614|AL445250.1 |
| LINC01614|HOXC-AS1 |
| LINC01614|AC015660.2 |
| LINC01614|AL445471.1 |
| LINC01614|AC012073.1 |
| LINC01614|SOCAR |
| LINC01614|DBH-AS1 |
| LINC01614|AC025176.1 |
| SLCO4A1-AS1|AC132192.2 |
| SLCO4A1-AS1|AC011461.1 |
| SLCO4A1-AS1|AC109460.2 |
| SLCO4A1-AS1|LINC01139 |
| SLCO4A1-AS1|AC024592.2 |
| SLCO4A1-AS1|AL139089.1 |
| SLCO4A1-AS1|LINC01322 |
| SLCO4A1-AS1|AC026785.3 |
| SLCO4A1-AS1|PCAT7 |
| SLCO4A1-AS1|LINC00839 |
| SLCO4A1-AS1|ELFN1-AS1 |
| SLCO4A1-AS1|AC012065.3 |
| SLCO4A1-AS1|LINC01305 |
| SLCO4A1-AS1|LINC01943 |
| SLCO4A1-AS1|AC011676.1 |
| SLCO4A1-AS1|AC015878.1 |
| SLCO4A1-AS1|AC009093.2 |
| SLCO4A1-AS1|AC104041.1 |
| SLCO4A1-AS1|AC002401.4 |
| SLCO4A1-AS1|AC008115.3 |
| SLCO4A1-AS1|AC104564.3 |
| SLCO4A1-AS1|AL136018.1 |
| SLCO4A1-AS1|AL513327.1 |
| SLCO4A1-AS1|CASC15 |
| SLCO4A1-AS1|KCNMB2-AS1 |
| SLCO4A1-AS1|LINC01096 |
| SLCO4A1-AS1|AL365181.3 |
| SLCO4A1-AS1|SLC12A5-AS1 |
| SLCO4A1-AS1|AC010719.1 |
| SLCO4A1-AS1|Z97653.1 |
| SLCO4A1-AS1|AC013731.1 |
| SLCO4A1-AS1|C5orf66-AS1 |
| SLCO4A1-AS1|NCBP2-AS1 |
| SLCO4A1-AS1|AC093788.1 |
| SLCO4A1-AS1|MCCC1-AS1 |
| SLCO4A1-AS1|AC112236.1 |
| SLCO4A1-AS1|TBILA |
| SLCO4A1-AS1|LINC02561 |
| SLCO4A1-AS1|LINC02454 |
| SLCO4A1-AS1|AC092171.4 |
| SLCO4A1-AS1|AL158166.1 |
| SLCO4A1-AS1|AL365181.2 |
| SLCO4A1-AS1|STARD4-AS1 |
| SLCO4A1-AS1|LINC01050 |
| SLCO4A1-AS1|U62317.4 |
| SLCO4A1-AS1|LINC02244 |
| SLCO4A1-AS1|AC007497.1 |
| SLCO4A1-AS1|AL122125.1 |
| SLCO4A1-AS1|AC006206.2 |
| SLCO4A1-AS1|AP002907.1 |
| SLCO4A1-AS1|AC128709.2 |
| SLCO4A1-AS1|AP005482.2 |
| SLCO4A1-AS1|LINC01929 |
| SLCO4A1-AS1|DLGAP1-AS2 |
| SLCO4A1-AS1|ATP1B3-AS1 |
| SLCO4A1-AS1|AF165147.1 |
| SLCO4A1-AS1|MIR9-3HG |
| SLCO4A1-AS1|AC053503.3 |
| SLCO4A1-AS1|MIR1-1HG |
| SLCO4A1-AS1|AC091563.1 |
| SLCO4A1-AS1|AL021807.1 |
| SLCO4A1-AS1|AC010789.1 |
| SLCO4A1-AS1|LINC02820 |
| SLCO4A1-AS1|LINC02100 |
| SLCO4A1-AS1|FOXD3-AS1 |
| SLCO4A1-AS1|AC021028.1 |
| SLCO4A1-AS1|AC026333.4 |
| SLCO4A1-AS1|AL596442.2 |
| SLCO4A1-AS1|LINC00707 |
| SLCO4A1-AS1|AC114956.1 |
| SLCO4A1-AS1|AC019171.1 |
| SLCO4A1-AS1|AC009093.1 |
| SLCO4A1-AS1|AC010331.1 |
| SLCO4A1-AS1|AC048341.2 |
[truncated: 498,918 more chars]
