# Supplementary figures and images for: Identification of Ferroptosis-Related lncRNA Pairs for Predicting the Prognosis of Head and Neck Squamous Cell Carcinoma
Source: J Oncol. 2022 Jul 20;2022:7602482. doi: 10.1155/2022/7602482 (PMC9328971; doi:10.1155/2022/7602482)

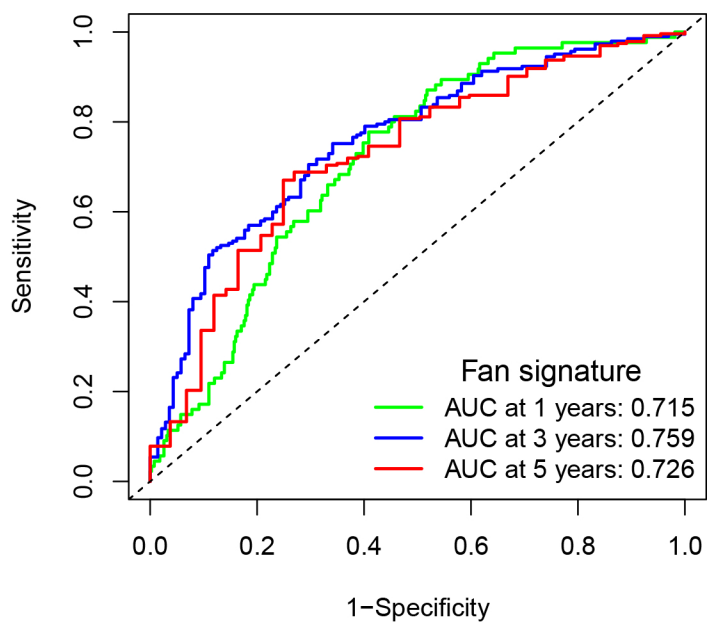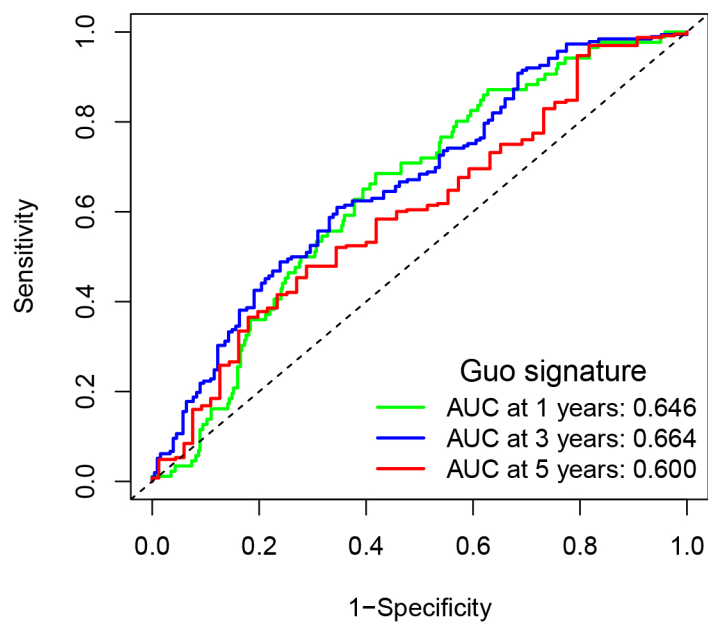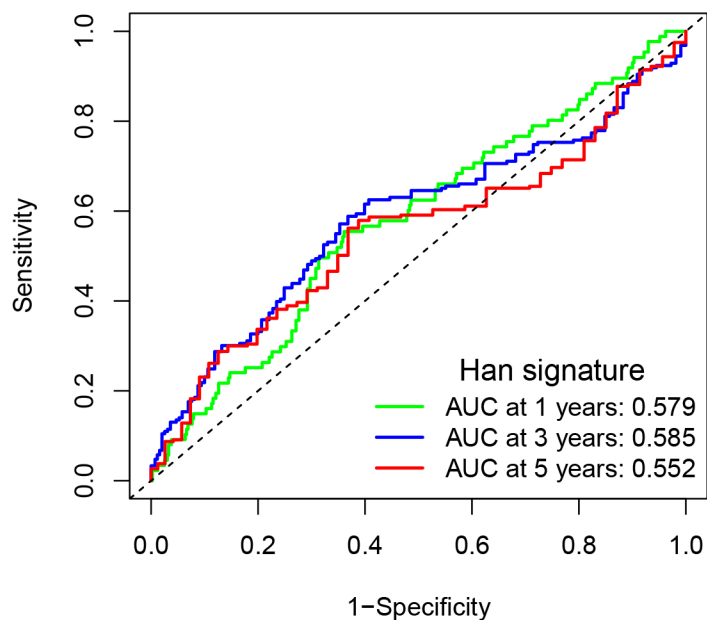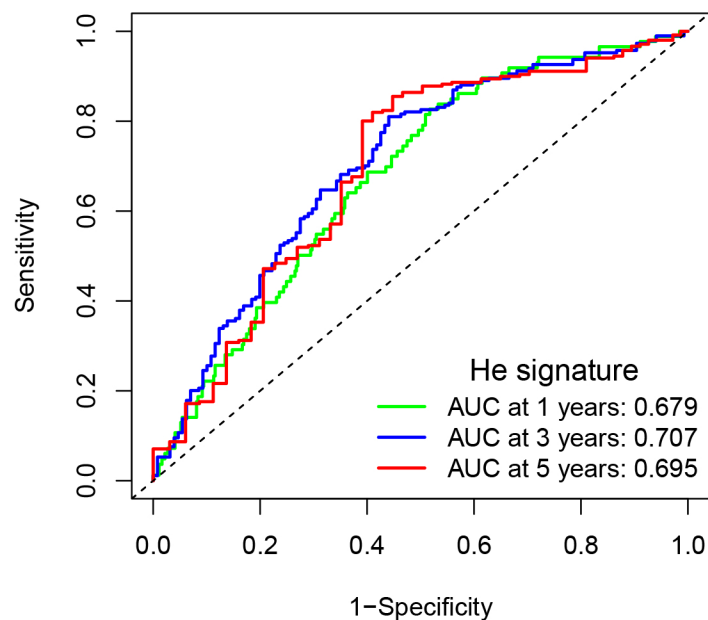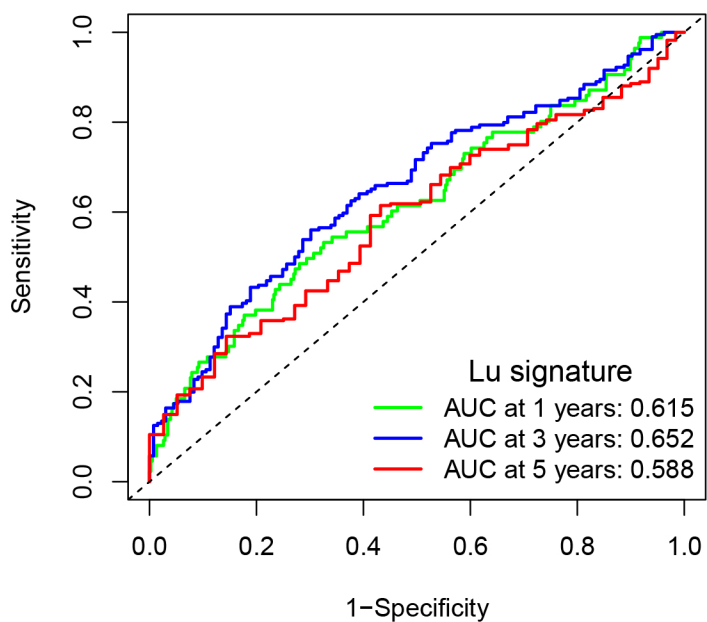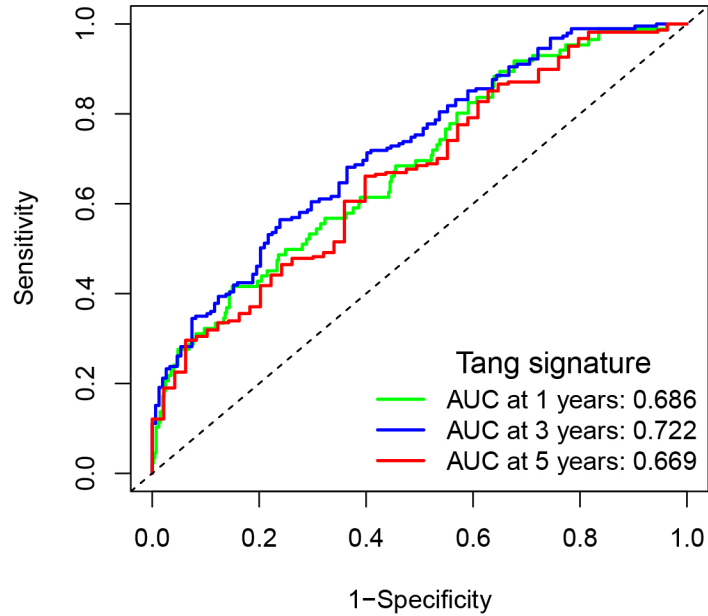

Supplement: Supplementary Materials — Supplementary Figure 1: a comparison of one-, three-, and five-year ROC curves with other established models indicates the superiority of this risk model. Supplementary Table 1: the ferroptosis-related genes. Supplementary Table 2: 722 FRlncRNAs by coexpression analysis. Supplementary Table 3: 196 DEFRlncRNA by differential expression analysis by the fresh pairing algorithm. Supplementary Table 4: 13,444 DEFRlncRNA pairs by differential expression analysis. Supplementary Table 5: 2,753 DEFRlncRNA pairs via univariate Cox regression analysis. Supplementary Table 6: 11 DEFRlncRNA pairs through multivariate Cox regression analysis. Supplementary Table 7: the details of univariate and multivariate Cox regression analysis. Supplementary Table 8: the detailed comparison of the relationship between tumour-infiltrating immune cells and risk sores. [file 7602482.f1.zip › Supplementary Figure 1.pdf]
